# Discovery and SAR Evolution of Pyrazole Azabicyclo[3.2.1]octane Sulfonamides as a Novel Class of Non-Covalent *N*-Acylethanolamine-Hydrolyzing Acid Amidase (NAAA) Inhibitors for Oral Administration

**DOI:** 10.1021/acs.jmedchem.1c00575

**Published:** 2021-09-01

**Authors:** Paolo Di Fruscia, Anna Carbone, Giovanni Bottegoni, Francesco Berti, Francesca Giacomina, Stefano Ponzano, Chiara Pagliuca, Annalisa Fiasella, Daniela Pizzirani, Jose Antonio Ortega, Andrea Nuzzi, Glauco Tarozzo, Luisa Mengatto, Roberta Giampà, Ilaria Penna, Debora Russo, Elisa Romeo, Maria Summa, Rosalia Bertorelli, Andrea Armirotti, Sine Mandrup Bertozzi, Angelo Reggiani, Tiziano Bandiera, Fabio Bertozzi

**Affiliations:** † D3-PharmaChemistry, Istituto Italiano di Tecnologia (IIT), 16163Genova, Italy; ‡ Department of Biological, Chemical and Pharmaceutical Sciences and Technologies (STEBICEF), University of Palermo, 90123Palermo, Italy; § Computational and Chemical Biology, Istituto Italiano di Tecnologia (IIT), 16163Genova, Italy; ∥ D3-Validation, Istituto Italiano di Tecnologia (IIT), 16163Genova, Italy; ⊥ Analytical Chemistry and Translational Pharmacology, Istituto Italiano di Tecnologia (IIT), 16163Genova, Italy

## Abstract

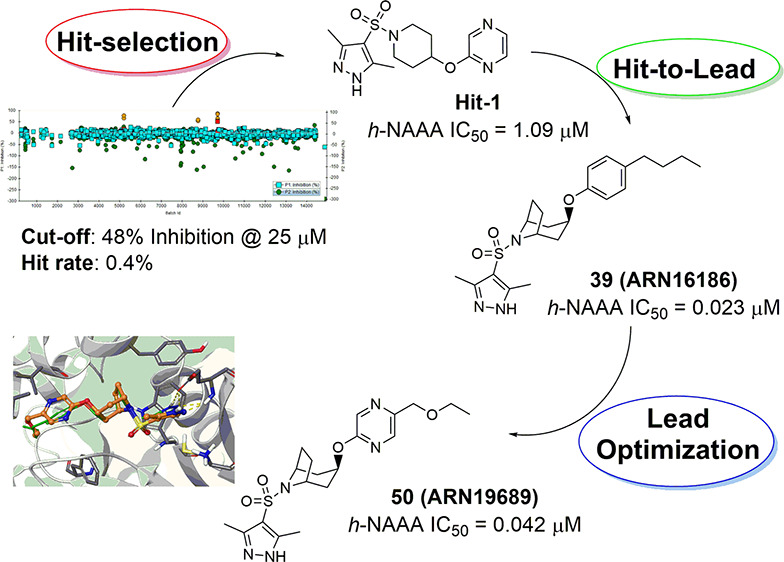

Inhibition of intracellular *N*-acylethanolamine-hydrolyzing acid amidase (NAAA) activity is a promising approach to manage the inflammatory response under disabling conditions. In fact, NAAA inhibition preserves endogenous palmitoylethanolamide (PEA) from degradation, thus increasing and prolonging its anti-inflammatory and analgesic efficacy at the inflamed site. In the present work, we report the identification of a potent, systemically available, novel class of NAAA inhibitors, featuring a pyrazole azabicyclo[3.2.1]octane structural core. After an initial screening campaign, a careful structure–activity relationship study led to the discovery of *endo*-ethoxymethyl-pyrazinyloxy-8-azabicyclo[3.2.1]octane-pyrazole sulfonamide **50** (**ARN19689**), which was found to inhibit human NAAA in the low nanomolar range (IC_50_ = 0.042 μM) with a non-covalent mechanism of action. In light of its favorable biochemical, in vitro and in vivo drug-like profile, sulfonamide **50** could be regarded as a promising pharmacological tool to be further investigated in the field of inflammatory conditions.

## Introduction

Inflammation is a multifaceted, dynamic process inducing a set of reactive modifications that occur in the affected organs and vascular tissue to repair the damage produced by harmful agents or stimuli of various kinds.^1,2^ Chronic inflammation is a long-lasting pathological process determined by the persistence of inflammatory stimuli, and is typically characterized by the infiltration of mononuclear cells (macrophages, lymphocytes, and plasma cells), and the simultaneous presence of tissue damage, angiogenesis, and fibrosis.^3^ Chronic inflammatory diseases can be considered as a leading cause of death in the world today, with more than 50% of all deaths linked to some degree to inflammation-related diseases (e.g., ischemic heart disease, cancer, diabetes mellitus, chronic kidney disease, stroke, and autoimmune and neurodegenerative conditions).^4^

Among the possible ways to manage inflammation, the inhibition of the cysteine hydrolase *N*-acylethanolamine-hydrolyzing acid amidase (NAAA) has been reported as a promising approach to control pain and inflammatory conditions.^5−8^ NAAA, a member of the *N*-terminal nucleophile family of hydrolases (*N*tn hydrolases),^9^ is expressed at high levels in immune cells, mainly in macrophages, and localized in the lysosomal compartment.^6,10^ The enzyme is produced as a precursor and activated by self-proteolysis at acidic pH (pH = 4.5–5) into two chains: α- and β-subunits. In human NAAA (*h*-NAAA), this process exposes the enzyme’s catalytic triad of nucleophilic Cys126, basic Arg142, and acidic Asp145 residues, producing a hydrolysis-competent enzyme.^11^

NAAA is involved in the deactivating hydrolysis of *N*-acylethanolamines (NAEs), a group of endogenous lipid mediators comprising ethanolamine bound to a variable fatty-acid-derived acyl moiety. NAEs play an important role in the control of multiple physiological functions including the regulation of pain,^12^ inflammation,^13^ feeding behavior,^14,15^ and the dopaminergic reward system.^16,17^ Previous studies have shown that NAAA exhibits substrate selectivity predominantly for saturated NAEs, such as palmitoylethanolamide (PEA).^10^ PEA is an anti-inflammatory, analgesic, and neuroprotective agent, mainly involved in the activation of peroxisome proliferator-activated receptor α (PPAR-α), a nuclear receptor, which plays a crucial role in biological processes enhancing the transcription of various anti-inflammatory genes and concomitantly interrupting the activity of proinflammatory transcription factors.^18−20^ PEA signaling activity is terminated by its degradation into ethanolamine and palmitic acid catalyzed preferentially by NAAA, and to a lesser extent by fatty acid amide hydrolase (FAAH),^21^ a membrane-bound serine amidase biologically related to NAAA. Although NAAA and FAAH share a similar mechanism, catalyzing the hydrolysis of *N*-acylethanolamines, they have no sequence homology. On the contrary, NAAA displays high homology to acid ceramidases (AC), another lysosomal cysteine amidase, showing 33–35% amino acid identity in their structures.^9,22^

From a drug discovery perspective, sustaining PEA levels through the inhibition of intracellular NAAA activity represents a promising approach to modulate the inflammatory response, by increasing and/or prolonging PEA’s anti-inflammatory and analgesic properties. Therefore, the use of NAAA inhibitors can provide a certain efficacy in the treatment of inflammatory conditions without causing the typical side effects due to generalized stimulation of PPAR-α, as with exogenous agonists.^23^

**Figure 1:**
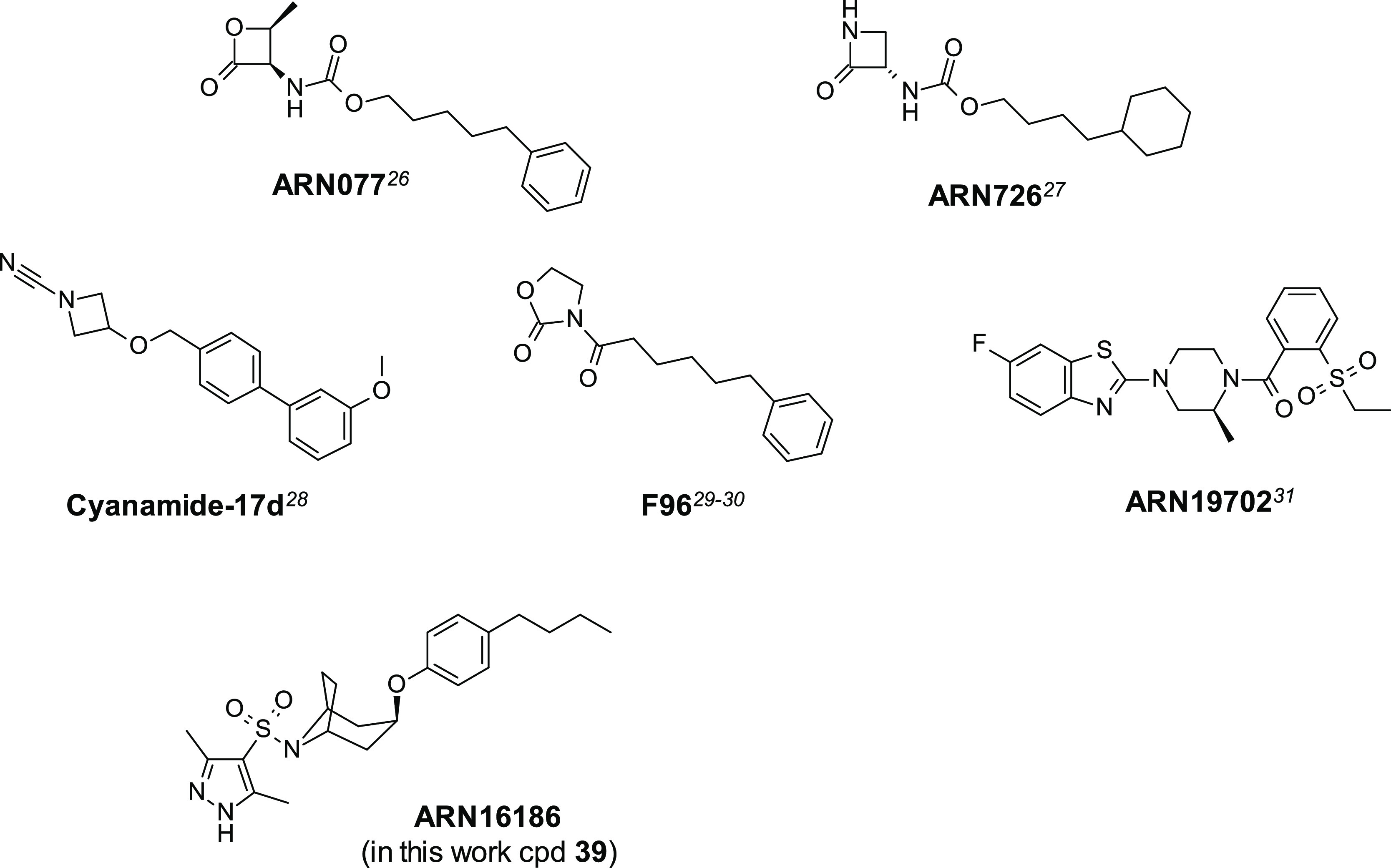
Representative structures of potent rat and human NAAA inhibitors.

Interestingly, it has been reported that alterations of PEA levels are found in inflammatory diseases, such as rheumatoid arthritis, osteoarthritis,^24^ and in cerebrospinal and plasma fluids in MS patients.^25^ Despite the encouraging pharmacological benefits achieved by restoring intracellular PEA levels through NAAA inhibition, only a few chemical classes of potent compounds have been identified (Figure 1).^26−31^

Over the last decade, few research groups have been involved in the discovery of novel NAAA inhibitors.^7,8^ In this context, our work led to the identification of low nanomolar inhibitors, featuring electrophilic warheads, as possible therapeutic agents suited for topical (***ARN077*** and analogues, Figure 1)^26,32^ and/or systemic administration (***ARN726*** and analogues, Figure 1).^27,33^ Depending on the nature of the small heterocyclic reactive moiety, experimental investigations showed for these chemotypes a covalent, partially reversible or irreversible mechanism of action both in vitro and in vivo. Lately, in order to avoid the possible drawbacks of covalent inhibition (i.e., idiosyncratic effects, dosing regimen, etc*.*),^34,35^ the identification of non-covalent and systemically available NAAA inhibitors was recently reported and demonstrated to be beneficial in a MS mouse model (**ARN19702**, Figure 1).^31,36^ Indeed, it was shown that NAAA contributed to disease progression in the experimental autoimmune encephalomyelitis (EAE) mouse model^37^ because induction of the enzyme’s expression was observed in the spinal cord of EAE-affected mice.^36^

In the present work, we report on the discovery of potent, systemically available pyrazole azabicyclooctane sulfonamide derivatives, as a novel class of NAAA inhibitors, endowed with a non-covalent mechanism of action. A biological screening, by the use of a fluorogenic human NAAA assay, allowed identifying few primary hits featuring a similar chemotype. An in-depth structure–activity relationship (SAR) analysis led initially to the discovery of lead compound **39** (**ARN16186**, Figure 1)^38^ with high inhibitory activity and good preliminary pharmacokinetic properties.^39^ Structural modifications to improve the physicochemical and drug-like properties of **39** led eventually to the identification of *endo*-ethoxymethyl-pyrazinyloxy-8-azabicyclo[3.2.1]octane-pyrazole sulfonamide **50** (**ARN19689**, Table 4), a compound with a superior pharmacological and pharmacokinetic profile. Due to its high inhibitory activity and selectivity for NAAA, supported by the in vitro biochemical and in vivo pharmacological data, novel azabicyclic compound **50** could represent a valuable tool to be further evaluated in the management of inflammatory conditions.

## Results and Discussion

**Figure 2:**
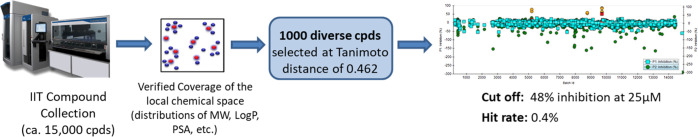
Selection of a diversity set of small molecules from our compound library and medium throughput screening (MTS) outcome. A hierarchical agglomerative cluster analysis procedure on our internal compound collection of 13,289 entries was carried out. Molecules are expressed as ECFP4^40^ fingerprints and distances between fingerprints were estimated in terms of Tanimoto similarity. The agglomerative process was arbitrarily terminated when 1000 clusters were generated (corresponding to an intracluster Tanimoto distance of 0.462). Each centroid was selected as representative of its entire cluster.

In early drug discovery, the screening of mid-large compound collections is a key activity to help identifying and/or expanding the portfolio of novel, active chemotypes on biological targets. In an effort to discover novel non-covalent human NAAA (*h*-NAAA) inhibitors, a small tailored set of 1000 compounds was selected from our internal collection of commercially available small molecules. These compounds were identified based on diversity in structural and physicochemical properties, and were tested via a medium throughput screening (MTS) fluorogenic *h*-NAAA assay (Figure 2).

**Figure 3:**
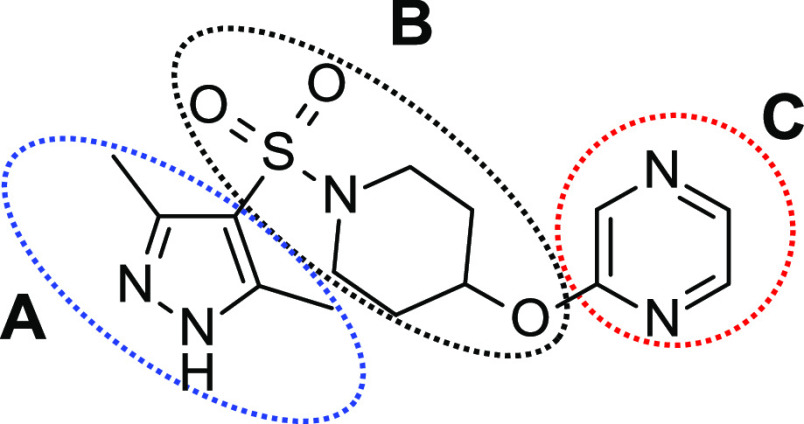
Chemical structure of MTS hit **1**. The regions of SAR explorations are indicated.

The MTS campaign identified a few hits belonging to structurally diverse chemical classes, showing promising inhibitory activity in the micromolar range. An accurate analysis highlighted the presence of compounds featuring a similar chemotype, lacking highly reactive/electrophilic chemical moieties, and therefore potentially matching our purpose to discover non-covalent inhibitors. Ultimately, we decided to focus our optimization efforts on hit compound **1**,^41^ which showed, along with a promising, initial single-digit micromolar inhibitory activity against *h*-NAAA (IC_50_ = 1.09 μM, Table 1), structural novelty, synthetic feasibility, and an encouraging lipophilic efficiency (LipE: 6.3)^42,43^ (Figure 3). From a preliminary structural analysis, hit **1** was envisaged as a reasonable starting point for the identification of novel *h*-NAAA inhibitors, featuring a non-covalent mechanism of action, as it does not contain known reactive moieties. Driven by these initial observations, we developed a structure–activity relationship (SAR) study by means of iterative designing, synthesis, and biological characterization cycles of analogues of sulfonamide **1**. Chemical investigations were undertaken exploring rationally the key structural regions in primary hit **1**, that is, the type and the substitution pattern of heteroaromatic moiety (***A***), the functionalization and structural changes of piperidine (***B***), and finally the modifications of the heteroaryl group (***C***) (Figure 3).

**Table 1:**
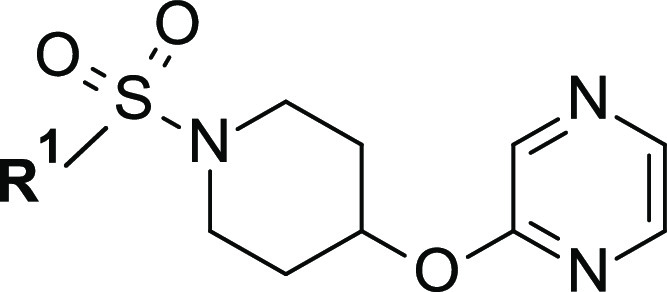

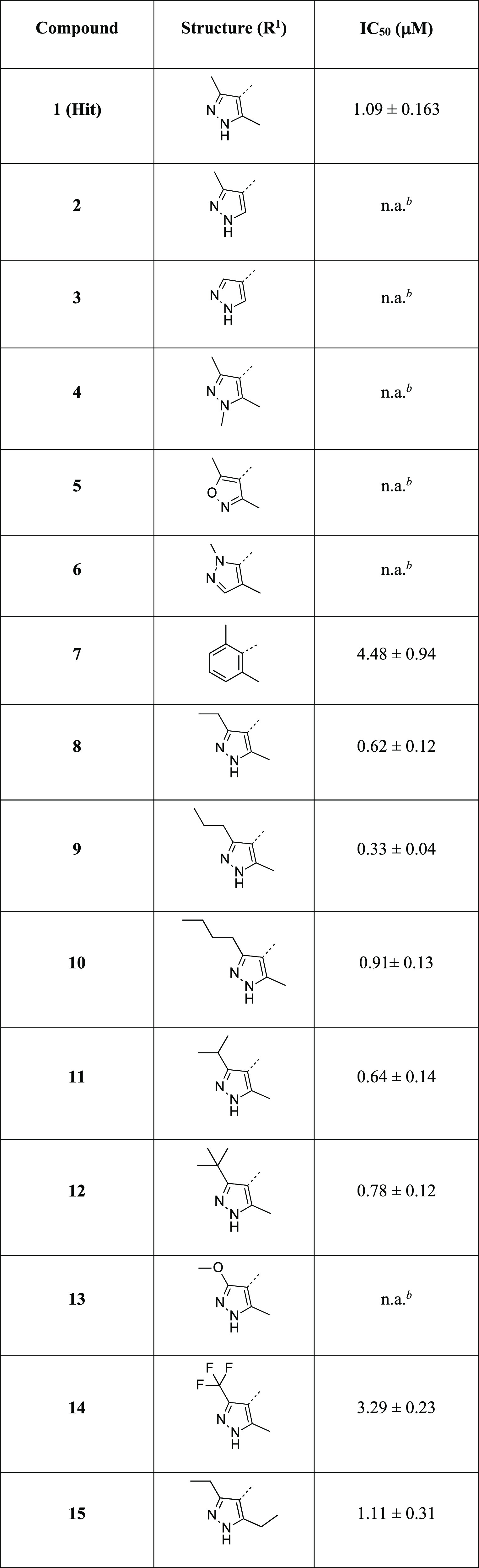
Structure and *h*-NAAA Inhibitory Activity (IC_50_) of Compounds **1–15**^a^

a *h*-NAAA (fluo.), data are expressed as mean ± SD (*n* ≥ 3).

b n.a.: not active @50 μM (<10% inhibition).

We started our SAR investigation exploring the pyrazole region ***A*** of compound **1** (Table 1). To gain broad structural information, a variety of substituted aromatic and heteroaromatic moieties were introduced as an alternative to the 3,5-dimethylpyrazole group.

Initially, we focused our study on 5-membered heteroaromatic rings having different substitution patterns. The effect of the 3,5-dialkyl substitution on the pyrazole ring resulted to be important for activity because the corresponding mono- (**2**) or unsubstituted (**3**) analogues turned out to be devoid of any activity with respect to dimethyl-pyrazole derivative **1** (Table 1). Similarly, the absence of any hydrogen bond donor, as for 1,3,5-trimethyl- (**4**), 1,2-isoxazolyl- (**5**), and regioisomeric 1,3-dimethyl- (**6**) analogues led to a complete loss of inhibitory effect, thus suggesting the crucial requirement of a hydrogen bond donor feature in that specific region of the protein pocket (Table 1). The low inhibitory activity of the 1,3-dimethyl phenyl analog (**7**) could be ascribed to either the lack of a hydrogen bond donor or alternatively due to the different stereoelectronic properties of the phenyl ring with respect to the five-membered heterocycles.

The influence on activity of aliphatic substituents with increasing length or bulkiness was also investigated. Extending the size of one of the alkyl groups was reasonably well tolerated, the corresponding 5-ethyl (**8**, IC_50_ = 0.62 μM), 5-*n*-butyl (**10**, IC_50_ = 0.91 μM), 5-*iso*-propyl (**11**, IC_50_ = 0.64 μM), and 5-*tert*-butyl (**12**, IC_50_ = 0.78 μM) derivatives being active in the submicromolar range. Interestingly, among this set of sulfonamides, an *n*-propyl chain in position 5 of the pyrazole, as in compound **9**, furnished a new analogue with a 3-fold higher potency (IC_50_ = 0.33 μM) compared to hit **1** (Table 1). These data could indicate the presence of a lipophilic pocket accommodating an aliphatic side chain of a specific size and bulkiness, in either the 3- or 5-position of the pyrazole. To further elucidate whether an increase of lipophilicity in both positions would favor the inhibitory effect against NAAA, the corresponding 3,5-diethyl substitution, as in pyrazole sulfonamide **15**, was investigated. However, the compound showed an about 2-fold drop in activity (IC_50_ = 1.11 μM) with respect to the corresponding 3-methyl-5-ethyl derivative **8**.

Electronic properties of the pyrazole substituents also seemed to have a significant impact on potency. Indeed, while still keeping a 5-methyl residue, an electron-withdrawing trifluoromethyl (**14**) or an electron-donating methoxy (**13**) group in the 3-position resulted in either a drop in efficacy with inhibition in the micromolar range (IC_50_ = 3.29 μM) or no detectable activity, respectively (Table 1).

We then conveyed our attention to the modification and functionalization of the piperidine heterocycle connecting the pyrazole ring to the pyrazine (***B***, Figure 3). While keeping unmodified the rest of the molecule in hit **1**, the key role of the tertiary sulfonamide was confirmed by the complete loss of activity shown by the amide analogue **16**. A similar outcome was found with the secondary sulfonamide **17**, featuring a functionalization of an exocyclic amino group on a cyclohexyl moiety (Table 2).

**Table 2:**
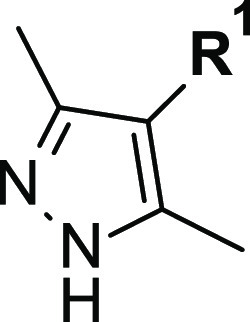

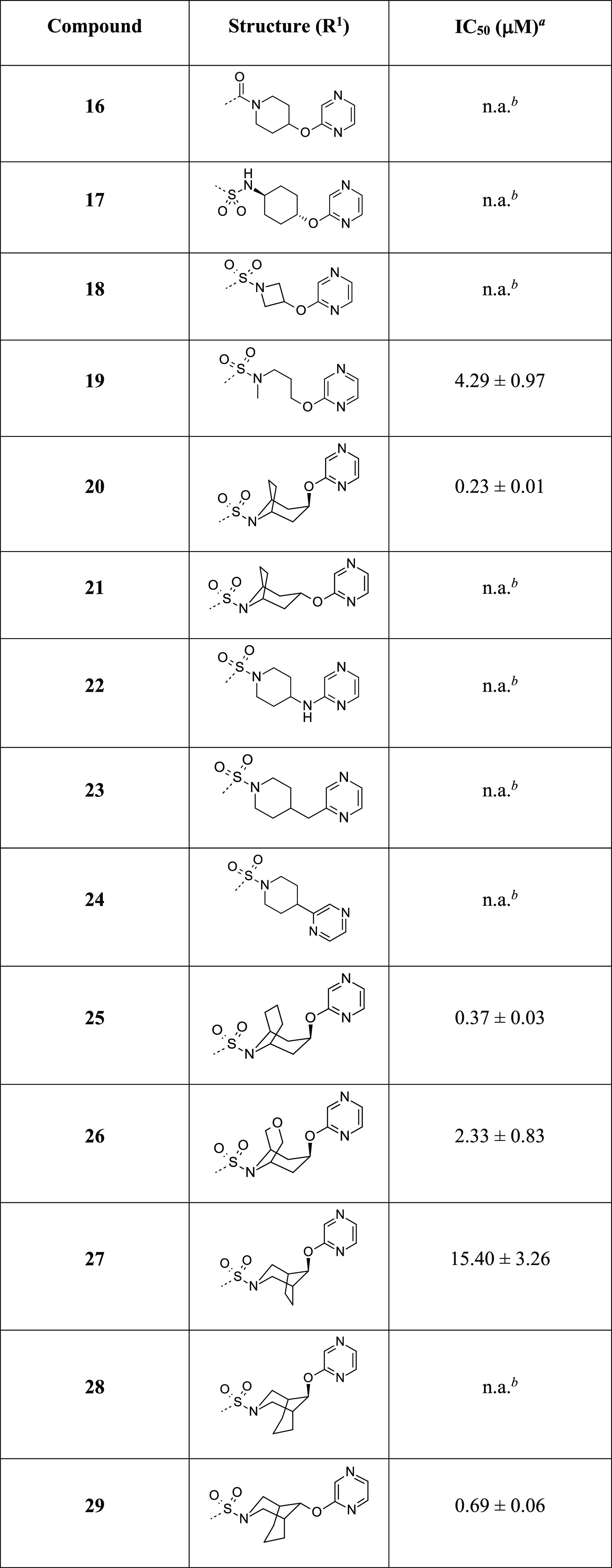
Structure and *h*-NAAA Inhibitory Activity (IC_50_) of Pyrazole-Substituted Compounds **16–29**^a^

a *h*-NAAA (fluo.), data are expressed as mean ± SD (*n* ≥ 3).

b n.a.: not active @50 μM (<10% inhibition).

Next, we explored alternatives to the piperidinyl moiety via ring morphing and scaffold hopping. We first prepared and evaluated the azetidine sulfonamide **18**, as a ring contraction analogue of **1**, which resulted in a complete loss of enzyme inhibition. The ring opening of the piperidine moiety was also investigated to determine the effect of more flexible substituents, leading to the corresponding acyclic tertiary sulfonamide **19**. This modification produced a drop of activity (IC_50_ = 4.29 μM), presumably caused by an increased entropic penalty of binding to the biological target (Table 2).

Finally, we tried to constrain the piperidine core into bridged bicyclic systems by designing, synthesizing, and profiling a series of aliphatic heterocyclic replacements. While ring opening or ring contraction was detrimental for inhibition, constraining the piperidine ring into a more conformationally rigid aza-bridged bicyclic scaffold was found to be beneficial. Sulfonamide analogue **20**, featuring an azabicyclo[3.2.1]octane core, showed submicromolar activity (*h*-NAAA IC_50_ = 0.23 μM, Table 2), with approximately 5-fold boost in potency compared to parent hit **1**. At this stage, the stereochemistry of the ether substitution at the pseudoasymmetric carbon in position 3 of the azabicyclic scaffold was evaluated. Notably, contrary to the beneficial conformation effect seen for the *endo*-isomer **20**, the corresponding *exo*-diastereoisomer **21** turned out to be devoid of any activity toward human NAAA.

As a natural development of our SAR investigation, we replaced the oxygen linker connecting the piperidine core to the pyrazine ring with a nitrogen (**22**) or a methylene unit (**23**). Unfortunately, removal (**24**) or modification (**22**, **23**) of the ether linker abolished the inhibitory activity (Table 2).

Encouraged by the increase in potency shown by *endo*-substituted tropyl sulfonamide **20**, we decided to prepare and profile additional bridged aza-bicyclic systems, varying the size, the lipophilic nature, and the position of the bridge on the piperidine ring. While expanding the bridge up to three methylene units, the activity remained in the submicromolar range (**25**, *h*-NAAA IC_50_ = 0.37 μM), the introduction of an oxygen caused a significant 10-fold drop of the inhibitory effect (**26**, *h*-NAAA IC_50_ = 2.33 μM). This outcome may be rationalized by hypothesizing that the bridged aza-bicyclic system is well accommodated into a lipophilic cleft within the *h*-NAAA active site (vide infra, the docking section).

Moving the bridge in proximity to the pyrazinyloxy substituent, the *endo*-isomer lost efficacy, providing sulfonamide **27** with only double-digit micromolar activity (IC_50_ = 15.4 μM). Bridge expansion to three carbon atoms was again tolerated, depending on the stereochemistry. Interestingly, in this case, the *endo*-isomer **28** was found to be inactive, while the corresponding *exo*-diastereoisomer **29** maintained some inhibitory effect (*h*-NAAA IC_50_ = 0.69 μM), with a marginal loss compared to *endo*-substituted tropyl analogue **20** (Table 2).

Taken together, these data suggest that conformational restrictions of the piperidine ring could improve *h*-NAAA inhibition, most likely by minimizing the entropic penalty of binding and increasing lipophilicity. In addition, the bridge seems to be well suited at either the vicinal or distal bridgeheads at the piperidine nitrogen atom, but the geometry of the substituents is crucial for activity. In general, *N*-vicinal bridges require *endo*-stereochemistry to produce the most favorable inhibitory effect, while *N*-distal bridges seem to be more effective in binding to the *h*-NAAA active site in their *exo*-configuration.

After exploration of the possible modifications on both the pyrazole ring and the piperidine moiety of sulfonamide **1**, having also identified new *h*-NAAA inhibitors with submicromolar affinity for the target, as a final step of our program of structural manipulations, we investigated the relevance of the terminal heteroaryl group (***C***, Figure 3).

Initially, the importance of the nitrogen atoms in the pyrazine ring was assessed by preparing and screening against *h*-NAAA both the corresponding pyridyl (**30**) and phenyl (**31**) analogues of *endo*-substituted sulfonamide **20**. Removal of one nitrogen from the pyrazine core, as for pyridine analogue **30**, showed a slightly improved activity (*h*-NAAA IC_50_ = 0.16 μM), compared to its more polar parent pyrazine **20**. Introducing a more lipophilic moiety, such as a phenyl ring (**31**), improved the potency further, furnishing a new *h*-NAAA inhibitor in the double-digit nanomolar range (IC_50_ = 0.093 μM, Table 3).

**Table 3:**
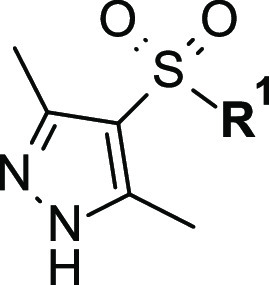

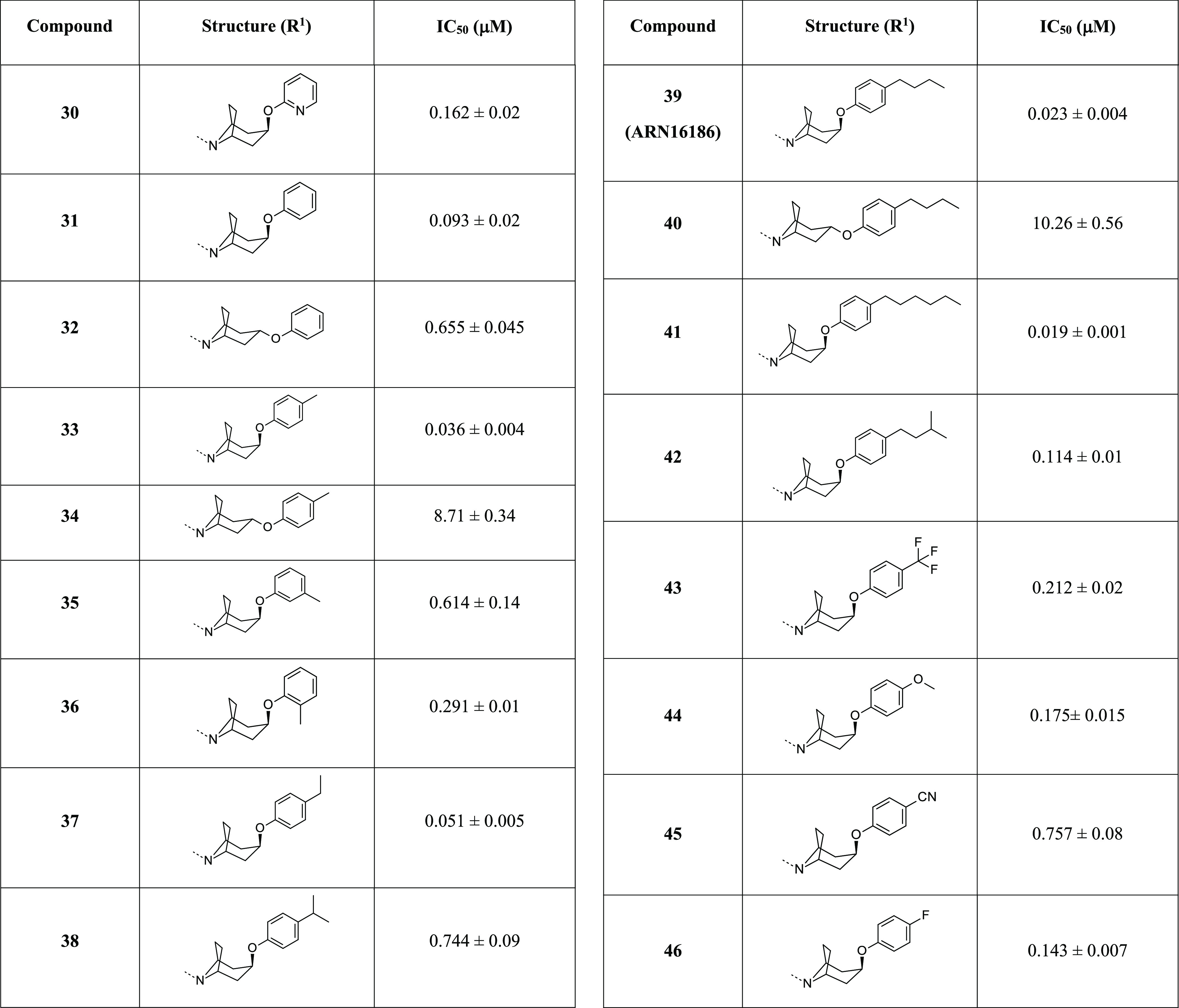
Structure and *h*-NAAA Inhibitory Activity (IC_50_) of Pyrazole Azabicyclo[3.2.1]octane Sulfonamides **30–46**^a^

a *h*-NAAA (fluo.), data are expressed as mean ± SD (*n* ≥ 3).

Consistent with the preferred *endo*-geometry observed for the pyrazine-matched pair *endo*-**20**/*exo*-**21**, the phenoxy derivative **31** was ca. 7-fold more active than its *exo*-diastereoisomer **32** (*h*-NAAA IC_50_ = 0.655 μM). A methyl scan around the phenyl ring was then explored, highlighting the *para* position as the preferred vector for further growing. In fact, while the *para*-methyl-substituted phenoxy sulfonamide **33** displayed a very promising inhibitory potency in the low double-digit nanomolar range (IC_50_ = 0.036 μM), the corresponding *ortho*- (**36**, IC_50_ = 0.291 μM) and *meta*- (**35**, IC_50_ = 0.614 μM) methyl-phenoxy analogues showed from 8- to 17-fold drop in activity, respectively (Table 3). The stereochemistry effect in terms of *h*-NAAA inhibition at the pseudoasymmetric carbon in position 3 on the azabicyclic system was further confirmed for the *para*-methyl phenoxy sulfonamide **34**. This compound, featuring an *exo*-configuration, was shown to inhibit *h*-NAAA only with a modest micromolar efficacy (IC_50_ = 8.71 μM).

Based on these new findings, a number of linear and branched 4-alkyl derivatives were prepared and screened. In general, linear alkyl chains, such as ethyl (**37**, IC_50_ = 0.051 μM), *n*-butyl (**39**, IC_50_ = 0.023 μM), and *n*-hexyl (**41**, IC_50_ = 0.019 μM), on the phenyl ring connected to the azabicyclic sulfonamide portion, were found to furnish more potent inhibitors compared to branched substituents (*iso*-propyl, **38** and *iso*-butyl, **42**, *h*-NAAA IC_50_ = 0.744 and 0.114 μM, respectively) (Table 3). Notably, inhibitor **39** (**ARN16186**), with an *endo*-4-*n*-butylphenoxy right-hand side portion, showed a very high inhibitory activity against *h*-NAAA with a striking 450-fold activity boost toward its corresponding *exo*-diastereoisomer **40** (*h*-NAAA, IC_50_ = 10.26 μM).

In addition to simple hydrophobic substituents, other small functionalities were investigated at the *para* position of the phenyl group, featuring an *endo*-configuration at the 3 position in the tropyl scaffold. While a polar 4-cyano residue (**45**, IC_50_ = 0.757 μM) resulted to be quite detrimental in terms of *h*-NAAA inhibition, the corresponding 4-trifluoromethyl (**43**, IC_50_ = 0.212 μM), 4-methoxy (**44**, IC_50_ = 0.175 μM), or 4-fluoro (**46**, IC_50_ = 0.143 μM) substitutions were found to be tolerated with only a slight loss in activity compared to the double-digit nanomolar methyl derivative **33**.

Having now in hand highly potent inhibitors, we focused our optimization efforts on improving their overall drug-like profile by carefully controlling lipophilicity, increasing lipophilic efficiency (LipE),^42,43^ and trying to balance potency with physicochemical and ADME properties.

Being an oxygen atom tolerated at the *para* position, as seen for methoxy analogue **44** (Table 3), we explored this substitution further by making a couple of extended *O*-alkyl derivatives. First, we hybridized the methoxy residue with the potency-builder *n*-butyl alkyl chain (as in **39**) to obtain a new *n*-propoxy derivative **47** (Table 4). Unfortunately, this modification determined an overall drop in inhibitory activity in the submicromolar range (*h*-NAAA, IC_50_ = 0.45 μM). To further explore the effect of a polar atom in the 4-carbon alkyl tail, we moved the oxygen along the aliphatic chain leading to the ethoxymethyl analogue **48**. This change resulted to be beneficial both in terms of gained affinity toward *h*-NAAA (IC_50_ = 0.016 μM) and improved overall polarity.

**Table 4:**
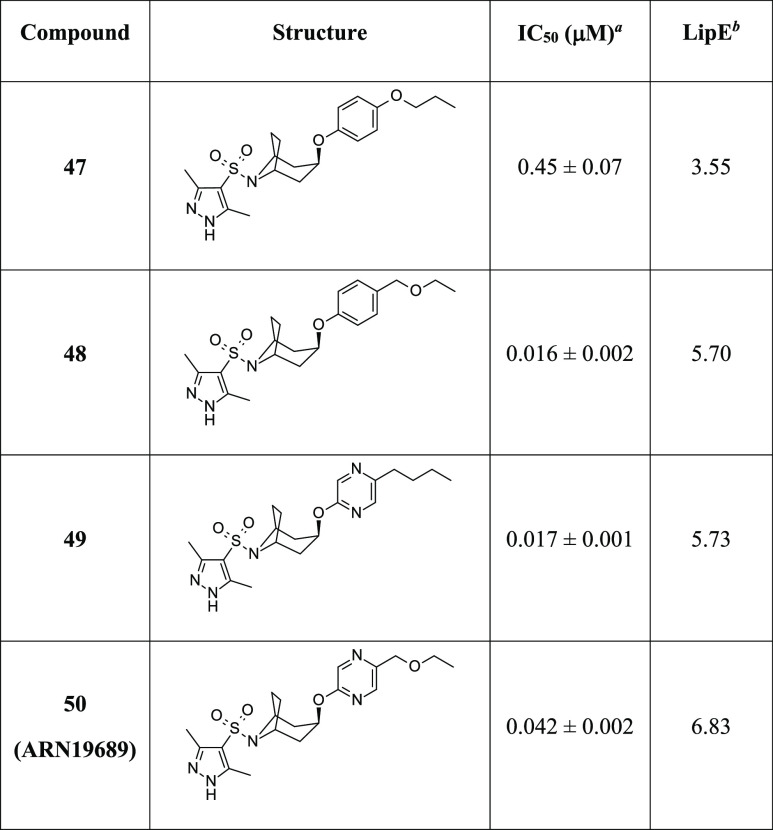
*h*-NAAA Inhibitory Activity (IC_50_) and LipE Values of Novel Pyrazole-Azabicyclic Sulfonamides **47–50**

a *h*-NAAA (fluo), data are expressed as mean ± SD (*n* ≥ 3).

b LipE = pIC_50_ – *c* Log *P* [*c* Log *P* computed using PipelinePilot WebPort 2017] (see also Table S1, Supporting Information).

Taking into account the positive effect on activity shown by the pyrazine ring, as seen for *endo*-substituted tropyl sulfonamide **20**, and aiming to reduce lipophilicity while keeping good *h*-NAAA inhibition (as proven by the *n*-butyl side chain on the aryloxy moiety), we tried to combine both features in an additive manner. Notably, azabicyclic sulfonamide **49** bearing an *endo*-5-*n*-butyl-pyrazyn-2-yloxy substitution was synthesized and tested to display low double-digit nanomolar activity (*h*-NAAA, IC_50_ = 0.017 μM). Intrigued by this last outcome, we ultimately sought to further modulate lipophilicity by incorporating an ethoxymethyl side chain on the pyrazine ring. Along with a substantial contribution to the polarity of the molecule, this modification led to optimized 5-ethoxymethyl-pyrazinyloxy-8-azabicyclo[3.2.1]octane pyrazol-sulfonamide **50** (**ARN19689**, *h*-NAAA, IC_50_ = 0.042 μM, Table 4), as an excellent compromise between reduced lipophilicity and sustained activity compared to *endo*-substituted tropyl derivative **39**.

**Figure 4:**
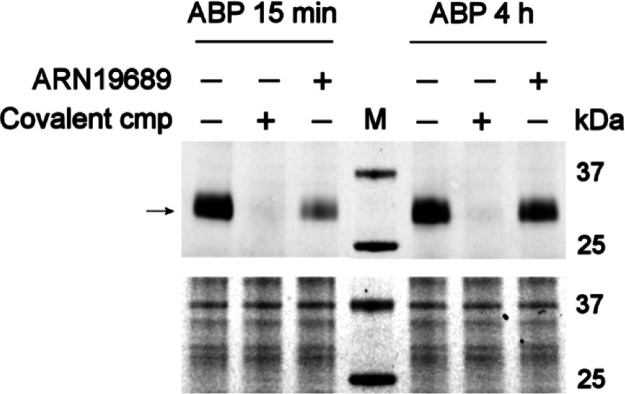
Sulfonamide **50** (**ARN19689**) binds to *human* NAAA in a non-covalent manner. Lysosomal protein extracts from *h*-NAAA overexpressing HEK-293 cells were incubated with **50** or a covalent reference compound *ARN15393*.^46^ Control samples (−/−) were incubated with DMSO alone. Activity-based probe (ABP) specific to NAAA, *ARN14686*,^45^ was next added for 15 min or 4 h and a fluorophore was inserted by click chemistry. The fluorescent band corresponding to ABP-bound NAAA is indicated by the arrow. Signal disappearing with respect to controls indicated that tested compounds were bound to *h*-NAAA. Increased band intensity in compound **50**-incubated samples after 4 h indicated that compound has detached from *h*-NAAA. Top, in gel fluorescence analysis; bottom, coomassie blue staining (loading control). M: molecular weight marker [for the structure of β-lactam covalent NAAA inhibitors *ARN15393* and *ARN14686*, see Figure S1, Supporting Information].

According to these promising biological data, sulfonamide **50** was further characterized for its in vitro profile. Interestingly, in biological assays, the compound showed a very high selectivity toward both human FAAH and human acid ceramidase (AC)^22^ (25 and 34% inhibition at 30 μM, respectively). Along with its NAAA inhibitory activity and selectivity toward FAAH and AC, an evaluation of the mode of action of compound **50** was also investigated. A competitive activity-based protein profiling (ABPP)^44^ biochemical analysis revealed also for this new azabicyclic compound, featuring no reactive chemical moieties toward the catalytic cysteine, a non-covalent interaction with *h*-NAAA. Compound **50** prevented the binding of the activity-based probe (ABP) *ARN14686*(45) (Figure S1) to *h*-NAAA in cell lysosomal extracts in a 15 min incubation experiment. However, its binding was almost totally reverted after 4 h, demonstrating a relatively transient interaction with the enzyme. Conversely, as expected, a known β-lactam covalent NAAA inhibitor (*ARN15393*)^46^ (Figure S1) stably antagonized *h*-NAAA labeling by the ABP at both short (15 min) and long (4 h) incubation times (Figure 4). In addition to demonstrating its reversibility as a NAAA inhibitor, this experiment allows speculations on the putative site of interaction of compound **50** with NAAA. Because the ABP *ARN14686* has been reported to bind the catalytic cysteine of *h*-NAAA (Cys-126),^45^ the prevention of its interaction with NAAA by preincubation with **50** can be explained by the compound’s binding to the same pocket or to an allosteric site that, upon engagement, induces a conformational change of the enzyme structure that precludes the interaction with the covalent inhibitor.

**Figure 5:**
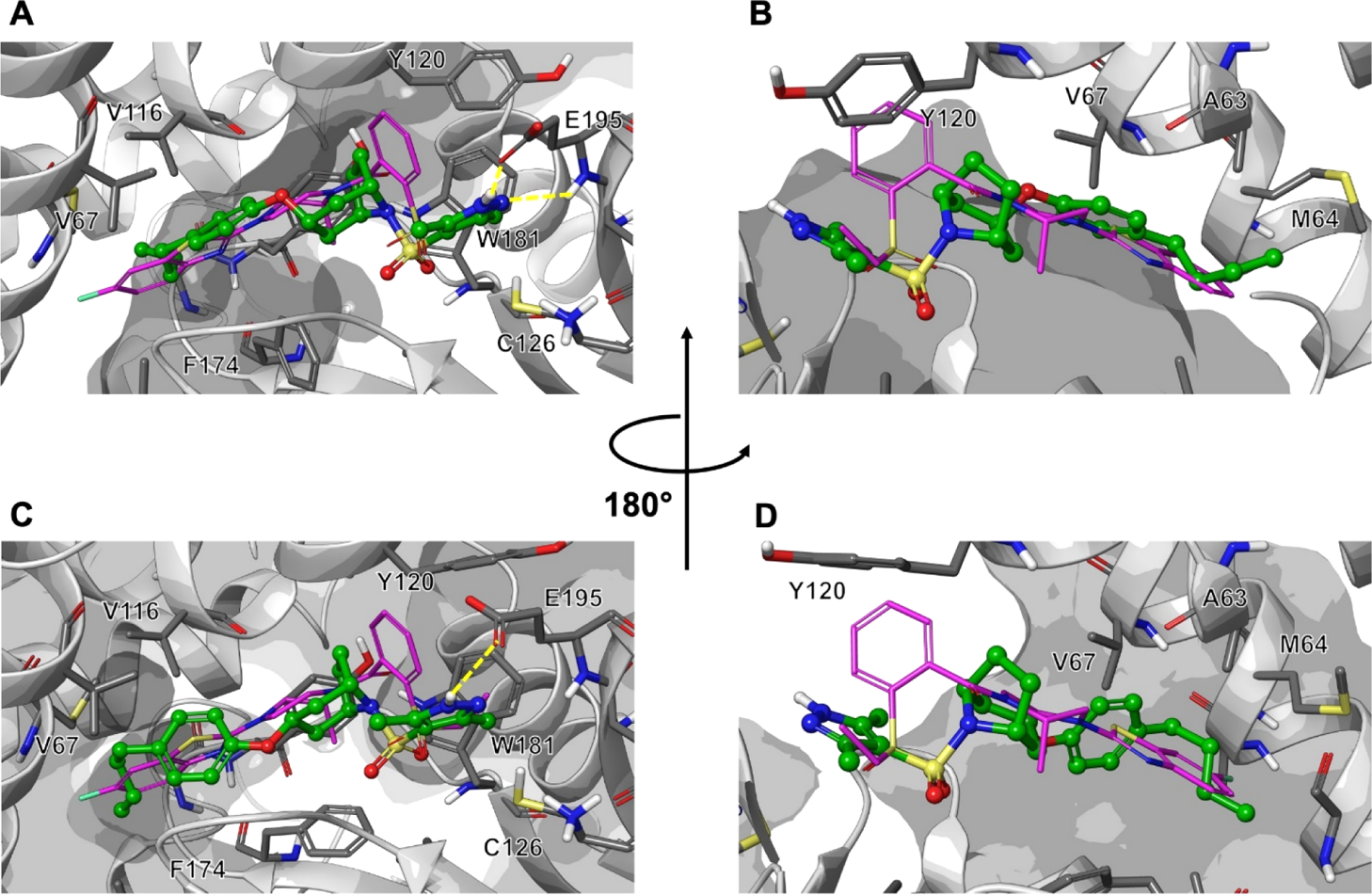
Predicted bound conformations of compounds **39** and **40** at the binding site of NAAA. The protein structure is reported in white ribbon (PDB ID: 6DXX). Residues interacting with the inhibitors are reported in stick representation with gray carbons and labeled explicitly. The cocrystallized conformation of *ARN19702*(11) (Figure 1) is reported in magenta for reference. (A) Bound conformation of **39** in ball and stick representation (green carbons). (B) Rear view of the bound conformation of **39** in ball and stick representation (green carbons). (C) Bound conformation of **40** in ball and stick representation (green carbons). (D) Rear view of the bound conformation of **40** in ball and stick representation (green carbons).

To support these outcomes, flexible ligand-docking studies in the NAAA active site were also performed on lead compound **39** and optimized analogue **50**. *Endo*-substituted azabicyclooctane **39** was shown to bind at the binding pocket of NAAA with an orientation similar to that of the cocrystallized non-covalent inhibitor *ARN19702* (Figure 5A).^11,31^ In particular, the common sulfonamide group was predicted to occupy approximately the same region as in the cocrystal, although with a different orientation. The substituted pyrazole ring established a double H-bond interaction with the side chain and backbone of E195. The *endo*-isomer of the substituted azabicyclic core positioned the phenyl ring in the same region occupied by the 1,3-thiazole ring of the benzothiazole group in *ARN19702*. In this way, the aliphatic *n*-butyl chain could be lodged in a deeply hydrophobic region of the binding site, slightly displacing the side chain of M64 (Figure 5B). Additionally, desolvation penalty also contributes positively to the higher binding affinity observed for the more lipophilic moieties.

The proposed binding mode explained why preventing one of the two *H*-bond interactions (i.e., introducing a substituent on the *N*-1 nitrogen of the pyrazole or replacing the pyrazole itself with an isoxazole ring) is detrimental for activity. Second, it accounts for the marked decrease in potency generally displayed by the *exo*-diastereoisomers. In the *exo*-series, the interaction with E195 is partially compromised (Figure 5C). Furthermore, pointing toward the backbone of A63, the vector for the aliphatic substituent in the phenyl ring displays a less-favorable orientation with respect to the *endo*-series (Figure 5D). Given the tightness of the pocket, the ca. 450-fold potency gap between the paired isomers **39** and **40** could be reasonably justified. While docking score does not usually correlate with activity within congeneric series, advanced docking protocols, that take receptor flexibility into account, have been reported to be able to correctly characterize activity cliffs, that is, significant changes in activity caused by local structural differences.^47^ Also in our case, the use of advanced docking protocols provided a possible explanation for the significant difference in NAAA inhibitory potency between diastereoisomers **39** and **40**.

The newly identified optimized *endo*-substituted azabicyclic sulfonamide **50** was also investigated in docking studies in the NAAA active site, displaying a binding mode almost perfectly overlapping with the one predicted for lead compound **39** (see Figure S2, Supporting Information).

Altogether, these data could indicate that the novel compound **50** may interact with NAAA by forming tight/non-covalent contacts via H-bond and hydrophobic interactions, as seen for lead compound **39** (Figure 5).

Although these studies support the binding of compound **50** to the NAAA active site, we cannot rule out that the compound inhibits the enzyme by binding to an allosteric site, thus inducing a conformational change of the enzyme structure that prevents the interaction with the substrate.

The excellent in vitro inhibitory activity, selectivity, and biochemical data of optimized compound **50** prompted us also to assess its physicochemical properties and in vivo pharmacokinetic profile, compared to its early analogue **39** (Table 5). While both compounds showed a high plasma (mouse and rat *t*_1/2_ > 120 min) and liver microsomal (mouse and human *t*_1/2_ > 60 min) stability, the structural modifications leading to compound **50** contributed to improve not only resistance to oxidative transformations in rat microsomes (*t*_1/2_ = 48 min) but also, more importantly, lipophilicity (*c* Log *P* = 0.54) and kinetic solubility in buffer (>250 μM in PBS at pH 7.4). Interestingly, the more polar character of NAAA inhibitor **50**, together with its favorable biological activity, resulted in an overall optimal lipophilic ligand efficiency value (LipE = 6.83), which is expected to positively impact on the developability of the compound as a possible preclinical candidate (Table 5).

**Table 5:** Plasma and Liver Microsomal Stability, Solubility, Efficiency Metrics, and Mouse Pharmacokinetic Data of Compounds **39** and **50**

					mouse PK^g^		
compound	plasma *t*_1/2_(min)^a^^,^^c^	LM_NADPH *t*_1/2_(min)^b^^,^^c^	solubility (μM)^d^	c Log P^e^/LipE^f^	parameters	i.v.(3 mg/kg)	p.o.(10 mg/kg)
**39**	*m*: >120 (93)^h^	*m*: >60 (93)^h^	<1	3.79/3.84	*C*_max_[ng/mL]	701.9	296.6
	*r*: >120 (93)^h^	*r*: 13 ± 4			*T*_max_ (min)	5	30
		*h*: >60 (83)^h^			AUC [ng × min/mL]	20800	20748
					*V*_d_(L/Kg)	4.45	
					CL (mL/min/Kg)	144	
					F (%)	30	
**50**	*m*: >120 (95)^h^	*m*: >60 (55)^h^	>250	0.54/6.83	*C*_max_[ng/mL]	5238	6872
	*r*: >120 (93)^h^	*r*: 48 ± 2			*T*_max_ (min)	5	15
		*h*: >60 (88)^h^			AUC [ng × min/mL]	202304	407508
					*V*_d_(L/Kg)	0.53	
					CL (mL/min/Kg)	13	
					F (%)	60	

a 2.0 μM, 100% *mouse* or *rat* plasma (+0.5% DMSO).

b 4.6 μM in *mouse, rat* or *human* liver microsomes (LM) with NADPH as a cofactor (0.1% DMSO).

c Data collected as *n* ≥ 3.

d Kinetic solubility (PBS, pH 7.4; *n* = 3).

e Calculated with PipelinePilot WebPort 2017.

f LipE: Lipophilic Efficiency [LipE = pIC_50_ – *c* Log *P*].^42^

g Pharmacokinetic parameters of **39** and **50** following intravenous (i.v.) and oral (p.o.) administration to male C57BL/6 mice (*n* = 3 per time-point).

h % compound remaining at last time-point.

Based on both its biological characterization, showing a good efficacy and biochemical profile, and preliminary in vitro ADME properties, the novel NAAA inhibitor **50** was further evaluated in vivo. The compound was administered to male C57BL/6 mice by intravenous (i.v.) infusion, at a dose of 3 mg/kg, and by oral gavage (p.o.), at a dose of 10 mg/kg, to determine its pharmacokinetic parameters (Table 5 and Figure S3). Compound **50** showed much higher plasma concentrations (*C*_max_ and AUC) following i.v. and p.o. administration, a lower volume of distribution (*V*_D_ = 0.53 L/kg) and clearance (CL = 13 mL/min/kg), and a 2-fold increase in overall oral bioavailability (*F* = *ca.* 60%) with respect to its earlier analogue **39** (Table 5).

Thanks to its encouraging in vitro and in vivo pharmacological data, the newly identified non-covalent azabicyclooctane sulfonamide **50** represents a valuable tool to be further investigated in animal models of inflammatory conditions.

## Chemistry

**Scheme 1:**
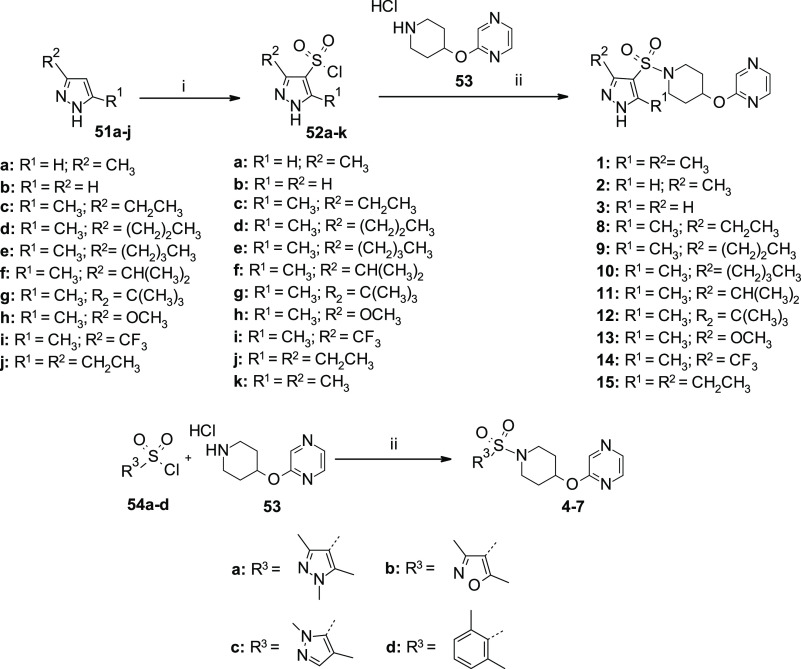
Synthesis of Substituted Sulfonamides **1–15** Reagents and reaction conditions: (i) HSO_3_Cl, 100 °C, 3 h; (ii) TEA, dry THF, r.t., overnight, 16–91%.

**Scheme 2:**
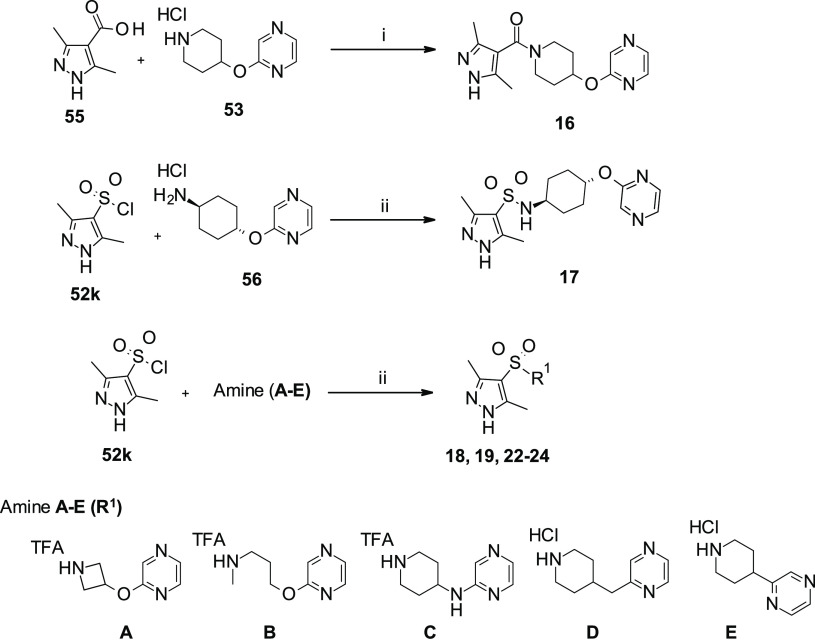
Synthesis of Substituted Pyrazol-Amide **16** and Sulfonamides **17–19**, **22–24** Reagents and reaction conditions: (i) TEA, HBTU, dry DCM/DMF (3/1), r.t., 30 min, then **53**, r.t., 48 h, 41%; (ii) TEA, dry THF, r.t., overnight, 34–87%. [TFA: trifluoroacetic acid].

The synthesis of desired compounds **1–50** was conveniently accomplished by a general coupling reaction between an appropriate sulfonyl chloride or carboxylic acid with a suited amine. In detail, sulfonamide analogues **1–15** (Scheme 1), **17–19**, and **22–24** (Scheme 2) were obtained (16–91% yield) in a one-step reaction by using sulfonyl chlorides **52a–k**, **54a–d** (Scheme 1), and opportunely prepared amines **53**, **56**, and **A–E** (Scheme 2) in the presence of triethylamine in THF. The amide derivative **16** (Scheme 2) was prepared (41% yield) by reaction between commercially available carboxylic acid **55** and amine **53** in the presence of triethylamine and HBTU in a mixture of DCM/DMF.

Sulfonyl chlorides **52a–j** were easily prepared by reaction of the corresponding substituted pyrazoles **51a–j** with chlorosulfonic acid (Scheme 1).^48^

**Scheme 3:**
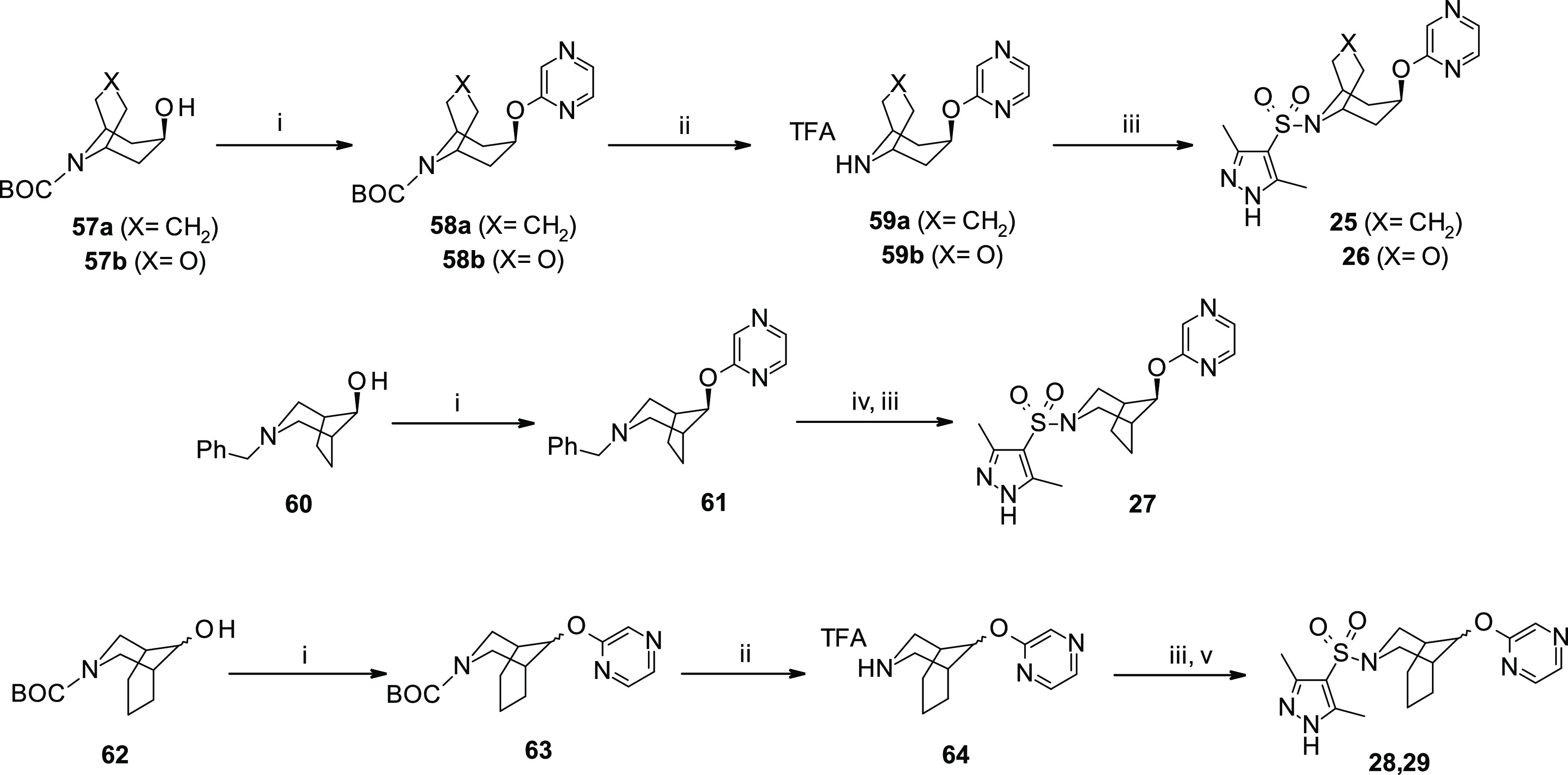
Synthesis of Substituted Pyrazol Azabicyclic-Sulfonamides **25–29** Reagents and reaction conditions: (i) 2-chloropyrazine (**73a**), *t*-BuOK, dry THF, reflux, overnight, 34–78%; (ii) TFA/DCM, 0 °C to r.t., 2 h; (iii) 3,5-dimethyl-1*H*-pyrazole-4-sulfonyl chloride (**52k**), TEA, dry THF or DCM, r.t., overnight, 7–48%; (iv) HCOONH_4_, EtOH, Pd/C (10%), r.t., 3 h; and (v) preparative HPLC purification. [TFA: trifluoroacetic acid; BOC: *tert*-butoxycarbonyl].

Bicyclic sulfonamides **25–26**, **28–29** were synthesized (7–13% yield) as reported in Scheme 3. Bicyclic *endo*- or *exo*-alcohols **57a**,^49^**b**^50^ and **62**(51) were used in an *O*-substitution reaction with 2-chloropyrazine (**73a**) to afford pyrazinyloxy intermediates **58a**,**b** and **63**, which were then subjected to Boc-deprotection leading to the secondary amines **59a**,**b** and **64** in moderate to good yields (34–78%). Although the desired sulfonamides **25**,**26** were obtained from the corresponding amines **59a**,**b** by treatment with 3,5-dimethyl-1*H*-pyrazole-4-sulfonyl chloride (**52k**), the use of pyrazine-ether **64**, as a mixture of two diastereoisomers, led to a mixture of *endo/exo*-sulfonamides **28**,**29**, which were isolated as pure stereoisomers after HPLC purification (Scheme 3).

Sulfonamide **27** was obtained in a straightforward manner starting from commercially available benzyl-azabicyclooctanol **60**, which was initially used in a substitution reaction with 2-chloropyrazine (**73a**) to give intermediate **61**. Next, the debenzylation with ammonium formate in the presence of Pd/C (10%), followed by coupling with sulfonyl chloride **52k** gave the desired compound in a 30% overall yield (Scheme 3).

**Scheme 4:**
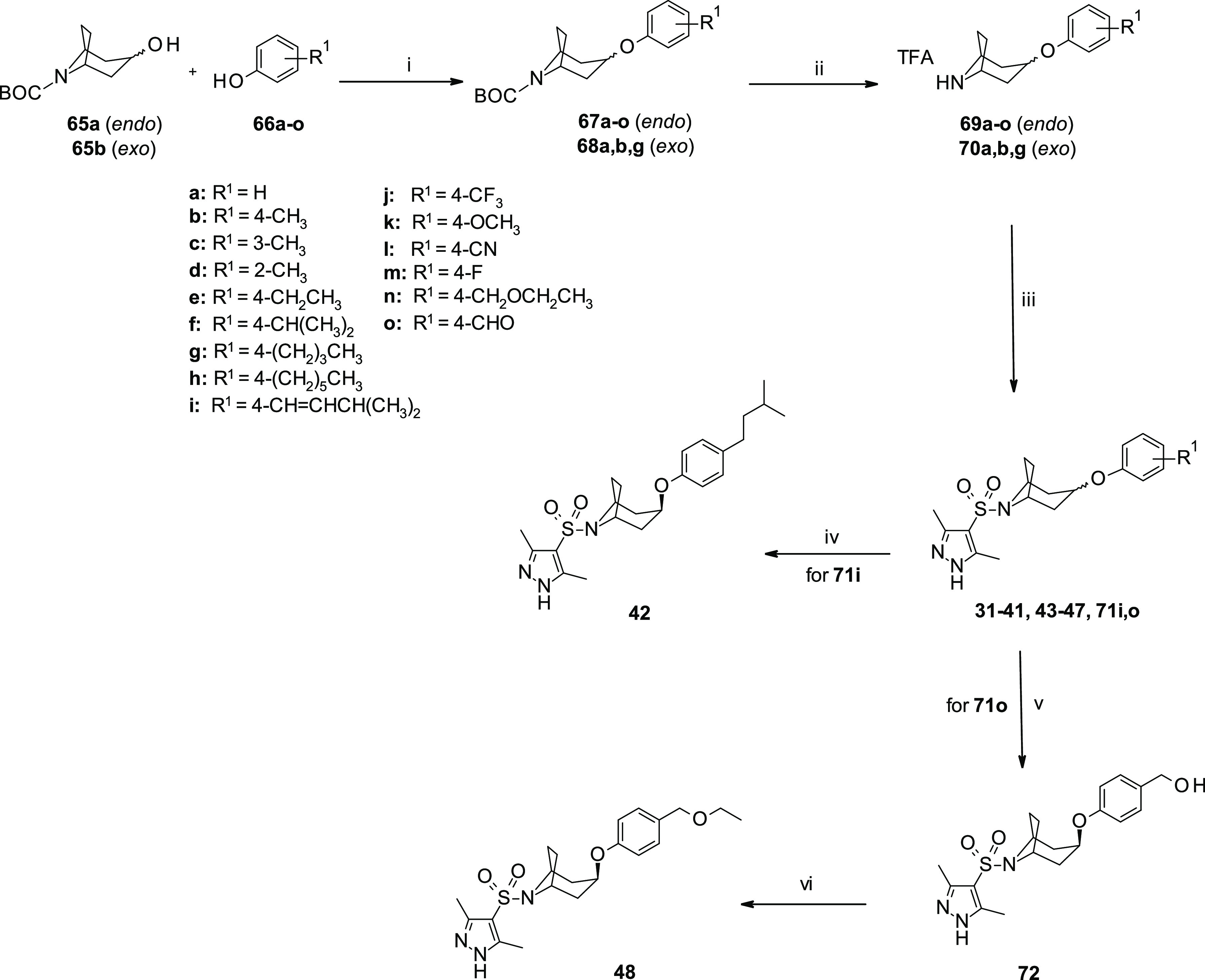
Synthesis of *Endo*- and *Exo*-Substituted Phenoxy Azabicyclic-Sulfonamides **31–48** Reagents and reaction conditions: (i) PPh_3_, DIAD, dry THF, 0 °C to r.t., overnight, 29–83%; (ii) TFA/DCM, 0 °C to r.t., 2 h; (iii) 3,5-dimethyl-1H-pyrazole-4-sulfonyl chloride (**52k**), TEA, dry THF, r.t., overnight, 10–91% (iv) HCOONH_4_, EtOH, Pd/C (10%), r.t., 30 min, 66%; (v) NaBH_4_, MeOH, 0 °C to r.t., 1 h; and (vi) Amberlist-15, EtOH, reflux, 16 h, 55%. [TFA: trifluoroacetic acid; BOC: *tert*-butoxycarbonyl].

The synthesis of diastereomeric *endo*- or *exo*-tropyl-sulfonamides **31–48** was performed as shown in Scheme 4. *N*-Boc-protected *endo*-(**65a**) or *exo*-tropanol (**65b**) were allowed to react with different substituted phenols **66a–o** via Mitsunobu conditions leading, after the *endo*-*/exo*-SN_2_ mechanism, to the corresponding *endo*- (**67a–o**) and *exo*- (**68a**–**b**,**g**) arylether derivatives in moderate to high yields (29–83%). Subsequent *N*-Boc-deprotection of these latter compounds gave the corresponding trifluoroacetate salts **69a–o** and **70a**–**b**,**g**, which were coupled with the desired sulfonyl chloride **52k** to obtain the final azabicyclooctane sulfonamides **31–41, 43–47** (10–91%), and intermediates **71i**,**o** (Scheme 4). Upon reduction of the side chain on the phenyl ring, compound **71i** furnished the final analogue **42** in good yield (66%). Azabicyclic sulfonamide **48** was prepared in 55% yield, following a two-step sequence starting from intermediate **71o**, which was initially converted into the corresponding primary alcohol **72** and then *O*-alkylated by using Amberlist-15.

**Scheme 5:**
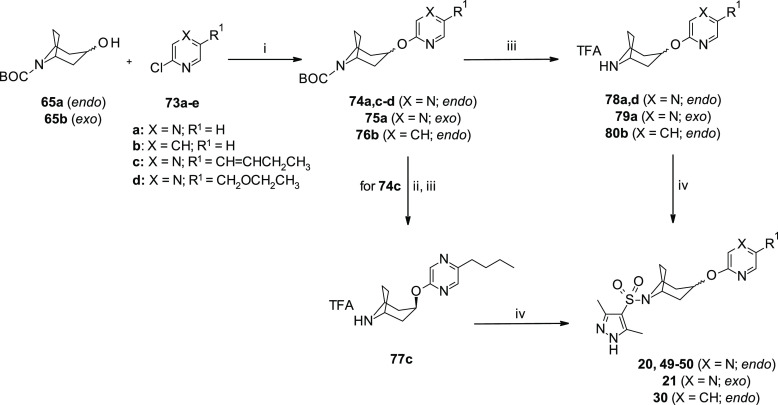
Synthesis of *Endo*- and *Exo*-Substituted Oxo-Pyrazine (**20–21**, **49–50**) and Oxo-Pyridine (**30**) Azabicyclooctane Sulfonamides Reagents and reaction conditions: (i) 2-Chloropyrazines (**73a,c–d**) or 2-chloropyridine (**73b**), *t*-BuOK, dry THF, reflux, overnight, 15–37%; (ii) cyclohexene, EtOH, Pd/C (10%), reflux, 2 h; (iii) TFA/DCM, 0 °C to r.t., 2 h; and (iv) 3,5-dimethyl-1*H*-pyrazole-4-sulfonyl chloride (**52k**), TEA, dry THF, r.t., overnight, 31–83%. [TFA: trifluoroacetic acid; BOC: *tert*-butoxycarbonyl].

Finally bicyclic sulfonamides **20**, **21**, **30**, and **50** were synthesized (3–26% yield) from the corresponding bicyclic *endo*-**65a** and *exo*-**65b** alcohols following a similar reaction sequence to that used for analogues **25**, **26** (Scheme 5). Sulfonamide **49** was obtained by a coupling reaction of sulfonyl chloride **52k** with trifluoroacetate salt **77c**, which was previously isolated after double bond reduction/Boc-deprotection of pyrazino intermediate **74c** (Scheme 5).

## Conclusions

The endogenous lipid mediator, PEA, has been extensively reported to have anti-inflammatory and analgesic properties.^52^ Among the potential ways to manage the inflammatory response, sustaining PEA levels by inhibiting its deactivating hydrolysis represents a promising therapeutic approach.^53^ Cellular breakdown of PEA is mainly mediated by the cysteine hydrolase NAAA, whose inhibition can prevent endogenous PEA hydrolysis, thereby eliciting an anti-inflammatory response.

In the present work, we report the identification of a potent, systemically available, novel class of NAAA inhibitors, endowed with a non-covalent mechanism of action. Starting from sulfonamide hit **1**, identified in an MTS campaign, an in-depth SAR exploration led us to the discovery of highly potent *h*-NAAA inhibitors, featuring an azabicyclo[3.2.1]octane-pyrazole sulfonamide, as a novel chemotype. Among them, optimized compound **50** (**ARN19689**) was found to inhibit *h*-NAAA with a median inhibitory concentration in the low nanomolar range, showing more than 25-fold activity improvement compared to the starting hit (**1** vs **50**, IC_50_ = 1.09 and 0.042 μM, respectively). The evolution of hit **1** led to the azabicyclo[3.2.1]octane-pyrazole lead compound **39**, a potent *h*-NAAA inhibitor with suboptimal drug-like properties. Structural modifications of the latter compound were undertaken to improve the physicochemical and drug-like properties, while retaining a good inhibitory activity against *h*-NAAA. This work ultimately allowed identifying *endo*-ethoxymethyl-pyrazinyloxy-8-azabicyclo[3.2.1]octane-pyrazole sulfonamide **50** (**ARN19689**), a compound with a good lipophilic efficiency (LipE = 6.83) and a superior pharmacological and pharmacokinetic properties.

The overall excellent profile of sulfonamide **50**, in terms of NAAA inhibitory activity and biochemical/drug-like properties, makes this compound an interesting candidate to be tested in animal models of inflammatory diseases.

## Experimental Section

### Chemistry

#### Synthetic Materials and Methods

All manipulations of air- or moisture-sensitive materials were carried out in oven- or flame-dried glassware under an inert atmosphere of nitrogen or argon. Syringes, which were used to transfer reagents and solvents, were purged with nitrogen prior to use. Reaction solvents were obtained anhydrous from commercial suppliers. All reagents were obtained from commercial suppliers and used without further purification unless indicated otherwise. Automated column chromatography purifications were performed on a Teledyne ISCO apparatus (CombiFlash Rf) with prepacked silica gel columns of different sizes (Redisep). NMR experiments were run at 300 K on a Bruker Avance III 400 system (400.13 MHz for ^1^H and 100.62 MHz for ^13^C), equipped with a BBI probe and Z-gradients, and a Bruker FT NMR Avance III 600 MHz spectrometer equipped with a 5 mm CryoProbeTM QCI ^1^H/^19^F–^13^C/^15^N–D quadruple resonance, a shielded z-gradient coil, and the automatic sample changer SampleJet NMR system (600 MHz for ^1^H, 151 MHz for ^13^C and 565 MHz for ^19^F). Chemical shifts for the ^1^H and ^13^C spectra were reported in parts per million (ppm), calibrating the residual non-deuterated solvent peak for the ^1^H and ^13^C, respectively, to 7.26 and 77.16 ppm for CDCl_3_ and 2.50 and 39.52 ppm for DMSO-*d*_6_. UPLC-MS analyses were performed on a Waters ACQUITY UPLC-MS system consisting of a single quadrupole detector (SQD) mass spectrometer equipped with an electrospray ionization interface and a photodiode array detector (PDA) from Waters Inc. (Milford, MA, USA). Electrospray ionization in positive and negative mode was applied in the mass scan range 100–500 Da. The PDA range was 210–400 nm. The mobile phase was 10 mM NH_4_OAc in H_2_O at pH 5 adjusted with AcOH (A) and 10 mM NH_4_OAc in CH_3_CN–H_2_O (95:5) at pH 5 (B) with 0.5 mL/min as a flow rate. For intermediates, the analyses were run on an ACQUITY UPLC BEH C_18_ column (50 × 2.1 mm ID, particle size 1.7 μm) with a VanGuard BEH C_18_ precolumn (5 × 2.1 mm ID, particle size 1.7 μm). A linear gradient was applied: 0–0.2 min: 5% B; 0.2–2.2 min: 5–95% B; 2.2–2.3 min: 95–100% B; and 2.3–3.0 min: 100% B. For final compounds **1-50**, a 10 mM DMSO stock solution of the test compound was prepared in DMSO-*d*_6_ and further diluted 20-fold in CH_3_CN–H_2_O (1:1) for analysis. The analyses were run on an ACQUITY UPLC BEH C_18_ column (100 × 2.1 mm ID, particle size 1.7 μm) with a VanGuard BEH C_18_ precolumn (5 × 2.1 mm ID, particle size 1.7 μm). *Generic method:* a linear gradient was applied starting at 0–0.2 min: 10% B; 0.2–6.2 min: 10–90% B; 6.2–6.3 min: 90–100% B; and 6.3–7.0 min: 100% B; *Apolar method:* a linear gradient was applied starting at 0–0.2 min: 50% B; 0.2–6.2 min: 50–100% B; and 6.2–7.0 min: 100% B. The purifications by HPLC-MS were performed on a Waters Autopurification system consisting of a 3100 single quadrupole mass spectrometer equipped with an electrospray ionization interface and a 2998 photodiode array detector. The HPLC system included a 2747 sample manager, a 2545 binary gradient module, a system fluidic organizer, and a 515 HPLC pump from Waters Inc. (Milford, MA, USA). Electrospray ionization in positive and negative mode was used in the mass scan range 100–500 Da. The PDA range was 210–400 nm. The purifications were run on a XBridge Prep C_18_ OBD column (100 × 19 mm ID, particle size 5 μm) with a XBridge Prep C_18_ (10 × 19 mm ID, particle size 5 μm) guard cartridge with a flow rate = 20 mL/min. High-resolution mass spectrometry (HRMS) measurements were performed on a Waters Synapt G2 Q-ToF mass spectrometer equipped with an electrospray ionization interface and coupled to a Waters ACQUITY UPLC from Waters Inc. (Milford, MA, USA). Leucine enkephalin (2 ng/mL) was used as lock mass reference compound for spectral recalibration. The analyses were run on an ACQUITY UPLC BEH C_18_ column (100 × 2.1 mm ID, particle size 1.7 μm) with a VanGuard BEH C_18_ precolumn (5 × 2.1 mm ID, particle size 1.7 μm). The mobile phase was H_2_O + 0.1% HCOOH (A) and CH_3_CN + 0.1% HCOOH (B) with 0.5 mL/min as a flow rate. A linear gradient was applied: 0–0.2 min: 10% B; 0.2–6.2 min: 10–90% B; 6.2–6.3 min: 90–100% B; and 6.3–7.0 min: 100% B. The synthesis and characterization of all final compounds **1-50** is reported below. Purity of the final compounds was determined by UPLC-MS and quantitative ^1^H NMR (qNMR, see the Supporting Information) and was equal or greater than 95% for all of the compounds, except for analogue **3** (91% purity).

#### General Procedure (GP) for the Synthesis of Amines **53**, **56**, **B**

##### Step 1

To a suspension of sodium hydride (60% dispersion in mineral oil, 1.4 equiv) in DMF (10 mL/equiv) at 0 °C, commercially available *tert*-butyl 4-hydroxypiperidine-1-carboxylate (1.8 equiv) or *tert*-butyl (4-hydroxycyclohexyl)carbamate (1.2 equiv) or *tert*-butyl (3-hydroxypropyl)-*N*-methyl-carbamate (1.2 equiv) in DMF (10 mL/equiv) was added. The resulting mixture was stirred for 30 min at room temperature, then 2-chloropyrazine (**73a**) (1.0 equiv) was added and the mixture stirred for further 90 min at room temperature. Water (20 mL/equiv) was added and the crude product extracted with EtOAc (3 × 20 mL). The combined organic phases were dried over Na_2_SO_4_, filtered, and evaporated under vacuo.

##### Step 2a

*tert*-Butyl 4-(pyrazin-2-yloxy)piperidine-1-carboxylate or *tert*-butyl (4-(pyrazin-2-yloxy)cyclohexyl)carbamate (1.0 equiv) was dissolved in EtOH (16 mL/equiv) and HCl conc. (8 mL/equiv) was added at 0 °C. The reaction was stirred at room temperature overnight. The organic layer was concentrated under vacuo, and the obtained residue was recrystallized from Et_2_O to give the desired compound as a white solid in quantitative yield.

##### Step 2b

*tert*-Butyl methyl-(3-pyrazin-2-yloxy)propyl-carbamate (0.1 g, 0.38 mmol, 1.0 equiv) was dissolved in 3:1 DCM/TFA (4.0 mL/equiv) at 0 °C. The reaction was stirred at room temperature overnight. The organic layer was concentrated under vacuo, and the obtained residue was used in the next step without any further purification.

##### *tert*-Butyl 4-(pyrazin-2-yloxy)piperidine-1-carboxylate

Following GP-step 1, 2-chloropyrazine (**73a**) (1.15 g, 10.04 mmol) was used to produce a crude product, which was subjected to flash chromatography eluting with cyclohexane/*tert*-butyl methyl ether (6:4) to give the pure title compound (2.1 g, 75%) as a white solid. UPLC-MS: *t*_R_ = 2.45 min (generic method); MS (ESI) *m*/*z*: calcd. for C_14_H_22_N_3_O_3_ [M + H]^+^, 280.2; found, 280.1. ^1^H NMR (400 MHz, DMSO-*d*_6_): δ 8.28 (d, *J* = 1.0 Hz, 1H), 8.21–8.20 (m, 2H), 5.21–5.15 (m, 1H), 3.73–3.67 (m, 2H), 3.19 (t, *J* = 11.3 Hz, 2H), 2.00–1.93 (m, 2H), 1.63–1.57 (m, 2H), 1.41 (s, 9H).

##### 2-(Piperidin-4-yloxy)pyrazine Hydrochloride (**53**)

Following GP-Step 2a, the pure title compound was obtained (1.55 g, 97%) as a white solid. UPLC-MS: *t*_R_ = 0.80 min (generic method); MS (ESI) *m*/*z*: calcd. for C_9_H_14_N_3_O [M + H]^+^, 180.1; found, 180.1. ^1^H NMR (400 MHz, CD_3_OD): δ 7.48 (d, *J* = 1.5 Hz, 1H), 7.44–7.43 (m, 1H), 7.38 (d, *J* = 2.9 Hz, 1H), 4.62–4.57 (m, 1H), 2.65–2.59 (m, 2H), 2.48–2.43 (m, 2H), 1.49–1.41 (m, 2H), 1.35–1.27 (m, 2H).

##### *tert*-Butyl (4-(pyrazin-2-yloxy)cyclohexyl)carbamate

Following GP-Step 1, 2-chloropyrazine (**73a**) (0.2 g, 1.75 mmol) was used to produce a crude product, which was subjected to flash chromatography eluting with DCM/MeOH (98:2) to give the pure title compound (0.224 g, 44%) as a white solid. UPLC-MS: *t*_R_ = 2.41 min (generic method); MS (ESI) *m*/*z*: calcd. for C_15_H_24_N_3_O_3_ [M + H]^+^, 294.2; found, 294.1. ^1^H NMR (400 MHz, DMSO-*d*_6_): δ 8.24 (d, *J* = 1.4 Hz, 1H), 8.19 (dd, *J* = 2.8, 1.4 Hz, 1H), 8.17 (d, *J* = 2.8 Hz, 1H), 6.77 (d, *J* = 7.9 Hz, 1H), 4.92–4.85 (m, 1H), 2.09–2.05 (m, 2H), 1.84 (d, *J* = 12.6 Hz, 2H), 1.54–1.43 (m, 2H), 1.39 (s, 9H), 1.36–1.23 (m, 3H).

##### (1*r*,4*r*)-4-(Pyrazin-2-yloxy)cyclohexan-1-amine Hydrochloride (**56**)

Following GP-Step 2a, the pure title compound was obtained (1.65 g, 97%) as a white solid. UPLC-MS: *t*_R_ = 0.98 min (generic method); MS (ESI) *m*/*z*: calcd. for C_10_H_16_N_3_O [M + H]^+^, 194.1; found, 194.1 ^1^H NMR (400 MHz, DMSO-*d*_6_): δ 8.26 (d, *J* = 1.2 Hz, 1H), 8.21–8.19 (m, 2H), 4.95–4.88 (m, 1H), 3.09 (br s, 1H), 2.14–2.12 (m, 2H), 2.05–2.02 (m, 2H), 1.58–1.47 (m, 4H).

##### *tert*-Butyl methyl(3-pyrazin-2-yloxy)propyl)carbamate

Following GP-Step 1, 2-chloropyrazine (**73a**) (0.2 g, 1.75 mmol) was used to produce a crude product, which was subjected to flash chromatography eluting with cyclohexane/ethyl acetate (5:5) to give the pure title compound (0.1 g, 21%) as a white solid. UPLC-MS: *t*_R_ = 2.28 min (generic method); MS (ESI) *m*/*z*: calcd. for C_13_H_22_N_3_O_3_ [M + H]^+^, 268.2; found, 268.2. ^1^H NMR (400 MHz, DMSO-*d*_6_): δ 8.29 (d, *J* = 1.1 Hz, 1H), 8.21–8.19 (m, 2H), 4.28 (t, *J* = 6.2 Hz, 2H), 2.79 (br s, 3H), 2.00–1.91 (m, 2H), 1.39–1.31 (m, 11H).

##### *N*-methyl-(3-pyrazin-2-yloxy)propan-1-amine trifluoroacetate (**B**)

Following GP-Step 2b, the obtained salt was used in the next step without any further purification.

##### 2-(Azetidin-3-yloxy)pyrazine trifluoroacetate (**A**),^54^*N*-(piperidin-4-yl)pyrazin-2-amine trifluoroacetate (**C**),^55^ and 2-(piperidin-4-yl)pyrazine hydrochloride (**E**)^56^

These were synthesized as previously reported.

##### tert-Butyl 4-(pyrazin-2-ylmethyl)piperidine-1-carboxylate

*tert*-Butyl 4-methylenepiperidine-1-carboxylate (0.1 g, 0.51 mmol, 1.0 equiv) and 9-BBN (0.5 M in THF, 1.02 mL, 0.51 mmol, 1.0 equiv) were heated at 65 °C for 1 h. Then, freshly prepared boronate was added dropwise to a mixture of 2-chloropyrazine (**73a**) (0.058 g, 0.51 mmol, 1.0 equiv), Pd(dppf)Cl_2_ (0.037 g, 0.051 mmol, 0.1 equiv), and K_2_CO_3_ (0.08 g, 0.58 mmol, 1.14 equiv) in a mixture of DMF/H_2_O (0.5/0.1 mL). The reaction mixture was heated at 60 °C for 2 h, then a sat. aq. NaHCO_3_ solution (10 mL) was added, and the mixture was extracted with EtOAc (3 × 10 mL). The combined organic phases were dried over Na_2_SO_4_, filtered, and evaporated under vacuo. The obtained residue was purified by flash chromatography eluting with cyclohexane/ethyl acetate (1:1) to give the pure title compound (0.088 g, 62%) as a white solid. UPLC-MS: *t*_R_ = 2.22 min (generic method); MS (ESI) *m*/*z*: calcd. for C_15_H_24_N_3_O_2_ [M + H]^+^, 278.2; found, 278.0. ^1^H NMR (400 MHz, DMSO-*d*_6_): δ 8.57–8.56 (m, 1H), 8.54 (d, *J* = 1.5 Hz, 1H), 8.47 (d, *J* = 2.5 Hz, 1H), 3.90 (d, *J* = 13.1 Hz, 2H), 2.72–2.67 (m, 4H), 1.98–1.87 (m, 1H), 1.54–1.51 (m, 2H), 1.39 (s, 9H), 1.14–1.04 (m, 2H).

##### 2-(Piperidin-4-ylmethyl)pyrazine Hydrochloride (**D**)

*tert*-Butyl 4-(pyrazin-2-ylmethyl)piperidine-1-carboxylate (0.05 g, 0.18 mmol, 1.0 equiv) was dissolved in 1,4-dioxane (1.8 mL), and HCl (0.45 mL, 4 M in dioxane) was added at 0 °C. The reaction was stirred at room temperature overnight. The organic layer was concentrated under vacuo, and the obtained crude was used in the next step without any further purification.

#### General Procedure for the Synthesis of Pyrazoles **51a**,**b**,**d**–**g**,**i**–**j**

To a solution of diketone (1.0 equiv) in EtOH (2.0 mL/equiv), hydrazine hydrate (1.2 equiv) at 0 °C was added. The reaction mixture was warmed up to room temperature and stirred for 1 h. The solution was concentrated under vacuo and extracted with EtOAc (3 × 10 mL). The organic phases were dried over Na_2_SO_4_, filtered, and evaporated under reduced pressure to obtain the desired pyrazoles, which were used in the next step without any further purification.

5-Ethyl-3-methyl-1*H*-pyrazole (**51c**)^57^ and 5-methoxy-3-methyl-1*H*-pyrazole (**51h**)^58^ were synthesized as previously reported.

#### General Procedure for the Synthesis of Pyrazole-4-Sulfonyl Chlorides **52a–j**

Chlorosulfonic acid (5.0 equiv) was added to the appropriate pyrazole (1.0 equiv) at 0 °C and the reaction mixture was heated at 100 °C for 3 h. The solution was cooled at room temperature and added to a stirrer solution of ice and DCM (15 mL). The organic phase was separated, dried over Na_2_SO_4_, and evaporated under reduced pressure to obtain the desired sulfonyl chlorides, which were used in the next step without any further purification.

### Synthesis of Compounds **1–50** (Schemes 1–5)

#### General Procedure (GP1) for the Synthesis of Sulfonamides

To a solution of proper amine (1.1 equiv) in dry THF or DCM (6.0 mL/equiv), TEA (3.0 equiv) and the appropriate sulfonyl chloride (1.0 equiv) were added, and the reaction mixture was stirred at room temperature overnight. The mixture was quenched by the addition of aq. HCl 2 N (5.0 mL/equiv) and extracted with EtOAc (3 × 10 mL). The combined organic phases were dried over Na_2_SO_4_, filtered, and evaporated under vacuo. The crude product was purified by flash chromatography or by recrystallization from Et_2_O to give the pure title compound.

#### General Procedure (GP2) for the Mitsunobu Reaction

To a solution of *tert*-butyl-hydroxy-azabicyclo carboxylate (1.05 equiv), substituted phenol (1.0 equiv), and PPh_3_ (1.05 equiv) in dry THF (10 mL/equiv), under a nitrogen atmosphere, at 0 °C, DIAD (1.05 equiv) was added dropwise. The reaction mixture was allowed to warm to room temperature and stirred overnight. Then, the mixture was quenched with aq. HCl 2 N (10 mL) and extracted with EtOAc (2 × 10 mL). The organic extracts were washed with brine, dried over Na_2_SO_4_, and concentrated under vacuo to give a crude residue, which was purified by flash chromatography to give the title compound.

#### General Procedure (GP3) for O-Substitution

To a solution of *tert*-butyl-hydroxy-azabicyclo carboxylate (1.0 equiv) and substituted heteroaryl chloride (1.0 equiv) in dry THF (10 mL/equiv), *t*-BuOK (1.0 equiv) was added. The reaction mixture was refluxed overnight, then quenched with water (10 mL) and extracted with DCM (2 × 10 mL). The organic extracts were washed with brine, dried over Na_2_SO_4_, and concentrated under vacuo to give a crude residue, which was purified by flash chromatography to give the title compound.

#### General Procedure (GP4) for the Boc-Deprotection

The appropriate *N*-Boc-substituted azabicyclo derivative (1.0 equiv) was treated at 0 °C with a 3:1 DCM/TFA mixture (4.0 mL/equiv) and the reaction was stirred at room temperature for 2 h. The crude mixture was concentrated under vacuo, and the subjected to three cycles of suspension (DCM, 10 mL)/concentration to obtain the desired product, which was used in the next step without any further purification.

##### 2-((1-((3,5-Dimethyl-1*H*-pyrazol-4-yl)sulfonyl)piperidin-4-yl)oxy)pyrazine (**1**)

Following GP1, commercially available 3,5-dimethyl-1*H*-pyrazole-4-sulfonyl chloride (**52k**) (0.045 g, 0.23 mmol) and 2-(piperidin-4-yloxy)pyrazine hydrochloride (**53**) (0.064 g, 0.25 mmol) were used in dry THF. Flash chromatography eluting with cyclohexane/acetone (8:2) gave the pure title compound (0.071 g, 91%) as a white solid. UPLC-MS: *t*_*R*_ = 2.86 min (generic method); MS (ESI) *m*/*z*: calcd. for C_14_H_20_N_5_O_3_S [M + H]^+^, 338.1; found, 338.1. ^1^H NMR (400 MHz, DMSO-*d*_6_): δ 13.07 (br s, 1H, NH), 8.26 (d, *J* = 1.3 Hz, 1H), 8.19–8,17 (m, 2H), 5.10–5.04 (m, 1H), 3.29–3.23 (m, 2H), 2.96–2.90 (m, 2H), 2.38 (s, 3H), 2.28 (s, 3H), 2.10–2.04 (m, 2H), 1.83–1.74 (m, 2H). ^13^C NMR (101 MHz, DMSO-*d*_6_): δ 159.3, 148.3, 143.3, 141.1, 137.3, 136.1, 111.1, 70.2, 43.2, 29.9, 13.8, 11.2. HRMS (ESI^+^) *m*/*z*: calcd. for C_14_H_20_N_5_O_3_S, 338.1287 [M + H]^+^; found, 338.129.

##### 2-((1-((3-Methyl-1*H*-pyrazol-4-yl)sulfonyl)piperidin-4-yl)oxy)pyrazine (**2**)

Following GP1, 3-methyl-1*H*-pyrazole-4-sulfonyl chloride (**52a**) (0.045 g, 0.25 mmol) and 2-(piperidin-4-yloxy)pyrazine hydrochloride (**53**) (0.06 g, 0.28 mmol) were used in dry THF. Flash chromatography eluting with cyclohexane/acetone (8:2) gave the pure title compound (0.038 g, 47%) as a white solid. UPLC-MS: *t*_*R*_ = 2.75 min (generic method); MS (ESI) *m*/*z*: calcd. for C_13_H_18_N_5_O_3_S [M + H]^+^, 324.1; found, 324.0. ^1^H NMR (400 MHz, CDCl_3_): δ 8.19 (s, 1H), 8.13 (d, *J* = 2.8 Hz, 1H), 8.05 (s, 1H), 7.85 (s, 1H), 5.18–5.15 (m, 1H), 3.32–3.27 (m, 2H), 3.20–3.14 (m, 2H), 2.56 (s, 3H), 2.17–2.10 (m, 2H), 2.04–1.96 (m, 2H). ^13^C NMR (101 MHz, DMSO-*d*_6_): δ 159.3, 141.1, 137.3, 136.1, 113.7, 70.2, 43.4, 29.9, 11.3. HRMS (ESI^+^) *m*/*z*: calcd. for C_13_H_18_N_5_O_3_S, 324.1130 [M + H]^+^; found, 324.1128.

##### 2-((1-((1*H*-Pyrazol-4-yl)sulfonyl)piperidin-4-yl)oxy)pyrazine (**3**)

Following GP1, 1*H*-pyrazole-4-sulfonyl chloride (**52b**) (0.045 g, 0.27 mmol) and 2-(piperidin-4-yloxy)pyrazine hydrochloride (**53**) (0.065 g, 0.30 mmol) were used in dry THF. Flash chromatography eluting with cyclohexane/acetone (8:2) gave the pure title compound (0.032 g, 38%) as a white solid. UPLC-MS: *t*_*R*_ = 2.58 min (generic method); MS (ESI) *m*/*z*: calcd. for C_12_H_16_N_5_O_3_S [M + H]^+^, 310.1; found, 310.0. ^1^H NMR (400 MHz, CDCl_3_): δ 8.18 (d, *J* = 1.4 Hz, 1H), 8.13 (d, *J* = 2.8 Hz, 1H), 8.05 (dd, *J* = 2.8, 1.4 Hz, 1H), 7.96 (s, 2H), 5.19–5.14 (s, 1H), 3.28–3.22 (m, 2H), 3.19–3.13 (m, 2H), 2.18–2.11 (m, 2H), 2.07–1.98 (m, 2H). ^13^C NMR (101 MHz, DMSO-*d*_6_): δ 159.3, 141.1, 137.3, 136.1, 135.3, 116.6, 70.1, 43.5, 29.8. HRMS (ESI^+^) *m*/*z*: calcd. for C_12_H_16_N_5_O_3_S, 310.0974 [M + H]^+^; found, 310.0974.

##### 2-((1-((1,3,5-Trimethyl-1*H*-pyrazol-4-yl)sulfonyl)piperidin-4-yl)oxy)pyrazine (**4**)

Following GP1, commercially available 1,3,5-trimethylpyrazole-4-sulfonyl chloride (**54a**) (0.045 g, 0.22 mmol) and 2-(piperidin-4-yloxy)pyrazine hydrochloride (**53**) (0.052 g, 0.24 mmol) were used in dry THF. Flash chromatography eluting with cyclohexane/acetone (8:2) gave the pure title compound (0.052 g, 67%) as a white solid. UPLC-MS: *t*_*R*_ = 3.15 min (generic method); MS (ESI) *m*/*z*: calcd. for C_15_H_22_N_5_O_3_S [M + H]^+^, 352.1; found, 352.1. ^1^H NMR (400 MHz, DMSO-*d*_6_): δ 8.26 (d, *J* = 1.3 Hz, 1H), 8.19 (d, *J* = 2.8 Hz, 1H), 8.17 (dd, *J* = 2.8, 1.3 Hz, 1H), 5.08–5.02 (m, 1H), 3.73 (s, 3H), 3.29–3.24 (m, 2H), 2.92–2.86 (m, 2H), 2.42 (s, 3H), 2.26 (s, 3H), 2.11–2.04 (m, 2H), 1.82–1.74 (m, 2H). ^13^C NMR (101 MHz, DMSO-*d*_6_): δ 159.3, 146.9, 142.8, 141.1, 137.3, 136.1, 111.6, 70.3, 43.2, 36.8, 30.0, 13.6, 11.0. HRMS (ESI^+^) *m*/*z*: calcd. for C_15_H_22_N_5_O_3_S, 352.1443 [M + H]^+^; found, 352.1441.

##### 3,5-Dimethyl-4-((4-(pyrazin-2-yloxy)piperidin-1-yl)sulfonyl)isoxazole (**5**)

Following GP1, commercially available 3,5-dimethylisoxazole-4-sulfonyl chloride (**54b**) (0.057 g, 0.29 mmol) and 2-(piperidin-4-yloxy)pyrazine hydrochloride (**53**) (0.07 g, 0.319 mmol) were used in dry THF. Recrystallization of the crude product from diethyl ether gave the pure title compound (0.032 g, 33%) as a white solid. UPLC-MS: *t*_*R*_ = 3.76 min (generic method); MS (ESI) *m*/*z*: calcd. for C_14_H_19_N_4_O_4_S [M + H]^+^, 339.1; found, 339.1. ^1^H NMR (400 MHz, DMSO-*d*_6_): δ 8.28 (d, *J* = 1.3 Hz, 1H), 8.20 (d, *J* = 2.8 Hz, 1H), 8.18 (dd, *J* = 2.8, 1.3 Hz, 1H), 5.14–5.08 (m, 1H), 3.40–3.34 (m, 2H), 3.11–3.05 (m, 2H), 2.63 (s, 3H), 2.35 (s, 3H), 2.13–2.06 (m, 2H), 1.84–1.76 (m, 2H). ^13^C NMR (101 MHz, DMSO-*d*_6_): δ 174.4, 159.3, 158.1, 141.1, 137.3, 136.2, 113.2, 69.9, 43.2, 30.0, 13.1, 11.4. HRMS (ESI^+^) *m*/*z*: calcd. for C_14_H_19_N_4_O_4_S, 339.1127 [M + H]^+^; found, 339.1135.

##### 2-((1-((1,4-Dimethyl-1*H*-pyrazol-5-yl)sulfonyl)piperidin-4-yl)oxy)pyrazine (**6**)

Following GP1, commercially available 2,4-dimethylpyrazole-3-sulfonyl chloride (**54c**) (0.051 g, 0.26 mmol) and 2-(piperidin-4-yloxy)pyrazine hydrochloride (**53**) (0.062 g, 0.28 mmol) were used in dry THF. Flash chromatography eluting with cyclohexane/*tert*-butyl methylether (7:3) gave the pure title compound (0.051 g, 58%) as a white solid. UPLC-MS: *t*_*R*_ = 3.67 min (generic method); MS (ESI) *m*/*z*: calcd. for C_14_H_20_N_5_O_3_S [M + H]^+^, 338.1; found, 338.1. ^1^H NMR (400 MHz, DMSO-*d*_6_): δ 8.27 (d, *J* = 1.3 Hz, 1H), 8.20–8.28 (m, 2H), 7.52 (s, 1H), 5.16–5.10 (s, 1H), 4.00 (s, 3H), 3.44–3.38 (m, 2H), 3.18–3.12 (m, 2H), 2.20 (s, 3H), 2.11–2.04 (m, 2H), 1.81–1.73 (m, 2H). ^13^C NMR (101 MHz, DMSO-*d*_6_): δ 159.2, 141.2, 139.6, 137.3, 136.2, 132.9, 122.0, 69.9, 42.9, 30.1, 23.8, 10.4. HRMS (ESI^+^) *m*/*z*: calcd. for C_14_H_20_N_5_O_3_S, 338.1287 [M + H]^+^; found, 338.1288.

##### 2-((1-((2,6-Dimethylphenyl)sulfonyl)piperidin-4-yl)oxy)pyrazine (**7**)

Following GP1, commercially available 2,6-dimethylbenzenesulfonyl chloride (**54d**) (0.057 g, 0.28 mmol) and 2-(piperidin-4-yloxy)pyrazine hydrochloride (**53**) (0.066 g, 0.308 mmol) were used in dry THF. Flash chromatography eluting with cyclohexane/*tert*-butyl methylether (7:3) gave the pure title compound (0.016 g, 16%) as a white solid. UPLC-MS: *t*_*R*_ = 4.80 min (generic method); MS (ESI) *m*/*z*: calcd. for C_17_H_22_N_3_O_3_S [M + H]^+^, 348.1; found, 348.1. ^1^H NMR (400 MHz, DMSO-*d*_6_): δ 8.29 (d, *J* = 1.1 Hz, 1H), 8.21–8.19 (m, 2H), 7.43 (t, *J* = 7.6 Hz, 1H), 7.28 (s, 2H), 5.22–5.16 (m, 1H), 3.40–3.34 (m, 2H), 3.15–3.09 (m, 2H), 2.61 (s, 6H), 2.06–1.99 (m, 2H), 1.78–1.70 (m, 2H). ^13^C NMR (151 MHz, DMSO-*d*_6_): δ 159.3, 141.1, 140.1, 137.3, 136.2, 135.2, 132.9, 131.8, 70.4, 41.7, 30.1, 22.9. HRMS (ESI^+^) *m*/*z*: calcd. for C_17_H_22_N_3_O_3_S, 348.1382 [M + H]^+^; found, 348.1376.

##### 2-((1-((3-Ethyl-5-methyl-1*H*-pyrazol-4-yl)sulfonyl)piperidin-4-yl)oxy)pyrazine (**8**)

Following GP1, 3-ethyl-5-methyl-1*H*-pyrazole-4-sulfonyl chloride (**52c**) (0.05 g, 0.24 mmol) and 2-(piperidin-4-yloxy)pyrazine hydrochloride (**53**) (0.057 g, 0.26 mmol) were used in dry THF. Flash chromatography eluting with cyclohexane/acetone (8:2) gave the pure title compound (0.065 g, 77%) as a white solid. UPLC-MS: *t*_*R*_ = 3.14 min (generic method); MS (ESI) *m*/*z*: calcd. for C_15_H_22_N_5_O_3_S [M + H]^+^: 352.1; found, 352.1. ^1^H NMR (400 MHz, DMSO-*d*_6_): δ 13.10 (br s, 1H, NH), 8.27 (d, *J* = 1.3 Hz, 1H), 8.19 (d, *J* = 2.8 Hz, 1H), 8.18 (dd, *J* = 2.8, 1.3 Hz, 1H), 5.11–5.05 (m, 1H), 3.29–3.23 (m, 2H), 2.96–2.90 (m, 2H), 2.76 (q, *J* = 7.5 Hz, 2H), 2.34 (s, 3H), 2.10–2.04 (m, 2H), 1.82–1.74 (m, 2H), 1.19 (t, *J* = 7.5 Hz, 3H). ^13^C NMR (101 MHz, DMSO-*d*_6_): δ 159.3, 141.1, 137.3, 136.1, 110.6, 70.2, 43.1, 30.1, 13.7. HRMS (ESI^+^) *m*/*z*: calcd. for C_15_H_22_N_5_O_3_S, 352.1443 [M + H]^+^; found, 352.1443.

##### 2-((1-((5-Methyl-3-propyl-1*H*-pyrazol-4-yl)sulfonyl)piperidin-4-yl)oxy)pyrazine (**9**)

Following GP1, 5-methyl-3-propyl-1*H*-pyrazole-4-sulfonyl chloride (**52d**) (0.05 g, 0.22 mmol) and 2-(piperidin-4-yloxy)pyrazine hydrochloride (**53**) (0.052 g, 0.24 mmol) were used in dry THF. Flash chromatography eluting with cyclohexane/acetone (8:2) gave the pure title compound, as a mixture of two rotamers (0.016 g, 20%), as a white solid. UPLC-MS: *t*_*R*_ = 3.51 min (generic method); MS (ESI) *m*/*z*: calcd. for C_16_H_24_N_5_O_3_S [M + H]^+^, 366.2; found, 366.1. ^1^H NMR (600 MHz, DMSO-*d*_6_): δ 13.09 (br s, 1H, NH), 8.26 (d, *J* = 1.4 Hz, 1H), 8.19 (d, *J* = 2.8 Hz, 1H), 8.18 (dd, *J* = 2.8, 1.4 Hz, 1H), 5.09–5.05 (m, 1H), 3.27–3.23 (m, 2H), 2.94–2.91 (m, 2H), 2.76 and 2.66 (br s, 2H), 2.38 and 2.29 (s, 3H), 2.08–204 (m, 2H), 1.80–1.75 (m, 2H), 1.67–1.61 (m, 2H), 0.91–0.90 (m, 3H). ^13^C NMR (151 MHz, DMSO-*d*_6_): δ 159.3, 152.2, 148.1, 147.2, 143.1, 141.1, 137.3, 136.2, 110.9, 70.2, 43.1, 30.1, 29.3, 26.9, 22.4, 22.1, 14.4, 14.1, 13.9, 11.3. HRMS (ESI^+^) *m*/*z*: calcd. for C_16_H_24_N_5_O_3_S, 366.1600 [M + H]^+^; found, 366.1600.

##### 2-((1-((3-Butyl-5-methyl-1*H*-pyrazol-4-yl)sulfonyl)piperidin-4-yl)oxy)pyrazine (**10**)

Following GP1, 3-butyl-5-methyl-1*H*-pyrazole-4-sulfonyl chloride (**52e**) (0.05 g, 0.21 mmol) and 2-(piperidin-4-yloxy)pyrazine hydrochloride (**53**) (0.05 g, 0.23 mmol) were used in dry THF. Flash chromatography eluting with cyclohexane/acetone (8:2) gave the pure title compound, as a mixture of two rotamers (0.065 g, 82%), as a white solid. UPLC-MS: *t*_*R*_ = 3.83 min (generic method); MS (ESI) *m*/*z*: calcd. for C_17_H_26_N_5_O_3_S [M + H]^+^, 380.2; found, 380.1. ^1^H NMR (600 MHz, DMSO-*d*_6_): δ 13.11 (br s, 1H, NH), 8.26 (d, *J* = 1.3 Hz, 1H), 8.19 (d, *J* = 2.8 Hz, 1H), 8.17 (dd, *J* = 2.8, 1.4 Hz, 1H), 5.08–5.04 (m, 1H), 3.26–3.22 (m, 2H), 2.91 (t, *J* = 10.1 Hz, 2H), 2.79–2.76 and 2.69–2.66 (m, 2H), 2.37 and 2.28 (s, 3H), 2.07–2.04 (m, 2H), 1.80–1.74 (m, 2H), 1.60–1.59 (m, 2H), 1.34–1.27 (m, 2H), 0.88 (t, *J* = 7.4 Hz, 3H).·^13^C NMR (151 MHz, DMSO-*d*_6_): δ 159.3, 152.3, 148.1, 147.4, 143.1, 141.1, 137.3, 137.3, 136.2, 110.8, 70.2, 43.1, 31.2, 30.9, 30.0, 26.9, 24.7, 22.4, 22.3, 14.3, 14.1, 13.9, 11.3. HRMS (ESI^+^) *m*/*z*: calcd. for C_17_H_26_N_5_O_3_S, 380.1756 [M + H]^+^; found, 380.1750.

##### 2-((1-((3-*iso*Propyl-5-methyl-1*H*-pyrazol-4-yl)sulfonyl)piperidin-4-yl)oxy)pyrazine (**11**)

Following GP1, 3-*iso*propyl-5-methyl-1*H*-pyrazole-4-sulfonyl chloride (**52f**) (0.035 g, 0.16 mmol) and 2-(piperidin-4-yloxy)pyrazine hydrochloride (**53**) (0.04 g, 0.176 mmol) were used in dry THF. Flash chromatography eluting with cyclohexane/acetone (8:2) gave the pure title compound (0.035 g, 60%) as a white solid. UPLC-MS: *t*_*R*_ = 3.48 min (generic method); MS (ESI) *m*/*z*: calcd. for C_16_H_24_N_5_O_3_S [M + H]^+^, 366.2; found, 366.1. ^1^H NMR (400 MHz, DMSO-*d*_6_): δ ^1^H NMR (400 MHz, DMSO-*d*_6_): δ 13.10 (br s, 1H, NH), 8.26 (d, *J* = 1.3 Hz, 1H), 8.19 (d, *J* = 2.8 Hz, 1H), 8.18 (dd, *J* = 2.8, 1.3 Hz, 1H), 5.11–5.06 (m, 1H), 3.26–3.22 (m, 2H), 2.95–2.89 (m, 2H), 2.34–2.33 (m, 4H), 2.09–2.04 (m, 2H), 1.82–1.73 (m, 2H), 1.22 (d, *J* = 6.9 Hz, 6H). ^13^C NMR (101 MHz, DMSO-*d*_6_): δ 159.3, 158.0, 141.1, 137.3, 136.1, 109.9, 70.3, 42.9, 30.1, 22.9. HRMS (ESI^+^) *m*/*z*: calcd. for C_16_H_24_N_5_O_3_S, 366.1600 [M + H]^+^; found, 366.1599.

##### 2-((1-((3-(*tert*-Butyl)-5-methyl-1*H*-pyrazol-4-yl)sulfonyl)piperidin-4-yl)oxy)pyrazine (**12**)

Following GP1, 3-*tert*-butyl-5-methyl-1*H*-pyrazole-4-sulfonyl chloride (**52g**) (0.05 g, 0.21 mmol) and 2-(piperidin-4-yloxy)pyrazine hydrochloride (**53**) (0.05 g, 0.23 mmol) were used in dry THF. Flash chromatography eluting with cyclohexane/acetone (8:2) gave the pure title compound (0.060 g, 75%) as a white solid. UPLC-MS: *t*_*R*_ = 3.84 min (generic method); MS (ESI) *m*/*z*: calcd. for C_17_H_26_N_5_O_3_S [M + H]^+^, 380.2; found, 380.2. ^1^H NMR (400 MHz, DMSO-*d*_6_): δ 13.02 (br s, 1H, NH), 8.29 (d, *J* = 1.0 Hz, 1H), 8.20–8.19 (m, 2H), 5.19–5.13 (m, 1H), 3.30–3.27 (m, 2H), 3.08–3.03 (m, 2H), 2.37 (s, 3H), 2.04–1.99 (m, 2H), 1.77–1.70 (m, 2H), 1.36 (s, 9H). ^13^C NMR (101 MHz, DMSO-*d*_6_): δ 159.3, 141.1, 137.3, 136.2, 70.4, 41.9, 34.2, 30.2, 29.9, 11.9. HRMS (ESI^+^) *m*/*z*: calcd. for C_17_H_26_N_5_O_3_S, 380.1756 [M + H]^+^; found, 380.1749.

##### 2-((1-((3-Methoxy-5-methyl-1*H*-pyrazol-4-yl)sulfonyl)piperidin-4-yl)oxy)pyrazine (**13**)

Following GP1, 3-methoxy-5-methyl-1*H*-pyrazole-4-sulfonyl chloride (**52h**) (0.045 g, 0.21 mmol) and 2-(piperidin-4-yloxy)pyrazine hydrochloride (**53**) (0.05 g, 0.23 mmol) were used in dry THF. Flash chromatography eluting with cyclohexane/acetone (8:2) gave the pure title compound (0.035 g, 47%) as a white solid. UPLC-MS: *t*_*R*_ = 2.77 min (generic method); MS (ESI) *m*/*z*: calcd. for C_14_H_20_N_5_O_4_S [M + H]^+^, 354.1; found, 354.1. ^1^H NMR (400 MHz, DMSO-*d*_6_): δ 12.55 (br s, 1H, NH), 8.27 (d, *J* = 1.3 Hz, 1H), 8.19 (d, *J* = 2.8 Hz, 1H), 8.18 (dd, *J* = 2.8, 1.3 Hz, 1H), 5.09–5.03 (m, 1H), 3.83 (s, 3H), 3.20–3.25 (m, 2H), 2.32 (s, 3H), 2.95–2.88 (m, 2H), 2.09–2.02 (m, 2H), 1.80–1.71 (m, 2H). ^13^C NMR (151 MHz, DMSO-*d*_6_): δ 160.7, 159.3, 144.1, 143.9, 141.2, 137.3, 136.1, 98.6, 70.4, 56.5, 56.5, 43.4, 30.0, 11.7. HRMS (ESI^+^) *m*/*z*: calcd. for C_14_H_20_N_5_O_4_S, 354.1236 [M + H]^+^; found, 354.1237.

##### 2-((1-((5-Methyl-3-(trifluoromethyl)-1*H*-pyrazol-4-yl)sulfonyl)piperidin-4-yl)oxy)pyrazine (**14**)

Following GP1, 5-methyl-3-(trifluoromethyl)-1*H*-pyrazole-4-sulfonyl chloride (**52i**) (0.03 g, 0.12 mmol) and 2-(piperidin-4-yloxy)pyrazine hydrochloride (**53**) (0.03 g, 0.132 mmol) were used in dry THF. Flash chromatography eluting with cyclohexane/acetone (8:2) gave the pure title compound (0.041 g, 87%) as a white solid. UPLC-MS: *t*_*R*_ = 3.64 min (generic method); MS (ESI) *m*/*z*: calcd. for C_14_H_17_F_3_N_5_O_3_S [M + H]^+^, 392.1; found, 392.1. ^1^H NMR (400 MHz, DMSO-*d*_6_): δ 14.16 (br s, 1H, NH), 8.27 (d, *J* = 1.3 Hz, 1H), 8.19 (d, *J* = 2.8 Hz, 1H), 8.18 (dd, *J* = 2.8, 1.3 Hz, 1H), 5.13–5.08 (m, 1H), 3.39–3.33 (m, 2H), 3.10–3.04 (m, 2H), 2.49 (s, 3H), 2.09–2.04 (m, 2H), 1.81–1.73 (m, 2H). ^13^C NMR (101 MHz, DMSO-*d*_6_): δ 159.3, 145.9, 141.1, 139.7, 137.3, 136.2, 123.5 (q, *J* = 269.1 Hz), 113.4, 70.1, 43.0, 30.1, 11.3. ^19^F NMR (565 MHz, DMSO-*d*_6_): δ −57.94. HRMS (ESI^+^) *m*/*z*: calcd. for C_14_H_17_F_3_N_5_O_3_S, 392.1004 [M + H]^+^; found, 392.1007.

##### 2-((1-((3,5-Diethyl-1*H*-pyrazol-4-yl)sulfonyl)piperidin-4-yl)oxy)pyrazine (**15**)

Following GP1, 3,5-diethyl-1*H*-pyrazole-4-sulfonyl chloride (**52j**) (0.03 g, 0.13 mmol) and 2-(piperidin-4-yloxy)pyrazine hydrochloride (**53**) (0.03 g, 0.132 mmol) were used in dry THF. Flash chromatography eluting with cyclohexane/acetone (8:2) gave the pure title compound (0.04 g, 84%) as a white solid. UPLC-MS: *t*_*R*_ = 3.43 min (generic method); MS (ESI) *m*/*z*: calcd. for C_16_H_24_N_5_O_3_S [M + H]^+^, 366.2; found, 366.2. ^1^H NMR (400 MHz, DMSO-*d*_6_): δ 13.10 (br s, 1H, NH), 8.26 (d, *J* = 1.3 Hz, 1H), 8.19 (d, *J* = 2.8 Hz, 1H), 8.17 (dd, *J* = 2.8, 1.3 Hz, 1H), 5.11–5.05 (m, 1H), 3.29–3.23 (m, 2H), 2.96–2.90 (m 2H), 2.09–2.02 (m, 2H), 1.81–1.73 (m, 2H), 2.82 (q, *J* = 7.5 Hz, 2H), 2.72 (q, *J* = 7.5 Hz, 2H), 1.20 (q, *J* = 7.5 Hz, 6H). ^13^C NMR (101 MHz, DMSO-*d*_6_): δ 159.3, 153.2, 148.6, 141.1, 137.3, 136.2, 109.9, 70.3, 42.9, 30.1, 20.8, 18.5, 13.9, 13.5. HRMS (ESI^+^) *m*/*z*: calcd. for C_16_H_24_N_5_O_3_S, 366.1600 [M + H]^+^; found, 366.1605.

##### (3,5-Dimethyl-1*H*-pyrazol-4-yl)(4-(pyrazin-2-yloxy)piperidin-1-yl)methanone (**16**)

Under a nitrogen atmosphere, to a solution of 3,5-dimethyl-1*H*-pyrazole-4-carboxylic acid (**55**) (0.06 g, 0.43 mmol, 1.0 equiv) in dry DCM/DMF (3/1 mL), TEA (0.19 mL, 1.38 mmol, 3.2 equiv) was added at room temperature. Subsequently, HBTU (0.178 g, 0.47 mmol, 1.1 equiv) was added and the reaction mixture was stirred for 30 min. Then, 2-(piperidin-4-yloxy)pyrazine hydrochloride (**53**) (0.102 g, 0.47 mmol, 1.1 equiv) was added and the reaction stirred at room temperature for further 48 h. Water was added (10 mL) and the mixture was extracted with EtOAc (3 × 10 mL). The combined organic phases were dried over Na_2_SO_4_, filtered, and evaporated under vacuo. The obtained residue was purified by flash chromatography eluting with cyclohexane/acetone (3:7) to give the pure title compound (0.053 g, 41%) as a white solid. UPLC-MS: *t*_*R*_ = 2.13 min (generic method); MS (ESI) *m*/*z*: calcd. for C_15_H_20_N_5_O_2_ [M + H]^+^, 302.2; found, 302.0. ^1^H NMR (400 MHz, DMSO-*d*_6_): δ 12.41 (br s, 1H, NH), 8.30 (d, *J* = 1.0 Hz, 1H), 8.22–8.20 (m, 2H), 5.28–5.22 (m, 1H), 3.79 (br s, 2H), 3.37–3.33 (m, 2H), 2.18 (s, 3H), 2.10 (s, 3H), 2.02–1.99 (m, 2H), 1.67–1.58 (m, 2H). ^13^C NMR (151 MHz, DMSO-*d*_6_): δ 165.6, 159.4, 146.24, 141.17, 138.46, 137.24, 136.27, 136.23, 113.04, 71.40, 31.16, 12.98, 10.32. HRMS (ESI^+^) *m*/*z*: calcd. for C_15_H_20_N_5_O_2_, 302.1617 [M + H]^+^; found, 302.1604.

##### 3,5-Dimethyl-*N*-((1*r*,4*r*)-4-(pyrazin-2-yloxy)cyclohexyl)-1*H*-pyrazole-4-sulfonamide (**17**)

Following GP1, 3,5-dimethyl-1*H*-pyrazole-4-sulfonyl chloride (**52k**) (0.05 g, 0.26 mmol) and (1*r*, 4*r*)-4-(pyrazin-2-yloxy)cyclohexan-1-amine hydrochloride (**56**) (0.07 g, 0.286 mmol) were used in dry THF. Flash chromatography eluting with DCM/MeOH (95:5) gave the pure title compound (0.05 g, 55%) as a white solid. UPLC-MS: *t*_*R*_ = 2.66 min (generic method); MS (ESI) *m*/*z*: calcd. for C_15_H_22_N_5_O_3_S [M + H]^+^, 352.1; found, 352.1. ^1^H NMR (400 MHz, DMSO-*d*_6_): δ 12.82 (br s, 1H, NH), 8.21 (d, *J* = 1.2 Hz, 1H), 8.18–8.16 (m, 2H), 7.35 (d, *J* = 7.6 Hz, 1H), 4.89–4.82 (m, 1H), 3.01–2.93 (m, 1H), 2.34 (s, 6H), 2.03–1.99 (m, 2H), 1.73–1.70 (m, 2H), 1.48–1.29 (m, 4H). ^13^C NMR (151 MHz, DMSO-*d*_6_): δ 159.6, 141.2, 136.9, 136.1, 116.7, 73.2, 50.9, 30.8, 29.8. HRMS (ESI^+^) *m*/*z*: calcd. for C_15_H_22_N_5_O_3_S, 352.1443 [M + H]^+^; found, 352.1434.

##### 2-((1-((3,5-Dimethyl-1*H*-pyrazol-4-yl)sulfonyl)azetidin-3-yl)oxy)pyrazine (**18**)

Following GP1, 3,5-dimethyl-1*H*-pyrazole-4-sulfonyl chloride (**52k**) (0.25 g, 0.13 mmol) and 2-(azetidin-3-yloxy)pyrazine trifluoroacetate (**A**) 0.04 g, 0.14 mmol) were used in dry THF. Flash chromatography eluting with DCM/MeOH (95:5) gave the pure title compound (0.19 g, 47%) as a white solid. UPLC-MS: *t*_*R*_ = 2.41 min (generic method); MS (ESI) *m*/*z*: calcd. for C_12_H_16_N_5_O_3_S [M + H]^+^, 310.1; found, 310.0. ^1^H NMR (400 MHz, DMSO-*d*_6_): δ 13.20 (br s, 1H, NH), 8.34 (d, *J* = 1.4 Hz, 1H), 8.27 (d, *J* = 2.8 Hz, 1H), 8.19 (dd, *J* = 2.8, 1.4 Hz, 1H), 5.30–5.24 (m, 1H), 4.12–4.08 (m, 2H), 3.67–3.63 (m, 2H), 2.39 (s, 3H), 2.29 (s, 3H). ^13^C NMR (101 MHz, DMSO-*d*_6_): δ 158.6, 148.8, 144.0, 141.3, 138.2, 135.7, 109.3, 64.0, 56.7, 13.8, 11.2. HRMS (ESI^+^) *m*/*z*: calcd. for C_12_H_16_N_5_O_3_S, 310.0974 [M + H]^+^; found, 310.0973.

##### *N*,3,5-Trimethyl-*N*-(3-(pyrazin-2-yloxy)propyl)-1*H*-pyrazole-4-Sulfonamide (**19**)

Following GP1, 3,5-dimethyl-1*H*-pyrazole-4-sulfonyl chloride (**52k**) (0.1 g, 0.51 mmol) and *N*-methyl-3-(pyrazin-2-yloxy)propan-1-amine trifluoroacetate (**B**) (0.016 g, 0.56 mmol) were used in dry THF. Flash chromatography eluting with DCM/MeOH (95:5) gave the pure title compound (0.056 g, 34%) as a white solid. UPLC-MS: *t*_*R*_ = 2.64 min (generic method); MS (ESI) *m*/*z*: calcd. for C_13_H_20_N_5_O_3_S [M + H]^+^, 326.1; found, 326.0. ^1^H NMR (400 MHz, DMSO-*d*_6_): δ 12.99 (br s, 1H, *NH*), 8.27 (br s, 1H), 8.21 (br s, 2H), 4.31 (t, *J* = 6.3 Hz, 2H), 3.11 (t, *J* = 7.0 Hz, 2H), 2.68 (s, 3H), 2.36 (s, 3H), 2.25 (s, 3H), 2.00–1.93 (m, 2H). ^13^C NMR (101 MHz, DMSO-*d*_6_): δ 160.1, 141.2, 137.2, 135.8, 112.6, 63.8, 46.4, 34.5, 26.8, 13.7, 11.1. HRMS (ESI^+^) *m*/*z*: calcd. for C_13_H_20_N_5_O_3_S, 326.1287 [M + H]^+^; found, 326.1290.

##### *tert*-Butyl (1*R*,3*r*,5*S*)-3-(pyrazin-2-yloxy)-8-azabicyclo[3.2.1]octane-8-carboxylate (**74a**)

Following GP3, commercially available *tert*-butyl (1*R*,3*r*,5*S*)-3-hydroxy-8-azabicyclo[3.2.1]octane-8-carboxylate (**65a**) (0.2 g, 0.88 mmol) and 2-chloropyrazine (**73a**) (0.1 g, 0.88 mmol) were used. Flash chromatography eluting with cyclohexane/EtOAc (5:5) gave the pure title compound (0.1 g, 37%) as a colorless oil. UPLC-MS: *t*_*R*_ = 1.16 min (generic method); MS (ESI) *m*/*z*: calcd. for C_16_H_24_N_3_O_3_ [M + H]^+^, 306.2; found, 306.1. ^1^H NMR (400 MHz, DMSO-*d*_6_): δ 8.26 (d, *J* = 1.0 Hz, 1H), 8.19–8.15 (m, 2H), 5.29 (t, *J* = 5.0 Hz, 1H), 4.12–4.03 (m, 2H), 2.14–2.00 (m, 4H), 1.86 (t, *J* = 14.9 Hz, 4H), 1.39 (s, 9H).

##### (1*R*,3*r*,5*S*)-3-(Pyrazin-2-yloxy)-8-azabicyclo[3.2.1]octane Trifluoroacetate (**78a**)

Following GP4, *tert*-butyl (1*R*,3*r*,5*S*)-3-(pyrazin-2-yloxy)-8-azabicyclo[3.2.1]octane-8-carboxylate (**74a**) was used to give the title compound (*quant*.), which was used in the next step without any purification.

##### (1*R*,3*r*,5*S*)-8-((3,5-Dimethyl-1*H*-pyrazol-4-yl)sulfonyl)-3-(pyrazin-2-yloxy)-8-azabicyclo[3.2.1]octane (**20**)

Following GP1, 3,5-dimethyl-1*H*-pyrazole-4-sulfonyl chloride (**52k**) (0.06 g, 0.31 mmol) and (1*R*,3*r*,5*S*)-3-(pyrazin-2-yloxy)-8-azabicyclo[3.2.1]octane trifluoroacetate (**78a**) (0.11 g, 0.34 mmol) were used in dry THF. Flash chromatography eluting with DCM/MeOH (95:5) gave the pure title compound (0.035 g, 31%) as a white solid. UPLC-MS: *t*_*R*_ = 3.03 min (generic method); MS (ESI) *m*/*z*: calcd. for C_16_H_22_N_5_O_3_S [M + H]^+^, 364.1; found, 364.1. ^1^H NMR (400 MHz, DMSO-*d*_6_): δ 8.23 (d, *J* = 1.1 Hz, 1H), 8.17–8.16 (m, 2H), 5.26 (t, *J* = 5.0 Hz, 1H), 4.12–4.10 (m, 2H), 2.31 (s, 6H), 2.15–1.91 (m, 6H), 1.63–1.60 (m, 2H). ^13^C NMR (101 MHz, DMSO-*d*_6_): δ 159.2, 141.2, 137.1, 136.4, 114.9, 68.8, 55.4, 37.3, 28.4, 12.2. HRMS (ESI^+^) *m*/*z*: calcd. for C_16_H_22_N_5_O_3_S, 364.1443 [M + H]^+^; found, 364.1443.

##### *tert*-Butyl (1*R*,3*s*,5*S*)-3-(pyrazin-2-yloxy)-8-azabicyclo[3.2.1]octane-8-carboxylate (**75a**)

Following GP3, commercially available *tert*-butyl (1*R*,3*s*,5*S*)-3-hydroxy-8-azabicyclo[3.2.1]octane-8-carboxylate (**65b**) (0.2 g, 0.88 mmol) and 2-chloropyrazine (**73a**) (0.1 g, 0.88 mmol) were used. Flash chromatography eluting with DCM/MeOH (95:5) gave the pure title compound (0.096 g, 36%) as a white solid. UPLC-MS: *t*_R_ = 2.27 min (generic method); MS (ESI) *m*/*z*: calcd. for C_16_H_24_N_3_O_3_ [M + H]^+^, 306.2; found, 306.2. ^1^H NMR (400 MHz, DMSO-*d*_6_): δ 8.36–8.35 (m, 2H), 8.24 (d, *J* = 1.1 Hz, 1H), 4.52–4.46 (m, 1H), 3.82–3.77 (m, 2H), 2.07–1.88 (m, 4H), 1.93–1.88 (m, 2H), 1.77–1.71 (m, 2H).

##### (1*R*,3*s*,5*S*)-3-(Pyrazin-2-yloxy)-8-azabicyclo[3.2.1]octane Trifluoroacetate (**79a**)

Following GP4, *tert*-butyl (1*R*,3*s*,5*S*)-3-(pyrazin-2-yloxy)-8-azabicyclo[3.2.1]octane-8-carboxylate (**75a**) was used to give the title compound (*quant*.), which was used in the next step without any purification.

##### (1*R*,3*s*,5*S*)-8-((3,5-Dimethyl-1*H*-pyrazol-4-yl)sulfonyl)-3-(pyrazin-2-yloxy)-8-azabicyclo[3.2.1]octane (**21**)

Following GP1, 3,5-dimethyl-1*H*-pyrazole-4-sulfonyl chloride (**52k**) (0.03 g, 0.15 mmol) and (1*R*,3*s*,5*S*)-3-(pyrazin-2-yloxy)-8-azabicyclo[3.2.1]octane trifluoroacetate (**79a**) (0.053 g, 0.165 mmol) were used in dry THF. Flash chromatography eluting with DCM/MeOH (95:5) gave the pure title compound (0.041 g, 75%) as a white solid. UPLC-MS: *t*_*R*_ = 3.02 min (generic method); MS (ESI) *m*/*z*: calcd. for C_16_H_22_N_5_O_3_S [M + H]^+^, 364.1; found, 364.1. ^1^H NMR (400 MHz, DMSO-*d*_6_): δ 13.00 (br s, 1H, *NH*), 8.25 (d, *J* = 1.0 Hz, 1H), 8.20–8.18 (m, 2H), 5.37–5.29 (m, 1H), 4.20–4.19 (m, 2H), 2.33 (s, 6H), 2.25–2.20 (m, 2H), 1.78–1.63 (m, 6H). ^13^C NMR (101 MHz, DMSO-*d*_6_): δ 159.4, 141.2, 137.3, 136.1, 114.8, 68.4, 55.8, 37.9, 28.6, 11.6. HRMS (ESI^+^) *m*/*z*: calcd. for C_16_H_22_N_5_O_3_S, 364.1443 [M + H]^+^; found, 364.1444.

##### *N*-(1-((3,5-Dimethyl-1*H*-pyrazol-4-yl)sulfonyl)piperidin-4-yl)pyrazin-2-amine (**22**)

Following GP1, 3,5-dimethyl-1*H*-pyrazole-4-sulfonyl chloride (**52k**) (0.056 g, 0.29 mmol) and *N*-(piperidin-4-yl)pyrazin-2-amine trifluoroacetate (**C**) (0.076 g, 0.32 mmol) were used in dry THF. Flash chromatography eluting with cyclohexane/acetone (3:7) gave the pure title compound (0.069 g, 71%) as a white solid. UPLC-MS: *t*_*R*_ = 2.32 min (generic method); MS (ESI) *m*/*z*: calcd. for C_14_H_21_N_6_O_2_S [M + H]^+^, 337.1; found, 337.1. ^1^H NMR (400 MHz, DMSO-*d*_6_): δ 12.05 (br s, 1H, NH), 7.89–7.88 (m, 2H), 7.63 (d, *J* = 2.6 Hz, 1H), 7.02 (d, *J* = 7.2 Hz, 1H), 3.75–3.67 (m, 1H), 3.48–3.45 (m, 2H), 2.68–2.58 (m, 2H), 2.37 (s, 3H), 2.28 (s, 3H), 2.00–1.95 (m, 2H), 1.54–1.44 (m, 2H). ^13^C NMR (101 MHz, DMSO-*d*_6_): δ 154.7, 148.2, 143.1, 141.9, 133.9, 131.5, 111.3, 46.5, 44.7, 30.9, 13.8, 11.2. HRMS (ESI^+^) *m*/*z*: calcd. for C_14_H_21_N_6_O_2_S, 337.1447 [M + H]^+^; found, 337.1449.

##### 2-((1-((3,5-Dimethyl-1*H*-pyrazol-4-yl)sulfonyl)piperidin-4-yl)methyl)pyrazine (**23**)

Following GP1, 3,5-dimethyl-1*H*-pyrazole-4-sulfonyl chloride (**52k**) (0.089 g, 0.46 mmol) and 2-(piperidin-4-ylmethyl)pyrazine hydrochloride (**D**) (0.012 g, 0.51 mmol) were used in dry THF. Flash chromatography eluting with cyclohexane/acetone (6:4) gave the pure title compound (0.077 g, 50%) as a white solid. UPLC-MS: *t*_*R*_ = 2.49 min (generic method); MS (ESI) *m*/*z*: calcd. for C_15_H_22_N_5_O_2_S [M + H]^+^, 336.1; found, 336.0. ^1^H NMR (400 MHz, DMSO-*d*_6_): δ 13.03 (br s, 1H, NH), 8.55–8.54 (m, 1H), 8.52 (d, *J* = 1.5 Hz, 1H), 8.46 (d, *J* = 2.5 Hz, 1H), 3.57–3.53 (m, 2H), 2.70 (d, *J* = 7.1 Hz, 2H), 2.30–2.24 (m, 8H), 1.80–1.71 (m, 1H), 1.66–1.61 (m, 2H), 1.31–1.20 (m, 2H). ^13^C NMR (101 MHz, DMSO-*d*_6_): δ 155.9, 145.4, 144.5, 142.8, 111.1, 45.8, 41.1, 35.2, 31.1, 13.9, 11.2. HRMS (ESI^+^) *m*/*z*: calcd. for C_15_H_22_N_5_O_2_S, 336.1494 [M + H]^+^; found, 336.1495.

##### 2-(1-((3,5-Dimethyl-1*H*-pyrazol-4-yl)sulfonyl)piperidin-4-yl)pyrazine (**24**)

Following GP1, 3,5-dimethyl-1*H*-pyrazole-4-sulfonyl chloride (**52k**) (0.02 g, 0.1 mmol) and 2-(piperidin-4-yl)pyrazine hydrochloride (**E**) (0.03 g, 0.11 mmol) were used in dry THF. Flash chromatography eluting with DCM/MeOH (98:2) gave the pure title compound (0.028 g, 87%) as a white solid. UPLC-MS: *t*_*R*_ = 2.34 min (generic method); MS (ESI) *m*/*z*: calcd. for C_14_H_20_N_5_O_2_S [M + H]^+^, 322.1; found, 322.1. ^1^H NMR (400 MHz, DMSO-*d*_6_): δ 8.58–8.56 (m, 2H), 8.49 (d, *J* = 2.3 Hz, 1H), 3.74–3.09 (m, 2H), 2.86–2.78 (m, 1H), 2.47–2.43 (m, 2H), 2.34 (s, 6H), 1.98–1.94 (m, 2H), 1.82–1.71 (m, 2H). ^13^C NMR (101 MHz, DMSO-*d*_6_): δ 159.5, 155.8, 144.4, 144.0, 143.3, 116.0, 111.1, 45.9, 30.6, 12.5. HRMS (ESI^+^) *m*/*z*: calcd. for C_14_H_20_N_5_O_2_S, 322.1338 [M + H]^+^; found, 322.1335.

##### *tert*-Butyl-(1*R*,3*r*,5*S*)-3-hydroxy-9-azabicyclo[3.3.1]nonane-9-carboxylate (**57a**)

*tert*-Butyl-(1*R*,3*r*,5*S*)-3-hydroxy-9-azabicyclo[3.3.1]nonane-9-carboxylate (**57a**) was prepared using a previously reported procedure. Analytical and spectroscopic data are in agreement with those previously reported.^49^

##### *tert*-Butyl (1*R*,3*r*,5*S*)-3-(pyrazin-2-yloxy)-9-azabicyclo[3.3.1]nonane-9-carboxylate (**58a**)

Following GP3, *tert*-butyl (1*R*,3*r*,5*S*)-3-hydroxy-9-azabicyclo[3.3.1]nonane-9-carboxylate (**57a**) (0.2 g, 0.83 mmol) and 2-chloropyrazine (**73a**) (0.095 g, 0.83 mmol) were used. Flash chromatography eluting with cyclohexane/EtOAc (5:5) gave the pure title compound (0.09 g, 34%) as a colorless oil. UPLC-MS: *t*_R_ = 2.77 (generic method); MS (ESI) *m*/*z*: calcd. for C_17_H_26_N_3_O_3_ [M + H]^+^, 320.2; found, 320.1 ^1^H NMR (400 MHz, DMSO-*d*_6_): δ 8.27 (d, *J* = 1.3 Hz, 1H), 8.23–8.17 (m, 2H), 5.02–4.92 (m, 1H), 4.35 (t, *J* = 13.4 Hz, 2H), 2.46–2.29 (m, 2H), 2.28–2.13 (m, 1H), 1.65–1.45 (m, 7H), 1.43 (s, 9H).

##### (1*R*,3*r*,5*S*)-3-(Pyrazin-2-yloxy)-9-azabicyclo[3.3.1]nonane Trifluoroacetate (**59a**)

Following GP4 *tert*-butyl (1*R*,3*r*,5*S*)-3-(pyrazin-2-yloxy)-9-azabicyclo[3.3.1]nonane-9-carboxylate (**58a**) was used to give the title compound (*quant*.), which was used in the next step without any purification.

##### (1*R*,3*r*,5*S*)-9-((3,5-Dimethyl-1*H*-pyrazol-4-yl)sulfonyl)-3-(pyrazin-2-yloxy)-9-azabicyclo[3.3.1]nonane (**25**)

Following GP1, 3,5-dimethyl-1*H*-pyrazole-4-sulfonyl chloride (**52k**) (0.05 g, 0.26 mmol) and (1*R*,3*r*,5*S*)-3-(pyrazin-2-yloxy)-9-azabicyclo[3.3.1]nonane trifluoroacetate (**59a**) (0.095 g, 0.29 mmol) were used in dry THF. Flash chromatography eluting with DCM/MeOH (98:2) gave the pure title compound (0.02 g, 20%) as a white solid. UPLC-MS: *t*_R_ = 3.48 (generic method); MS (ESI) *m*/*z*: calcd. for C_17_H_24_N_5_O_3_S [M + H]^+^, 378.2; found, 378.1. ^1^H NMR (400 MHz, DMSO-*d*_6_): δ 12.95 (s, 1H, NH), 8.24 (d, *J* = 1.3 Hz, 1H), 8.22–819 (m, 2H), 4.97–4.90 (m, 1H), 4.16–4.13 (m, 2H), 2.41–2.18 (m, 9H), 1.60–1.46 (m, 7H). ^13^C NMR (151 MHz, DMSO-*d*_6_): δ 159.3, 141.3, 137.2, 135.9, 116.1, 67.6, 46.6, 31.3, 30.3, 14.1. HRMS (ESI^+^) *m*/*z*: calcd. for C_17_H_24_N_5_O_3_S, 378.1600 [M + H]^+^; found, 378.1589.

##### *tert*-Butyl (1*R*,5*S*,7*s*)-7-hydroxy-3-oxa-9-azabicyclo[3.3.1]nonane-9-carboxylate (**57b**)

*tert*-Butyl (1*R*,5*S*,7*s*)-7-hydroxy-3-oxa-9-azabicyclo[3.3.1]nonane-9-carboxylate (**57b**) was prepared using a previously reported procedure. Analytical and spectroscopic data are in agreement with those previously reported.^50^

##### *tert*-Butyl (1*R*,5*S*,7*s*)-7-(Pyrazin-2-yloxy)-3-oxa-9-azabicyclo[3.3.1]nonane-9-carboxylate (**58b**)

Following GP3, *tert*-butyl (1*R*,5*S*,7*s*)-7-hydroxy-3-oxa-9-azabicyclo[3.3.1]nonane-9-carboxylate (**57b**) (0.2 g, 0.83 mmol) and 2-chloropyrazine (**73a**) (0.095 g, 0.83 mmol) were used. Flash chromatography eluting with cyclohexane/EtOAc (2:8) gave the pure title compound (0.14 g, 53%) as a colorless oil. UPLC-MS: *t*_R_ = 2.24 (generic method); MS (ESI) *m*/*z*: calcd. for C_16_H_24_N_3_O_4_ [M + H]^+^, 322.2; found, 322.1. ^1^H NMR (400 MHz, DMSO-*d*_6_): δ 8.22 (d, *J* = 1.3 Hz, 1H), 8.21–8.18 (m, 1H), 8.18–8.16 (m, 1H), 5.03–4.95 (m, 1H), 4.13–3.99 (m, 2H), 3.67–3.60 (m, 2H), 3.47 (dd, *J* = 11.1, 2.5 Hz, 2H), 2.44–2.31 (m, 2H), 1.84–1.71 (m, 2H), 1.44 (s, 9H).

##### (1*R*,5*S*,7*s*)-7-(Pyrazin-2-yloxy)-3-oxa-9-azabicyclo[3.3.1]nonane Trifluoroacetate (**59b**)

Following GP4, *tert*-butyl (1*R*,5*S*,7*s*)-7-(pyrazin-2-yloxy)-3-oxa-9-azabicyclo[3.3.1]nonane-9-carboxylate (**58b**) was used to give the title compound (*quant*.), which was used in the next step without any purification.

##### (1*R*,5*S*,7*s*)-9-((3,5-Dimethyl-1*H*-pyrazol-4-yl)sulfonyl)-7-(pyrazin-2-yloxy)-3-oxa-9-azabicyclo[3.3.1]nonane (**26**)

Following GP1, 3,5-dimethyl-1*H*-pyrazole-4-sulfonyl chloride (**52k**) (0.08 g, 0.41 mmol) and (1*R*,5*S*,7*s*)-7-(pyrazin-2-yloxy)-3-oxa-9-azabicyclo[3.3.1]nonane trifluoroacetate (**59b**) (0.15 g, 0.45 mmol) were used in dry THF. Flash chromatography eluting with DCM/MeOH (96:4). followed by preparative HPLC-MS purification gave the pure title compound (0.02 g, 13%) as a white solid. UPLC-MS: *t*_R_ = 2.58 (generic method); MS (ESI) *m*/*z*: calcd. for C_16_H_22_N_5_O_4_S [M + H]^+^, 380.1; found, 380.1. ^1^H NMR (400 MHz, DMSO-*d*_6_): δ 8.21–8.18 (m, 3H), 5.05–4.98 (m, 1H), 3.91 (d, *J* = 8.6 Hz, 2H), 3.64 (d, *J* = 11.1 Hz, 2H), 3.47 (dd, *J* = 11.1, 2.4 Hz, 2H), 2.44–2.37 (m, 2H), 2.34 (s, 6H), 1.82–1.76 (m, 2H). ^13^C NMR (151 MHz, DMSO-*d*_6_): δ 159.6, 141.2, 136.9, 136.2, 115.3, 70.7, 67.6, 47.8, 31.1, 12.3. HRMS (ESI^+^) *m*/*z*: calcd. for C_16_H_22_N_5_O_4_S, 380.1379 [M + H]^+^; found, 380.1378.

##### (1*R*,5*S*,8*r*)-3-Benzyl-8-(pyrazin-2-yloxy)-3-azabicyclo[3.2.1]octane (**61**)

Following GP3, commercially available *endo*-3-benzyl-3-azabicyclo[3.2.1]octan-8-ol (**60**) (0.1 g, 0.46 mmol) and 2-chloropyrazine (**73a**) (0.053 g, 0.46 mmol) were used. Flash chromatography eluting with DCM/MeOH (95:5) gave the pure title compound (0.08 g, 59%) as a pale yellow oil. UPLC-MS: *t*_R_ = 1.71 (generic method); MS (ESI) *m*/*z*: calcd. for C_18_H_22_N_3_O [M + H]^+^, 296.2; found, 296.1. ^1^H NMR (400 MHz, DMSO-*d*_6_): δ 8.34 (s, 1H), 8.21 (m, 2H), 7.33–7.31 (m, 4H), 7.27–7.21 (m, 1H), 4.93 (t, *J* = 4.7 Hz, 1H), 3.52 (s, 2H, CH_2_), 2.56–2.52 (m, 2H), 2.43 (dd, *J* = 10.8, 3.6 Hz, 2H), 2.34–2.31 (m, 2H), 1.82–1.68 (m, 4H).

#### (1*R*,5*S*,8*r*)-3-((3,5-Dimethyl-1*H*-pyrazol-4-yl)sulfonyl)-8-(pyrazin-2-yloxy)-3-azabicyclo[3.2.1]octane (**27**)

##### Step 1

To a solution of (1*R*,5*S*,8*r*)-3-benzyl-8-pyrazin-2-yloxy-3-azabicyclo[3.2.1]octane (**61**) (0.08 g, 1.0 equiv) in EtOH (5.0 mL) were added ammonium formate (0.171 g, 2.7 mmol, 10 equiv) and 10% Pd/C (*ca.* 10 mg). The resulting mixture was left at room temperature for 3 h. The suspension was then filtered and the residue concentrated to dryness. The crude product was partitioned between EtOAc and brine, dried over Na_2_SO_4_, and concentrated under vacuo to furnish (1*R*,5*S*,8*r*)-8-(pyrazin-2-yloxy)-3-azabicyclo[3.2.1]octane (*quant*.) as a colorless oil, which was used in the next step without any further purification.

##### Step 2

Following GP1, 3,5-dimethyl-1*H*-pyrazole-4-sulfonyl chloride (**52k**) (0.135 g, 0.69 mmol) and (1*R*,5*S*,8*r*)-8-(pyrazin-2-yloxy)-3-azabicyclo[3.2.1]octane (0.156 g, 0.76 mmol) were used in dry DCM. Flash chromatography eluting with DCM/MeOH (95:5) gave the pure title compound (0.12 g, 48%) as a white solid. UPLC-MS: *t*_R_ = 3.28 (generic method); MS (ESI) *m*/*z*: calcd. for C_16_H_22_N_5_O_3_S [M + H]^+^, 364.1; found, 364.3. ^1^H NMR (400 MHz, DMSO-*d*_6_): δ 13.03 (br s, 1H, NH), 8.17 (d, *J* = 1.2 Hz, 1H), 8.23–8.20 (m, 2H), 4.94 (t, *J* = 4.9 Hz, 1H), 3.21 (dd, *J* = 11.1, 3.4 Hz, 2H), 2.93 (d, *J* = 10.8 Hz, 2H), 2.46 (br s, 2H), 2.30 (s, 6H), 1.81–1.76 (m, 2H), 1.64–1.59 (m, 2H). ^13^C NMR (101 MHz, DMSO-*d*_6_): δ 159.4, 141.4, 137.7, 135.6, 112.0, 74.3, 45.5, 35.2, 23.9, 13.8, 11.2. HRMS (ESI^+^) *m*/*z*: calcd. for C_16_H_22_N_5_O_3_S, 364.1443 [M + H]^+^; found, 364.1444.

##### *tert*-Butyl 9-Hydroxy-3-azabicyclo[3.3.1]nonane-3-carboxylate (**62**)

*tert*-Butyl 9-hydroxy-3-azabicyclo[3.3.1]nonane-3-carboxylate (**62**) was prepared using a previously reported procedure. Analytical and spectroscopic data are in agreement with those previously reported.^51^

##### *tert*-Butyl (1*R*,5*S*,9*r*)-9-(pyrazin-2-yloxy)-3-azabicyclo[3.3.1]nonane-3-carboxylate (**63**)

Following GP3, *tert*-butyl (1*R*,5*S*,9*r*)-9-hydroxy-3-azabicyclo[3.3.1]nonane-3-carboxylate (**62**) (0.24 g, 1.0 mmol) and 2-chloropyrazine (**73a**) (0.114 g, 1.0 mmol) were used. Flash chromatography eluting with DCM/MeOH (95:5) gave the pure title compound (0.25 g, 78%), as a 8:2 mixture of *endo/exo* diastereoisomers, as a colorless oil. UPLC-MS: *t*_R_ = 2.82 min (generic method); MS (ESI) *m*/*z*: calcd. for C_17_H_26_N_3_O_3_ (M + H)^+^: 320.2; found, 320.2. ^1^H NMR (400 MHz, DMSO-*d*_6_): δ 8.36–8.35 (m, 1H), 8.33–8.32 (m, 2H), 4.83 (t, *J* = 5.9 Hz, 1H), 2.74–3.66 (m, 2H), 3.26–3.37 (m, 2H), 2.23–2.17 (m, 2H), 1.78–1.67 (m, 2H), 1.64–1.56 (m 1H), 1.52–1.44 (m, 2H), 1.35–1.28 (m, 1H).

##### (1*R*,5*S*,9*r*)-9-Pyrazin-2-yloxy-3-azabicyclo[3.3.1]nonane Trifluoroacetate (**64**)

Following GP4, *tert*-butyl (1*R*,5*S*,9*r*)-9-(pyrazin-2-yloxy)-3-azabicyclo[3.3.1]nonane-3-carboxylate (**63**) was used to give the title compound (*quant*.), a 8:2 mixture of *endo/exo* diastereoisomers, which was used in the next step without any further purification.

##### (1*R*,5*S*,9*r*)-3-((3,5-Dimethyl-1*H*-pyrazol-4-yl)sulfonyl)-9-(pyrazin-2-yloxy)-3-azabicyclo[3.3.1]nonane (**28**)

Following GP1, 3,5-dimethyl-1*H*-pyrazole-4-sulfonyl chloride (**52k**) (0.175 g, 0.54 mmol) and (1*R*,5*S*,9*r*)-9-pyrazin-2-yloxy-3-azabicyclo[3.3.1]nonane trifluoroacetate (**64**) (0.2 g, 0.59 mmol) were used in dry DCM. Flash chromatography eluting with cyclohexane/EtOAc (2:8) gave a ca. 7:3 mixture of *endo/exo* diastereoisomers. The diastereoisomeric mixture was further purified by preparative HPLC-MS to afford the pure title compound (0.018 g, 9%), as *endo*-diastereoisomer, as a white solid. UPLC-MS: *t*_R_ = 3.61 min (generic method); MS (ESI) *m*/*z*: calcd. for C_17_H_24_N_5_O_3_S [M + H]^+^, 378.2; found, 378.3. ^1^H NMR (400 MHz, DMSO-*d*_6_): δ 8.12–8.11 (m, 2H), 8.08 (d, *J* = 1.0 Hz, 1H), 4.90 (t, *J* = 3.4 Hz, 1H), 3.44 (d, *J* = 11.0 Hz, 2H), 2.83 (d, *J* = 12.4 Hz, 2H), 2.24 (s, 6H), 2.21–2.12 (m, 3H), 1.87–1.70 (m, 4H), 1.37–1.31 (m, 1H). ^13^C NMR (101 MHz, DMSO-*d*_6_): δ 159.2, 145.6, 141.2, 137.4, 136.0, 110.9, 73.8, 45.1, 32.1, 30.5, 19.8, 12.7. HRMS (ESI^+^) *m*/*z*: calcd. for C_17_H_24_N_5_O_3_S, 378.1600 [M + H]^+^; found, 378.1597.

#### (1*R*,5*S*,9*s*)-3-((3,5-Dimethyl-1*H*-pyrazol-4-yl)sulfonyl)-9-(pyrazin-2-yloxy)-3-azabicyclo[3.3.1]nonane (**29**)

After preparative HPLC-MS (see synthesis of *endo*-diastereoisomer **28**), the pure title compound was isolated (0.015 g, 7%), as an *exo*-diastereoisomer, as a white solid. UPLC-MS: *t*_R_ = 3.63 min (generic method); MS (ESI) *m*/*z*: calcd. for C_17_H_24_N_5_O_3_S [M + H]^+^, 378.2; found, 378.3. ^1^H NMR (400 MHz, DMSO-*d*_6_): δ 8.27 (d, *J* = 1.4 Hz, 1H), 8.12 (d, *J* = 2.7 Hz, 1H), 8.08 (dd, *J* = 2.7, 1.4 Hz, 1H), 4.87 (t, *J* = 3.6 Hz, 1H), 3.67 (d, *J* = 10.0 Hz, 2H), 2.70 (d, *J* = 12.5 Hz, 2H), 2.27 (s, 6H), 2.25–2.12 (m, 3H), 1.92–1.85 (m,2H), 1.57 (dd, *J* = 13.7, 5.8 Hz, 2H). 1.41–1.35 (m, 1H). ^13^C NMR (101 MHz, DMSO-*d*_6_): δ 159.3, 141.2, 137.4, 136.2, 110.5, 74.3, 50.7, 31.1, 24.4, 19.7, 12.7. HRMS (ESI^+^) *m*/*z*: calcd. for C_17_H_24_N_5_O_3_S, 378.1600 [M + H]^+^; found, 378.1598.

##### *tert*-Butyl (1*R*,3*r*,5*S*)-3-(Pyridin-2-yloxy)-8-azabicyclo[3.2.1]octane-8-carboxylate (**76b**)

Following GP3, *tert*-butyl (1*R*,3*r*,5*S*)-3-hydroxy-8-azabicyclo[3.2.1]octane-8-carboxylate (**65a**) (0.2 g, 0.88 mmol) and 2-chloropyridine (**73b**) (0.1 g, 0.88 mmol) were used in dry THF. Flash chromatography eluting with cyclohexane/EtOAc (4:6) gave the pure title compound (0.04 g, 15%) as a colorless oil. UPLC-MS: *t*_R_ = 1.57 min (generic method); MS (ESI) *m*/*z*: calcd. for C_17_H_25_N_2_O_3_ [M + H]^+^, 305.2; found, 305.2. ^1^H NMR (400 MHz, DMSO-*d*_6_): δ 8.15 (ddd, *J* = 5.0, 2.1, 0.8 Hz, 1H), 7.71 (ddd, *J* = 8.3, 7.1, 2.0 Hz, 1H), 6.95 (ddd, *J* = 7.1, 5.0, 0.9 Hz, 1H), 6.79 (dt, *J* = 8.3, 0.9 Hz, 1H), 5.31 (t, *J* = 5.0 Hz, 1H), 4.15–4.03 (m, 2H), 2.13–1.98 (m, 4H), 1.93–1.77 (m, 4H), 1.42 (s, 9H).

##### (1*R*,3*r*,5*S*)-3-(Pyrid-2-yloxy)-8-azabicyclo[3.2.1]octane Trifluoroacetate (**80b**)

Following GP4, *tert*-butyl (1*R*,3*r*,5*S*)-3-(pyridin-2-yloxy)-8-azabicyclo[3.2.1]octane-8-carboxylate (**76b**) was used to give the title compound (*quant*.), which was used in the next step without any purification.

##### (1*R*,3*r*,5*S*)-8-((3,5-Dimethyl-1*H*-pyrazol-4-yl)sulfonyl)-3-(pyridin-2-yloxy)-8-azabicyclo[3.2.1]octane (**30**)

Following GP1, 3,5-dimethyl-1*H*-pyrazole-4-sulfonyl chloride (**52k**) (0.02 g, 0.1 mmol) and (1*R*,3*r*,5*S*)-3-(2-pyridyloxy)-8-azabicyclo[3.2.1]octane trifluoroacetate (**80b**) (0.035 g, 0.11 mmol) were used in dry THF. Flash chromatography eluting with cyclohexane/EtOAc (2:8) gave the pure title compound (0.03 g, 83%) as a white solid. UPLC-MS: *t*_R_ = 3.79 min (generic method); MS (ESI) *m*/*z*: calcd. for C_17_H_23_N_4_O_3_S [M + H]^+^, 363.1; found, 363.0. ^1^H NMR (400 MHz, DMSO-*d*_6_): δ 12.99 (br s, 1H, NH), 8.14 (dd, *J* = 5.0, 2.0 Hz, 1H), 7.69 (ddd, *J* = 8.7, 7.1, 2.1 Hz, 1H), 6.95 (dd, *J* = 7.1, 5.0 Hz, 1H), 6.75 (d, *J* = 8.7 Hz, 1H), 5.28 (t, *J* = 5.0 Hz, 1H), 4.12–4.10 (m, 2H), 2.37 (s, 3H), 2.28 (s, 3H), 2.10–1.93 (m, 6H), 1.64–1.61 (m, 2H). ^13^C NMR (101 MHz, DMSO-*d*_6_): δ 159.4, 141.4, 137.7, 135.6, 112.0, 74.4, 45.5, 35.2, 23.9, 13.5, 11.1. HRMS (ESI^+^) *m*/*z*: calcd. for C_17_H_23_N_4_O_3_S, 363.1491 [M + H]^+^; found, 363.1496.

##### *tert*-Butyl (1*R*,3*r*,5*S*)-3-Phenoxy-8-azabicyclo[3.2.1]octane-8-carboxylate (**67a**)

Following GP2, phenol (**66a**) (0.12 g, 1.28 mmol) and *tert*-butyl (1*R*,3*s*,5*S*)-3-hydroxybicyclo[3.2.1]octane-8-carboxylate (**65b**) were used. Flash chromatography eluting with EtOAc gave the pure title compound (0.13 g, 34%) as a colorless oil. UPLC-MS: *t*_R_ = 3.24 (generic method); MS (ESI) *m*/*z*: calcd. for C_18_H_26_NO_3_ [M + H]^+^, 304.2; found, 304.1. ^1^H NMR (400 MHz, DMSO-*d*_6_): δ 7.33–7.24 (m, 2H), 6.96–6.85 (m, 3H), 4.72 (t, *J* = 4.9 Hz, 1H), 4.12–3.98 (m, 2H), 2.13–1.95 (m, 4H), 1.94–1.79 (m, 3H), 1.42 (s, 9H), 1.40–1.38 (m, 1H).

##### (1*R*,3*r*,5*S*)-3-Phenoxy-8-azabicyclo[3.2.1]octane Trifluoroacetate (**69a**)

Following GP4, *tert*-butyl (1*R*,3*r*,5*S*)-3-phenoxy-8-azabicyclo[3.2.1]octane-8-carboxylate (**67a**) was used to give the title compound (*quant*.), which was used in the next step without any purification.

##### (1*R*,3*r*,5*S*)-8-((3,5-Dimethyl-1*H*-pyrazol-4-yl)sulfonyl)-3-phenoxy-8-azabicyclo[3.2.1]octane (**31**)

Following GP1, 3,5-dimethyl-1*H*-pyrazole-4-sulfonyl chloride (**52k**) (0.04 g, 0.2 mmol) and (1*R*,3*r*,5*S*)-3-phenoxy-8-azabicyclo[3.2.1]octane trifluoroacetate (**69a**) (0.07 g, 0.22 mmol) were used in dry THF. Flash chromatography eluting with DCM/MeOH (98:2) gave the pure title compound (0.02 g, 29%) as a white solid. UPLC-MS: *t*_R_ = 4.39 (generic method); MS (ESI) *m*/*z*: calcd. for C_18_H_24_N_3_O_3_S [M + H]^+^, 362.1; found, 362.2. ^1^H NMR (400 MHz, DMSO-*d*_6_): δ 12.99 (br s, 1H, *NH*), 7.30–7.26 (m, 2H), 6.91 (tt, *J* = 7.4, 1.1 Hz, 1H), 6.87–6.85 (m, 2H), 4.67 (t, *J* = 4.8 Hz, 1H), 4.10–4.08 (m, 2H), 2.37 (s, 3H), 2.27 (s, 3H), 2.07–1.93 (m, 6H), 1.63–1.60 (m, 2H). ^13^C NMR (101 MHz, DMSO-*d*_6_): δ 157.1, 147.8, 142.7, 130.1, 121.1, 115.9, 114.9, 69.2, 55.4, 36.8, 28.4, 13.5, 10.9. HRMS (ESI^+^) *m*/*z*: calcd. for C_18_H_24_N_3_O_3_S, 362.1538 [M + H]^+^; found, 362.1539.

##### *tert*-Butyl (1*R*,3*s*,5*S*)-3-Phenoxy-8-azabicyclo[3.2.1]octane-8-carboxylate (**68a**)

Following GP2, phenol (**66a**) (0.07 g, 0.79 mmol) and *tert*-butyl (1*R*,3*r*,5*S*)-3-hydroxybicyclo[3.2.1]octane-8-carboxylate (**65a**) (0.19 g, 0.83 mmol) were used in dry THF. Flash chromatography eluting with cyclohexane/EtOAc (2:8) gave the pure title compound (0.07 g, 29%) as a colorless oil. UPLC-MS: *t*_R_ = 1.86 min (generic method); MS (ESI) *m*/*z*: calcd. for C_18_H_26_NO_3_ [M + H]^+^, 304.2; found, 304.1. ^1^H NMR (400 MHz, DMSO-*d*_6_): δ 7.30–7.23 (m, 2H), 7.01–6.95 (m, 2H), 6.92 (td, *J* = 7.3, 1.1 Hz, 1H), 4.85–4.74 (m, 1H), 4.19–4.10 (m, 2H), 2.15–2.03 (m, 2H), 1.95–1.73 (m, 4H), 1.58–1.46 (m, 2H), 1.42 (s, 9H).

##### (1*R*,3*s*,5*S*)-3-Phenoxy-8-azabicyclo[3.2.1]octane Trifluoroacetate (**70a**)

Following GP4, *tert*-butyl (1*R*,3*s*,5*S*)-3-phenoxy-8-azabicyclo[3.2.1]octane-8-carboxylate (**68a**) was used to give the title compound (*quant*.), which was used in the next step without any purification.

##### (1*R*,3*s*,5*S*)-8-((3,5-Dimethyl-1*H*-pyrazol-4-yl)sulfonyl)-3-phenoxy-8-azabicyclo[3.2.1]octane (**32**)

Following GP1, 3,5-dimethyl-1*H*-pyrazole-4-sulfonyl chloride (**52k**) (0.04 g, 0.21 mmol) and (1*R*,3*s*,5*S*)-3-phenoxy-8-azabicyclo[3.2.1]octane trifluoroacetate (**70a**) (0.073 g, 0.23 mmol) were used in dry THF. Flash chromatography eluting with DCM/MeOH (98:2) gave the pure title compound (0.05 g, 66%) as a white solid. UPLC-MS: *t*_R_ = 4.10 min (generic method); MS (ESI) *m*/*z*: calcd. for C_18_H_24_N_3_O_3_S [M + H]^+^, 362.2; found, 362.0. ^1^H NMR (400 MHz, DMSO-*d*_6_): δ 12.99 (br s, 1H, NH), 7.26 (dd, *J* = 8.6, 7.2 Hz, 2H), 6.97 (d, *J* = 8.6 Hz, 2H), 6.92 (t, *J* = 7.2 Hz, 1H), 4.73–4.65 (m, 1H), 4.18–4.26 (m, 2H), 2.37 (s, 3H), 2.30 (s, 3H), 2.19–2.14 (m, 2H), 1.85–1.79 (m, 2H), 1.68–1.56 (m, 4H). ^13^C NMR (101 MHz, DMSO-*d*_6_): δ 157.5, 142.76, 129.9, 121.2, 116.2, 114.9, 69.3, 55.8, 38.4, 28.5, 13.5, 10.9. HRMS (ESI^+^) *m*/*z*: calcd. for C_18_H_24_N_3_O_3_S, 362.1538 [M + H]^+^; found, 362.1536.

##### *tert*-Butyl (1*R*,3*r*,5*S*)-3-(*p*-Tolyloxy)-8-azabicyclo[3.2.1]octane-8-carboxylate (**67b**)

Following GP2, 4-methylphenol (**66b**) (0.08 mL, 0.73 mmol) and *tert*-butyl (1*R*,3*s*,5*S*)-3-hydroxybicyclo[3.2.1]octane-8-carboxylate (**65b**) (0.17 g, 0.77 mmol) were used in dry THF. Flash chromatography eluting with EtOAc gave the pure title compound (0.12 g, 52%) as a colorless oil. UPLC-MS: *t*_R_ = 2.66 min (generic method); MS (ESI) *m*/*z*: calcd. for C_19_H_28_NO_3_ [M + H]^+^, 318.2; found, 318.1. ^1^H NMR (400 MHz, DMSO-*d*_6_): δ 7.06 (d, *J* = 8.5 Hz, 2H), 6.78 (d, *J* = 8.5 Hz, 2H), 4.61 (t, *J* = 4.9 Hz, 1H), 4.04–4.01 (m, 2H), 2.26 (s, 3H), 2.06–1.97 (m, 4H), 1.83–1.80 (m, 4H), 1.42 (s, 9H).

##### (1*R*,3*r*,5*S*)-3-(*p*-Tolyloxy)-8-azabicyclo[3.2.1]octane Trifluoroacetate (**69b**)

Following GP4, *tert*-butyl (1*R*,3*r*,5*S*)-3-(*p*-tolyloxy)-8-azabicyclo[3.2.1]octane-8-carboxylate (**67b**) was used to give the title compound (*quant*.), which was used in the next step without any purification.

##### (1*R*,3*r*,5*S*)-8-((3,5-Dimethyl-1*H*-pyrazol-4-yl)sulfonyl)-3-(*p*-tolyloxy)-8-azabicyclo[3.2.1]octane (**33**)

Following GP1, 3,5-dimethyl-1*H*-pyrazole-4-sulfonyl chloride (**52k**) (0.1 g, 0.52 mmol) and (1*R*,3*r*,5*S*)-3-(*p*-tolyloxy)-8-azabicyclo[3.2.1]octane trifluoroacetate (**69b**) (0.19 g, 0.57 mmol) were used in dry THF. Flash chromatography eluting with DCM/MeOH (95:5) gave the pure title compound (0.03 g, 15%) as a white solid. UPLC-MS: *t*_R_ = 4.73 min (generic method); MS (ESI) *m*/*z*: calcd. for C_19_H_26_N_3_O_3_S [M + H]^+^, 376.2; found, 376.1. ^1^H NMR (400 MHz, DMSO-*d*_6_): δ 7.08 (d, *J* = 8.2 Hz, 2H), 6.76 (d, *J* = 8.2 Hz, 2H), 4.63 (t, *J* = 4.7 Hz, 1H), 4.10–4.08 (m, 2H), 2.37 (s, 3H), 2.27 (s, 3H), 2.22 (s, 3H), 2.05–1.93 (m, 6H), 1.63–1.60 (m, 2H). ^13^C NMR (151 MHz, DMSO-*d*_6_): δ 154.9, 147.8, 142.7, 130.5, 129.7, 115.9, 114.9, 69.3, 55.4, 36.8, 28.4, 20.5, 13.5, 10.9. HRMS (ESI^+^) *m*/*z*: calcd. for C_19_H_26_N_3_O_3_S, 376.1695 [M + H]^+^; found, 376.1696.

##### *tert*-Butyl (1*R*,3*s*,5*S*)-3-(*p*-Tolyloxy)-8-azabicyclo[3.2.1]octane-8-carboxylate (**68b**)

Following GP2, 4-methylphenol (**66b**) (0.11 mL, 1.05 mmol) and *tert*-butyl (1*R*,3*r*,5*S*)-3-hydroxybicyclo[3.2.1]octane-8-carboxylate (**65a**) (0.25 g, 1.1 mmol) were used in dry THF. Flash chromatography eluting with EtOAc gave the pure title compound (0.15 g, 45%) as a colorless oil. UPLC-MS: *t*_R_ = 1.90 min (generic method); MS (ESI) *m*/*z*: calcd. for C_19_H_28_NO_3_ [M + H]^+^, 318.2; found, 318.1. ^1^H NMR (400 MHz, DMSO-*d*_6_): δ 7.00 (d, *J* = 8.3 Hz, 2H), 6.81 (d, *J* = 8.3 Hz, 2H), 4.61–4.52 (m, 1H), 4.18–4.11 (m, 2H), 2.21 (s, 3H), 2.15–1.73 (m, 4H), 1.64–1.49 (m, 4H), 1.36 (s, 9H).

##### (1*R*,3*s*,5*S*)-3-(*p*-Tolyloxy)-8-azabicyclo[3.2.1]octane Trifluoroacetate (**70b**)

Following GP3, *tert*-butyl (1*R*,3*s*,5*S*)-3-(*p*-tolyloxy)-8-azabicyclo[3.2.1]octane-8-carboxylate (**68b**) was used to give the title compound (*quant*.), which was used in the next step without any purification.

##### (1*R*,3*s*,5*S*)-8-((3,5-Dimethyl-1*H*-pyrazol-4-yl)sulfonyl)-3-(*p*-tolyloxy)-8-azabicyclo[3.2.1]octane (**34**)

Following GP1, 3,5-dimethyl-1*H*-pyrazole-4-sulfonyl chloride (**52k**) (0.07 g, 0.36 mmol) and (1*R*,3*s*,5*S*)-3-(4-methylphenoxy)-8-azabicyclo[3.2.1]octane trifluoroacetate (**72b**) (0.13 g, 0.4 mmol) were used in dry THF. Flash chromatography eluting with DCM/MeOH (95:5) gave the pure title compound (0.06 g, 44%) as a white solid. UPLC-MS: *t*_R_ = 4.45 min (generic method); MS (ESI) *m*/*z*: calcd. for C_19_H_26_N_3_O_3_S [M + H]^+^, 376.2; found, 376.0. ^1^H NMR (400 MHz, DMSO-*d*_6_): δ 7.04 (d, *J* = 8.3 Hz, 2H), 6.83 (d, *J* = 8.3 Hz, 2H), 4.63–4.55 (m, 1H), 4.15–4.12 (m, 2H), 2.31 (s, 6H), 2.20 (s, 3H), 2.15-2-10 (m, 2H), 1.79–1.74 (m, 2H), 1.64–1.61 (m, 2H), 1.57–1.51 (m, 2H). ^13^C NMR (101 MHz, DMSO-*d*_6_): δ 155.3, 130.3, 130.1, 116.3, 69.5, 55.8, 38.4, 28.5, 21.5, 20.5, 12.2. HRMS (ESI^+^) *m*/*z*: calcd. for C_19_H_26_N_3_O_3_S, 376.1695 [M + H]^+^; found, 376.1690.

##### *tert*-Butyl (1*R*,3*r*,5*S*)-3-(*m*-tolyloxy)-8-azabicyclo[3.2.1]octane-8-carboxylate (**67c**)

Following GP2, 3-methylphenol (**66c**) (0.08 mL, 0.73 mmol) and *tert*-butyl (1*R*,3*s*,5*S*)-3-hydroxybicyclo[3.2.1]octane-8-carboxylate (**65b**) (0.18 g, 0.77 mmol) were used in dry THF. Flash chromatography eluting with EtOAc gave the pure title compound (0.18 g, 79%) as a colorless oil. UPLC-MS: *t*_R_ = 2.69 min (generic method); MS (ESI) *m*/*z*: calcd. for C_19_H_28_NO_3_ [M + H]^+^, 318.2; found, 318.1. ^1^H NMR (400 MHz, DMSO-*d*_6_): δ 7.16 (t, *J* = 7.8 Hz, 1H), 6.77–6.70 (m, 2H), 6.67 (dd, *J* = 8.1, 2.5 Hz, 1H), 4.69 (t, *J* = 4.9 Hz, 1H), 4.09–4.04 (m, 2H), 2.27 (s, 3H), 2.08–1.97 (m, 4H), 1.83 (d, *J* = 17.4 Hz, 4H), 1.42 (s, 9H).

##### (1*R*,3*r*,5*S*)-3-(*m*-Tolyloxy)-8-azabicyclo[3.2.1]octane Trifluoroacetate (**69c**)

Following GP4, *tert*-butyl (1*R*,3*r*,5*S*)-3-(*m*-tolyloxy)-8-azabicyclo[3.2.1]octane-8-carboxylate (**67c**) was used to give the title compound (*quant*.), which was used in the next step without any purification.

##### (1*R*,3*r*,5*S*)-8-((3,5-Dimethyl-1*H*-pyrazol-4-yl)sulfonyl)-3-(*m*-tolyloxy)-8-azabicyclo[3.2.1]octane (**35**)

Following GP1, 3,5-dimethyl-1*H*-pyrazole-4-sulfonyl chloride (**52k**) (0.1 g, 0.51 mmol) and (1*R*,3*r*,5*S*)-3-(*m*-tolyloxy)-8-azabicyclo[3.2.1]octane trifluoroacetate (**69c**) (0.18 g, 0.54 mmol) were used in dry THF. Flash chromatography eluting with DCM/MeOH (95:5), followed by preparative HPLC-MS purification gave the pure title compound (0.02 g, 10%) as a white solid. UPLC-MS: *t*_R_ = 4.74 min (generic method); MS (ESI) *m*/*z*: calcd. for C_19_H_26_N_3_O_3_S [M + H]^+^, 376.2; found, 376.4. ^1^H NMR (400 MHz, DMSO-*d*_6_): δ 7.15 (t, *J* = 7.8 Hz, 1H), 6.73 (d, *J* = 7.5 Hz, 1H), 6.69–6.64 (m, 2H), 4.65 (t, *J* = 4.7 Hz, 1H), 4.09–4.97 (m, 2H), 2.32 (s, 6H), 2.25 (s, 3H), 2.06–1.93 (m, 6H), 1.63–1.59 (m, 2H). ^13^C NMR (101 MHz, DMSO-*d*_6_): δ 157.1, 139.7, 129.8, 121.8, 116.8, 114.9, 112.8, 69.2, 55.4, 36.8, 28.4, 21.5. HRMS (ESI^+^) *m*/*z*: calcd. for C_19_H_26_N_3_O_3_S, 376.1695 [M + H]^+^; found, 376.1693.

##### *tert*-Butyl (1*R*,3*r*,5*S*)-3-(*o*-tolyloxy)-8-azabicyclo[3.2.1]octane-8-carboxylate (**67d**)

Following GP2, 2-methylphenol (**66d**) (0.08 g, 0.73 mmol) and *tert*-butyl (1*R*,3*s*,5*S*)-3-hydroxybicyclo[3.2.1]octane-8-carboxylate (**65b**) (0.17 g, 0.77 mmol) were used in dry THF. Flash chromatography eluting with EtOAc gave the pure title compound (0.18 g, 66%) as a colorless oil. UPLC-MS: *t*_R_ = 2.65 min (generic method); MS (ESI) *m*/*z*: calcd. for C_19_H_28_NO_3_ [M + H]^+^, 318.2; found, 318.1. ^1^H NMR (400 MHz, DMSO-*d*_6_): δ 7.15 (t, *J* = 7.8 Hz, 1H), 6.77–6.66 (m, 2H), 6.63 (dd, *J* = 8.1, 2.4 Hz, 1H), 4.70 (t, *J* = 4.9 Hz, 1H), 4.09–4.00 (m, 2H), 2.27 (s, 3H), 2.08–1.95 (m, 4H), 1.80 (d, *J* = 17.4 Hz, 4H), 1.42 (s, 9H).

##### (1*R*,3*r*,5*S*)-3-(*o*-Tolyloxy)-8-azabicyclo[3.2.1]octane Trifluoroacetate (**69d**)

Following GP4, *tert*-butyl (1*R*,3*r*,5*S*)-3-(*o*-tolyloxy)-8-azabicyclo[3.2.1]octane-8-carboxylate (**67d**) was used to give the title compound (*quant*.), which was used in the next step without any purification.

##### (1*R*,3*r*,5*S*)-8-((3,5-Dimethyl-1*H*-pyrazol-4-yl)sulfonyl)-3-(*o*-tolyloxy)-8-azabicyclo[3.2.1]octane (**36**)

Following GP1, 3,5-dimethyl-1*H*-pyrazole-4-sulfonyl chloride (**52k**) (0.1 g, 0.51 mmol) and (1*R*,3*r*,5*S*)-3-(*o*-tolyloxy)-8-azabicyclo[3.2.1]octane trifluoroacetate (**69d**) (0.19 g, 0.56 mmol) were used in dry THF. Flash chromatography eluting with DCM/MeOH (95:5) gave the pure title compound (0.03 g, 15%) as a white solid. UPLC-MS: *t*_R_ = 4.83 min (generic method); MS (ESI) *m*/*z*: calcd. for C_19_H_26_N_3_O_3_S [M + H]^+^, 376.2; found, 376.0. ^1^H NMR (400 MHz, DMSO-*d*_6_): δ 7.13–7.09 (m, 2H), 6.80–6.76 (m, 2H), 4.64 (t, *J* = 4.9 Hz, 1H), 4.12–4.09 (m, 2H), 2.32 (s, 6H), 2.12 (s, 3H), 2.10–1.95 (m, 6H), 1.64–1.60 (m, 2H). ^13^C NMR (101 MHz, DMSO-*d*_6_): δ 155.2, 131.3, 127.3, 126.4, 120.3, 114.9, 111.6, 68.5, 55.4, 37.1, 28.4, 16.6, 12.2. HRMS (ESI^+^) *m*/*z*: calcd. for C_19_H_26_N_3_O_3_S, 376.1695 [M + H]^+^; found, 376.1695.

##### *tert*-Butyl (1*R*,3*r*,5*S*)-3-(4-ethylphenoxy)-8-azabicyclo[3.2.1]octane-8-carboxylate (**67e**)

Following GP2, 3-ethylphenol (**66e**) (0.09 g, 0.73 mmol) and *tert*-butyl (1*R*,3*s*,5*S*)-3-hydroxybicyclo[3.2.1]octane-8-carboxylate (**65b**) (0.18 g, 0.77 mmol) were used in dry THF. Flash chromatography eluting with EtOAc gave the pure title compound (0.12 g, 50%) as a colorless oil. UPLC-MS: *t*_R_ = 2.72 min (generic method); MS (ESI) *m*/*z*: calcd. for C_20_H_30_NO_3_ [M + H]^+^, 332.2; found, 332.1. ^1^H NMR (400 MHz, DMSO-*d*_6_): δ 7.15–7.08 (m, 2H), 6.84–6.77 (m, 2H), 4.67 (t, J = 4.8 Hz, 1H), 4.09–4.03 (m, 2H), 2.58–2.51 (m, 2H), 2.10–1.94 (m, 4H), 1.92–1.80 (m, 4H), 1.42 (s, 9H), 1.15 (t, *J* = 7.6 Hz, 3H).

##### (1*R*,3*r*,5*S*)-3-(4-Ethylphenoxy)-8-azabicyclo[3.2.1]octane Trifluoroacetate (**69e**)

Following GP4, *tert*-butyl (1*R*,3*r*,5*S*)-3-(4-ethylphenoxy)-8-azabicyclo[3.2.1]octane-8-carboxylate (**67e**) was used to give the title compound (*quant*.), which was used in the next step without any purification.

##### (1*R*,3*r*,5*S*)-8-((3,5-Dimethyl-1*H*-pyrazol-4-yl)sulfonyl)-3-(4-ethylphenoxy)-8-azabicyclo[3.2.1]octane (**37**)

Following GP1, 3,5-dimethyl-1*H*-pyrazole-4-sulfonyl chloride (**52k**) (0.07 g, 0.36 mmol) and (1*R*,3*r*,5*S*)-3-(4-ethylphenoxy)-8-azabicyclo[3.2.1]octane trifluoroacetate (**69e**) (0.14 g, 0.4 mmol) were used in dry THF. Flash chromatography eluting with cyclohexane/EtOAc (4:6) gave the pure title compound (0.08 g, 57%) as a white solid. UPLC-MS: *t*_R_ = 5.09 min (generic method); MS (ESI) *m*/*z*: calcd. for C_20_H_28_N_3_O_3_S [M + H]^+^, 390.2; found, 390.2. ^1^H NMR (400 MHz, DMSO-*d*_6_): δ 12.97 (br s, 1H, *N*H), 7.17–7.06 (m, 2H), 6.84–6.72 (m, 2H), 4.63 (t, *J* = 4.1 Hz, 1H), 4.15–4.04 (m, 2H), 2.57–2.52 (m, 2H), 2.37 (s, 3H), 2.28 (s, 3H), 2.09–1.92 (m, 6H), 1.67–1.58 (m, 2H), 1.14 (t, *J* = 7.6 Hz, 3H). ^13^C NMR (101 MHz, DMSO-*d*_6_): δ 155.1, 136.3, 129.3, 115.9, 114.9, 69.3, 55.5, 36.8, 28.4, 27.7, 26.8, 16.3, 13.5, 10.9. HRMS (ESI^+^) *m*/*z*: calcd. for C_20_H_28_N_3_O_3_S, 390.1851 [M + H]^+^; found, 390.1865.

##### *tert*-Butyl (1*R*,3*r*,5*S*)-3-(4-isopropylphenoxy)-8-azabicyclo[3.2.1]octane-8-carboxylate (**67f**)

Following GP2, 4-isopropylphenol (**66f**) (0.1 g, 0.73 mmol) and *tert*-butyl (1*R*,3*s*,5*S*)-3-hydroxybicyclo[3.2.1]octane-8-carboxylate (**65b**) (0.18 g, 0.77 mmol) were used in dry THF. Flash chromatography eluting with EtOAc gave the pure title compound (0.14 g, 56%) as a colorless oil. UPLC-MS: *t*_R_ = 2.93 min (generic method); MS (ESI) *m*/*z*: calcd for C_21_H_32_NO_3_ [M + H]^+^, 346.2; found, 346.1. ^1^H NMR (400 MHz, DMSO-*d*_6_): δ 7.18–7.11 (m, 2H), 6.83–6.77 (m, 2H), 4.67 (t, *J* = 4.8 Hz, 1H), 4.10–4.01 (m, 2H), 2.89–2.76 (m, 1H), 2.10–1.94 (m, 4H), 1.93–1.79 (m, 4H), 1.42 (s, 9H), 1.18 (d, J = 6.9 Hz, 6H).

##### (1*R*,3*r*,5*S*)-3-(4-Isopropylphenoxy)-8-azabicyclo[3.2.1]octane Trifluoroacetate (**69f**)

Following GP4, *tert*-butyl (1*R*,3*r*,5*S*)-3-(4-isopropylphenoxy)-8-azabicyclo[3.2.1]octane-8-carboxylate (**67f**) was used to give the title compound (*quant*.), which was used in the next step without any purification.

##### (1*R*,3*r*,5*S*)-8-((3,5-Dimethyl-1*H*-pyrazol-4-yl)sulfonyl)-3-(4-isopropylphenoxy)-8-azabicyclo[3.2.1]octane (**38**)

Following GP1, 3,5-dimethyl-1*H*-pyrazole-4-sulfonyl chloride (**52k**) (0.07 g, 0.36 mmol) and (1*R*,3*r*,5*S*)-3-(4-isopropylphenoxy)-8-azabicyclo[3.2.1]octane trifluoroacetate (**69f**) (0.14 g, 0.4 mmol) were used in dry THF. Flash chromatography eluting with cyclohexane/EtOAc (4:6) gave the pure title compound (0.09 g, 62%) as a white solid. UPLC-MS: *t*_R_ = 5.43 min (generic method); MS (ESI) *m*/*z*: calcd. for C_21_H_30_N_3_O_3_S [M + H]^+^, 404.2; found, 404.2. ^1^H NMR (400 MHz, DMSO-*d*_6_): δ 12.98 (br s, 1H, *NH*), 7.14 (d, *J* = 8.6 Hz, 2H), 6.78 (d, *J* = 8.6 Hz, 2H), 4.63 (t, *J* = 4.7 Hz, 1H), 4.10-4-08 (m, 2H), 2.87–2.76 (m, 1H), 2.32 (s, 6H), 2.06–1.94 (m, 6H), 1.63–1.0 (m, 2H), 1.17 (d, *J* = 6.9 Hz, 6H). ^13^C NMR (101 MHz, DMSO-*d*_6_): δ 155.2, 140.9, 127.7, 115.8, 114.9, 69.3, 55.4, 36.8, 33.1, 28.4, 24.5. HRMS (ESI^+^) *m*/*z*: calcd. for C_21_H_30_N_3_O_3_S, 404.2008 [M + H]^+^; found, 404.2017.

##### *tert*-Butyl (1R,3r,5S)-3-(4-butylphenoxy)-8-azabicyclo[3.2.1]octane-8-carboxylate (**67g**)

Following GP2, 4-butylphenol (**66g**) (0.11 mL, 0.73 mmol) and *tert*-butyl (1*R*,3*s*,5*S*)-3-hydroxybicyclo[3.2.1]octane-8-carboxylate (**65b**) (0.18 g, 0.77 mmol) were used in dry THF. Flash chromatography eluting with EtOAc gave the pure title compound (0.21 g, 78%) as a colorless oil. UPLC-MS: *t*_R_ = 2.85 min (apolar method); MS (ESI) *m*/*z*: calcd. for C_22_H_34_NO_3_ [M + H]^+^, 360.2; found, 360.1. ^1^H NMR (400 MHz, DMSO-*d*_6_): δ 7.13–7.06 (m, 2H), 6.82–6.76 (m, 2H), 4.66 (t, *J* = 4.9 Hz, 1H), 4.10–4.04 (m, 2H), 2.08–1.96 (m, 4H), 1.92–1.77 (m, 4H), 1.51 (tt, *J* = 7.9, 6.4 Hz, 2H), 1.42 (s, 9H), 1.34–1.23 (m, 3H), 1.18 (t, *J* = 7.1 Hz, 1H), 0.89 (td, *J* = 7.3, 3.2 Hz, 3H).

##### (1*R*,3*s*,5*S*)-3-(4-Butylphenoxy)-8-azabicyclo[3.2.1]octane Trifluoroacetate (**69g**)

Following GP4, *tert*-butyl (1*R*,3*r*,5*S*)-3-(4-butylphenoxy)-8-azabicyclo[3.2.1]octane-8-carboxylate (**67g**) was used to give the title compound (*quant*.), which was used in the next step without any purification.

##### (1*R*,3*r*,5*S*)-3-(4-Butylphenoxy)-8-((3,5-dimethyl-1*H*-pyrazol-4-yl)sulfonyl)-8-azabicyclo[3.2.1]octane (**39**)

Following GP1, 3,5-dimethyl-1*H*-pyrazole-4-sulfonyl chloride (**52k**) (0.1 g, 0.51 mmol) and (1*R*,3*r*,5*S*)-3-(4-butylphenoxy)-8-azabicyclo[3.2.1]octane trifluoroacetate (**69g**) (0.21 g, 0.57 mmol) were used in dry THF. Flash chromatography eluting with DCM/MeOH (95:5) gave the pure title compound (0.158 g, 74%) as a white solid. UPLC-MS: *t*_R_ = 3.57 min (apolar method); MS (ESI) *m*/*z*: calcd. for C_22_H_32_N_3_O_3_S [M + H]^+^, 418.2; found, 418.2. ^1^H NMR (400 MHz, DMSO-*d*_6_): δ 7.08 (d, *J* = 8.5 Hz, 2H), 6.76 (d, *J* = 8.5 Hz, 2H), 4.61 (t, *J* = 4.7 Hz, 1H), 4.09–4.07 (m, 2H), 2.47 (d, *J* = 7.7 Hz, 2H), 2.31 (s, 6H), 2.04–1.92 (m, 6H), 1.62–1.58 (m, 2H), 1.53–1.46 (m, 2H), 1.33–1.23 (m, 2H), 0.88 (t, *J* = 7.3 Hz, 3H). ^13^C NMR (101 MHz, DMSO-*d*_6_): δ 155.1, 134. 8, 129. 8, 115.8, 114. 9, 69.2, 55. 5, 36.8, 34.4, 33.8, 28.4, 22.2, 14.2. HRMS (ESI^+^) *m*/*z*: calcd. for C_22_H_32_N_3_O_3_S, 418.2164 [M + H]^+^; found, 418.2166.

##### *tert*-Butyl (1*R*,3*s*,5*S*)-3-(4-Butylphenoxy)-8-azabicyclo[3.2.1]octane-8-carboxylate (**68g**)

Following GP2, 4-butylphenol (**66g**) (0.11 mL, 0.73 mmol) and *tert*-butyl (1*R*,3*r*,5*S*)-3-hydroxybicyclo[3.2.1]octane-8-carboxylate (**65a**) (0.18 g, 0.77 mmol) were used in dry THF. Flash chromatography eluting with EtOAc gave the pure title compound (0.15 g, 57%) as a colorless oil. UPLC-MS: *t*_R_ = 2.80 min (generic method); MS (ESI) *m*/*z*: calcd. for C_22_H_34_NO_3_ [M + H]^+^, 360.2; found, 360.1. ^1^H NMR (400 MHz, DMSO-*d*_6_): δ 7.10–7.03 (m, 2H), 6.91–6.84 (m, 2H), 4.77–4.67 (m, 1H), 4.18–4.02 (m, 2H), 2.51–2.46 (m, 2H), 2.13–1.98 (m, 2H), 1.95–1.70 (m, 4H), 1.60–1.44 (m, 4H), 1.42 (s, 9H), 1.28 (h, *J* = 7.3 Hz, 2H), 0.88 (td, *J* = 7.3, 1.9 Hz, 3H).

##### (1*R*,3*s*,5*S*)-3-(4-Butylphenoxy)-8-azabicyclo[3.2.1]octane Trifluoroacetate (**70g**)

Following GP4, *tert*-butyl (1*R*,3*s*,5*S*)-3-(4-butylphenoxy)-8-azabicyclo[3.2.1]octane-8-carboxylate (**68g**) was used to give the title compound (*quant*.), which was used in the next step without any purification.

##### (1*R*,3*s*,5*S*)-3-(4-Butylphenoxy)-8-((3,5-dimethyl-1*H*-pyrazol-4-yl)sulfonyl)-8-azabicyclo[3.2.1]octane (**40**)

Following GP1, 3,5-dimethyl-1*H*-pyrazole-4-sulfonyl chloride (**52k**) (0.07 g, 0.36 mmol) and (1*R*,3*s*,5*S*)-3-(4-butylphenoxy)-8-azabicyclo[3.2.1]octane trifluoroacetate (**70g**) (0.15 g, 0.4 mmol) were used in dry THF. Flash chromatography eluting with DCM/MeOH (95:5) gave the pure title compound (0.06 g, 40%) as a white solid. UPLC-MS: *t*_R_ = 5.58 min (generic method); MS (ESI) *m*/*z*: calcd. for C_22_H_32_N_3_O_3_S [M + H]^+^, 418.2; found, 418.2. ^1^H NMR (400 MHz, DMSO-*d*_6_): δ 7.05 (d, *J* = 8.6 Hz, 2H), 6.85 (d, *J* = 8.6 Hz, 2H), 4.65–4.57 (m, 1H), 4.17–4.13 (m, 2H), 2.47 (t, *J* = 7.3 Hz, 2H), 2.32 (s, 6H), 2.16–2.11 (m, 2H), 1.81–1.76 (m, 2H), 1.65–1.45 (m, 6H), 1.32–1.23 (m, 2H), 0.88 (t, *J* = 7.3 Hz, 3H). ^13^C NMR (101 MHz, DMSO-*d*_6_): δ 155.5, 147.8, 142.7, 134.9, 129.6, 116.1, 114.8, 69.3, 55.8, 38.5, 34.4, 33.8, 28.5, 22.1, 14.2, 13.5, 10.9. HRMS (ESI^+^) *m*/*z*: calcd. for C_22_H_32_N_3_O_3_S, 418.2164 [M + H]^+^; found, 418.2169.

##### *tert*-Butyl (1R,3r,5S)-3-(4-hexylphenoxy)-8-azabicyclo[3.2.1]octane-8-carboxylate (**67h**)

Following GP2, 4-hexylphenol (**66h**) (0.13 g, 0.73 mmol) and *tert*-butyl (1*R*,3*s*,5*S*)-3-hydroxybicyclo[3.2.1]octane-8-carboxylate (**65b**) (0.18 g, 00.77 mmol) were used in dry THF. Flash chromatography eluting with cyclohexane/EtOAc (2:8) gave the pure title compound (0.19 g, 67%) as a colorless oil. UPLC-MS: *t*_R_ = 3.06 min (method A); MS (ESI) *m*/*z*: calcd. for C_24_H_38_NO_3_ [M + H]^+^, 388.3; found, 388.2. ^1^H NMR (400 MHz, DMSO-*d*_6_): δ 7.12–7.05 (m, 2H), 6.87–6.74 (m, 2H), 4.66 (t, *J* = 4.8 Hz, 1H), 4.09–4.02 (m, 2H), 2.50–2.46 (m, 2H), 2.02 (d, *J* = 18.3 Hz, 4H), 1.84 (d, *J* = 17.2 Hz, 4H), 1.59–1.47 (m, 2H), 1.42 (s, 9H), 1.32–1.21 (m, 6H), 0.90–0.82 (m, 3H).

##### (1*R*,3*r*,5*S*)-3-(4-Hexylphenoxy)-8-azabicyclo[3.2.1]octane Trifluoroacetate (**69h**)

Following GP4, *tert*-butyl (1*R*,3*r*,5*S*)-3-(4-hexylphenoxy)-8-azabicyclo[3.2.1]octane-8-carboxylate (**67h**) was used to give the title compound (*quant*.), which was used in the next step without any purification.

##### (1*R*,3*r*,5*S*)-8-((3,5-Dimethyl-1*H*-pyrazol-4-yl)sulfonyl)-3-(4-hexylphenoxy)-8-azabicyclo[3.2.1]octane (**41**)

Following GP1, 3,5-dimethyl-1*H*-pyrazole-4-sulfonyl chloride (**52k**) (0.09 g, 0.46 mmol) and (1*R*,3*r*,5*S*)-3-(4-hexylphenoxy)-8-azabicyclo[3.2.1]octane trifluoroacetate (**69h**) (0.2 g, 0.5 mmol) were used in dry THF. Flash chromatography eluting with cyclohexane/EtOAc (3:7) gave the pure title compound (0.08 g, 39%) as a white solid. UPLC-MS: *t*_R_ = 6.61 min (generic method); MS (ESI) *m*/*z*: calcd. for C_24_H_36_N_3_O_3_S [M + H]^+^, 446.2; found, 446.3. ^1^H NMR (400 MHz, DMSO-*d*_6_): δ 12.97 (br s, 1H, NH), 7.08 (d, *J* = 8.5 Hz, 2H), 6.77 (d, *J* = 8.5 Hz, 2H), 4.62 (d, *J* = 4.7 Hz, 1H), 4.10–4.08 (m, 2H), 2.47 (t, *J* = 7.8 Hz, 2H), 2.37 (s, 3H), 2.28 (s, 3H), 2.05–1.93 (m, 6H), 1.63–1.60 (m, 2H), 1.53–1.48 (m, 2H), 1.26 (br s, 6H), 0.85 (t, *J* = 7.8 Hz, 3H). ^13^C NMR (101 MHz, DMSO-*d*_6_): δ 155.1, 147.5, 142.6, 134.8, 129.7, 115.8, 114.9, 69.3, 55.4, 36.8, 34.7, 31.6, 31.6, 28. 8, 28.4, 22.5, 14.4. HRMS (ESI^+^) *m*/*z*: calcd. for C_24_H_36_N_3_O_3_S, 446.2477 [M + H]^+^; found, 446.2479.

##### *tert*-Butyl (1*R*,3*r*,5*S*)-3-(4-((*E*)-3-methylbut-1-enyl)phenoxy)-8-azabicyclo[3.2.1]octane-8-carboxylate (**67i**)

Following GP2, 4-[(*E*)-3-methylbut-1-enyl]phenol (**66i**)^59^ (0.072 g, 0.44 mmol) and *tert*-butyl (1*R*,3*s*,5*S*)-3-hydroxybicyclo[3.2.1]octane-8-carboxylate (**65b**) (0.17 g, 0.46 mmol) were used in dry THF. Flash chromatography eluting with cyclohexane/EtOAc (9:1) gave the pure title compound (0.9 g, 55%), as a mixture of *E/Z* isomers, as a pale-yellow oil. UPLC-MS: *t*_R_ = 3.22 min (apolar method); MS (ESI) *m*/*z*: calcd. for C_23_H_34_NO_3_ [M + H]^+^, 372.2; found, 372.2. ^1^H NMR (400 MHz, DMSO-*d*_6_): δ 7.19 (d, *J* = 8.6 Hz, 2H), 6.87 (d, *J* = 8.6 Hz, 2H), 6.20 (d, *J* = 11.6 Hz, 1H), 5.36 (dd, *J* = 11.6, 10.1 Hz, 1H), 4.71 (*app*-t, *J* = 4.9 Hz, 1H), 4.06 (br s, 2H), 2.98–2.69 (m, 1H), 2.24–1.66 (m, 8H), 1.41 (s, 9H), 1.01 (d, *J* = 6.6 Hz, 6H).

##### (1*R*,3*r*,5*S*)-3-(4-((*E*)-3-Methylbut-1-enyl)phenoxy)-8-azabicyclo[3.2.1]octane Trifluoroacetate (**69i**)

Following GP4, *tert*-butyl (1*R*,3*r*,5*S*)-3-(4-((*E/Z*)-3-methylbut-1-enyl)phenoxy)-8-azabicyclo[3.2.1]octane-8-carboxylate (**67i**) was used to give the title compound (*quant*.), which was used in the next step without any purification.

##### (1*R*,3*r*,5*S*)-8-((3,5-Dimethyl-1*H*-pyrazol-4-yl)sulfonyl)-3-(4-((*E*)-3-methylbut-1-en-1-yl)phenoxy)-8-azabicyclo[3.2.1]octane (**71i**)

Following GP1, 3,5-dimethyl-1*H*-pyrazole-4-sulfonyl chloride (**52k**) (0.05 g, 0.25 mmol) and (1*R*,3*r*,5*S*)-3-(4-((*E*)-3-methylbut-1-enyl)phenoxy)-8-azabicyclo[3.2.1]octane trifluoroacetate (**69i**) (0.11 g, 0.28 mmol) were used in dry THF. Flash chromatography eluting with cyclohexane/EtOAc (4:6) to give the pure title compound (0.042 g, 39%) as a white solid. UPLC-MS: *t*_R_ = 2.98 min (apolar method); MS (ESI) *m*/*z*: calcd. for C_23_H_32_N_3_O_3_S [M + H]^+^, 430.2; found, 430.1. ^1^H NMR (400 MHz, DMSO-*d*_6_): δ 12.97 (br s, 1H), 7.30 (d, *J* = 8.7 Hz, 2H), 6.81 (d, *J* = 8.7 Hz, 2H), 6.27 (d, *J* = 16.0 Hz, 1H), 6.09 (dd, *J* = 6.7, 16.0 Hz, 1H), 4.67 (*app*-t, *J* = 4.2 Hz, 1H), 4.09 (br s, 2H), 2.46–2.37 (m, 1H), 2.32 (br s, 6H), 2.09–1.90 (m, 6H), 1.67–1.55 (m, 2H), 1.04 (d, *J* = 6.7 Hz, 6H).

##### (1*R*,3*r*,5*S*)-8-((3,5-Dimethyl-1*H*-pyrazol-4-yl)sulfonyl)-3-(4-isopentylphenoxy)-8-azabicyclo[3.2.1]octane (**42**)

To a solution of (1*R*,3*r*,5*S*)-8-((3,5-dimethyl-1*H*-pyrazol-4-yl)sulfonyl)-3-(4-((*E*)-3-methylbut-1-en-1-yl)phenoxy)-8-azabicyclo[3.2.1]octane (**71i**) (0.015 g, 0.035 mmol, 1.0 equiv) in EtOH (5.0 mL) were added ammonium formate (0.022 g, 0.35 mmol, 10 equiv) and 10% Pd/C (*ca.* 3 mg). The resulting mixture was sonicated at room temperature for ca. 30 min. The suspension was then filtered and the residue concentrated to dryness. The crude product was partitioned between EtOAc and brine, dried over Na_2_SO_4_, and concentrated under vacuo to furnish the crude product, which was purified by flash chromatography eluting with cyclohexane/EtOAc (1:1) to give the title compound (0.01 g, 66%) as a white solid. UPLC-MS: *t*_R_ = 3.98 min (apolar method); MS (ESI) *m*/*z*: calcd. for C_23_H_34_N_3_O_3_S [M + H]^+^, 432.2; found, 432.0. ^1^H NMR (400 MHz, DMSO-*d*_6_): δ 12.98 (br s, 1H, NH), 7.09 (d, *J* = 8.6 Hz, 2H), 6.77 (d, *J* = 8.6 Hz, 2H), 4.62 (t, *J* = 4.1 Hz, 1H), 4.09 (br s, 2H), 2.32 (s, 6H), 2.04–1.93 (m, 6H), 1.63–1.60 (m, 2H), 1.55–1.49 (m, 1H), 1.4–1.38 (m, 2H), 0.90 (d, *J* = 6.6 Hz, 6H). HRMS (ESI^+^) *m*/*z*: calcd. for C_23_H_34_N_3_O_3_S, 432.2321 [M + H]^+^; found, 432.2337.

##### *tert*-Butyl (1*R*,3*r*,5*S*)-3-(4-(Trifluoromethyl)phenoxy)-8-azabicyclo[3.2.1]octane-8-carboxylate (**67j**)

Following GP2, 4-(trifluoromethyl)phenol (**66j**) (0.118 g, 0.73 mmol) and *tert*-butyl (1*R*,3*s*,5*S*)-3-hydroxybicyclo[3.2.1]octane-8-carboxylate (**65b**) (0.18 g, 0.77 mmol) were used in dry THF. Flash chromatography eluting with cyclohexane/EtOAc (9:1) gave the pure title compound (0.21 g, 77%) as a white solid. UPLC-MS: *t*_*R*_ = 2.53 min (apolar method); MS (ESI) *m*/*z*: calcd. for C_19_H_25_F_3_NO_3_ [M + 2]^+^: 372.2; found, 372.1. ^1^H NMR (400 MHz, DMSO-*d*_6_): δ 7.64 (d, *J* = 8.6 Hz, 2H), 7.07 (d, *J* = 8.6 Hz, 2H), 4.83 (app-t, *J* = 4.9 Hz, 1H), 4.07 (br s, 2H), 2.14–1.70 (m, 8H), 1.42 (s, 9H).

##### (1*R*,3*r*,5*S*)-3-(4-Trifluoromethyl-phenoxy)-8-azabicyclo[3.2.1]octane Trifluoroacetate (**69j**)

Following GP4, *tert*-butyl (1*R*,3*r*,5*S*)-3-(4-(trifluoromethyl)phenoxy)-8-azabicyclo[3.2.1]octane-8-carboxylate (**67j**) was used to give the title compound (*quant*.), which was used in the next step without any purification.

##### (1*R*,3*r*,5*S*)-8-((3,5-Dimethyl-1*H*-pyrazol-4-yl)sulfonyl)-3-(4-(trifluoromethyl)phenoxy)-8-azabicyclo[3.2.1]octane (**43**)

Following GP1, 3,5-dimethyl-1*H*-pyrazole-4-sulfonyl chloride (**52k**) (0.1 g, 0.51 mmol) and (1*R*,3*r*,5*S*)-3-(4-trifluoromethyl-phenoxy)-8-azabicyclo[3.2.1]octane trifluoroacetate (**69j**) (0.22 g, 0.56 mmol) were used in dry THF. Flash chromatography eluting with cyclohexane/EtOAc (1:1) gave the pure title compound (0.2 g, 91%) as a white solid. UPLC-MS: *t*_R_ = 4.97 min (generic method); MS (ESI) *m*/*z*: calcd. for C_19_H_23_F_3_N_3_O_3_S [M + H]^+^, 430.1; found, 430.0. ^1^H NMR (400 MHz, DMSO-*d*_6_): δ 7.65 (d, *J* = 8.6 Hz, 2H), 7.06 (d, *J* = 8.6 Hz, 2H), 4.12–4.10 (m, 2H), 4.81 (t, *J* = 4.8 Hz, 1H), 2.33 (s, 6H), 2.12–2.06 (m 2H), 1.99–1.96 (m, 4H), 1.65–1.62 (m, 2H). ^13^C NMR (101 MHz, DMSO-*d*_6_): δ 160.1, 127.6, 127.5, 127.5, 127.5, 124.9 (d, *J* = 271.4 Hz), 121.7, 121.3, 116.2, 114.9, 70.0, 55.3, 36.7, 28.4. ^19^F NMR (565 MHz, DMSO-*d*_6_): δ −58.83. HRMS (ESI^+^) *m*/*z*: calcd. for C_19_H_23_F_3_N_3_O_3_S, 430.1412 [M + H]^+^; found, 430.1416.

##### *tert*-Butyl (1*R*,3*r*,5*S*)-3-(4-methoxyphenoxy)-8-azabicyclo[3.2.1]octane-8-carboxylate (**67k**)

Following GP2, 4-methoxyphenol (**66k**) (0.09 g, 0.73 mmol) and *tert*-butyl (1*R*,3*s*,5*S*)-3-hydroxybicyclo[3.2.1]octane-8-carboxylate (**67b**) (0.18 g, 0.8 mmol) were used in dry THF. Flash chromatography eluting with EtOAc gave the pure title compound (0.09 g, 37%) as a colorless oil. UPLC-MS: *t*_R_ = 1.92 min (generic method); MS (ESI) *m*/*z*: calcd. for C_19_H_28_NO_4_ [M + H]^+^, 334.2; found, 334.1. ^1^H NMR (400 MHz, DMSO-*d*_6_): δ 6.90–6.78 (m, 4H), 4.60 (t, *J* = 4.8 Hz, 1H), 4.10–4.00 (m, 2H), 3.70 (s, 3H), 2.15–1.94 (m, 4H), 1.92–1.77 (m, 4H), 1.41 (s, 9H).

##### (1*R*,3*r*,5*S*)-3-(4-Methoxyphenoxy)-8-azabicyclo[3.2.1]octane Trifluoroacetate (**69k**)

Following GP4, *tert*-butyl (1*R*,3*r*,5*S*)-3-(4-methoxyphenoxy)-8-azabicyclo[3.2.1]octane-8-carboxylate (**67k**) was used to give the title compound (*quant*.), which was used in the next step without any purification.

##### (1*R*,3*r*,5*S*)-8-((3,5-Dimethyl-1*H*-pyrazol-4-yl)sulfonyl)-3-(4-methoxyphenoxy)-8-azabicyclo[3.2.1]octane (**44**)

Following GP1, 3,5-dimethyl-1*H*-pyrazole-4-sulfonyl chloride (**52k**) (0.05 g, 0.26 mmol) and (1*R*,3*r*,5*S*)-3-(4-methoxyphenoxy)-8-azabicyclo[3.2.1]octane trifluoroacetate (**69k**) (0.1 g, 0.29 mmol) were used in dry THF. Flash chromatography eluting with DCM/MeOH (98:2) gave the pure title compound (0.06 g, 59%) as a white solid. UPLC-MS: *t*_R_ = 4.15 (generic method); MS (ESI) *m*/*z*: calcd. for C_19_H_26_N_3_O_4_S [M + H]^+^, 392.2; found, 392.2. ^1^H NMR (400 MHz, DMSO-*d*_6_): δ 12.97 (br s, 1H, *NH*), 6.86–6.80 (m, 4H), 4.56 (t, *J* = 4.6 Hz, 1H), 4.09–4.07 (m, 2H), 3.69 (s, 3H), 2.37 (s, 3H), 2.27 (s, 3H), 2.04–1.93 (m, 6H), 1.62–1.60 (m, 2H). ^13^C NMR (101 MHz, DMSO-*d*_6_): δ 153. 9, 151.0, 144. 5, 117.2, 115.2, 70.0, 55.8, 55.5, 36.8, 28.4. HRMS (ESI^+^) *m*/*z*: calcd. for C_19_H_26_N_3_O_4_S, 392.1644 [M + H]^+^; found, 392.1649.

##### *tert*-Butyl (1*R*,3*r*,5*S*)-3-(4-cyanophenoxy)-8-azabicyclo[3.2.1]octane-8-carboxylate (**67l**)

Following GP2, 4-cyanophenol (**66l**) (0.09 g, 0.73 mmol) and *tert*-butyl (1*R*,3*s*,5*S*)-3-hydroxybicyclo[3.2.1]octane-8-carboxylate (**67b**) (0.18 g, 0.77 mmol) were used in dry THF. Flash chromatography eluting with EtOAc gave the pure title compound (0.19 g, 82%) as a colorless oil. UPLC-MS: *t*_R_ = 1.66 (generic method); MS (ESI) *m*/*z*: calcd. for C_19_H_25_N_2_O_3_ [M + H]^+^, 328.2; found, 328.1. ^1^H NMR (400 MHz, DMSO-*d*_6_): δ 7.80–7.66 (m, 2H), 7.29–7.01 (m, 2H), 4.83 (t, *J* = 4.8 Hz, 1H), 4.10–4.00 (m, 2H), 2.04–1.75 (m, 8H), 1.40 (s, 9H).

##### (1*R*,3*s*,5*S*)-3-(4-Cyanophenoxy)-8-azabicyclo[3.2.1]octane Trifluoroacetate (**69l**)

Following GP4, *tert*-butyl (1*R*,3*r*,5*S*)-3-(4-cyanophenoxy)-8-azabicyclo[3.2.1]octane-8-carboxylate (**67l**) was used to give the title compound (*quant*.), which was used in the next step without any purification.

##### (1*R*,3*r*,5*S*)-8-((3,5-Dimethyl-1*H*-pyrazol-4-yl)sulfonyl)-3-(4-ethynylphenoxy)-8-azabicyclo[3.2.1]octane (**45**)

Following GP1, 3,5-dimethyl-1*H*-pyrazole-4-sulfonyl chloride (**52k**) (0.1 g, 0.51 mmol) and (1*R*,3*r*,5*S*)-3-(4-cyanophenoxy)-8-azabicyclo[3.2.1]octane trifluoroacetate (**69l**) (0.22 g, 0.56 mmol) were used in dry THF. Flash chromatography eluting with EtOAc gave the pure title compound (0.11 g, 57%) as a white solid. UPLC-MS: *t*_R_ = 3.87 (generic method); MS (ESI) *m*/*z*: calcd. for C_19_H_23_N_4_O_3_S [M + H]^+^, 387.1; found, 387.1. ^1^H NMR (400 MHz, DMSO-*d*_6_): δ 7.74 (d, *J* = 8.8 Hz, 2H), 7.03 (d, *J* = 8.8 Hz, 2H), 4.80 (t, *J* = 4.9 Hz, 1H), 4.11–4.09 (m, 2H), 2.31 (s, 6H), 1.11–2.05 (m, 2H), 1.98–1.92 (m, 4H), 1.63–1.60 (m, 2H). ^13^C NMR (101 MHz, DMSO-*d*_6_): δ 160.8, 147.8, 142.7, 134.8, 119.5, 116.8, 114.8, 103.2, 70.3, 55.3, 36.7, 28.4, 13.5, 10.9. HRMS (ESI^+^) *m*/*z*: calcd. for C_19_H_23_N_4_O_3_S, 387.1491 [M + H]^+^; found, 387.1502.

##### *tert*-Butyl (1*R*,3*r*,5*S*)-3-(4-Fluoro-phenoxy)-8-azabicyclo[3.2.1]octane-8-carboxylate (**67m**)

Following GP2, 4-fluorophenol (**66m**) (0.118 g, 1.06 mmol) and *tert*-butyl (1*R*,3*s*,5*S*)-3-hydroxybicyclo[3.2.1]octane-8-carboxylate (**65b**) (0.25 g, 1.11 mmol) were used in dry THF. Flash chromatography eluting with cyclohexane/EtOAc (9:1) gave the pure title compound (0.174 g, 51%) as a white solid. UPLC-MS: *t*_R_ = 1.98 min (apolar method); MS (ESI) *m*/*z*: calcd. for C_18_H_25_FNO_3_ [M + H]^+^, 322.2; found, 322.1. ^1^H NMR (400 MHz, DMSO-*d*_6_): δ 7.11–7.09 (m, 2H), 6.89–6.83 (m, 2H), 4.61 (t, *J* = 4.8 Hz, 1H), 4.13–4.08 (m, 2H), 2.02–1.66 (m, 8H), 1.42 (s, 9H).

##### (1*R*,3*r*,5*S*)-3-(2-Fluoro-4-methyl-phenoxy)-8-azabicyclo[3.2.1]octane Trifluoroacetate (**69m**)

Following GP4, *tert*-butyl (1*R*,3*r*,5*S*)-3-(4-fluoro-phenoxy)-8-azabicyclo[3.2.1]octane-8-carboxylate (**67m**) was used to give the title compound (*quant*.), which was used in the next step without any purification.

##### (1*R*,3*r*,5*S*)-8-((3,5-Dimethyl-1*H*-pyrazol-4-yl)sulfonyl)-3-(4-fluorophenoxy)-8-azabicyclo[3.2.1]octane (**46**)

Following GP1, 3,5-dimethyl-1*H*-pyrazole-4-sulfonyl chloride (**52k**) (0.166 g, 0.85 mmol) and (1*R*,3*r*,5*S*)-3-(4-fluoro-phenoxy)-8-azabicyclo[3.2.1]octane trifluoroacetate (**69m**) (0.32 g, 0.94 mmol) were used in dry THF. Flash chromatography eluting with cyclohexane/EtOAc (1:1) gave the pure title compound (0.099 g, 31%) as a white solid. UPLC-MS: *t*_R_ = 4.39 min (generic method); MS (ESI) *m*/*z*: calcd. for C_18_H_23_FN_3_O_3_S [M + H]^+^, 380.1; found, 380.2. ^1^H NMR (400 MHz, DMSO-*d*_6_): δ 12.97 (br s, 1H, *NH*), 7.13–7.09 (m, 2H), 6.916.87 (m, 2H), 4.63 (t, *J* = 4.8 Hz, 1H), 4.10–4.08 (m, 2H), 2.37 (s, 3H), 2.27 (s, 3H), 2.06–1.92 (m, 6H), 1.64–1.60 (m, 2H). ^13^C NMR (101 MHz, DMSO-*d*_6_): δ, 156.9 (d, *J* = 236.2 Hz), 153.5, 117.4, 117. 3, 116.6, 116.3, 114.9, 70.1, 55.4, 36.7, 28.4, 12.1. ^19^F NMR (565 MHz, DMSO-*d*_6_): δ −122.75. HRMS (ESI^+^) *m*/*z*: calcd. for C_18_H_23_FN_3_O_3_S, 380.1444 [M + H]^+^; found, 380.1444.

##### *tert*-Butyl (1*R*,3*r*,5*S*)-3-(4-propoxyphenoxy)-8-azabicyclo[3.2.1]octane-8-carboxylate (**67n**)

Following GP2, 4-propoxyphenol (**66n**) (0.11 g, 0.73 mmol) and *tert*-butyl (1*R*,3*s*,5*S*)-3-hydroxybicyclo[3.2.1]octane-8-carboxylate (**65b**) (0.18 g, 0.77 mmol) were used in dry THF. Flash chromatography eluting with EtOAc gave the pure title compound (0.13 g, 49%) as a colorless oil. UPLC-MS: *t*_R_ = 2.66 min (generic method); MS (ESI) *m*/*z*: calcd. for C_21_H_32_NO_4_ [M + H]^+^, 362.2; found, 362.1. ^1^H NMR (400 MHz, DMSO-*d*_6_): δ 6.85–6.75 (m, 7H), 4.53 (t, *J* = 4.7 Hz, 1H), 4.09–4.07 (m, 2H), 3.80 (t, *J* = 6.5 Hz, 2H), 2.36–1.64 (m, 5H), 1.62–1.57 (m, 2H), 1.42 (s, 9H), 0.93 (t, *J* = 7.4 Hz, 3H).

##### (1*R*,3*r*,5*S*)-3-(4-Propoxyphenoxy)-8-azabicyclo[3.2.1]octane Trifluoroacetate (**69n**)

Following GP4, *tert*-butyl (1*R*,3*r*,5*S*)-3-(4-propoxyphenoxy)-8-azabicyclo[3.2.1]octane-8-carboxylate (**67n**) was used to give the title compound (*quant*.), which was used in the next step without any purification.

##### (1*R*,3*r*,5*S*)-8-((3,5-Dimethyl-1*H*-pyrazol-4-yl)sulfonyl)-3-(4-propoxyphenoxy)-8-azabicyclo[3.2.1]octane (**47**)

Following GP1, 3,5-dimethyl-1*H*-pyrazole-4-sulfonyl chloride (**52k**) (0.07 g, 0.36 mmol) and (1*R*,3*r*,5*S*)-3-(4-propoxyphenoxy)-8-azabicyclo[3.2.1]octane trifluoroacetate (**69n**) (0.15 g, 0.4 mmol) were used in dry THF. Flash chromatography eluting with DCM/MeOH (98:2) gave the pure title compound (0.08 g, 53%) as a white solid. UPLC-MS: *t*_R_ = 5.05 (generic method); MS (ESI) *m*/*z*: calcd. for C_21_H_30_N_3_O_4_S [M + H]^+^, 420.2; found, 420.2. ^1^H NMR (400 MHz, DMSO-*d*_6_): δ 12.98 (br s, 1H, *NH*), 6.85–6.78 (m, 4H), 4.54 (t, *J* = 4.7 Hz, 1H), 4.09–4.07 (m, 2H), 3.84 (t, *J* = 6.5 Hz, 2H), 2.36 (s, 3H), 2.27 (s, 3H), 2.04–1.92 (m, 6H), 1.73–1.64 (m, 2H), 1.62–1.59 (m, 2H), 0.96 (t, *J* = 7.4 Hz, 3H). ^13^C NMR (101 MHz, CDCl_3_): δ 158.0, 155.7, 152.6, 121.9, 120.6, 119.7, 74.7, 74.5, 60.2, 41.5, 33.1, 27.3, 18.2, 15.6. HRMS (ESI^+^) *m*/*z*: calcd. for C_21_H_30_N_3_O_4_S, 420.1957 [M + H]^+^; found, 420.1961.

##### *tert*-Butyl (1*R*,3*r*,5*S*)-3-(4-formylphenoxy)-8-azabicyclo[3.2.1]octane-8-carboxylate (**67o**)

Following GP2, 4-hydroxybenzaldehyde (**66o**) (0.089 g, 0.73 mmol) and *tert*-butyl (1*R*,3*s*,5*S*)-3-hydroxybicyclo[3.2.1]octane-8-carboxylate (**65b**) (0.18 g, 0.77 mmol) were used in dry THF. Flash chromatography eluting with cyclohexane/EtOAc (8:2) gave the pure title compound (0.2 g, 83%) as a white solid. UPLC-MS: *t*_*R*_ = 1.87 min (generic method); MS (ESI) *m*/*z*: calcd. for C_19_H_26_NO_4_ [M + H]^+^, 332.2; found, 331.1. ^1^H NMR (400 MHz, DMSO-*d*_6_): δ 9.86 (s, 1H), 7.86 (d, *J* = 8.7 Hz, 2H), 7.08 (d, *J* = 8.7 Hz, 2H), 4.88 (app-t, *J* = 4.8 Hz, 1H), 4.07 (br s, 2H), 2.23–1.72 (m, 8H), 1.42 (s, 9H).

##### (1*R*,3*r*,5*S*)-3-(4-Formylphenoxy)-8-azabicyclo[3.2.1]octane Trifluoracetate (**69o**)

Following GP4, *tert*-butyl (1*R*,3*r*,5*S*)-3-(4-formylphenoxy)-8-azabicyclo[3.2.1]octane-8-carboxylate (**67o**) was used to give the title compound (*quant*.), which was used in the next step without any purification.

##### (4-(((1*R*,3*r*,5*S*)-8-((3,5-Dimethyl-1*H*-pyrazol-4-yl)sulfonyl)-8-azabicyclo[3.2.1]octan-3-yl)oxy)phenyl)methanol (**72**)

*tert*-Butyl (1*R*,3*r*,5*S*)-3-(4-formylphenoxy)-8-azabicyclo[3.2.1]octane-8-carboxylate (0.16 g, 0.58 mmol, 1.0 equiv) (**71o**) was dissolved in MeOH (10 mL) and the resulting solution was cooled to 0 °C. Sodium borohydride (0.055 g, 1.45 mmol, 2.5 equiv) was slowly added and the mixture was stirred at room temperature for 1 h. The reaction mixture was quenched with a sat. aq. NH_4_Cl solution (15 mL) and extracted with DCM (2 × 15 mL). The organic extracts were dried over Na_2_SO_4_ and concentrated under vacuo to furnish the crude product, which was used in the next step without any further purification.

##### (1*R*,3*r*,5*S*)-8-((3,5-Dimethyl-1*H*-pyrazol-4-yl)sulfonyl)-3-(4-(ethoxymethyl)phenoxy)-8-azabicyclo[3.2.1]octane (**48**)

A solution of (4-(((1*R*,3*r*,5*S*)-8-((3,5-dimethyl-1*H*-pyrazol-4-yl)sulfonyl)-8-azabicyclo[3.2.1]octan-3-yl)oxy)phenyl)methanol (**72**) (0.16 g, 0.48 mmol, 1.0 equiv) in EtOH (3.0 mL) was treated with Amberlist-15 (1.0 equiv). The reaction was left at reflux for 16 h. Upon completion of the reaction, the solution was filtered and the solvent evaporated to give the pure title compound (0.11 g, 55%) as a white solid. UPLC-MS: *t*_R_ = 4.48 min (generic method); MS (ESI) *m*/*z*: calcd. for C_21_H_30_N_3_O_4_S [M + H]^+^, 420.2; found, 420.0. ^1^H NMR (400 MHz, DMSO-*d*_6_): δ 12.96 (br s, 1H, *NH*), 7.22 (d, *J* = 8.6 Hz, 2H), 6.83 (d, *J* = 8.6 Hz, 2H), 4.66 (t, *J* = 4.8 Hz, 1H), 4.34 (s, 2H), 4.09–4.07 (m, 2H), 3.43 (q, *J* = 7.0 Hz, 3H), 2.31 (s, 6H), 2.07–1.93 (m, 5H), 1.63–1.60 (m, 2H), 1.12 (t, *J* = 7.0 Hz, 3H). ^13^C NMR (101 MHz, DMSO-*d*_6_): δ 156.5, 131.3, 129.7, 115.7, 114.9, 71.7, 69.4, 65.1, 55.4, 36.8, 28.4, 15.6. HRMS (ESI^+^) *m*/*z*: calcd. for C_21_H_30_N_3_O_4_S, 420.1957 [M + H]^+^; found, 420.1957.

##### *tert*-Butyl (1*R*,3*r*,5*S*)-3-((5-((*E*/*Z*)-but-1-en-1-yl)pyrazin-2-yl)oxy)-8-azabicyclo[3.2.1]octane-8-carboxylate (**74c**)

Step 1. To a solution of *n*-propyl-triphenylphosphonium bromide (0.745 g, 1.94 mmol, 1.1 equiv) in dry THF (168 mL), *n*-BuLi (2.5 M in hexane) (0.785 mL, 1.94 mmol, 1.1 equiv) was added dropwise at 0 °C, and the reaction mixture was stirred at the same temperature for 45 min. 5-Chloropyrazine-2-carbaldehyde (0.745 g, 1.94 mmol, 1.1 equiv) was added and the crude mixture stirred at room temperature for 16 h, quenched with water (20 mL), and extracted with EtOAc (2 × 30 mL). The organic extracts were washed with brine, dried over Na_2_SO_4_, and concentrated under vacuo to furnish a crude product, which was purified by flash chromatography eluting with cyclohexane/EtOAc (9:1) to give (*E*/*Z*)-2-(but-1-en-1-yl)-5-chloro-pyrazine (**73c**) (0.09 g, 30%) as a 1:2 *E*/*Z* mixture of isomers. UPLC-MS: *t*_R_ = 2.38 (generic method); MS (ESI) *m*/*z*: calcd. for C_8_H_10_ClN_2_ [M + H]^+^, 169.0; found, 169.1. *Z*-isomer: ^1^H NMR (400 MHz, DMSO-*d*_6_): δ 8.77 (d, *J* = 1.4 Hz, 1H), 8.46 (d, *J* = 1.4 Hz, 1H), 6.47 (dt, *J* = 11.8, 1.8 Hz, 1H), 6.09 (dt, *J* = 11.8, 7.5 Hz, 1H), 2.69–2.60 (m, 2H), 1.04 (t, *J* = 7.5 Hz, 3H). *E*-isomer: ^1^H NMR (400 MHz, DMSO-*d*_6_): δ 8.68 (d, *J* = 1.4 Hz, 1H), 8.53 (d, *J* = 1.4 Hz, 1H), 6.97 (dt, *J* = 15.8, 6.5 Hz, 1H), 6.57 (dt, *J* = 15.8, 1.7 Hz, 1H), 2.34–2.25 (m, 2H), 1.09 (t, *J* = 7.4 Hz, 3H).

Step 2. Following GP3, *tert*-butyl (1*R*,3*r*,5*S*)-3-hydroxy-8-azabicyclo[3.2.1]octane-8-carboxylate (**65a**) (0.123 g, 0.54 mmol) and (*E*/*Z*)-2-(but-1-enyl)-5-chloro-pyrazine (**73c**) (0.09 g, 0.54 mmol) were used in dry THF. Flash chromatography eluting with DCM/MeOH (95:5) gave the pure title compound (0.035 g, 18%), as a 1:2 *E*/*Z* mixture of isomers, as a white solid. UPLC-MS: *t*_R_ = 2.34 and 2.45 (generic method); MS (ESI) *m*/*z*: calcd. for C_20_H_30_N_3_O_3_ [M + H]^+^, 360.2; found, 360.1. *Z*-isomer: ^1^H NMR (400 MHz, DMSO-*d*_6_): δ 8.28 (d, *J* = 1.3 Hz, 1H), 8.13 (d, *J* = 1.3 Hz, 1H), 6.38–6.34 (m, 1H), 5.82 (dt, *J* = 11.8, 7.4 Hz, 1H), 5.32–5.28 (m, 1H), 4.10 (br s, 2H), 2.64–2.56 (m, 1H), 2.10–2.07 (m, 4H), 1.89–1.84 (m, 5H), 1.03 (t, *J* = 7.5 Hz, 3H). *E*-isomer: ^1^H NMR (400 MHz, DMSO-*d*_6_): δ 8.28 (d, *J* = 1.3 Hz, 1H), 8.21 (d, *J* = 1.3 Hz, 1H), 6.70 (dt, *J* = 15.7, 6.5 Hz, 1H), 6.49–6.44 (m, 1H), 5.32–5.28 (m, 1H), 4.10 (br s, 2H), 2.28–2.21 (m, 1H), 2.10–2.07 (m, 4H), 1.89–1.84 (m, 5H), 1.07 (t, *J* = 7.5 Hz, 3H).

#### (1*R*,3*r*,5*S*)-3-((5-Butylpyrazin-2-yl)oxy)-8-azabicyclo[3.2.1]octane Trifluoroacetate (**77**)

Step 1. To a solution of *tert*-butyl (1*R*,3*r*,5*S*)-3-((5-((*E/Z*)-but-1-en-1-yl)pyrazin-2-yl)oxy)-8-azabicyclo[3.2.1]octane-8-carboxylate (**74c**) (0.03 g, 0.084 mmol, 1.0 equiv) in EtOH (4.0 mL) were added cyclohexene (0.069 g, 0.84 mmol, 10 equiv) and 10% Pd/C (*ca.* 30 mg). The mixture was kept under refluxing for 2 h, cooled to room temperature, and the resulting suspension filtered and the residue concentrated to dryness. The crude product was partitioned between EtOAc and brine, dried over Na_2_SO_4_, and concentrated under vacuo to furnish *tert*-butyl (1*R*,3*r*,5*S*)-3-((5-butylpyrazin-2-yl)oxy)-8-azabicyclo[3.2.1]octane-8-carboxylate, which was used in the next step without any further purification.

Step 2. Following GP4, tert-butyl (1*R*,3*r*,5*S*)-3-((5-butylpyrazin-2-yl)oxy)-8-azabicyclo[3.2.1]octane-8-carboxylate was used to give the title compound (*quant*.), which was used in the next step without any purification.

##### (1*R*,3*r*,5*S*)-3-((5-Butylpyrazin-2-yl)oxy)-8-((3,5-dimethyl-1*H*-pyrazol-4-yl)sulfonyl)-8-azabicyclo[3.2.1]octane (**49**)

Following GP1, 3,5-dimethyl-1*H*-pyrazole-4-sulfonyl chloride (**52k**) (0.02 g, 0.1 mmol) and (1R,3r,5S)-3-((5-butylpyrazin-2-yl)oxy)-8-azabicyclo[3.2.1]octane trifluoroacetate (**77**) (0.04 g, 0.11 mmol) were used in dry THF. Flash chromatography eluting with DCM/MeOH (95:5) gave the pure title compound (0.021 g, 51%) as a white solid. UPLC-MS: *t*_*R*_ = 4.76 (generic method); MS (ESI) *m*/*z*: calcd. for C_20_H_30_N_5_O_3_S [M + H]^+^, 420.2; found, 420.4. ^1^H NMR (400 MHz, DMSO-d_6_): δ 12.98 (br s, 1H, NH), 8.16 (d, *J* = 1.4 Hz, 1H), 8.05 (d, *J* = 1.4 Hz, 1H), 5.24 (t, *J* = 5.0 Hz, 1H), 4.12–4.10 (m, 2H), 2.66 (t, *J* = 7.4 Hz, 2H), 2.37 (s, 3H), 2.28 (s, 3H), 2.12–1.94 (m, 6H), 1.65–1.57 (m, 4H), 1.35–1.24 (m, 2H), 0.89 (t, *J* = 7.3 Hz, 3H). ^13^C NMR (101 MHz, DMSO-d_6_): δ 157.6, 149.1, 147.8, 142. 7, 139.5, 134.9, 114.9, 68.6, 55.5, 37.4, 33.4, 31.7, 28.4, 22.1, 14.2, 13.5, 10.9. HRMS (ESI^+^) *m*/*z*: calcd. for C_20_H_30_N_5_O_3_S, 420.2069 [M + H]^+^; found, 420.2067.

##### 2-Chloro-5-(ethoxymethyl)pyrazine (**73d**)

(5-Chloropyrazin-2-yl)methanol (0.3 g, 2.08 mmol, 1.0 equiv) and iodoethane (0.17 mL, 2.08 mmol, 1.0 equiv) were dissolved in THF (10 mL), and the solution was cooled to 0 °C. Sodium hydride (0.25 g, 6.24 mmol, 3.0 equiv) was then added, and the reaction mixture was stirred at room temperature for 16 h. The reaction mixture was quenched with a sat. sol. NaHCO_3_ (15 mL) and extracted with EtOAc (2 × 15 mL). The organic extracts were dried over Na_2_SO_4_ and concentrated under vacuo to furnish the crude product, which was purified by flash chromatography eluting with cyclohexane/EtOAc (2:8) to give the pure title compound (0.03 g, 9%) as a colorless oil. UPLC-MS: *t*_*R*_ = 1.70 (generic method); MS (ESI) *m*/*z*: calcd. for C_7_H_10_ClN_2_O [M + H]^+^, 173.0; found, 173.0. ^1^H NMR (400 MHz, DMSO-d_6_): δ 8.77 (d, *J* = 1.4 Hz, 1H), 8.53 (d, *J* = 1.3 Hz, 1H), 4.62 (s, 2H), 3.59 (q, *J* = 7.0 Hz, 2H), 1.19 (t, *J* = 7.0 Hz, 3H).

##### *tert*-Butyl(1*R*,3*r*,5*S*)-3-((5-(ethoxymethyl)pyrazin-2-yl)oxy)-8-azabicyclo[3.2.1]octane-8-carboxylate (**74d**)

Following GP3, *tert*-butyl (1*R*,3*r*,5*S*)-3-hydroxy-8-azabicyclo[3.2.1]octane-8-carboxylate (**65a**) (0.2 g, 0.88 mmol) and 2-chloro-5-(ethoxymethyl)pyrazine (**73d**) (0.15 g, 0.88 mmol) were used in dry THF. Flash chromatography eluting with cyclohexane/EtOAc (2:8) gave the pure title compound (0.01 g, 31%) as a colorless oil. UPLC-MS: *t*_*R*_ = 2.56 (generic method); MS (ESI) *m*/*z*: calcd. for C_19_H_30_N_2_O_4_ [M + H]^+^, 364.1; found, 364.0. ^1^H NMR (400 MHz, DMSO-*d*_6_): δ 8.25 (d, *J* = 1.4 Hz, 1H), 8.20 (d, *J* = 1.4 Hz, 1H), 5.32 (t, *J* = 4.9 Hz, 1H), 4.50 (s, 2H), 4.18–4.01 (m, 2H), 3.55 (q, *J* = 7.0 Hz, 2H), 2.13–2.01 (m, 4H), 1.96–1.78 (m, 4H), 1.43 (s, 9H), 1.16 (t, *J* = 7.0 Hz, 3H).

##### (1*R*,3*r*,5*S*)-3-(5-(Ethoxymethyl)pyrazin-2-yl)oxy-8-azabicyclo[3.2.1]octane Trifluoroacetate (**78d**)

Following GP4, *tert*-butyl(1*R*,3*r*,5*S*)-3-[5-(ethoxymethyl)pyrazin-2-yl]oxy-8-azabicyclo[3.2.1]octane-8-carboxylate (**74d**) was used to give the title compound (quant.), which was used in the next step without any purification.

##### (1*R*,3*r*,5*S*)-8-((3,5-Dimethyl-1*H*-pyrazol-4-yl)sulfonyl)-3-((5-(ethoxymethyl)pyrazin-2-yl)oxy)-8-azabicyclo[3.2.1]octane (**50**)

Following GP1, 3,5-dimethyl-1*H*-pyrazole-4-sulfonyl chloride (**52k**) (0.01 g, 0.05 mmol) and (1*R*,3*r*,5*S*)-3-(5-(ethoxymethyl)pyrazin-2-yl)oxy-8-azabicyclo[3.2.1]octane trifluoroacetate (**78d**) (0.021 g, 0.055 mmol) were used in dry THF. Flash chromatography eluting with DCM/MeOH (98:2) gave the pure title compound (0.01 g, 48%) as a white solid. UPLC-MS: *t*_*R*_ = 3.62 (generic method); MS (ESI) *m*/*z*: calcd. for C_19_H_28_N_5_O_4_S [M + H]^+^, 422.2; found, 422.2. ^1^H NMR (400 MHz, DMSO-*d*_6_): δ 8.22 (d, *J* = 1.3 Hz, 1H), 8.19 (d, *J* = 1.3 Hz, 1H), 5.28 (t, *J* = 4.9 Hz, 1H), 4.48 (s, 2H, CH_2_), 4.12 (br s, 2H), 3.53 (q, *J* = 7.0 Hz, 2H), 2.33 (s, 6H), 2.13–1.95 (m, 6H), 1.66–1.63 (m, 2H), 1.15 (t, *J* = 7.0 Hz, 3H). ^13^C NMR (151 MHz, DMSO-*d*_6_): δ 158.5, 147. 7, 145.5, 142.8, 139. 8, 135.3, 114.9, 70.6, 68.9, 65.9, 55.4, 37.3, 28.4, 15.5, 13.3, 10.9. HRMS (ESI^+^) *m*/*z*: calcd. for C_19_H_28_N_5_O_4_S, 422.1862 [M + H]^+^; found, 422.1854.

### Biology

#### Cell Culture Conditions

Human recombinant proteins were obtained from HEK-293 stable overexpressing human NAAA, AC, and FAAH-1 cell lines, respectively. Cells were grown in Dulbecco’s modified Eagle medium (DMEM) containing 10% FBS, 1% glutamine, 1 mM sodium pyruvate, and 500 μg/mL geneticin (G418). To obtain a high-density cell proliferation, *h*-NAAA-HEK-293 cells were grown in BelloCell-500 bottle, a compact bioreactor. On the contrary, h-AC and h-FAAH-1-HEK-293 cells were grown in 150 mm dishes and scraped off with cold PBS 1× pH 7.4 at 80% confluence. Cell pellets were collected and stored at −80 °C until protein preparation.

#### Preparation of Enzyme-Enriched Lysate (*h*-NAAA and h-AC)

HEK-293 cells stably transfected with the human NAAA or human AC coding sequences were used as the enzyme source. Cells were suspended in 20 mM Tris-HCl (pH 7.4) with 0.32 M sucrose, sonicated, and centrifuged at 800*g* for 30 min at 4 °C. Supernatants were then centrifuged at 12,000*g* for 30 min at 4 °C. Pellets were resuspended in PBS buffer (pH 7.4) and subjected to three freeze-thaw cycles at −80 °C. The suspension was finally centrifuged at 105,000*g* for 1 h at 4 °C, supernatants were collected, protein concentration was measured, and samples were aliquoted and stored at −80 °C until use.

#### Preparation of Membrane-Enriched Lysate (h-FAAH-1)

Cell pellets, obtained by centrifugation at 300*g* for 7 min at 4 °C, were resuspended in 20 mM Tris-HCl pH 7.4, 0.32 M sucrose, disrupted by sonication (10 pulses, 5 times), and centrifuged (1000*g*, 10 min, 4 °C). Supernatants were then centrifuged at 12,000*g* for 10 min at 4 °C and then at 105,000*g* for 1 h at 4 °C. Membrane pellets were resuspended in PBS to obtain h-FAAH-1 preparation; the protein concentration was measured by the Bradford Protein Assay (BioRad) and samples were aliquoted and stored at −80 °C until use.

#### Fluorogenic Human-NAAA Assay

The assay was run in 96-well microplates (Black OptiPlate-96 F; PerkinElmer, Massachusetts, USA), in a total reaction volume of 200 μL. *h*-NAAA protein preparation (4 μg) was preincubated for 30 min with various concentrations of test compounds or vehicle control (DMSO 5%) in 100 mM citrate/phosphate buffer (pH 4.5) containing 3.0 mM DTT, 0.1% NP40, 0.05% BSA, and 150 mM NaCl. The background (no activity) samples were prepared using assay buffer without *h*-NAAA and DMSO (5%). *N*-(4-methyl-2-oxo-chromen-7-yl)-hexadecanamide (PAMCA)^60^ was used as a substrate (2 μM) and the reaction was carried out for 50 min at 37 °C. Fluorescence was measured with EnVision 2014 Multilabel Reader (PerkinElmer, Massachusetts, USA) using an excitation wavelength of 355 nm and an emission of 460 nm. IC_50_ values were calculated by non-linear regression analysis of log[concentration]/response curves generated with mean replicate values using a four-parameter Hill equation curve fitting with GraphPad Prism 5 (GraphPad Software Inc., CA, USA).

#### Fluorogenic Human-AC Assay

The assay was performed in Optiplate 96-wells black plates (PerkinElmer, Massachusetts, USA) in a total reaction volume of 250 μL. h-AC protein (2 μg) was preincubated for 10 min with various concentrations of test compounds or vehicle control (DMSO 5%) in 25 mM sodium acetate buffer (pH 4.5). The background (no activity) samples were prepared using assay buffer without h-AC and DMSO (5%). *N*-[(1S,2R)-2-hydroxy-1-(hydroxymethyl)-4-(2-oxochromen-7-yl)oxybutyl]dodecanamide was used as the substrate (5 μM) and the reaction was carried out for 3 h at 37 °C, and stopped with MeOH and 2.5 mg/mL NaIO_4_ (fresh solution in 100 mM glycine/NaOH buffer pH 10.6). The plates were further incubated for 2 h at 37 °C in the dark. Fluorescence was measured with the EnVision 2014 Multilabel Reader (PerkinElmer, Massachusetts, USA) at an excitation/emission wavelength of 355/460 nm. IC_50_ values were calculated by non-linear regression analysis of Log[concentration]/response curves generated with mean replicate values using a four-parameter Hill equation curve fitting with GraphPad Prism 5 (GraphPad Software Inc., CA, USA).

#### Fluorogenic Human-FAAH-1 Assay

The fluorescence assay to measure FAAH activity was performed in 96-well black plates (Black OptiPlate-96 F; PerkinElmer, Massachusetts, USA): 2.5 μg of human FAAH-1 membrane preparation was preincubated for 50 min at 37 °C, in 190 μL of assay buffer (50 mM Tris-HCl pH 7.4, 0.05% Fatty acid-free BSA) with 5 μL of inhibitor or 5 μL DMSO to measure FAAH total activity. The background (no activity) samples were prepared using 190 μL of assay buffer without human FAAH-1 and 5 μL of DMSO. The reaction was then started by the addition of 5 μL of substrate (AMC arachidonoyl amide, A6855 Merck) dissolved in DMSO and used at a final concentration of 800 nM. The reaction was carried out for 45 min at 37 °C and fluorescence was measured with the EnVision 2014 Multilabel Reader (PerkinElmer, Massachusetts, USA) (excitation wavelength, 355 nm/emission wavelength, 460 nm). The concentration causing half-maximal inhibition (IC_50_) was determined by non-linear regression analysis of the Log[concentration]/response curves generated with mean replicate values using a four-parameter Hill equation curve fitting with GraphPad Prism 5 (GraphPad Software Inc., CA, USA).

#### Human-NAAA Purification and Activation

*h*-NAAA was produced and purified from the *h*-NAAA-overexpressing HEK293 cell line.^61^ The purified enzyme was incubated in activation buffer (100 mM sodium phosphate/sodium citrate buffer, 3 mM DL-dithiothreitol (DTT), 0.1% Triton X100, pH 4.5) for 3 h at 37 °C, and the enzyme activation was checked by SDS-PAGE and Coomassie blue staining.

#### Competitive Activity-Based Protein Profiling (ABPP)

50 μL of lysosomal enrichment (0.5 mg/mL) from the *h*-NAAA-overexpressing HEK293 cell line was incubated 2 h at 37 °C with **50** or covalent inhibitor 4-cyclohexylbutyl-*N*-[(2S,3S)-2-methyl-1-(4-methylsulfonylphenoxy)-4-oxo-azetidin-3-yl]carbamate (*ARN15393*, see Figure S1, Supporting Information)^46^ at a final concentration of 20 μM (DMSO 2%). At the end of this preincubation time, the activity-based probe undec-10-ynyl-*N*-[(S)-2-oxoazetidin-3-yl]carbamate (*ARN14686*, see Figure S1, Supporting Information)^45^ was added at 20 μM for 15 min or for 4 h at 37 °C. Next, click chemistry reaction was performed by adding the following reagents at the indicated final concentrations: 100 μM azide-PEG3-alexa fluor 545 (CLK-AZ109, Jena Bioscience), 1.0 mM tris(2-carboxyethyl)phosphine (TCEP) hydrochloride, 100 μM tris-[(1-benzyl-1*H*-1,2,3-triazol-4-yl)methyl]amine (TBTA), and 1 mM CuSO_4_·5H_2_O.^62^ TBTA was first dissolved in DMSO at 83.5 mM and then diluted with four volumes of tert-butanol. The reaction was mixed by vortexing and incubated 2 h at 25 °C. Samples (10 μL) were analyzed by SDS-PAGE, and gel fluorescence was scanned at 532 nm wavelength (ChemidDoc MP, BIO-RAD).

### In Vitro ADMET

#### Aqueous Kinetic Solubility Assay

The aqueous kinetic solubility was determined from a 10 mM DMSO stock solution of test compound (**39** or **50**) in PBS buffer at pH 7.4. The study was performed by incubating an aliquot of 10 mM DMSO stock solution in PBS (pH 7.4) to a target concentration of 250 μM (2.5% DMSO). The incubation was carried out under shaking at 25 °C for 24 h, followed by centrifugation at 21,100*g* for 30 min. The supernatant was further diluted (4:1) with CH_3_CN, and the dissolved test compound was quantified by UV at 215 nm on a Waters ACQUITY UPLC-MS system consisting of a single quadrupole detector (SQD) mass spectrometer equipped with an electrospray ionization interface and a photodiode array detector (PDA) from Waters Inc. (Milford, MA, USA). Electrospray ionization in positive mode was used in the mass scan range 100–500 Da. The PDA range was 210–400 nm. The analyses were run on an ACQUITY UPLC BEH C18 column (50 × 2.1 mm ID, particle size 1.7 μm) with a VanGuard BEH C18 precolumn (5 × 2.1 mm ID, particle size 1.7 μm), using 10 mM NH_4_OAc in H_2_O at pH 5 adjusted with AcOH (A) and 10 mM NH_4_OAc in CN–H_2_O (95:5) at pH 5 (B) as the mobile phase. The aqueous kinetic solubility (in μM) was calculated by dividing the peak areas of dissolved test compound and test compound in the reference (250 μM of test compound in CH_3_CN) and multiplying by the target concentration and dilution factor.

#### Plasma Stability Assay

10 mM DMSO stock solution of test compound (**39** or **50**) was diluted 50-fold with DMSO/H_2_O (1:1) and incubated at 37 °C for 2 h with mouse or rat plasma added with 5% DMSO (preheated at 37 °C for 10 min). The final compound’s concentration was 2.0 μM. At each time point (0, 5, 15, 30, 60, and 120 min), 50 μL of incubation mixture was diluted with 150 μL cold CH_3_CN spiked with 200 nM of warfarin, as the internal standard, followed by centrifugation at 3.270*g* for 20 min. The supernatant was further diluted with H_2_O (1:1) and analyzed by LC–MS/MS on a Waters ACQUITY UPLC-MS TQD system consisting of a triple quadrupole detector (TQD) mass spectrometer equipped with an electrospray ionization interface and a photodiode array eλ detector (PDA) from Waters Inc. (Milford, MA, USA). Electrospray ionization was applied in positive mode. Compound-dependent parameters as MRM transitions and collision energy were developed for each compound. The analyses were run on an ACQUITY UPLC BEH C_18_ (50 × 2.1 mm ID, particle size 1.7 μm) with a VanGuard BEH C_18_ precolumn (5 × 2.1 mm ID, particle size 1.7 μm) at 40 °C, using H_2_O + 0.1% HCOOH (A) and CH_3_CN + 0.1% HCOOH (B) as the mobile phase. The percentage of the test compound remaining at each time point relative to *t* = 0 was calculated by the response factor on the basis of the internal standard peak area. The percentage of test compound versus time was plotted and fitted by GraphPad Prism (GraphPad Software, Version 5 for Windows, CA, USA, www.graphpad.com) to estimate the compound’s half-life (t_1/2_), which was reported as mean value along with the standard deviation (*n* = 3).

#### Liver Microsomal Stability Assay

Pooled CD1 mouse (M1000, male), IGS Sprague-Dawley rat (R1000, male), and human (H1000, male) liver microsomes were purchased from SEKISUI XenoTech. 10 mM DMSO stock solution of the test compound (**39** or **50**) was preincubated at 37 °C for 15 min with mouse, rat, or human liver microsomes in 0.1 M Tris-HCl buffer (pH 7.5) with 10% DMSO. The final concentration was 4.6 μM. After preincubation, the cofactor (NADPH) was added to the incubation mixture, and the incubation was continued at 37 °C for 1 h. At each time point (0, 5, 15, 30, and 60 min), 30 μL of incubation mixture was diluted with 200 μL cold CH_3_CN spiked with 200 nM of an appropriate internal standard, followed by centrifugation at 3.270*g* for 15 min. The supernatant was further diluted with H_2_O (1:1) for analysis. A reference incubation mixture of each specie (microsomes without cofactors) was prepared for each test compound and analyzed at *t* = 0 and 60 min in order to verify the compound stability in the matrix. The two time points were diluted as for the time points of the incubation mixture above. The supernatants were analyzed by LC–MS/MS on a Waters ACQUITY UPLC-MS TQD system consisting of a triple quadrupole detector (TQD) mass spectrometer equipped with an electrospray ionization interface and a photodiode array eλ detector (PDA) from Waters Inc. (Milford, MA, USA). Electrospray ionization was applied in positive mode. Compound-dependent parameters as MRM transitions and collision energy were developed for each compound. The analyses were run on an ACQUITY UPLC BEH C_18_ (50 × 2.1 mm ID, particle size 1.7 μm) with a VanGuard BEH C_18_ precolumn (5 × 2.1 mm ID, particle size 1.7 μm) at 40 °C, using H_2_O + 0.1% HCOOH (A) and CH_3_CN + 0.1% HCOOH (B) as the mobile phase. The percentage of test compound remaining at each time point relative to *t* = 0 was calculated by the response factor on the basis of the internal standard peak area. The percentage of test compound versus time was plotted and fitted by GraphPad Prism (GraphPad Software, Version 5 for Windows, CA, USA, www.graphpad.com) to estimate the compound’s half-life (*t*_1/2_), which was reported as mean value along with the standard deviation (*n* = 3).

### In Vivo Pharmacology

#### Animals

Male C57BL/6 mice, weighing 22–24 g, were used (Charles River). All procedures were performed in accordance with the Ethical Guidelines of European Communities Council (Directive 2010/63/EU of 22 September 2010) and accepted by the Italian Ministry of Health. All efforts were made to minimize animal suffering and to use the minimal number of animals required to produce reliable results, according to the “3Rs concept”. Animals were group-housed in ventilated cages and had free access to food and water. They were maintained under a 12 h light/dark cycle (lights on at 8:00 am) at controlled temperature (21 ± 1 °C) and relative humidity (55 ± 10%).

#### Pharmacokinetics Methods

Compound **39** or **50** was administered p.o. and i.v. to C57BL/6 male mice at 10 and 3 mg/kg. The vehicle used was PEG400/Tween 80/saline solution at 10/10/80% in volume, respectively. Three animals per each time point were treated. Blood samples at 0, 15, 30, 60, 120, 240, and 480 min after administration were collected for the p.o. arm. Blood samples at 0, 5, 15, 30, 60, 120, and 240 min after administration were collected for the i.v. arm. Plasma was separated from blood by centrifugation for 15 min at 1500 rpm a 4 °C, transferred to Eppendorf tubes, and frozen (−80 °C). Control animals treated with vehicle only were also included in the experimental protocol.

Plasma samples were centrifuged at 21,100*g* for 15 min at 4 °C. An aliquot of each sample was extracted (1:3) with cold CH_3_CN containing 200 nM of an appropriate internal standard as a close analogue of the parent compound (for compound **39**: (1*R*,3*r*,5*S*)-3-(4-phenylphenoxy)-8-((3,5-dimethyl-1*H*-pyrazol-4-yl)sulfonyl)-8 azabicyclo[3.2.1]octane; for compound **50**: (1*R*,3*r*,5*S*)-3-((5-methylpyrazin-2-yl)oxy)-8-((3,5-dimethyl-1*H*-pyrazol-4-yl)sulfonyl)-8-azabicyclo[3.2.1]octane). A calibration curve was obtained in blank mouse plasma over a 1 nM to 10 μM range. Three quality controls were prepared by spiking the parent compound in blank mouse plasma to 20, 200, and 2.000 nM, as final concentrations. The calibrators and quality controls were extracted (1:3) with the same extraction solution as the plasma sample. The plasma samples, the calibrators, and quality controls were centrifuged at 3.270*g* for 15 min at 4 °C. The supernatants were further diluted (1:1) with H_2_O + 0.1% HCOOH, and analyzed by LC–MS/MS on a Waters ACQUITY UPLC-MS TQD system consisting of a Triple Quadrupole Detector (TQD) Mass Spectrometer equipped with an Electrospray Ionization interface and a Photodiode Array eλ Detector from Waters Inc. (Milford, MA, USA). Electrospray ionization was applied in positive mode. Compound-dependent parameters such as MRM transitions and collision energy were developed for the parent compound and the internal standard. The analyses were run on an ACQUITY UPLC BEH C_18_ (50 × 2.1 mm ID, particle size 1.7 μm) with a VanGuard BEH C_18_ precolumn (5 × 2.1 mm ID, particle size 1.7 μm) at 40 °C. The mobile phase was H_2_O + 0.1% HCOOH (A) and CH_3_CN + 0.1% HCOOH (B) at a flow rate = 0.5 mL/min. A linear gradient was applied starting at 10% B with an initial hold for 0.2 min, then 10–100% B in 2 min. All samples (plasma samples, calibrators, and quality controls) were quantified by the MRM peak area response factor in order to determine the levels of the parent compound in plasma. The concentrations versus time were plotted and the profiles were fitted using PK Solutions Excel Application (Summit Research Service, USA) in order to determine the pharmacokinetic parameters.

### Computational Methods (Docking Study)

Ligand docking simulations were carried out with the induced fit docking (IFD) protocol^63^ as implemented in Schrödinger 2019-3 (Schrödinger LLC, New York, USA). The coordinates of the enzyme in complex with the non-covalent inhibitor ARN19702 (Figure 1) were processed with the protein preparation routine. Default parameters were used. The coordinates of the cocrystallized ligand (PDB ID: 6DXX)^11^ were used to define the size and the position of the binding box and then the ligand was deleted from the system. In the first step of the IFD protocol, each ligand was docked scaling by a factor of 0.5 the van der Waals radii of all the protein and ligand atoms with partial charges ≤0.25. The standard Glide SP protocol was used for docking.^64,65^ Up to 20 poses were retained and progressed to the next step. The van der Waals scaling factor was removed, and each complex was refined using Prime.^66^ All residues within 5 Å from the ligand were optimized. Finally, ligands were redocked in each complex using the optimized receptor coordinates, standard van der Waals radii, and the Glide XP protocol.^67^ For each ligand, the best scoring complex was retained. All calculations were carried out using the OPLS3e force field.^68^

## Supporting Information Available

Molecular formula strings for all final compounds (CSV)
Docking model for **39** (PDB)
Docking model for **40** (PDB)
Docking model for **50** (PDB)
^1^H, ^13^C, and ^19^F NMR spectral data and UPLC-MS analyses; structure and LipE data of selected, novel *h*-NAAA inhibitors (**1**, **39**, **47–50**); structure of known *h*-NAAA inhibitors as reference compounds; docking study in the *h*-NAAA binding site; and in vivo experimental procedures and graphic of compound **50** (PDF)

## Supplementary Material

jm1c00575_si_001.csv

jm1c00575_si_002.pdb

jm1c00575_si_003.pdb

jm1c00575_si_004.pdb

## Abbreviations

ABPP: activity-based protein profiling
AC: acid ceramidase
9-BBN: 9-borabicyclo(3.3.1)nonane
BSA: bovine serum albumin
DCM: dichloromethane
DIAD: diisopropyl azodicarboxylate
DIPEA: *N*,*N*-diisopropylethylamine
ECFP4: extended connectivity finger-print 4
FAAH: fatty acid amide hydrolase
FBS: fetal bovine serum
HBTU: O-(benzotriazol-1-yl)-*N*,*N*,*N*′,*N*′-tetramethyluronium tetrafluoroborate
NAAA: *N*-acylethanolamine-hydrolyzing acid amidase
NADPH: nicotinamide adenine dinucleotide phosphate (reduced)
PBS: phosphate-buffered saline
PEA: palmitoylethanolamide
SDS-PAGE: sodium dodecyl sulfate-polyacrylamide gel electrophoresis
TEA: triethylamine
TFA: trifluoroacetic acid
