# Supplementary material for: Discovery and SAR Evolution of Pyrazole Azabicyclo[3.2.1]octane Sulfonamides as a Novel Class of Non-Covalent N-Acylethanolamine-Hydrolyzing Acid Amidase (NAAA) Inhibitors for Oral Administration
Source: J Med Chem. 2021 Sep 1;64(18):13327–55. doi: 10.1021/acs.jmedchem.1c00575 (PMC8474119; doi:10.1021/acs.jmedchem.1c00575)
Supplement: Supplementary file 5 — jm1c00575_si_005.pdf [file jm1c00575_si_005.pdf]

SUPPORTING INFORMATION

Discovery and SAR Evolution of Pyrazole Azabicyclo[3.2.1]octane Sulfonamides as a Novel Class of Non-Covalent N-Acylethanolamine-hydrolyzing Acid Amidase (NAAA) Inhibitors for Oral Administration.

Paolo Di Fruscia,[1] Anna Carbone,[1,2] Giovanni Bottegoni,[3] Francesco Berti,[1] Francesca Giacomina,[1] Stefano Ponzano,[1] Chiara Pagliuca,[1] Annalisa Fiasella,[1] Daniela Pizzirani,[1] Jose Antonio Ortega,[1] Andrea Nuzzi,[1] Glauco Tarozzo,[1] Luisa Mengatto,[1] Roberta Giampà,[1] Ilaria Penna,[1] Debora Russo,[1] Elisa Romeo,[4] Maria Summa,[5] Rosalia Bertorelli,[5] Andrea Armirotti,[5] Sine Mandrup Bertozzi,[5] Angelo Reggiani,[4] Tiziano Bandiera,[1] Fabio Bertozzi[1,]*

[1]D3-PharmaChemistry, Istituto Italiano di Tecnologia (IIT), 16163 - Genova, Italy; [2]Department of Biological, Chemical and Pharmaceutical Sciences and Technologies (STEBICEF), University of Palermo, 90123 - Palermo, Italy; [3]D3-Computational Modelling of Nanoscale and Biophysical Systems, Istituto Italiano di Tecnologia (IIT), 16163 - Genova, Italy; [4]D3-Validation, Istituto Italiano di Tecnologia (IIT), 16163 - Genova, Italy; [5]Analytical Chemistry and Translational Pharmacology, Istituto Italiano di Tecnologia (IIT), 16163 - Genova, Italy.

Corresponding author's email address: fabio.bertozzi@iit.it

Table of contents

Experimental Part S3

- *1. ^{1}H, ^{13}C, ^{19}F NMR spectra of final compounds* ***1-50*** S3-S56
- *2. LC-MS analyses of final compounds* ***1-50*** S57-S82
- *3. Structure and LipE data of selected, novel h-NAAA inhibitors* S83
- *4. Known h-NAAA inhibitors as reference compounds* S84
- *5. Docking study of compound* ***50*** *in human NAAA binding site* S85
- *6. In-vivo characterization of compound* ***50*** S86
- 7. *References* S87

Experimental part

1. ^{1}H, ^{13}C, ^{19}F NMR Spectra of final compounds 1-50

NMR experiments were run at 300 K on a Bruker Avance III 400 system (400.13 MHz for ^{1}H, and 100.62 MHz for ^{13}C), equipped with a BBI probe and Z-gradients, and Bruker FT NMR Avance III 600 MHz spectrometer equipped with a 5 mm CryoProbeTM QCI ^{1}H/^{19}F–^{13}C/^{15}N–D quadruple resonance, a shielded z-gradient coil and the automatic sample changer SampleJet™ NMR system (600 MHz for ^{1}H, 151 MHz for ^{13}C and 565 MHz for ^{19}F). Chemical shifts for ^{1}H and ^{13}C spectra were reported in parts per million (ppm), calibrating the residual non-deuterated solvent peak for the ^{1}H and ^{13}C, respectively to 7.26 ppm and 77.16 ppm for $CDCl_3$ and 2.50 ppm and 39.52 ppm for DMSO-d_6.

Quantitative ^{1}H NMR

Purity of the final compounds was determined by UPLC-MS and quantitative ^{1}H NMR (qNMR). qNMR experiments were acquired with 64 transients, after an automatic 90° degree pulse length optimization,[1] by using 65536 digit points, 30 s of interpulses delay, and the receiver gain fixed (64), the spectral width was 22.55 ppm with the offset positioned at 6.17 ppm. An apodization exponential function equivalent to 0.3Hz was applied to FIDs before Fourier transform. Spectra were phased, and baseline corrected, automatically. For purity evaluation by NMR assay (qNMR), the signal of final compound (10 mM solution in DMSO-d_6), was compared to the peak of an equimolar external standard solution of maleic acid (TraceCERT, 99.99%, Sigma-Aldrich, Milan, Italy), after the normalization for the number of protons generating such signals, by using the PULCON method.[2]

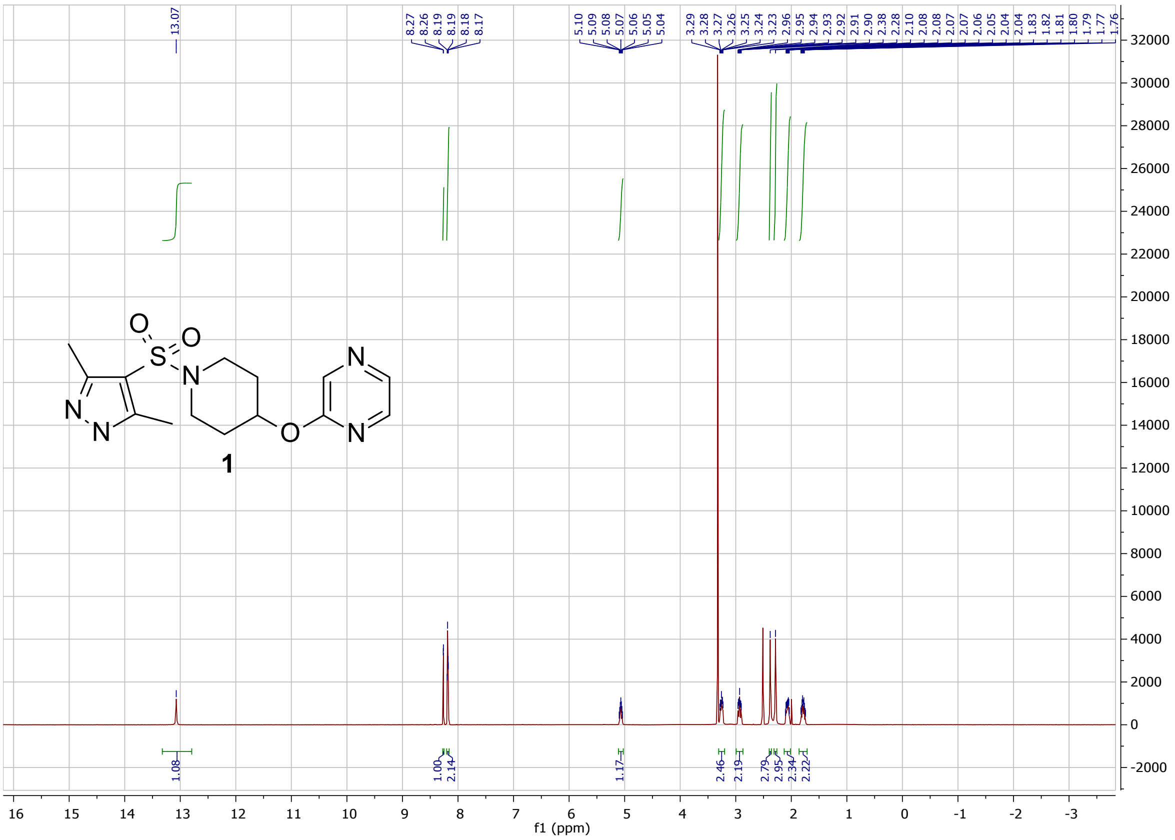
13.07
8.27 8.26 8.19 8.19 8.18 8.17
5.10 5.09 5.08 5.07 5.06 5.05 5.04
3.29 3.28 3.27 3.26 3.25 3.24 3.23 2.96 2.95 2.94 2.93 2.92 2.91 2.90 2.38 2.28 2.10 2.08 2.08 2.07 2.07 2.06 2.05 2.04 2.04 1.83 1.82 1.80 1.79 1.77 1.76
1
1.08
1.00 2.14
1.17
2.46 2.19 2.79 2.95 2.34 2.22
f1 (ppm)

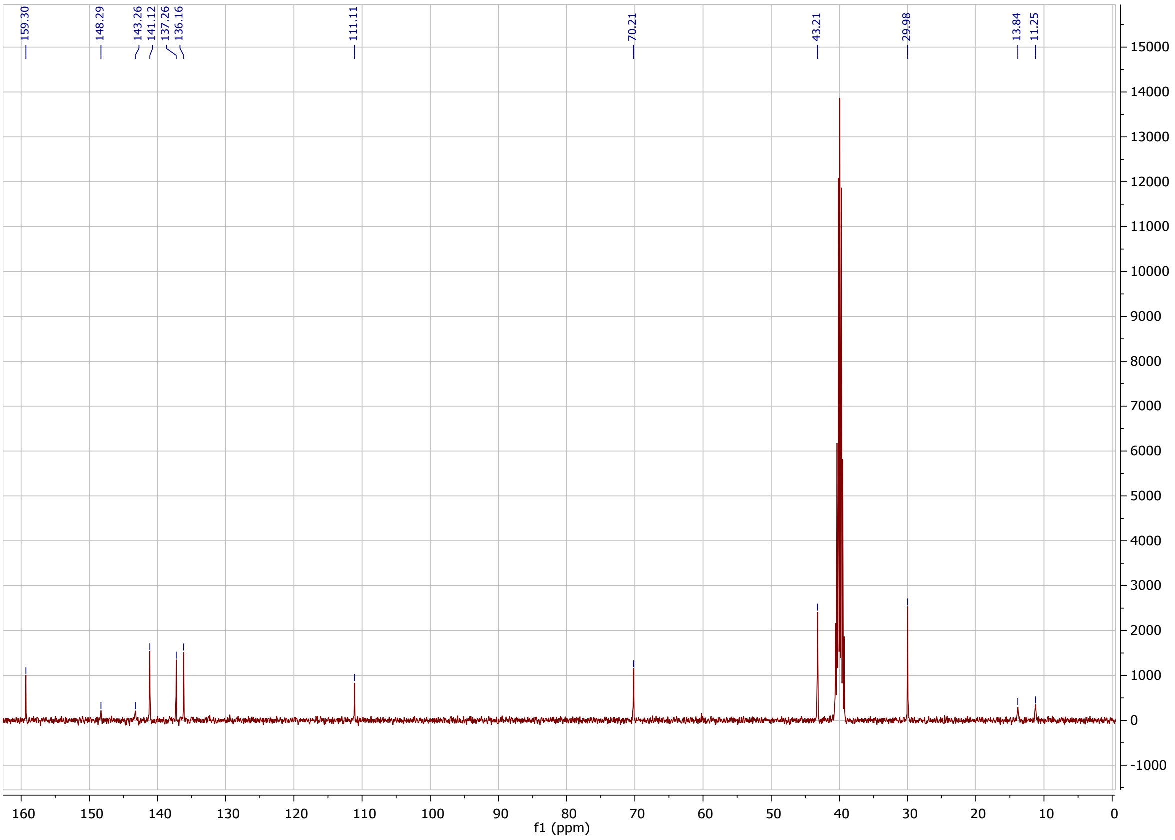
159.30
148.29
143.26 141.12 137.26 136.16
111.11
70.21
43.21
29.98
13.84 11.25
f1 (ppm)

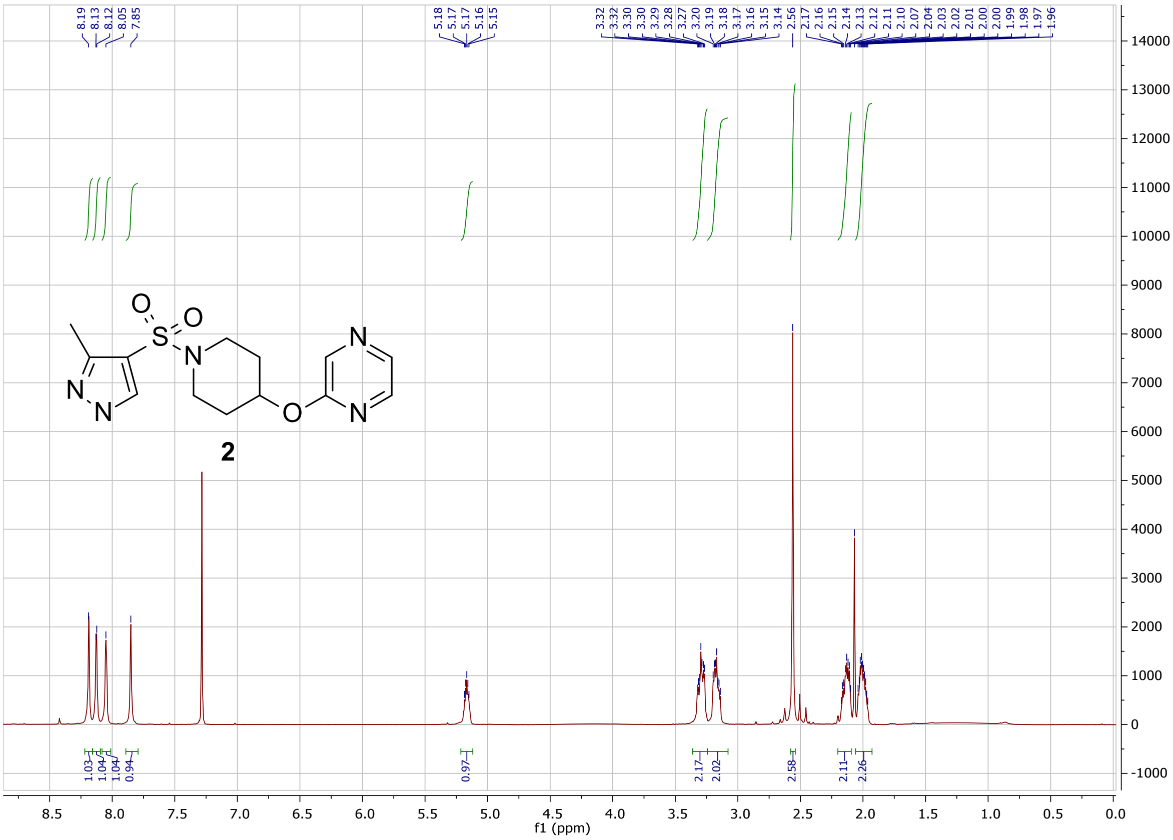
8.19 8.13 8.12 8.05 7.85
5.18 5.17 5.17 5.16 5.15
3.32 3.32 3.30 3.30 3.29 3.28 3.27 3.20 3.19 3.18 3.17 3.16 3.15 3.14 2.56 2.17 2.16 2.15 2.14 2.13 2.12 2.11 2.10 2.07 2.04 2.03 2.02 2.01 2.00 2.00 1.99 1.98 1.97 1.96
2
1.03 1.04 1.04 0.94 0.97 2.17 2.02 2.58 2.11 2.26
f1 (ppm)

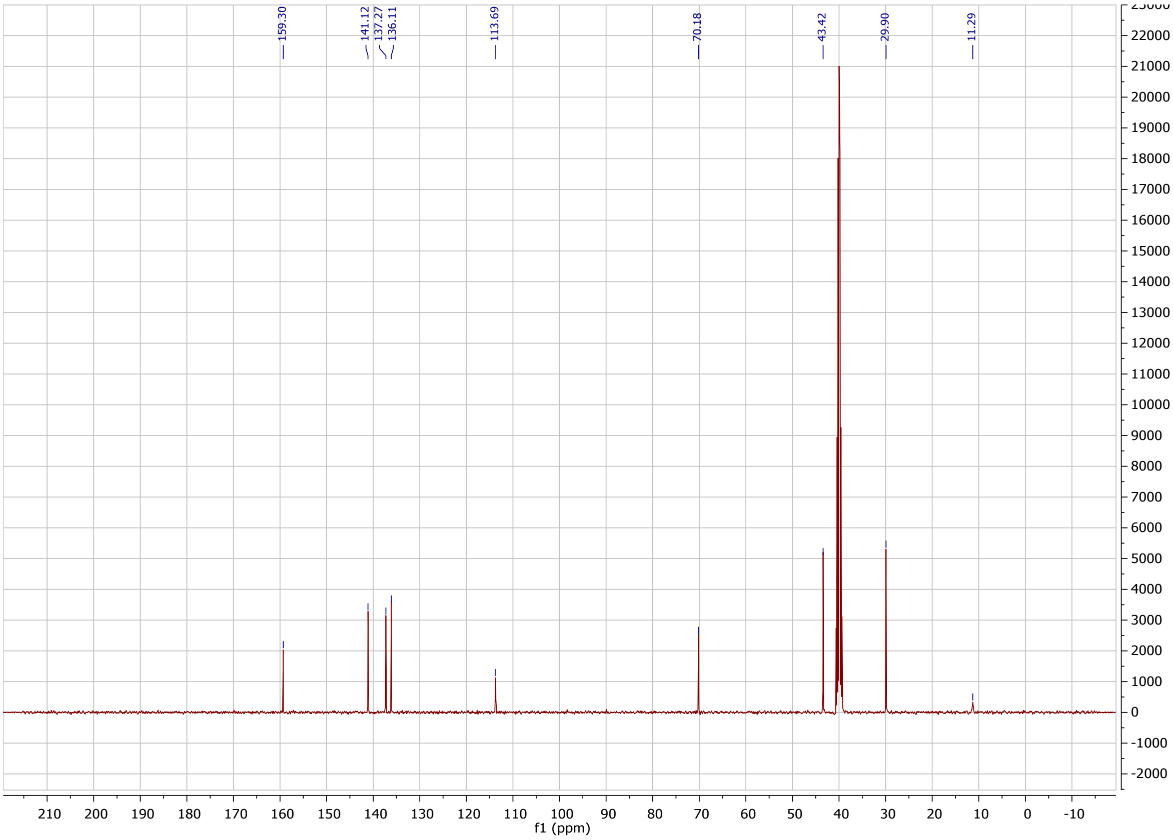
159.30
141.12 137.27 136.11
113.69
70.18
43.42
29.90
11.29
f1 (ppm)

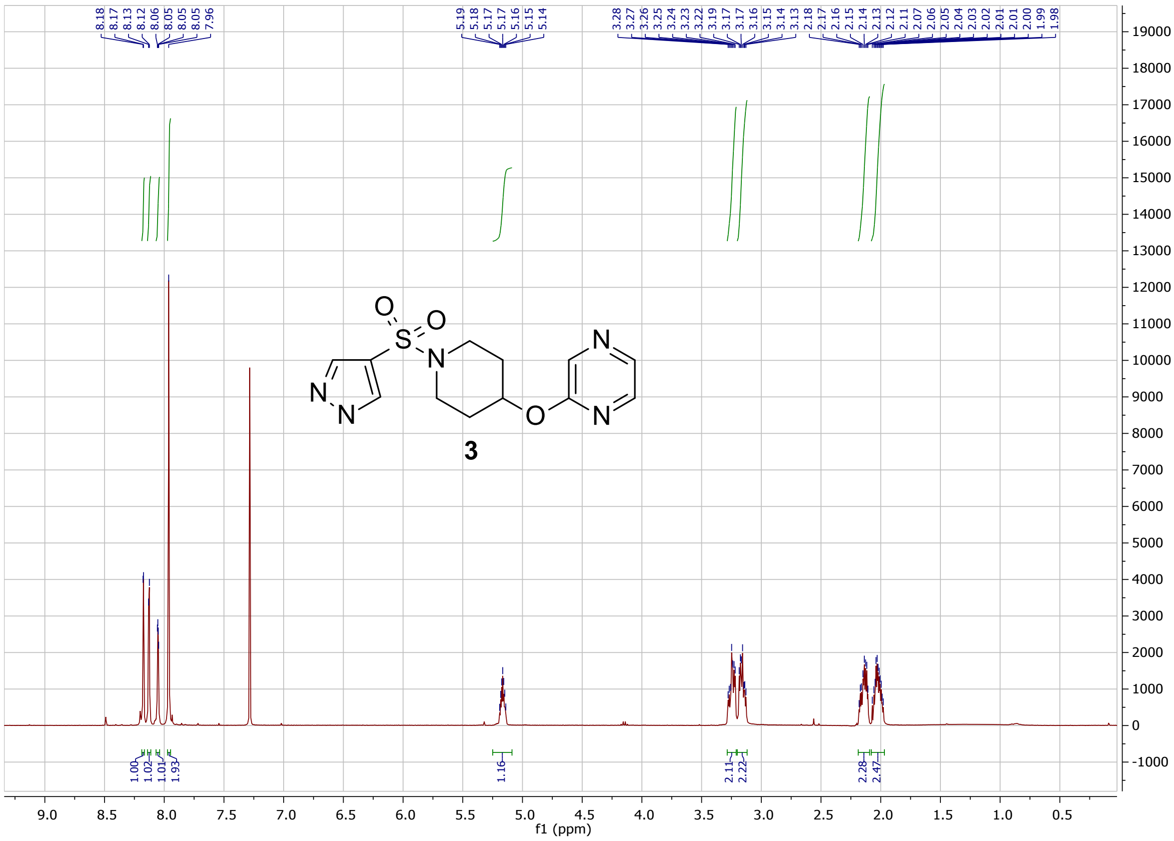
8.18
8.17
8.13
8.12
8.06
8.05
8.05
8.05
7.96
5.19
5.18
5.17
5.17
5.16
5.15
5.14
3.28
3.27
3.26
3.25
3.24
3.23
3.22
3.19
3.17
3.17
3.16
3.15
3.14
3.13
2.18
2.17
2.16
2.15
2.14
2.13
2.12
2.11
2.07
2.06
2.05
2.04
2.03
2.02
2.01
2.01
2.00
1.99
1.98
3
1.00
1.02
1.01
1.93
1.16
2.11
2.22
2.28
2.47
f1 (ppm)

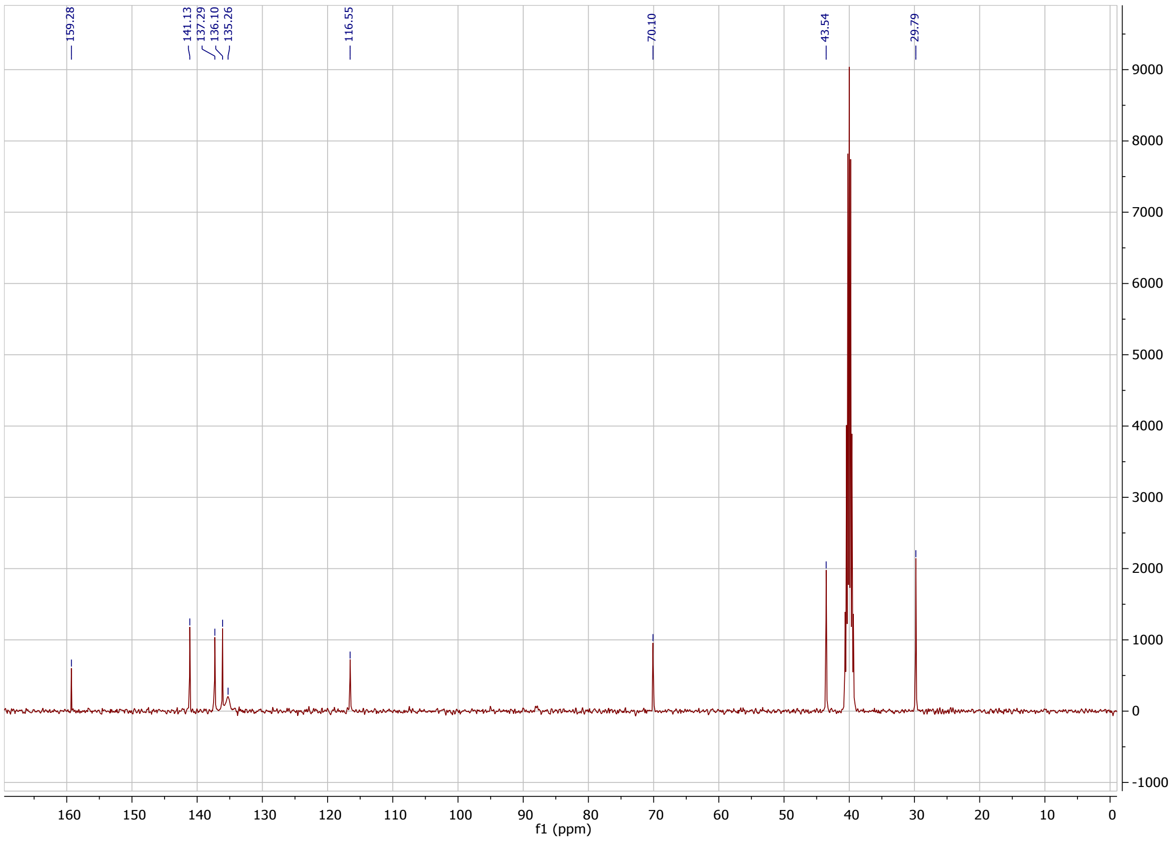
159.28
141.13
137.29
136.10
135.26
116.55
70.10
43.54
29.79
f1 (ppm)

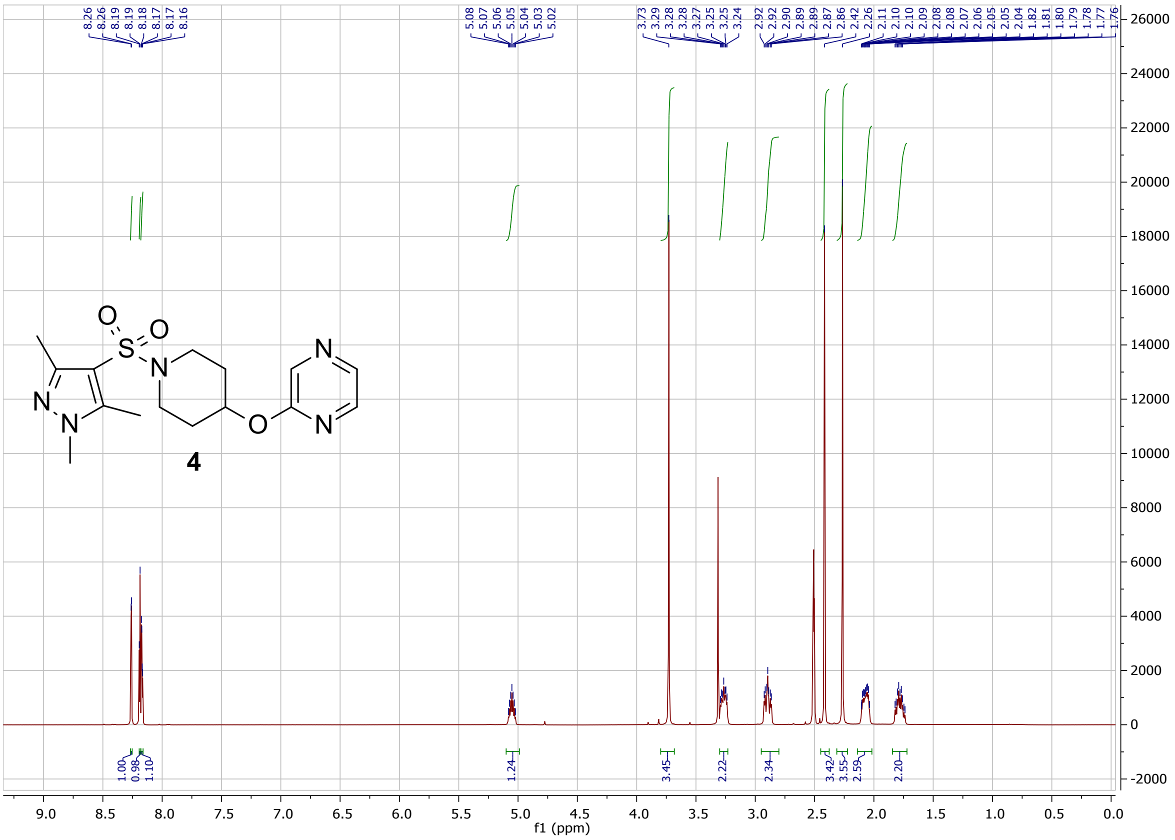
8.26 8.26 8.19 8.19 8.19 8.18 8.17 8.17 8.16
5.08 5.07 5.06 5.05 5.04 5.03 5.02
3.73 3.29 3.28 3.28 3.27 3.25 3.25 3.24
2.92 2.92 2.90 2.89 2.89 2.87 2.86 2.42 2.26 2.11 2.10 2.10 2.09 2.08 2.08 2.07 2.06 2.05 2.05 2.04 1.82 1.81 1.80 1.79 1.78 1.77 1.76
1.00 0.98 1.10 1.24 3.45 2.22 2.34 3.42 3.55 2.59 2.20
4
f1 (ppm)

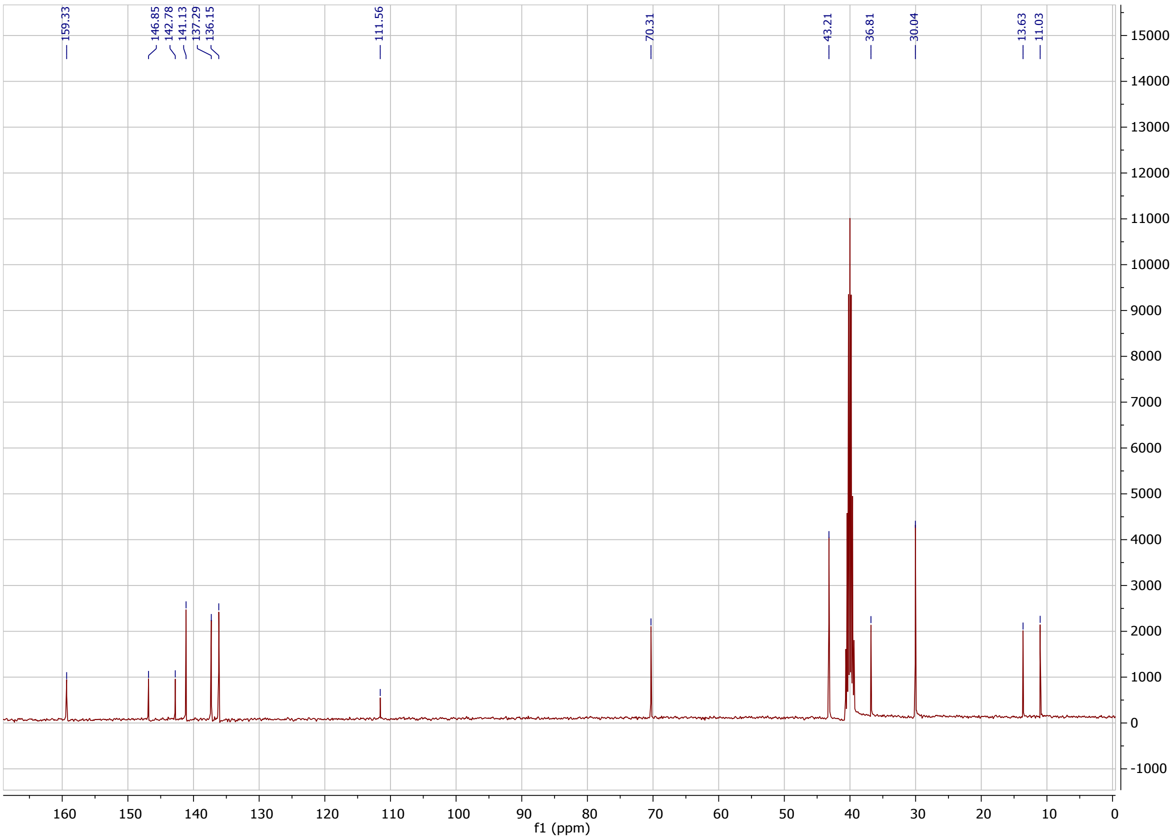
159.33
146.85 142.78 141.13 137.29 136.15
111.56
70.31
43.21
36.81
30.04
13.63
11.03
f1 (ppm)

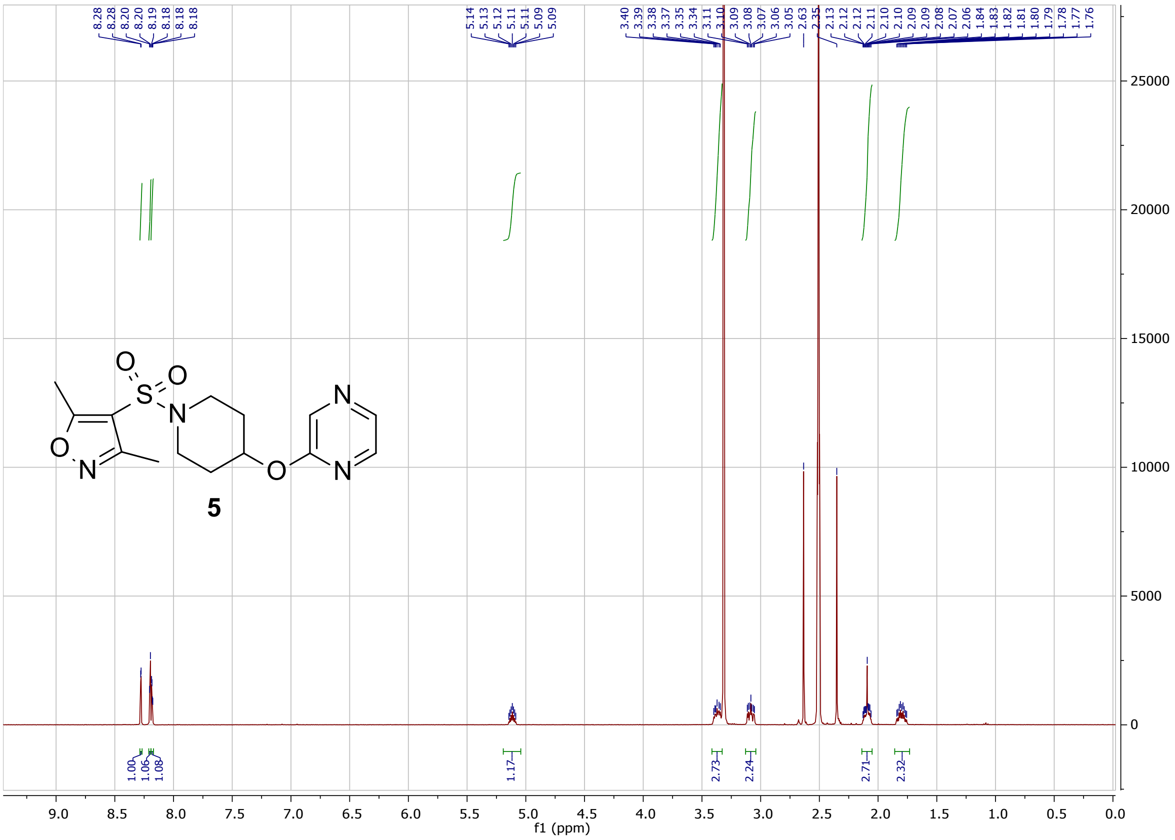
8.28 8.28 8.20 8.20 8.19 8.18 8.18 8.18
5.14 5.13 5.12 5.11 5.11 5.09 5.09
3.40 3.39 3.38 3.37 3.35 3.34 3.11 3.10 3.09 3.08 3.07 3.06 3.05 2.63 2.35 2.13 2.12 2.12 2.11 2.10 2.09 2.09 2.08 2.07 2.06 1.84 1.83 1.82 1.81 1.80 1.79 1.78 1.77 1.76
1.00 1.06 1.08 1.17 2.73 2.24 2.71 2.32
O S O N O N O N N
5
f1 (ppm)

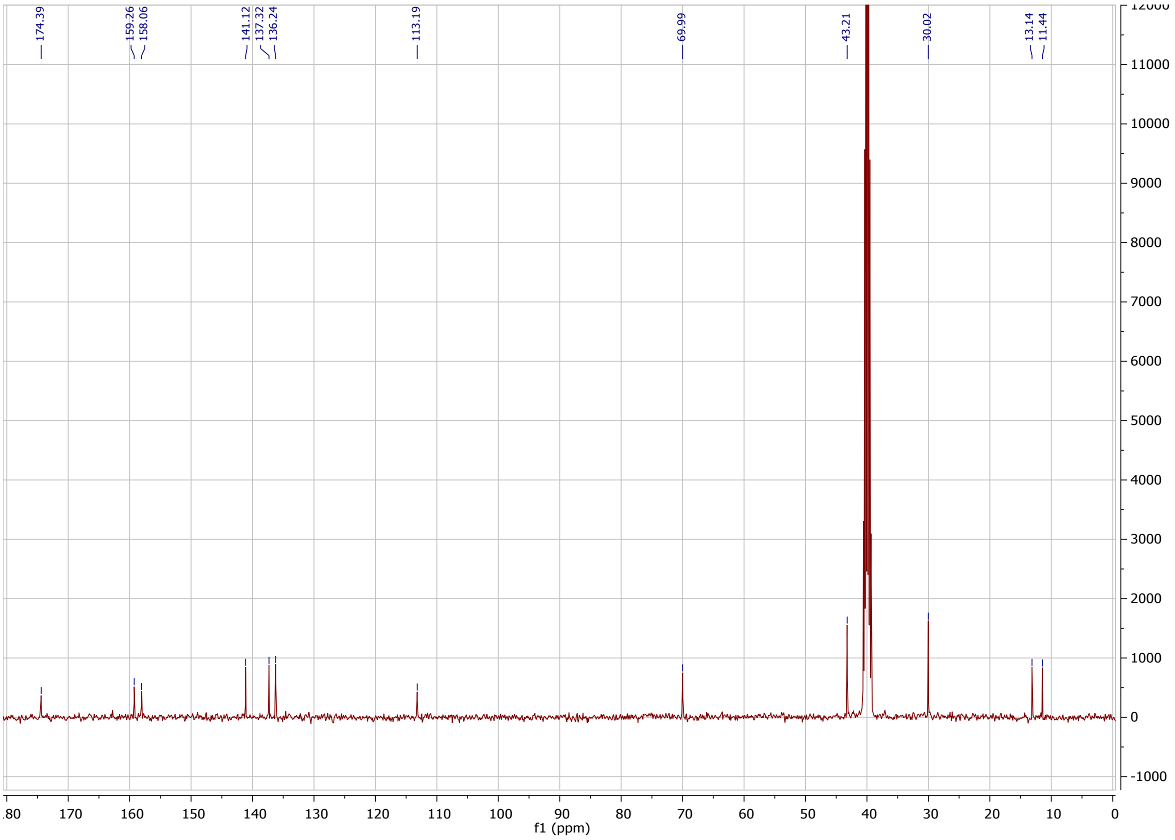
174.39
159.26 158.06
141.12 137.32 136.24
113.19
69.99
43.21
30.02
13.14 11.44
f1 (ppm)

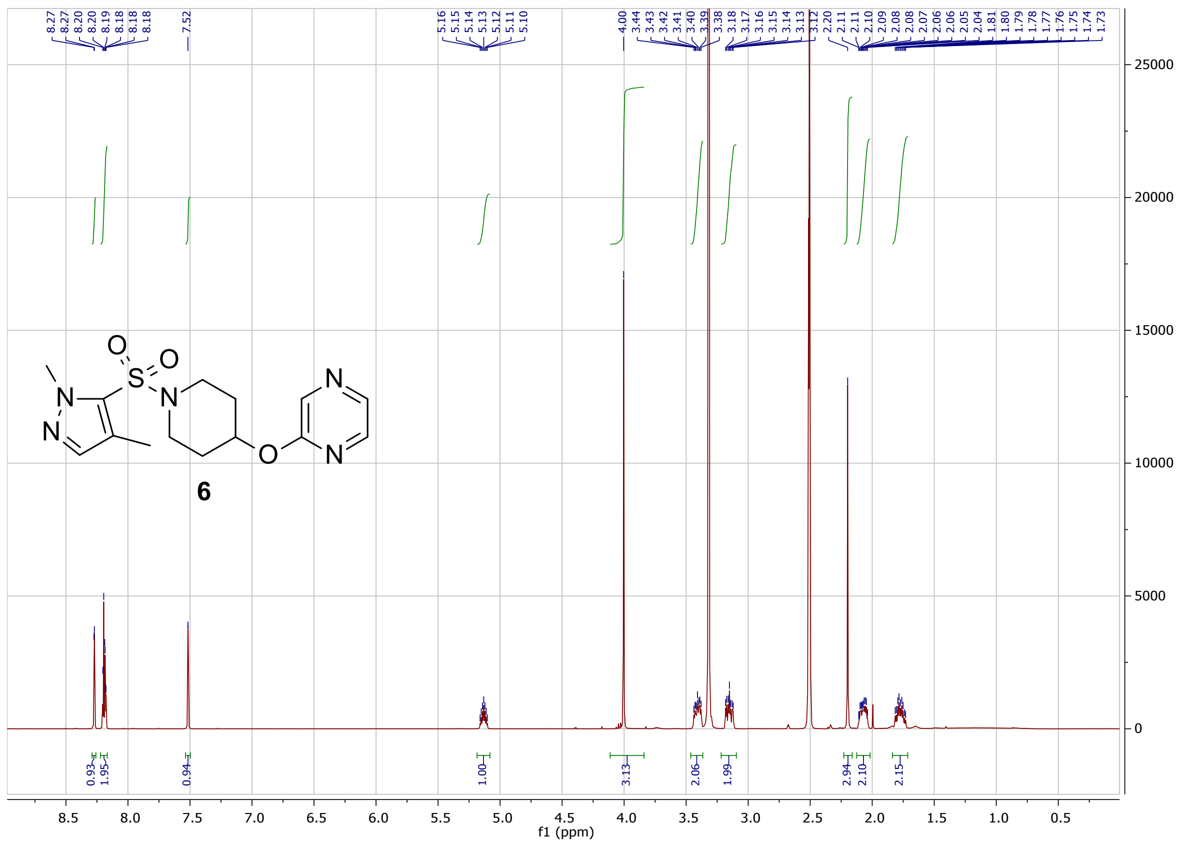
8.27
8.27
8.20
8.20
8.19
8.18
8.18
8.18
7.52
5.16
5.15
5.14
5.13
5.12
5.11
5.10
4.00
3.44
3.43
3.42
3.41
3.40
3.39
3.38
3.18
3.17
3.16
3.15
3.14
3.13
3.12
2.20
2.11
2.11
2.10
2.09
2.08
2.08
2.07
2.06
2.06
2.05
2.04
1.81
1.80
1.79
1.78
1.77
1.76
1.75
1.74
1.73
0.93
1.95
0.94
1.00
3.13
2.06
1.99
2.94
2.10
2.15
6
f1 (ppm)

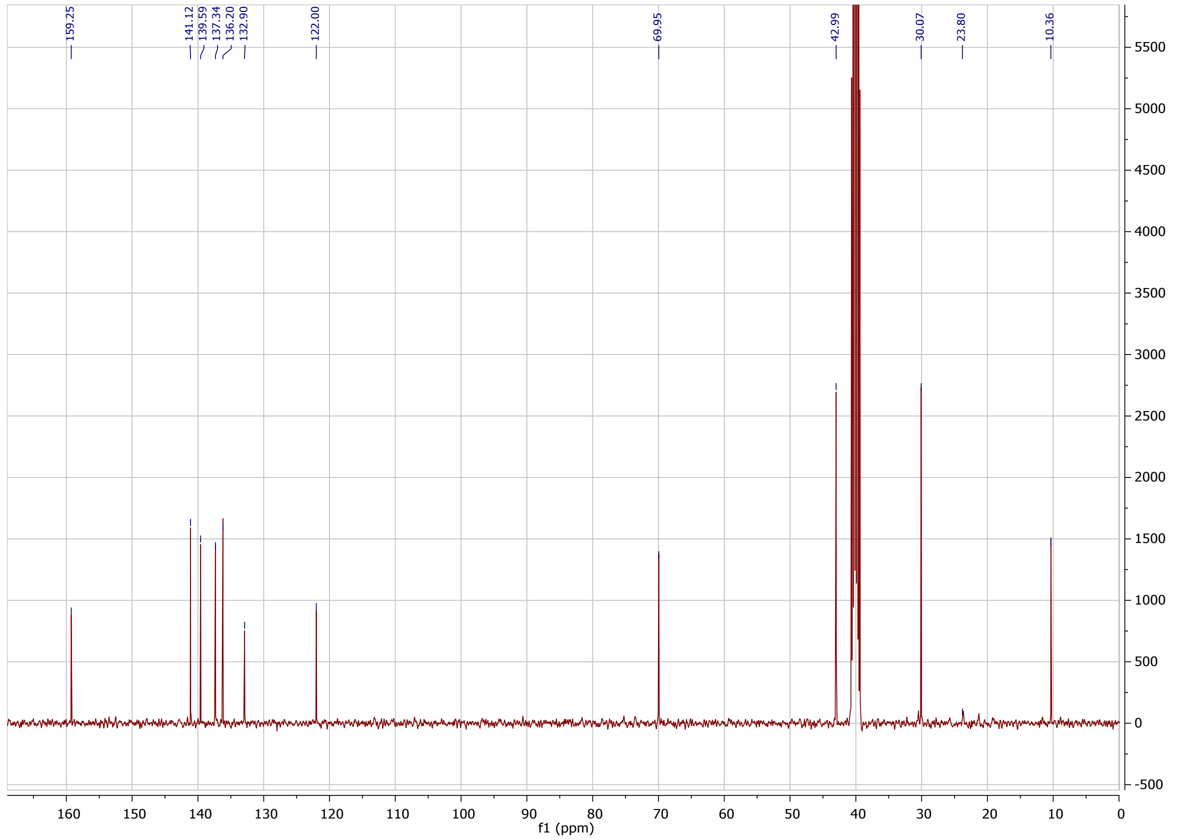
159.25
141.12
139.59
137.34
136.20
132.90
122.00
69.95
42.99
30.07
23.80
10.36
f1 (ppm)

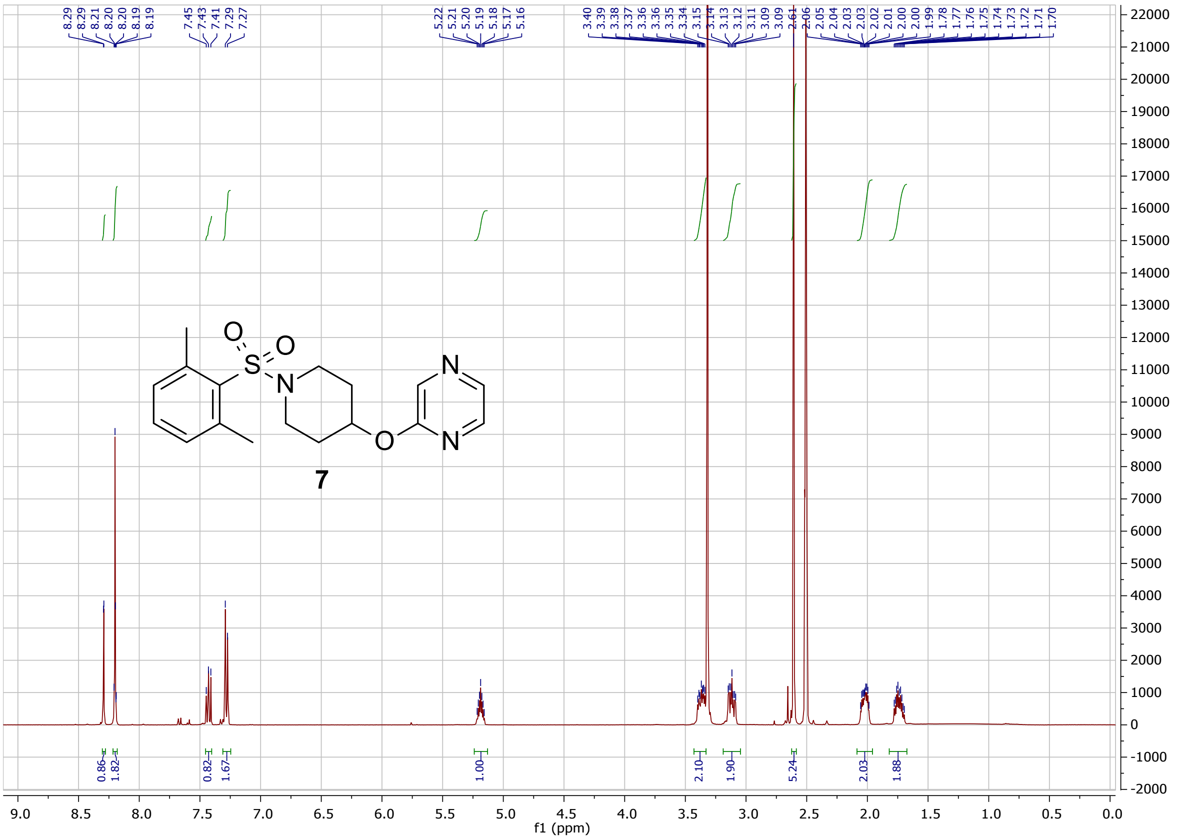
8.29 8.29 8.21 8.20 8.20 8.19 8.19
7.45 7.43 7.41 7.29 7.27
5.22 5.21 5.20 5.19 5.18 5.17 5.16
3.40 3.39 3.38 3.37 3.36 3.36 3.35 3.34 3.15 3.14 3.13 3.12 3.11 3.09 3.09 2.61 2.06 2.05 2.04 2.03 2.03 2.02 2.01 2.00 2.00 1.99 1.78 1.77 1.76 1.75 1.74 1.73 1.72 1.71 1.70
0.86 1.82 0.82 1.67 1.00 2.10 1.90 5.24 2.03 1.88
O
S
O
N
O
N
N
7
f1 (ppm)

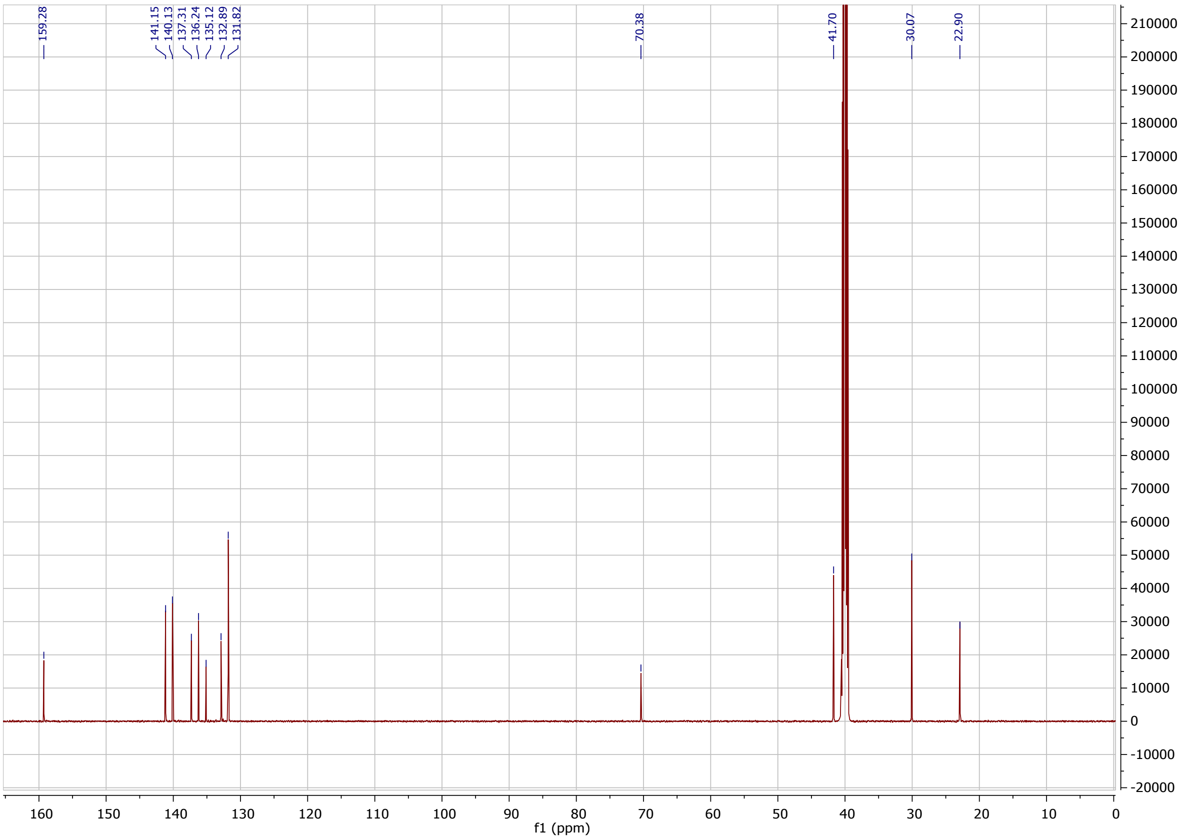
159.28
141.15 140.13 137.31 136.24 135.12 132.89 131.82
70.38
41.70
30.07
22.90
f1 (ppm)

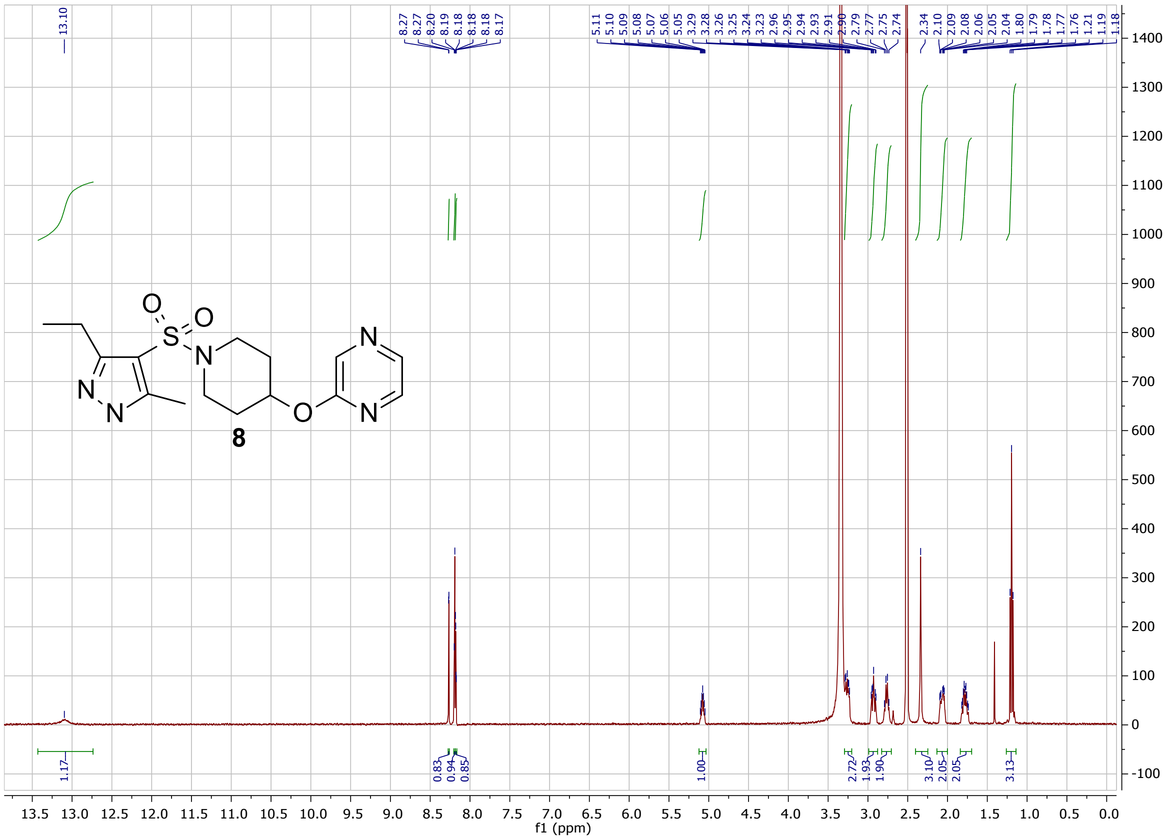

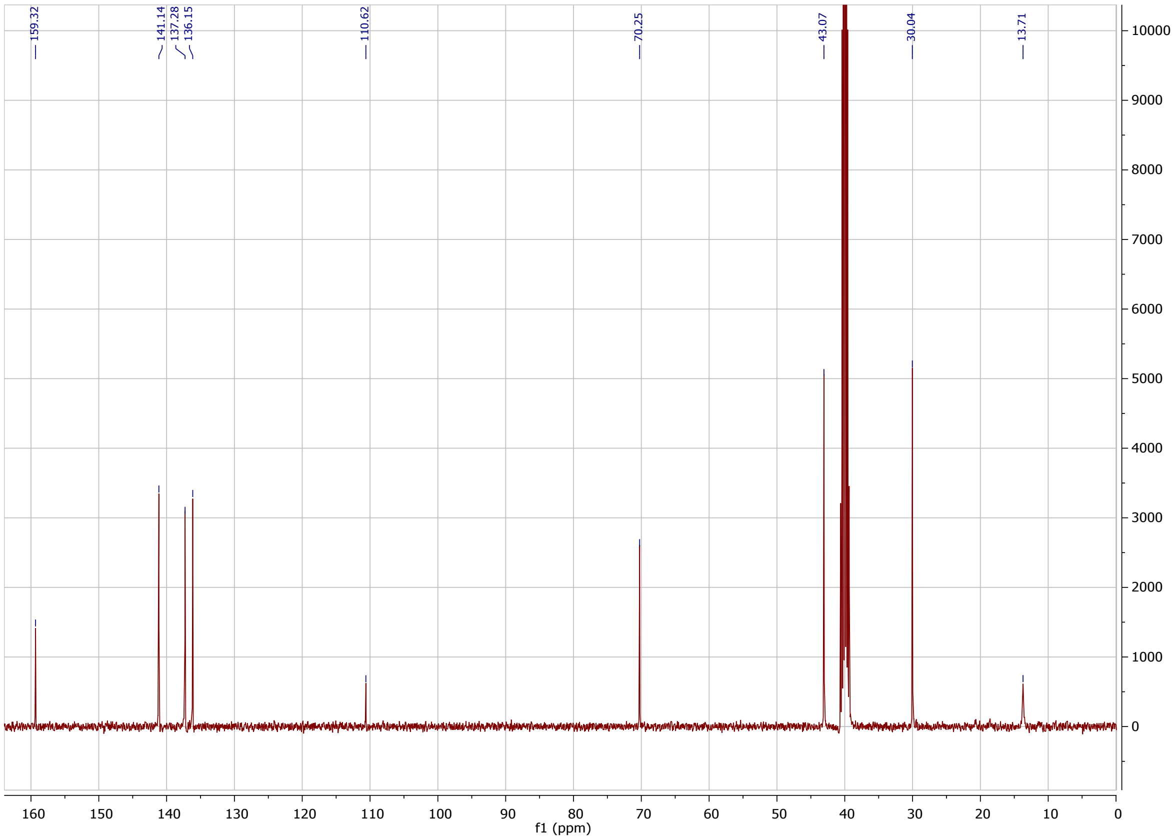

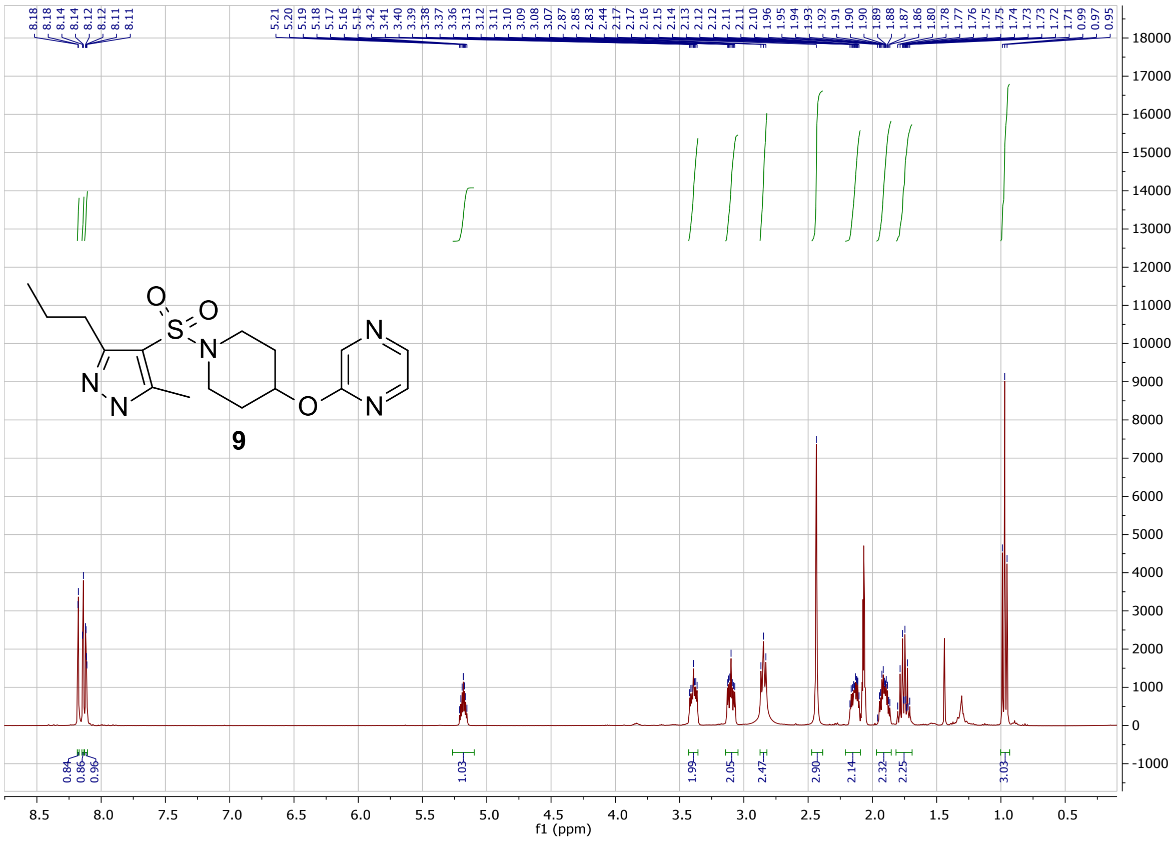
8.18
8.18
8.14
8.14
8.12
8.12
8.11
8.11
5.21
5.20
5.19
5.18
5.17
5.16
5.15
3.42
3.41
3.40
3.39
3.38
3.37
3.36
3.13
3.12
3.11
3.10
3.09
3.08
3.07
2.87
2.85
2.83
2.44
2.17
2.17
2.16
2.15
2.14
2.13
2.12
2.12
2.11
2.11
2.10
1.96
1.95
1.94
1.93
1.93
1.92
1.91
1.90
1.90
1.89
1.88
1.87
1.86
1.80
1.78
1.77
1.76
1.75
1.75
1.74
1.73
1.73
1.72
1.71
0.99
0.97
0.95
O
O
S
N
N
N
N
O
N
9
0.84
0.86
0.96
1.03
1.99
2.05
2.47
2.90
2.14
2.32
2.25
3.03
f1 (ppm)

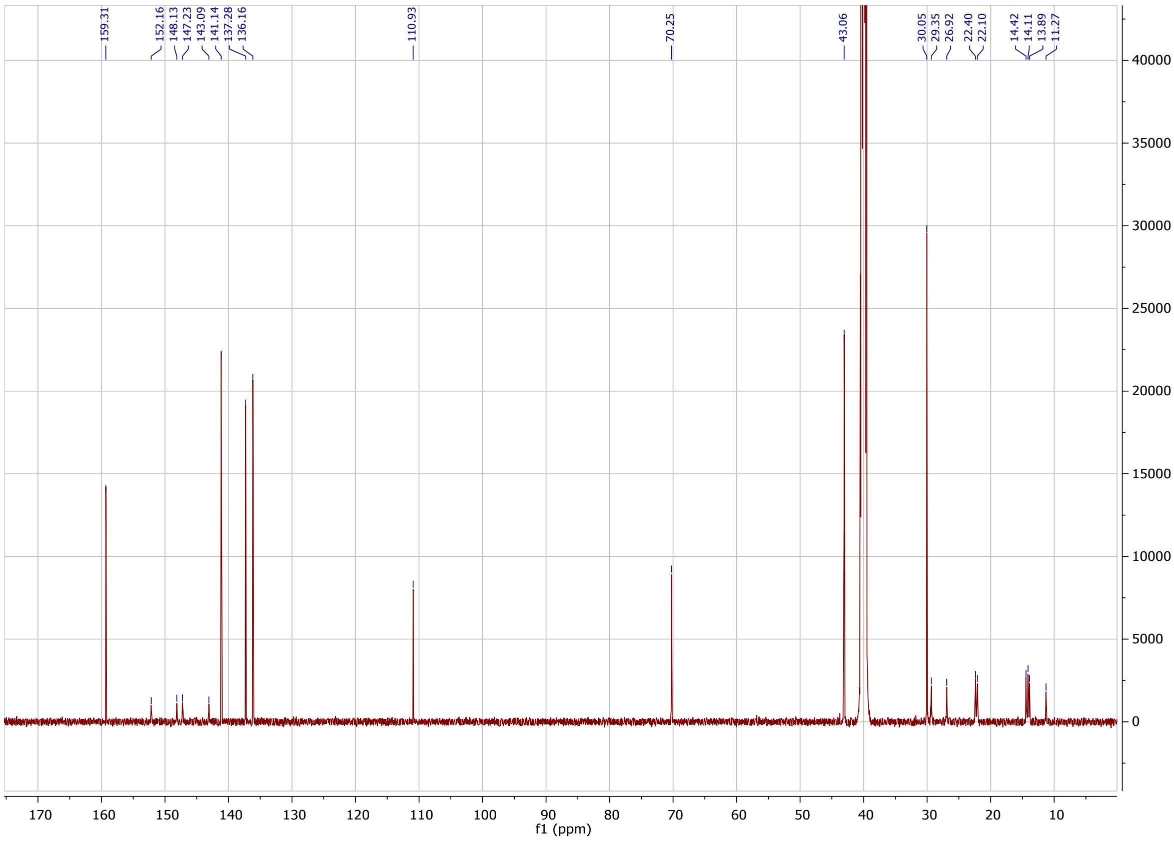
159.31
152.16
148.13
147.23
143.09
141.14
137.28
136.16
110.93
70.25
43.06
30.05
29.35
26.92
22.40
22.10
14.42
14.11
13.89
11.27
f1 (ppm)

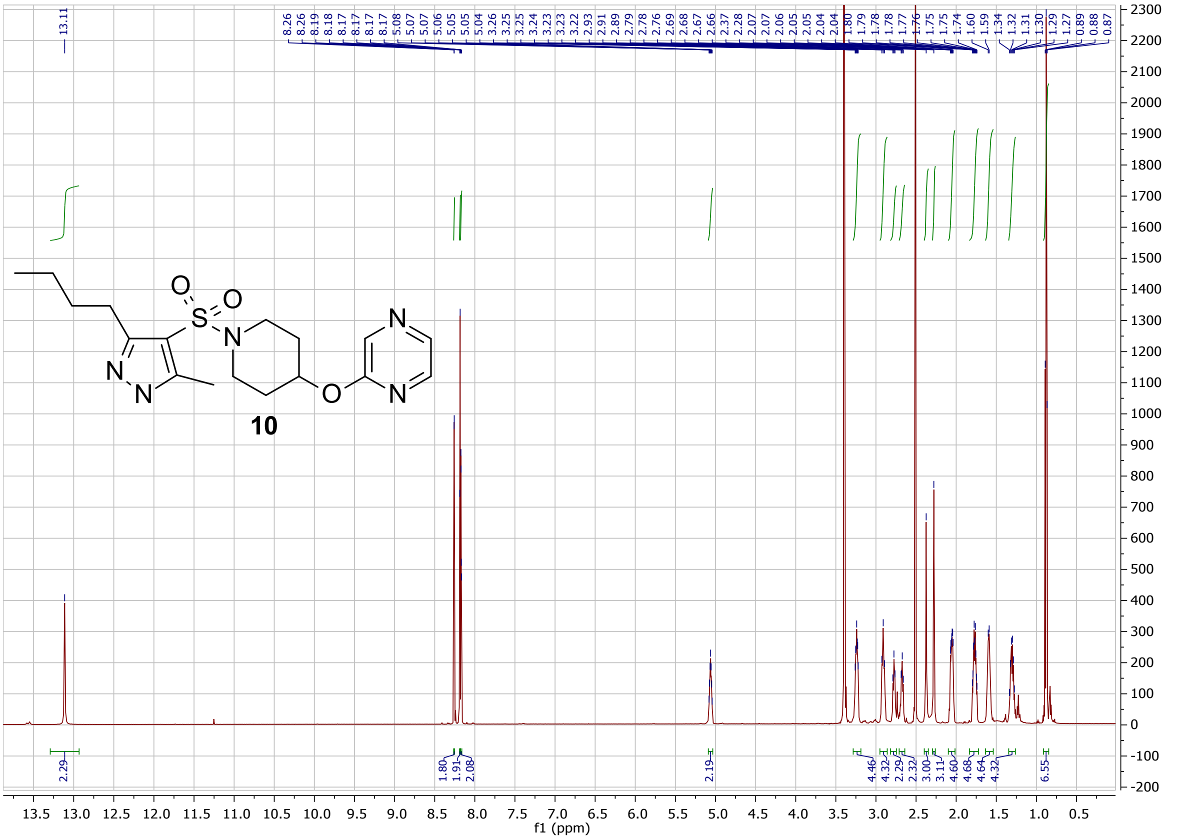
13.11
8.26 8.26 8.19 8.18 8.17 8.17 8.17 8.17 5.08 5.07 5.07 5.06 5.05 5.05 5.04 3.26 3.25 3.25 3.24 3.23 3.23 3.22 2.93 2.91 2.89 2.79 2.78 2.76 2.69 2.68 2.67 2.66 2.37 2.28 2.07 2.07 2.06 2.05 2.05 2.04 2.04 1.80 1.79 1.78 1.78 1.77 1.76 1.75 1.75 1.74 1.60 1.59 1.34 1.32 1.31 1.30 1.29 1.27 0.89 0.88 0.87
2.29 1.80 1.91 2.08 2.19 4.46 4.32 2.29 2.32 3.00 3.11 4.60 4.68 4.64 4.32 6.55
10
f1 (ppm)

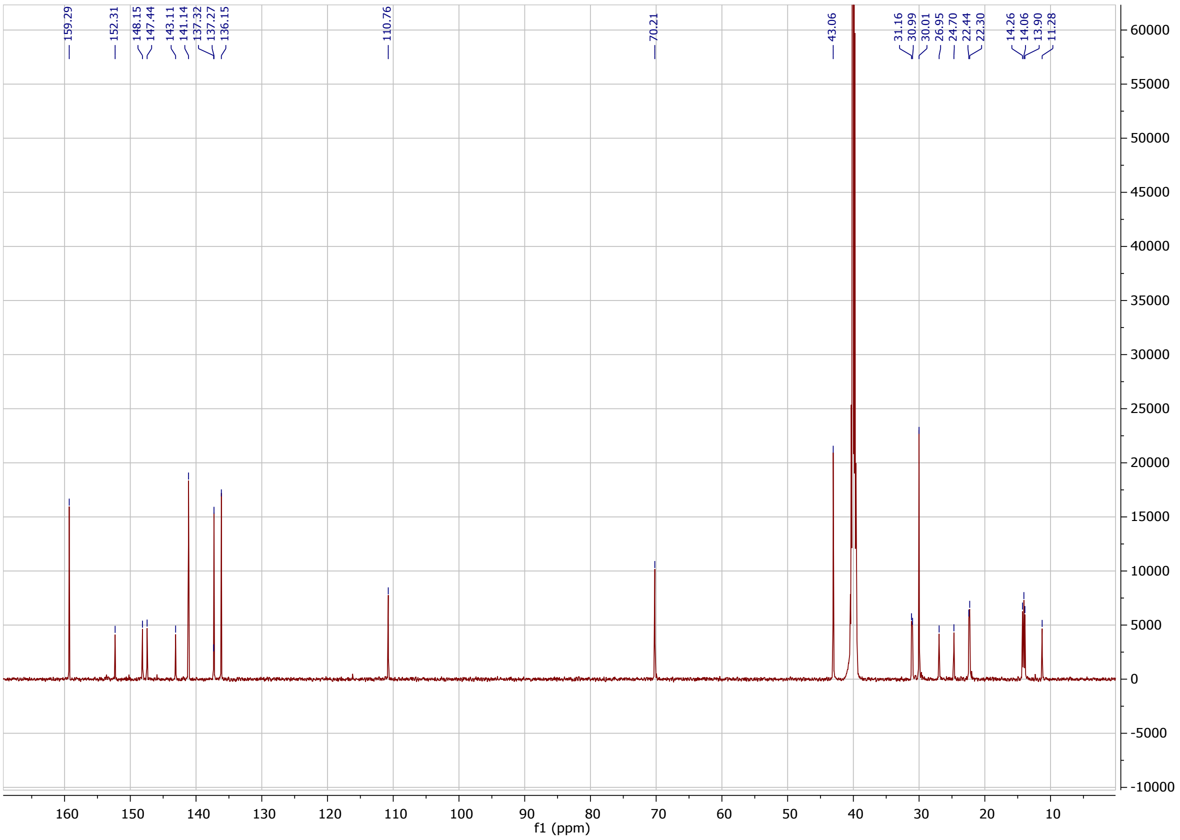
159.29 152.31 148.15 147.44 143.11 141.14 137.32 137.27 136.15 110.76 70.21 43.06 31.16 30.99 30.01 26.95 24.70 22.44 22.30 14.26 14.06 13.90 11.28
f1 (ppm)

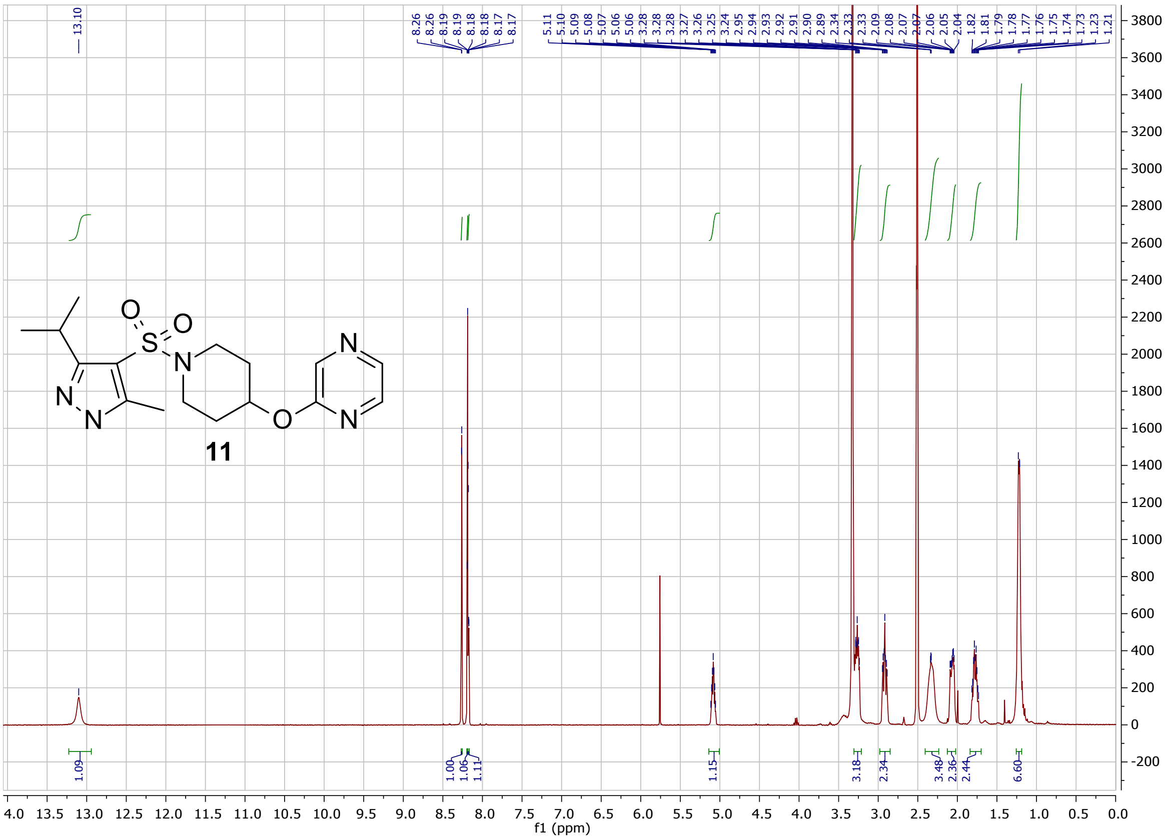
11
13.10
8.26 8.26 8.19 8.19 8.18 8.18 8.17 8.17
5.11 5.10 5.09 5.08 5.07 5.06 5.06 3.28 3.28 3.27 3.26 3.25 3.24 2.95 2.94 2.93 2.92 2.91 2.90 2.89 2.34 2.33 2.33 2.09 2.08 2.07 2.07 2.06 2.05 2.04 1.82 1.81 1.79 1.78 1.77 1.76 1.75 1.74 1.73 1.23 1.21
1.09 1.00 1.06 1.11 1.15 3.18 2.34 3.48 2.36 2.44 6.60
f1 (ppm)

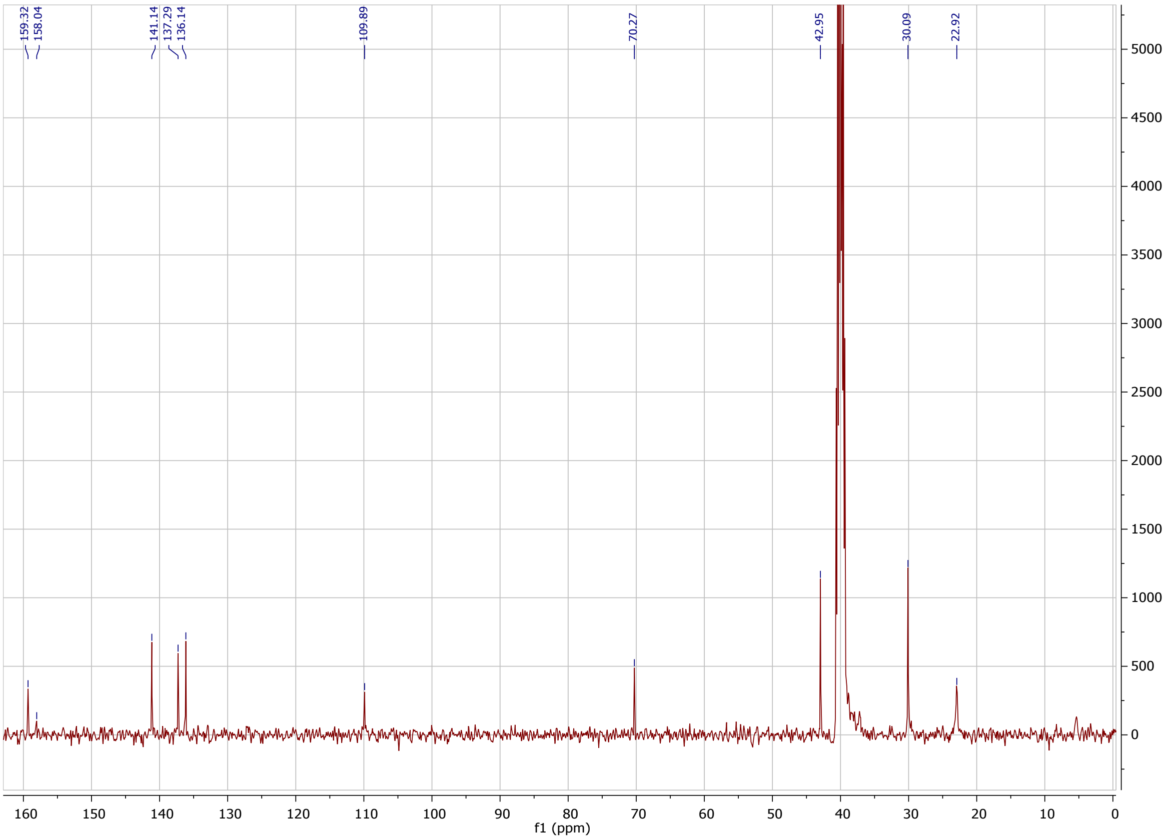
159.32 158.04 141.14 137.29 136.14 109.89 70.27 42.95 30.09 22.92
f1 (ppm)

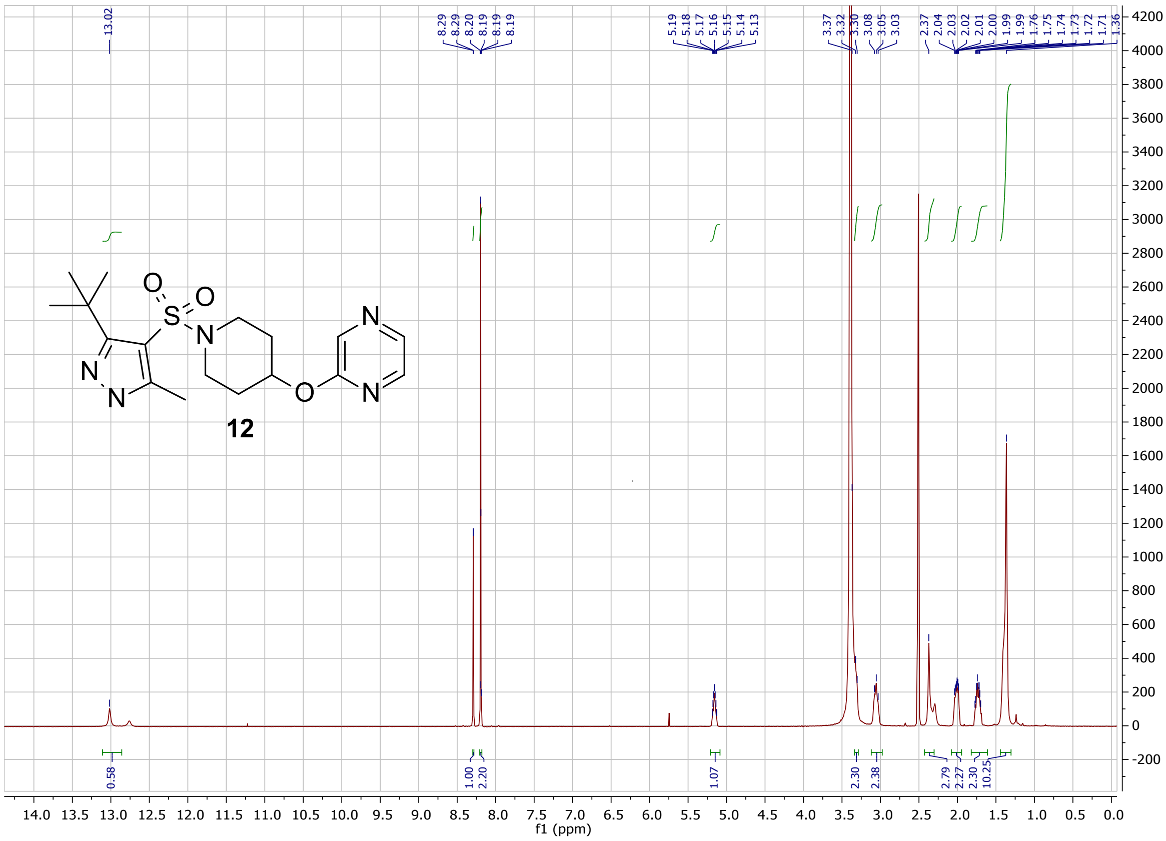
13.02
8.29
8.29
8.20
8.19
8.19
8.19
5.19
5.18
5.17
5.16
5.15
5.14
5.13
3.37
3.32
3.30
3.08
3.05
3.03
2.37
2.04
2.03
2.02
2.01
2.00
1.99
1.76
1.75
1.74
1.73
1.72
1.71
1.36
0.58
1.00
2.20
1.07
2.30
2.38
2.79
2.27
2.30
10.25
N
O
S
O
N
N
N
O
N
12
f1 (ppm)

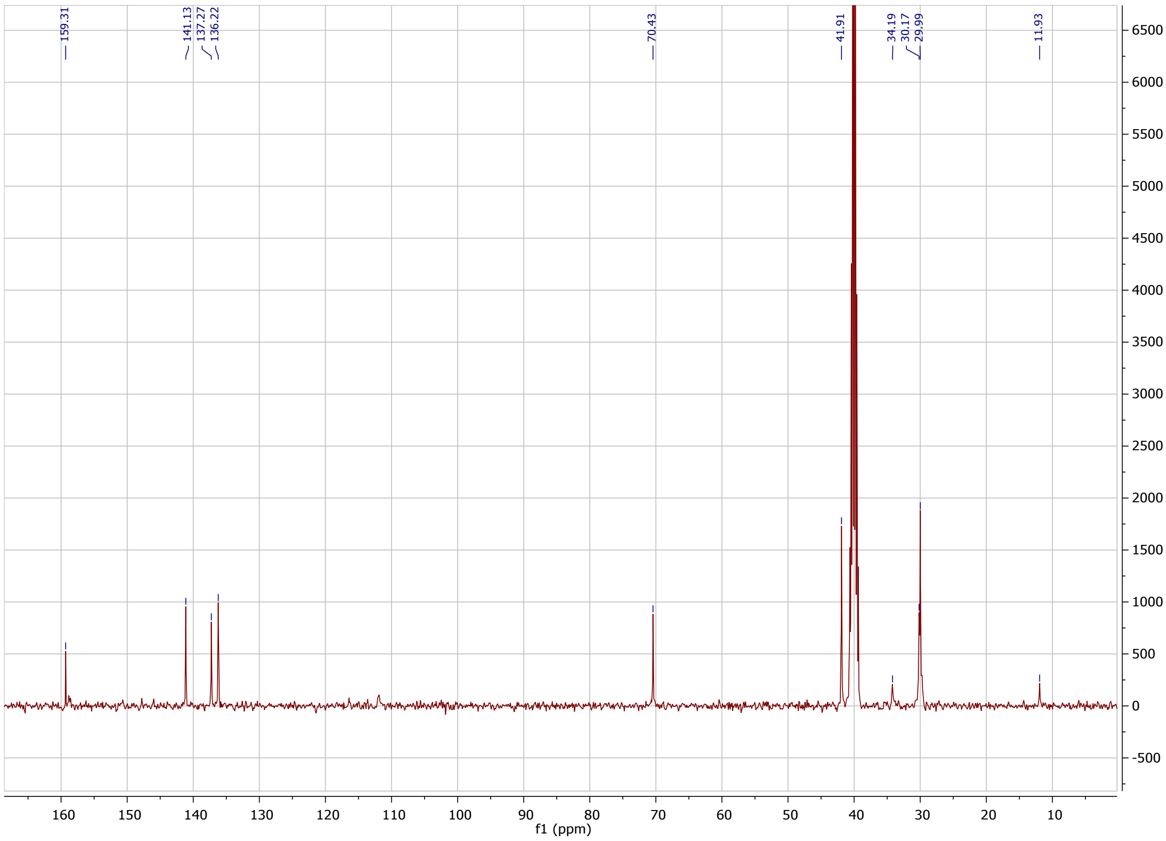
159.31
141.13
137.27
136.22
70.43
41.91
34.19
30.17
29.99
11.93
f1 (ppm)

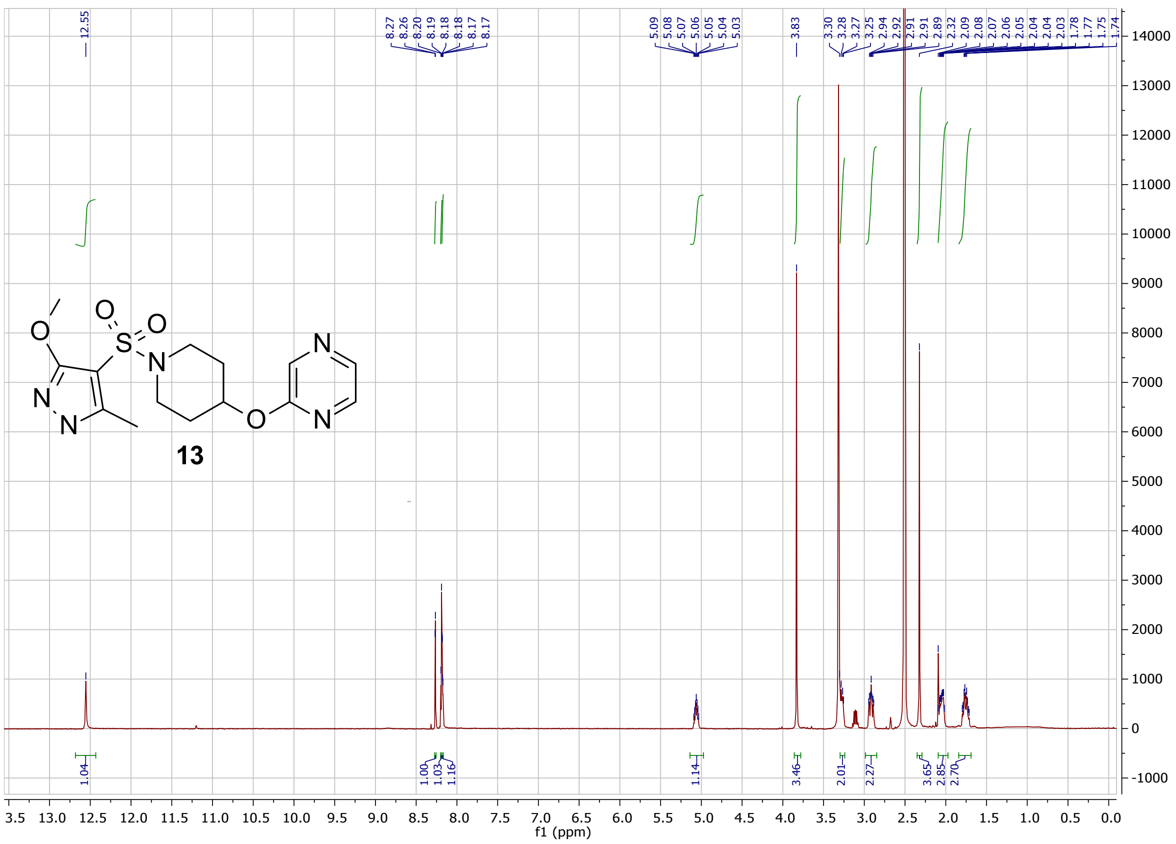
13
f1 (ppm)

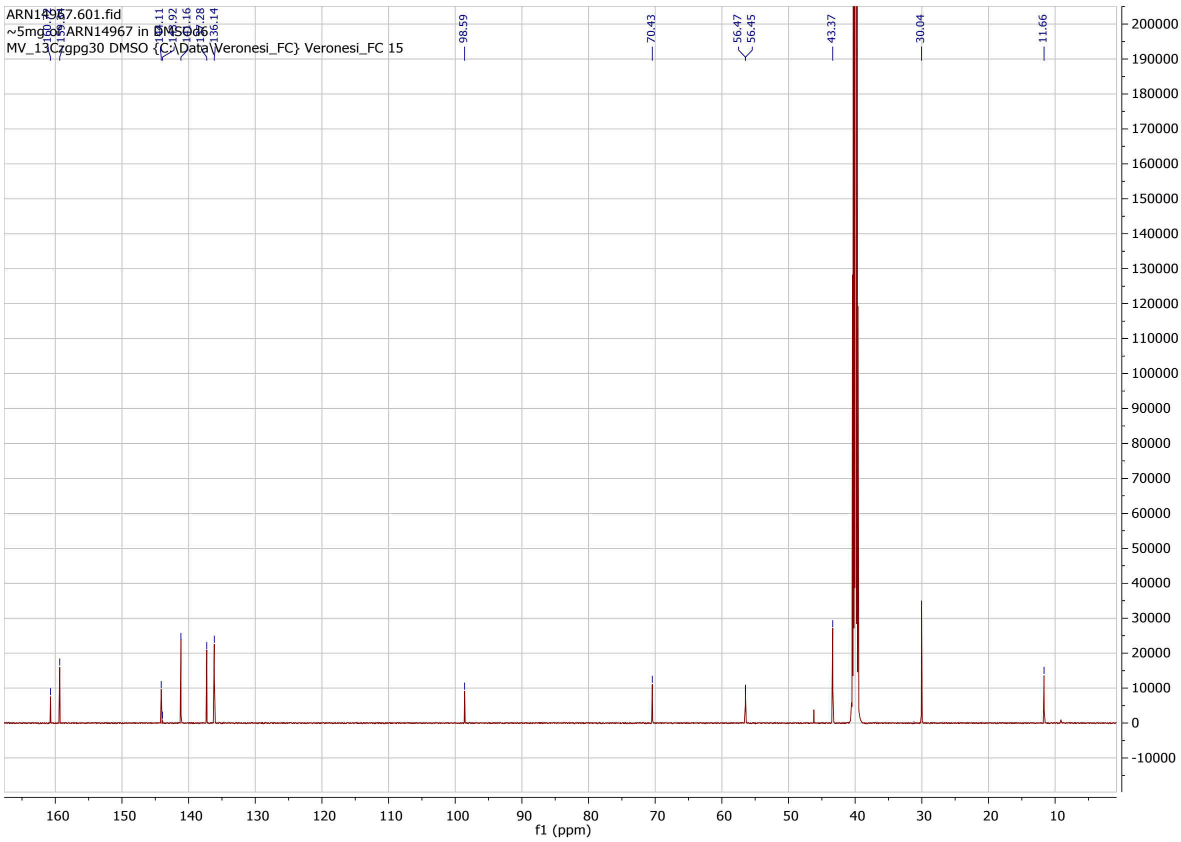
ARN14967.601.fid
~5mg of ARN14967 in DMSOd6
MV_13Czgpg30 DMSO {C:\Data\Veronesi_FC} Veronesi_FC 15
f1 (ppm)

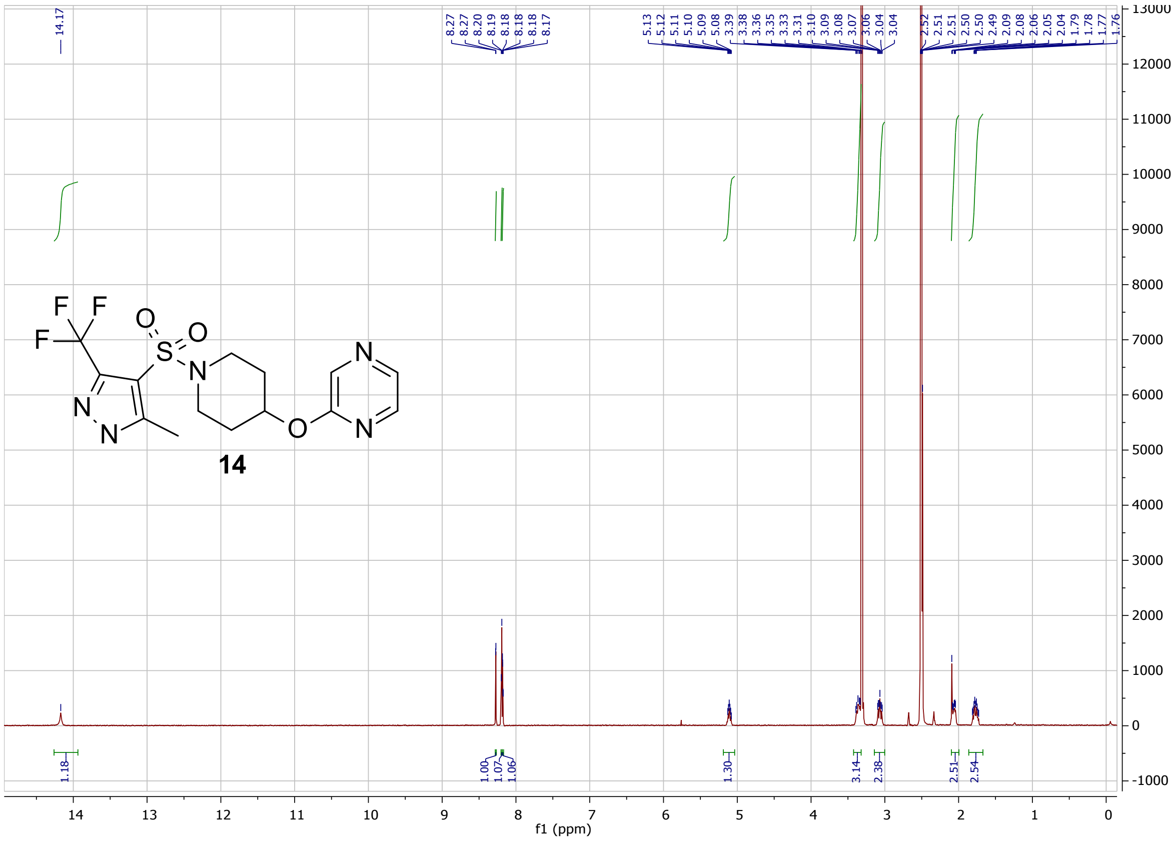

14.17
8.27 8.27 8.20 8.19 8.18 8.18 8.18 8.17
5.13 5.12 5.11 5.10 5.09 5.08 3.39 3.38 3.36 3.35 3.33 3.31 3.10 3.09 3.08 3.07 3.06 3.04 3.04
2.52 2.51 2.51 2.50 2.50 2.49 2.09 2.08 2.06 2.05 2.04 1.79 1.78 1.77 1.76
F F F O O S N N N O N N
14
1.18 1.00 1.07 1.06 1.30 3.14 2.38 2.51 2.54
f1 (ppm)

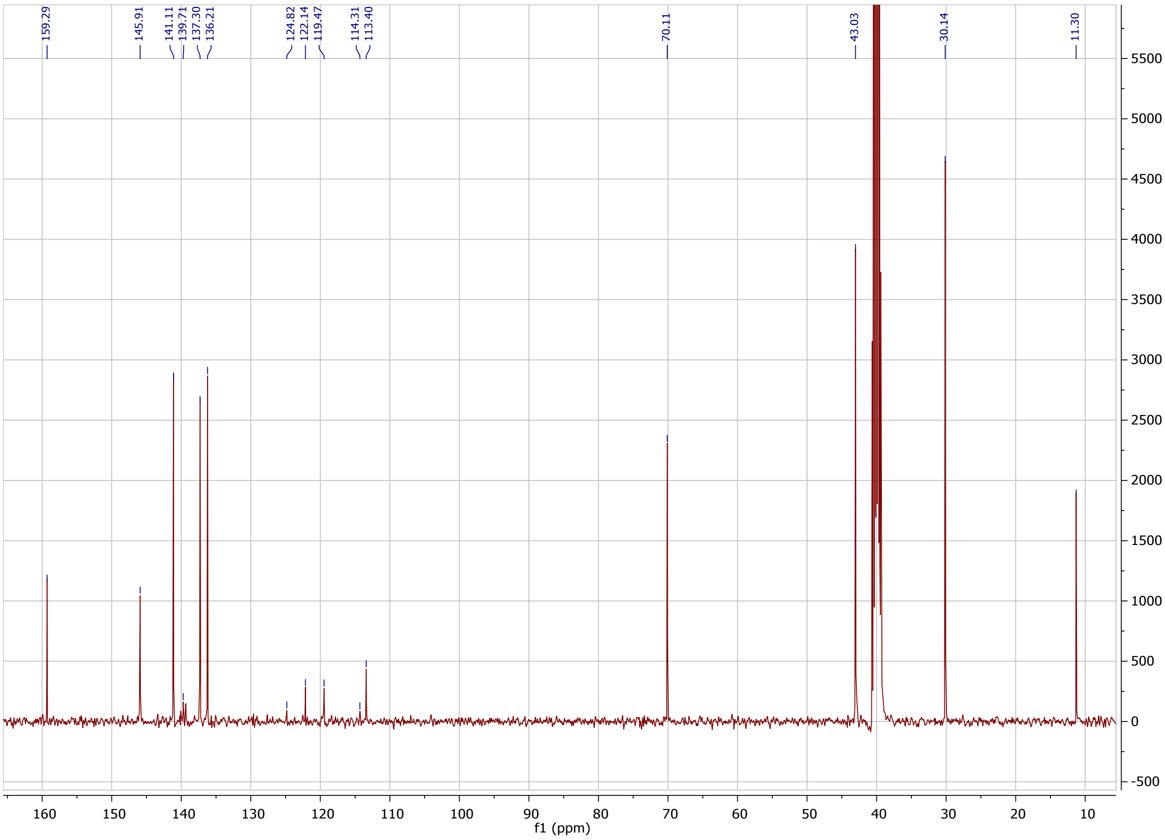

159.29
145.91 141.11 139.71 137.30 136.21
124.82 122.14 119.47 114.31 113.40
70.11
43.03
30.14
11.30
f1 (ppm)

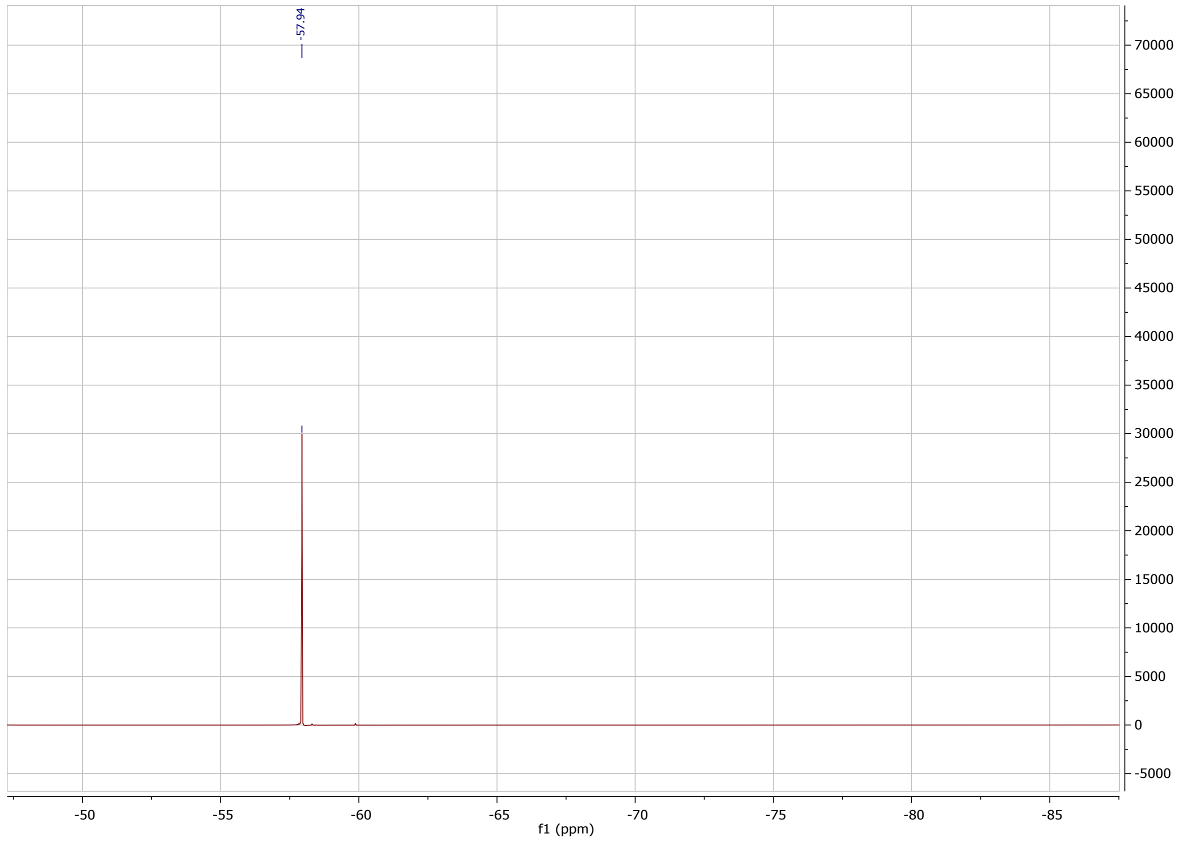

-57.94
70000
65000
60000
55000
50000
45000
40000
35000
30000
25000
20000
15000
10000
5000
0
-5000
-50
-55
-60
-65
-70
-75
-80
-85
f1 (ppm)

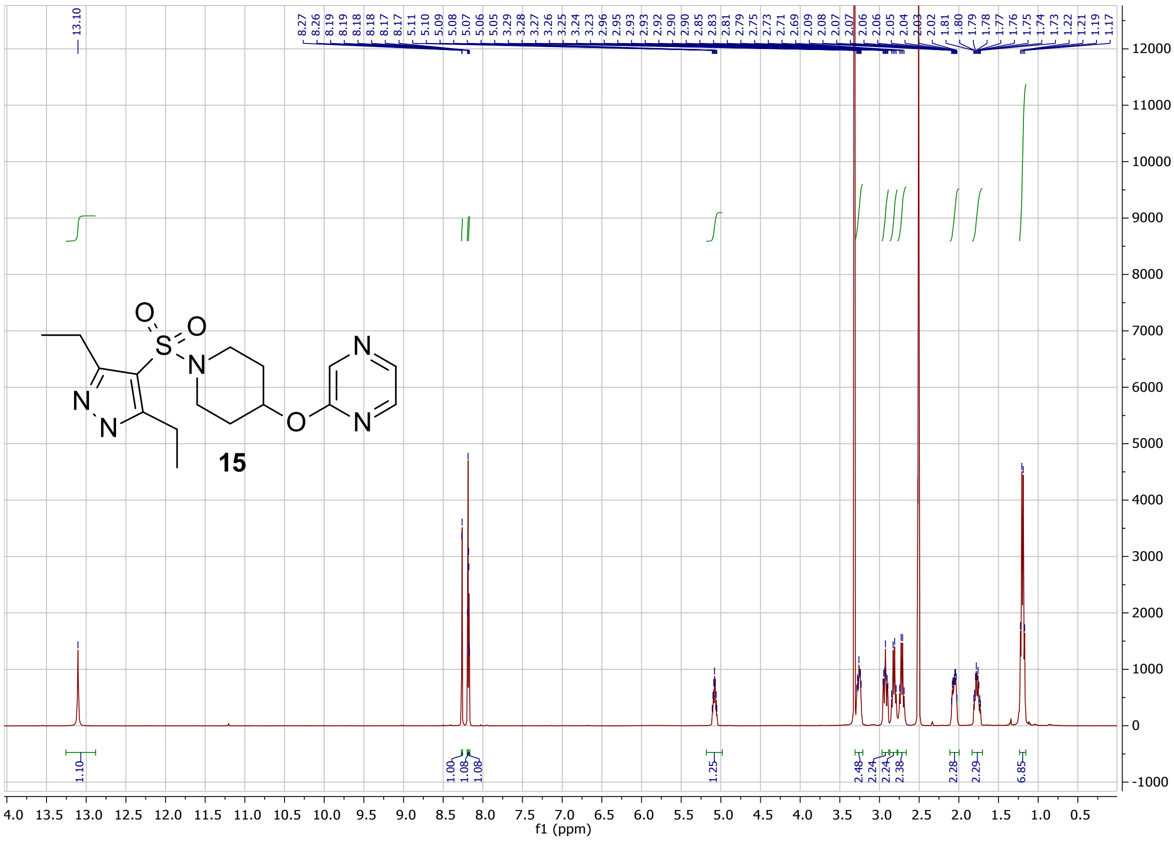
15
f1 (ppm)

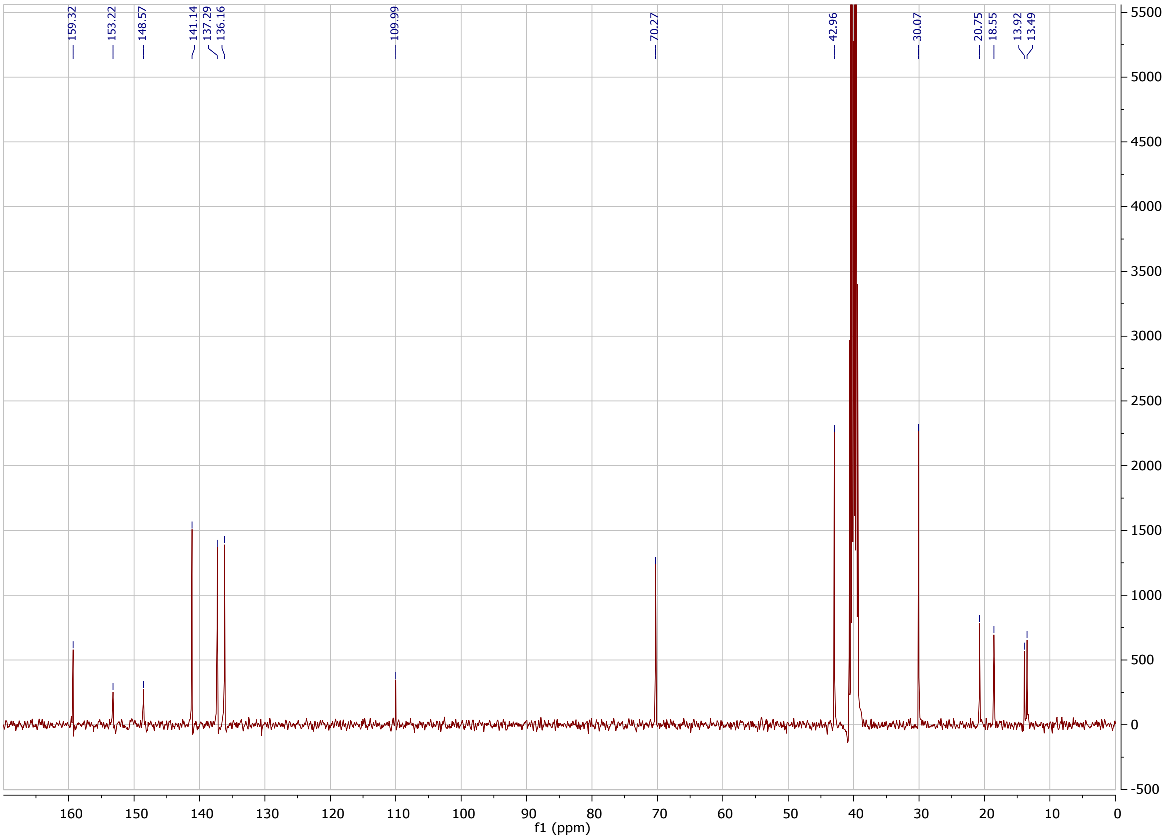
f1 (ppm)

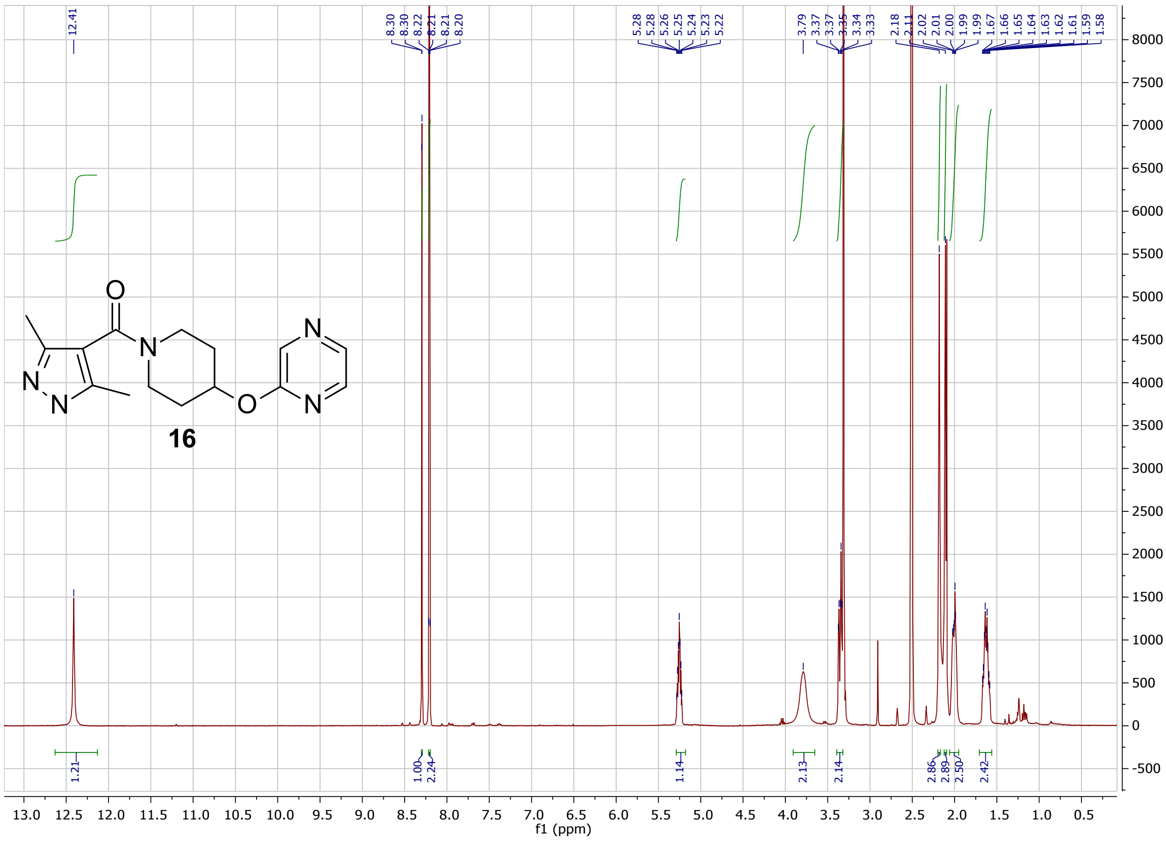
12.41
8.30 8.30 8.22 8.21 8.21 8.20
5.28 5.28 5.26 5.25 5.24 5.23 5.22
3.79 3.37 3.37 3.35 3.34 3.33
2.18 2.11 2.02 2.01 2.00 1.99 1.99 1.67 1.66 1.65 1.64 1.63 1.62 1.61 1.59 1.58
16
1.21 1.00 2.24 1.14 2.13 2.14 2.86 2.89 2.50 2.42
f1 (ppm)

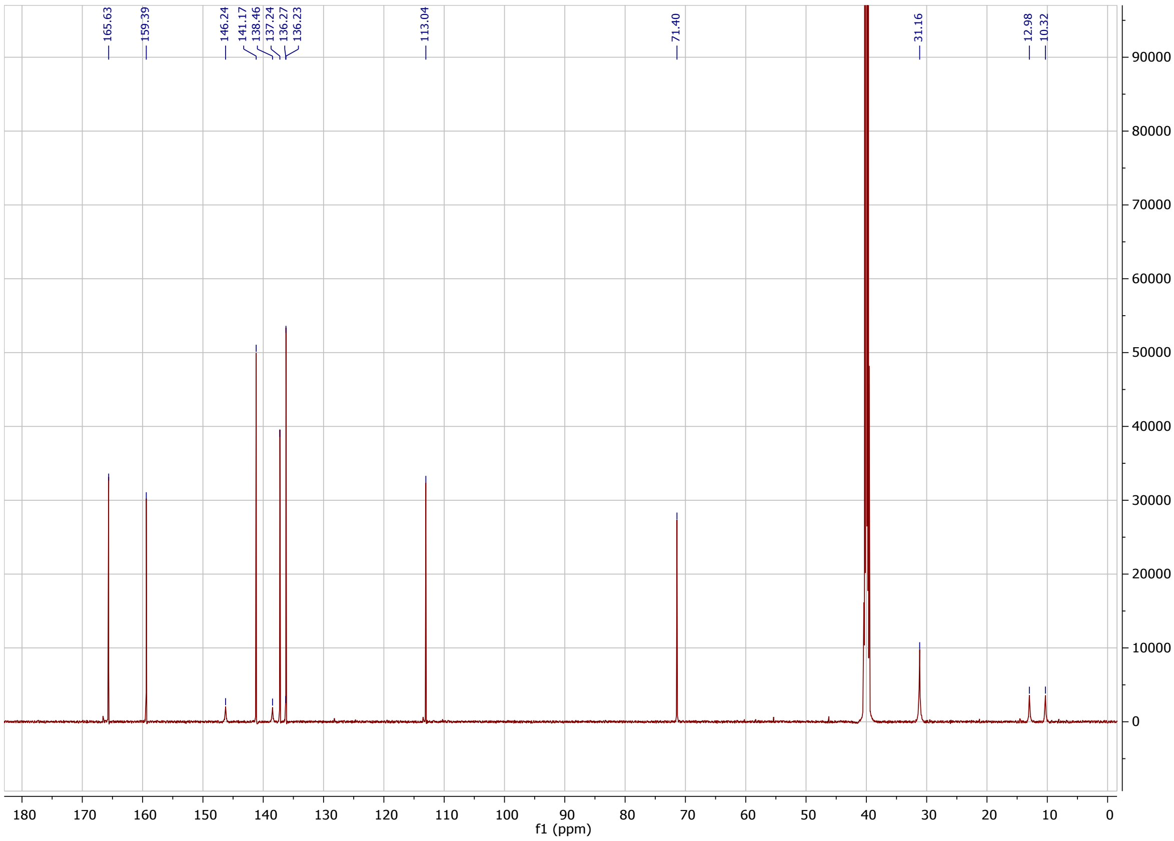
165.63
159.39
146.24 141.17 138.46 137.24 136.27 136.23
113.04
71.40
31.16
12.98 10.32
f1 (ppm)

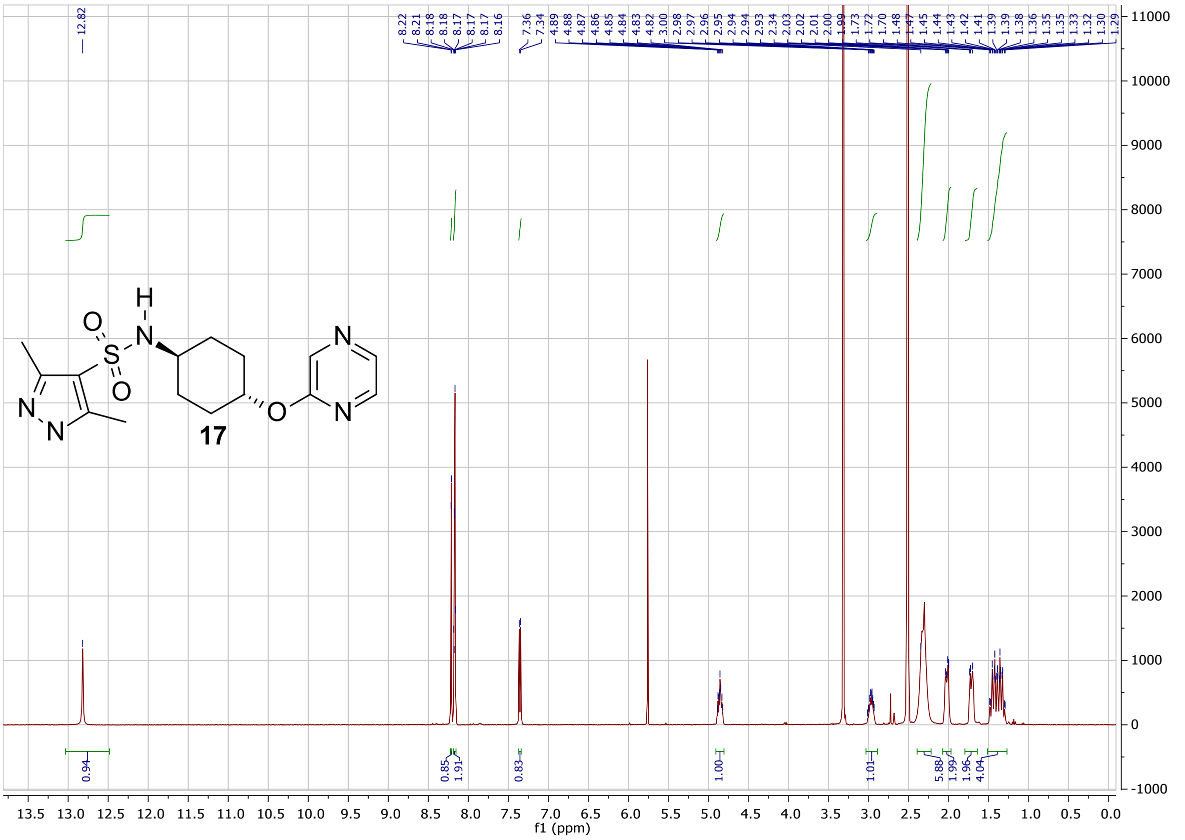
12.82
8.22
8.21
8.18
8.17
8.17
8.17
8.16
7.36
7.34
17
0.94
0.85
1.91
0.83
1.00
1.01
5.88
1.99
1.96
4.04
f1 (ppm)

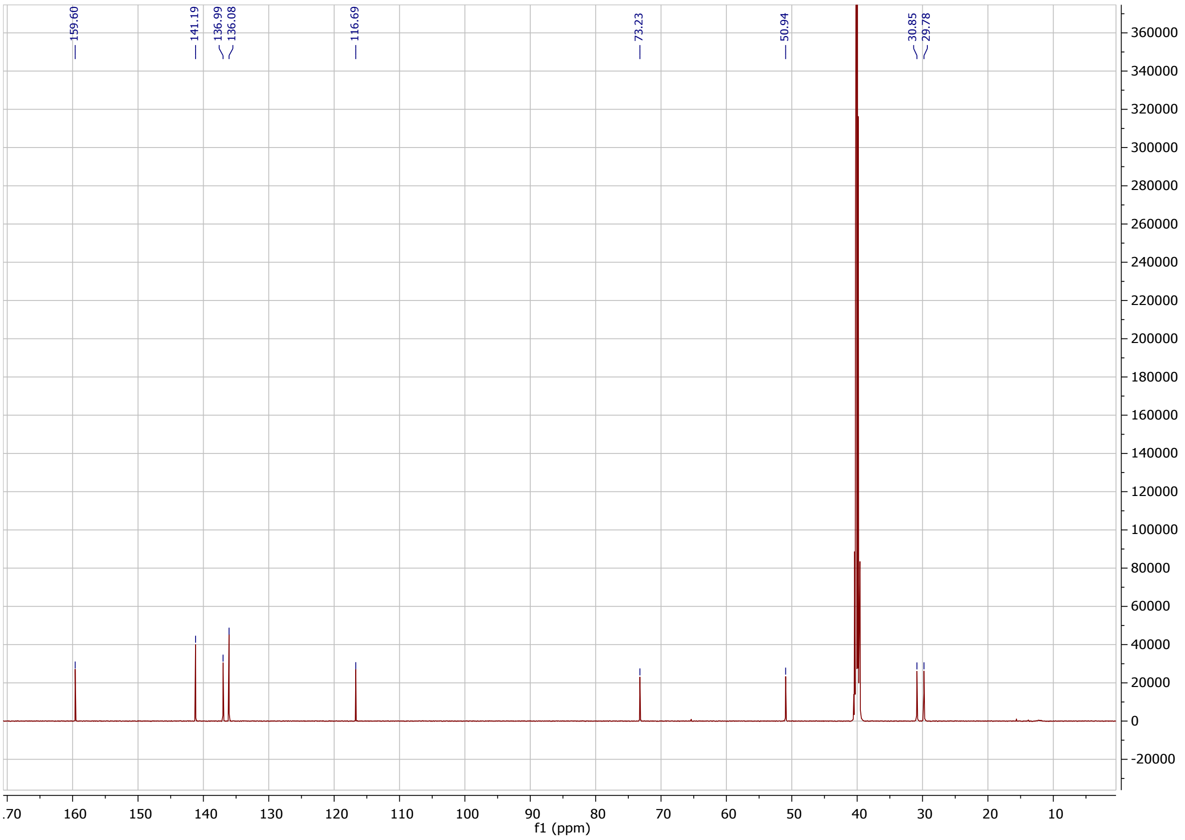
159.60
141.19
136.99
136.08
116.69
73.23
50.94
30.85
29.78
f1 (ppm)

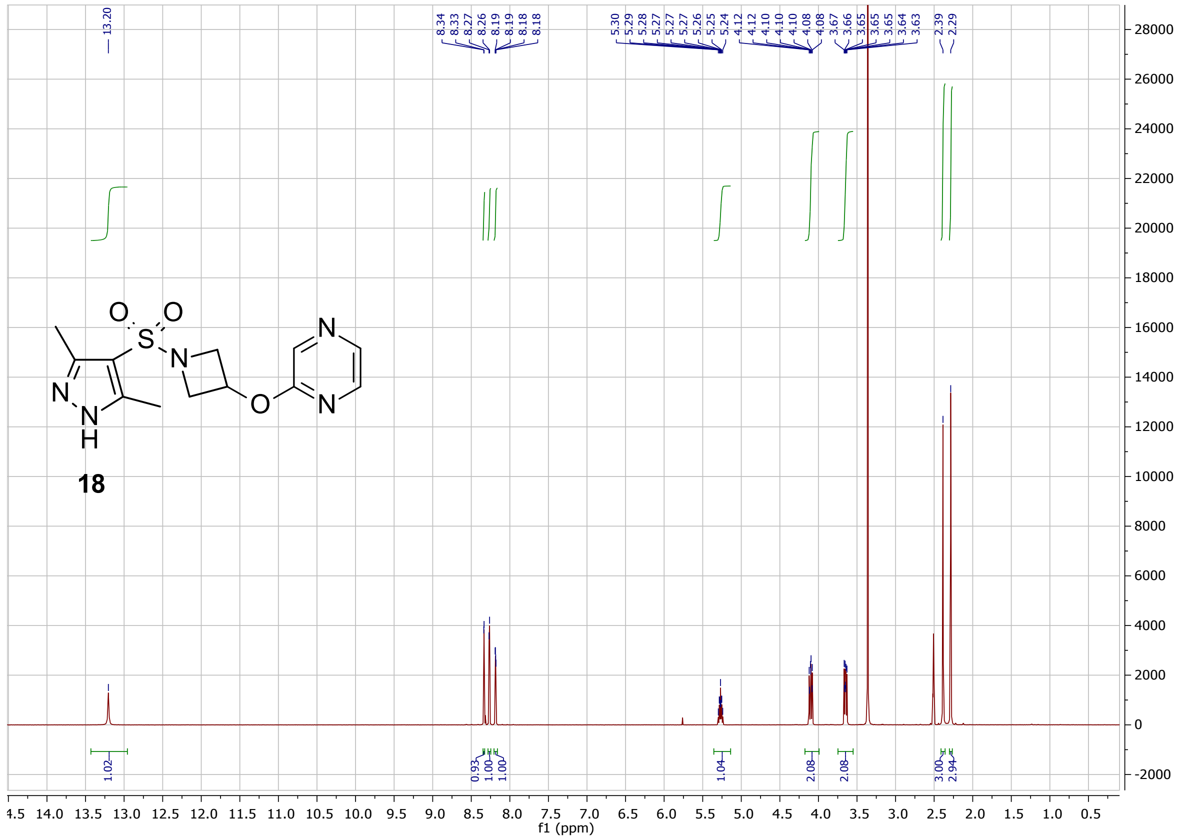
13.20
8.34
8.33
8.27
8.26
8.19
8.19
8.18
8.18
5.30
5.29
5.28
5.27
5.27
5.27
5.26
5.25
5.24
4.12
4.12
4.10
4.10
4.08
4.08
3.67
3.66
3.65
3.65
3.65
3.64
3.63
2.39
2.29
1.02
0.93
1.00
1.00
1.04
2.08
2.08
3.00
2.94
18
f1 (ppm)

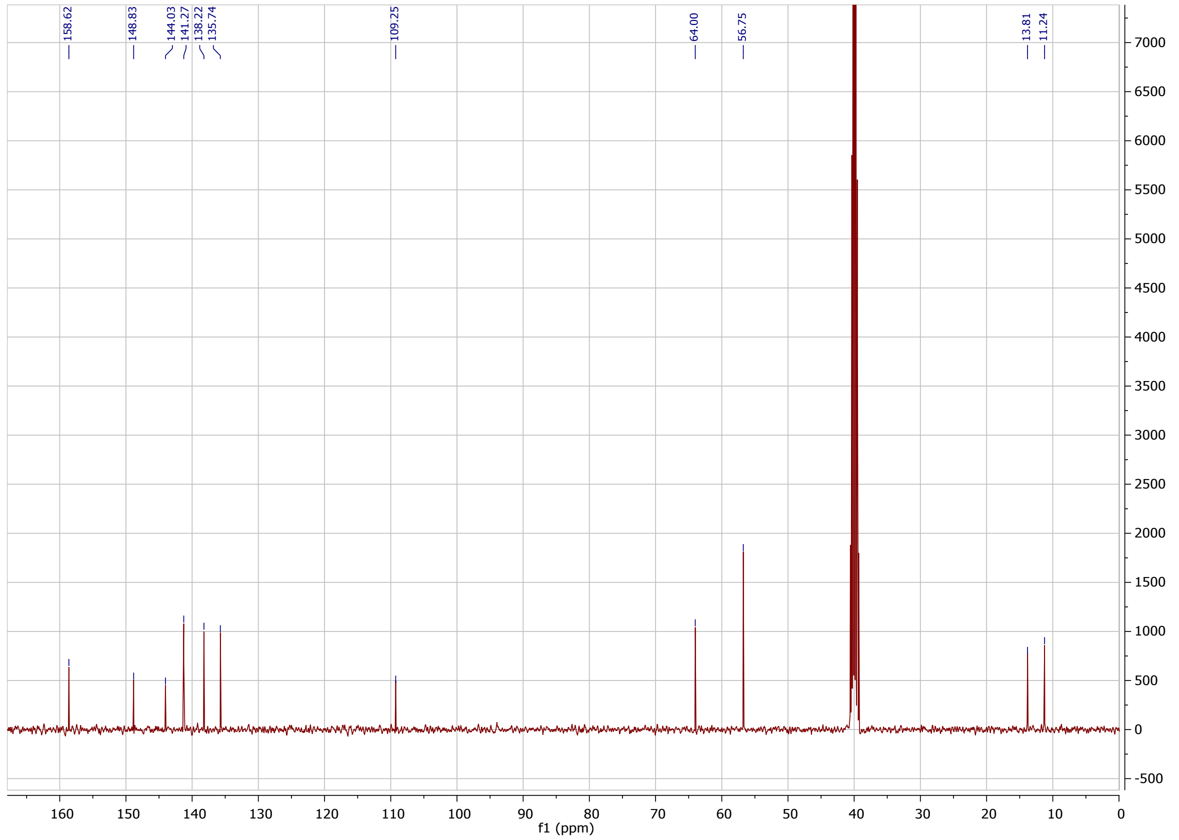
158.62
148.83
144.03
141.27
138.22
135.74
109.25
64.00
56.75
13.81
11.24
f1 (ppm)

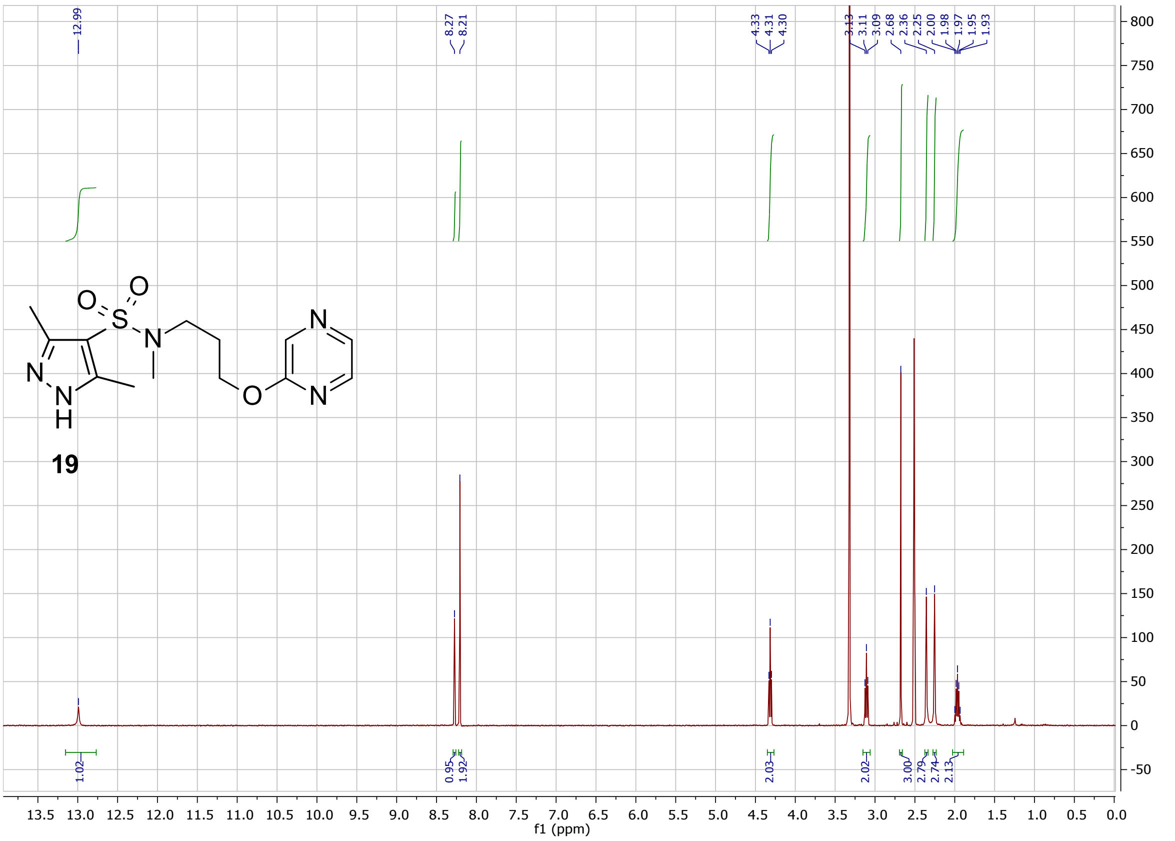
12.99
8.27
8.21
4.33
4.31
4.30
3.13
3.11
3.09
2.68
2.36
2.25
2.00
1.98
1.97
1.95
1.93
19
1.02
0.95
1.92
2.03
2.02
3.00
2.79
2.74
2.13
f1 (ppm)

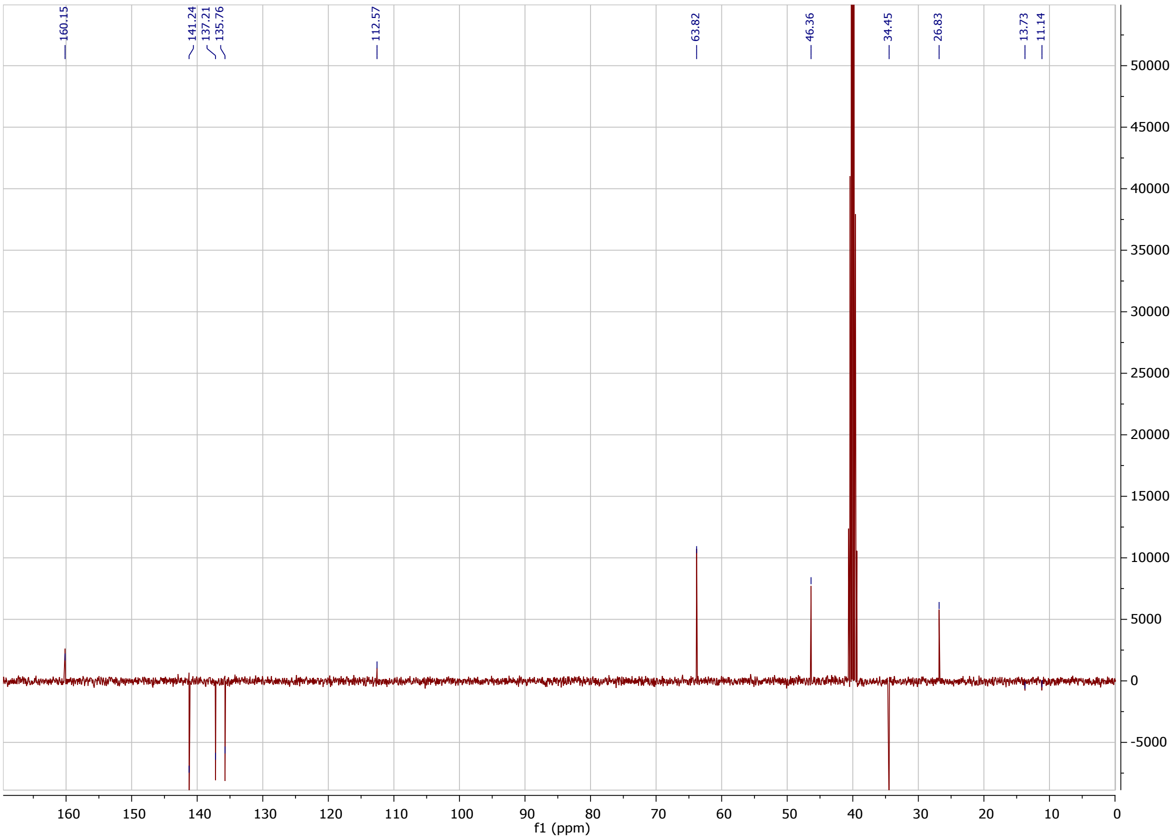
160.15
141.24
137.21
135.76
112.57
63.82
46.36
34.45
26.83
13.73
11.14
f1 (ppm)

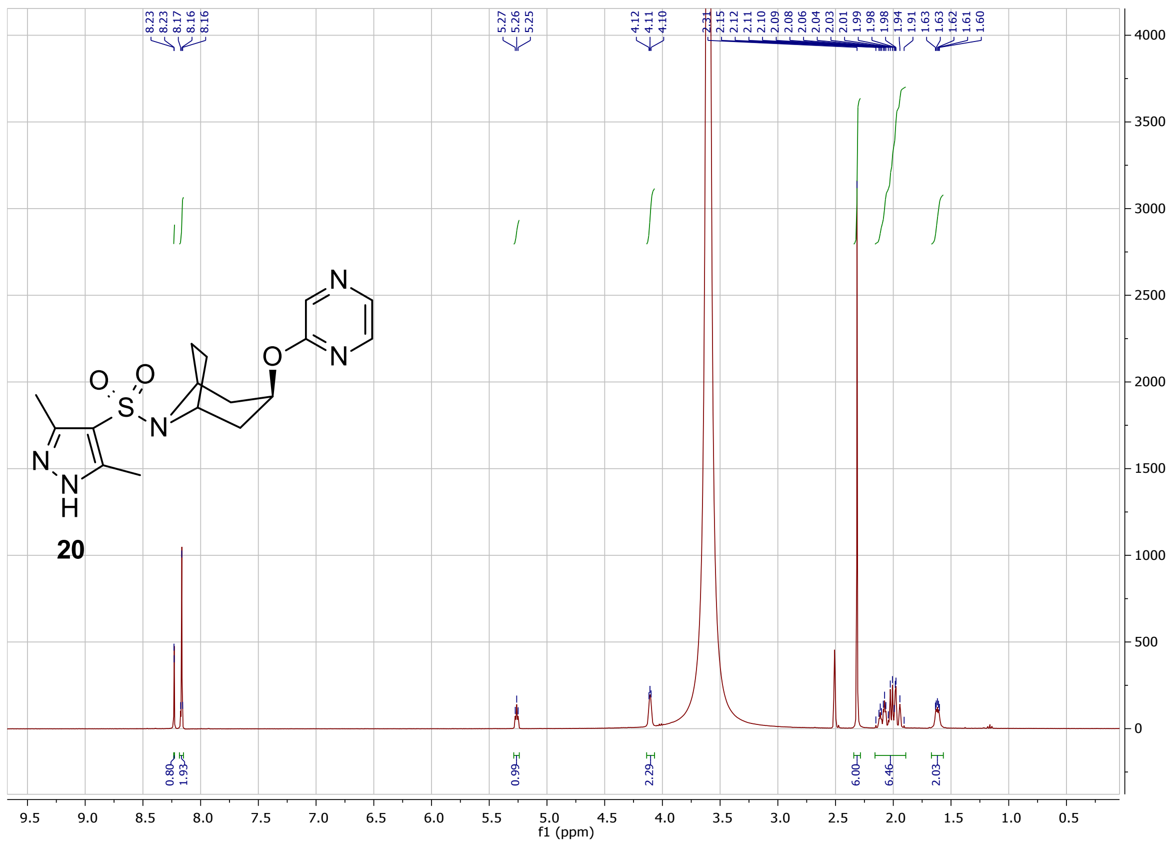

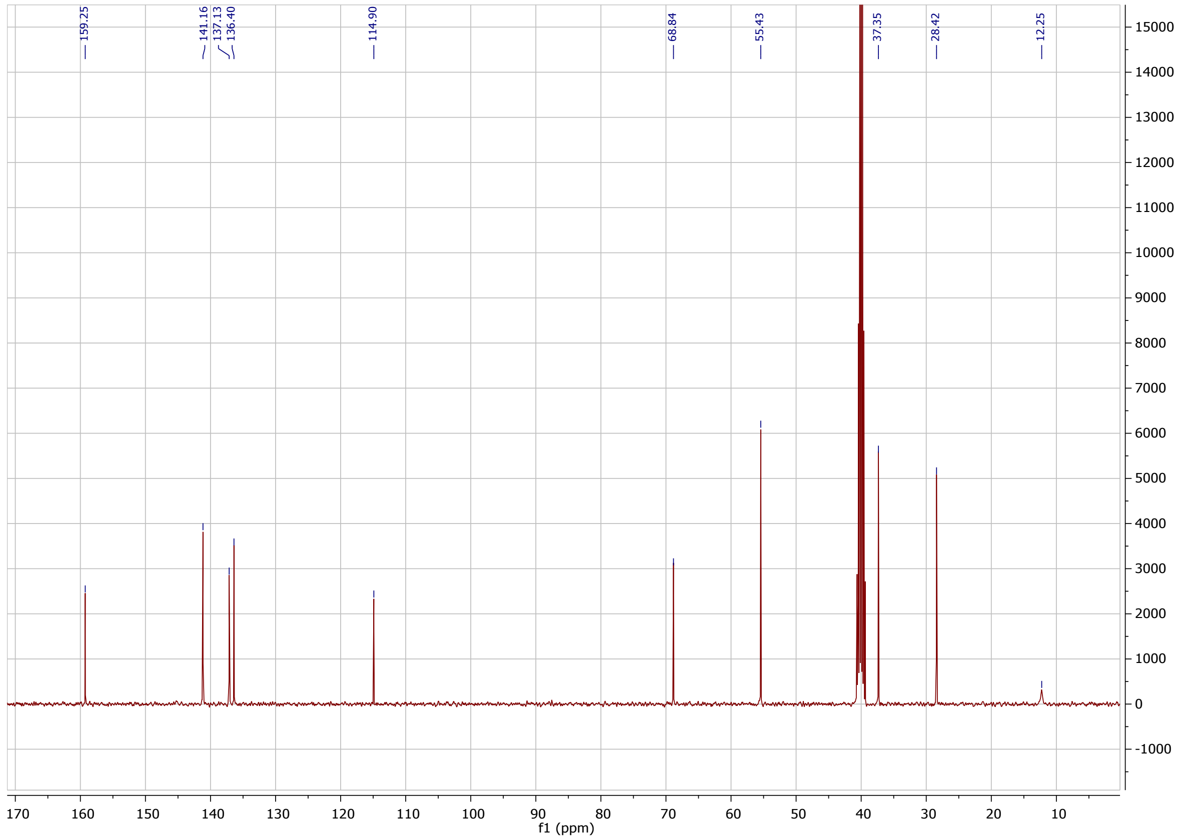

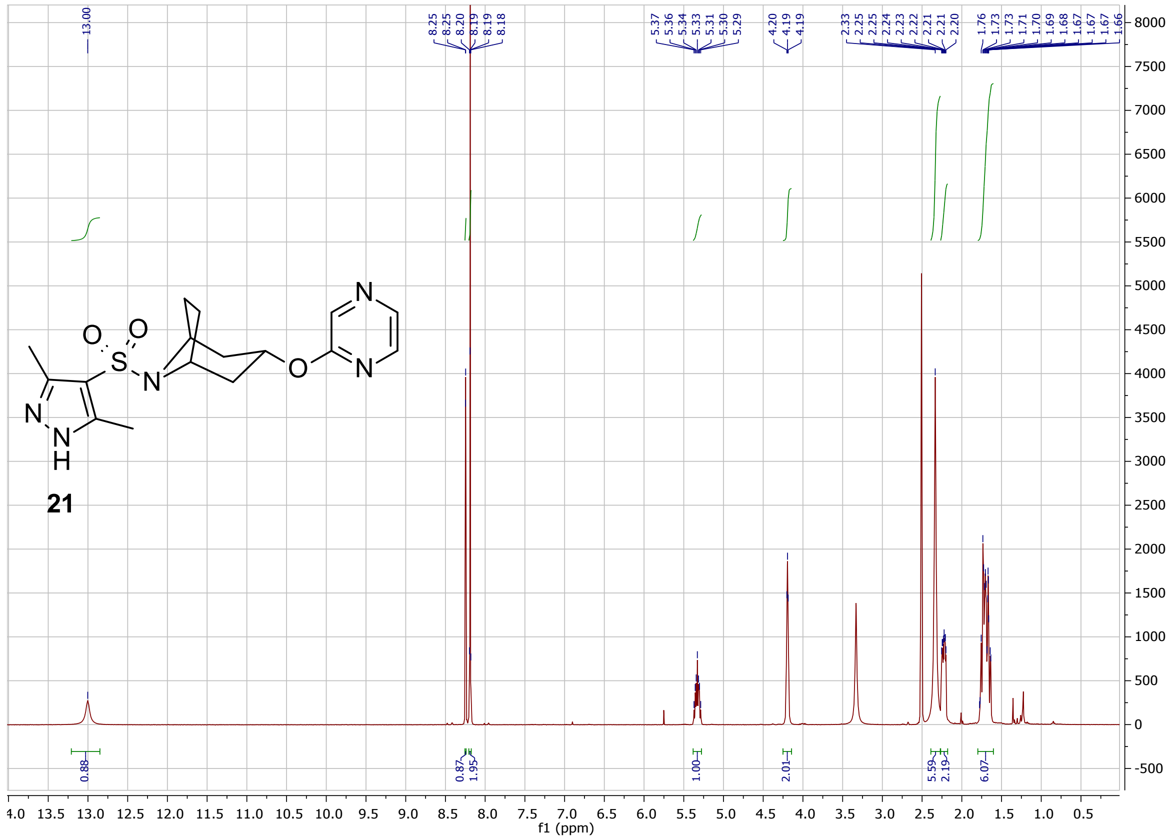

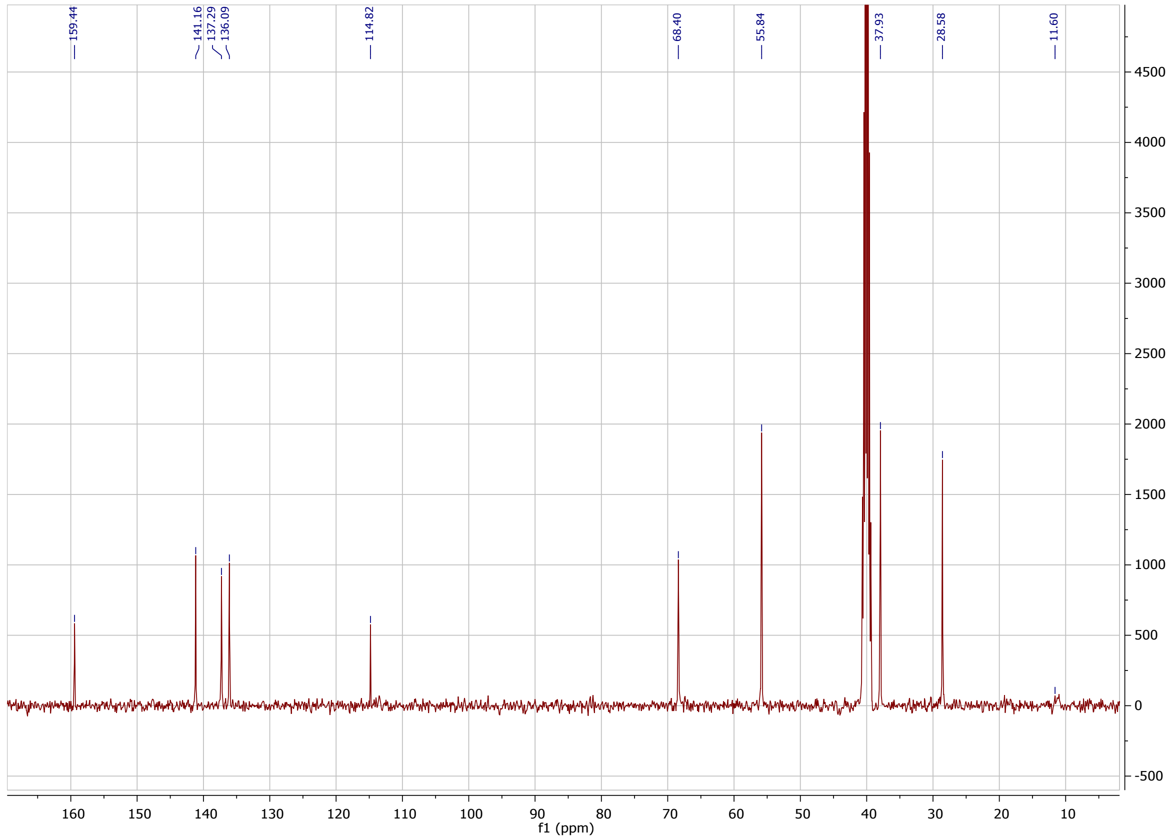

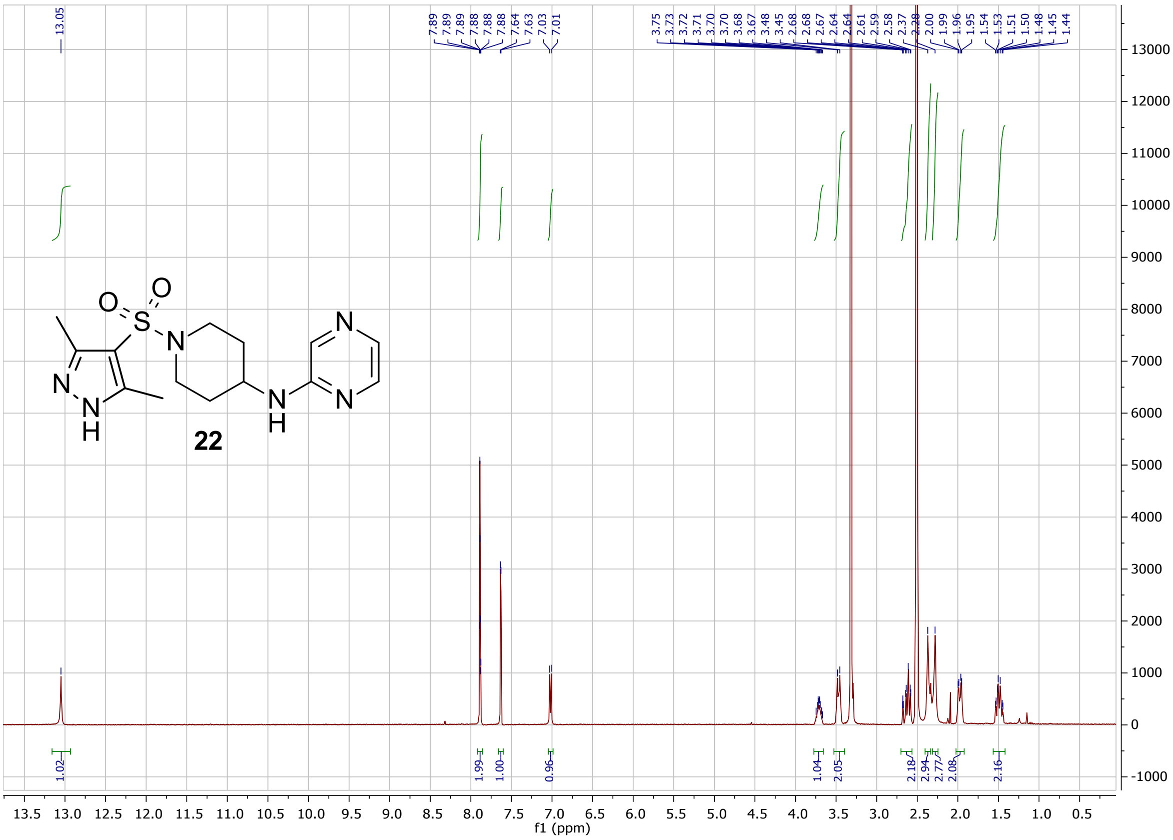

13.05
7.89 7.89 7.89 7.88 7.88 7.88 7.64 7.63 7.03 7.01
3.75 3.73 3.72 3.71 3.70 3.70 3.68 3.67 3.48 3.45 2.68 2.67 2.64 2.64 2.61 2.59 2.58 2.37 2.28 2.00 1.99 1.96 1.95 1.54 1.53 1.51 1.50 1.48 1.45 1.44
22
1.02
1.99
1.00
0.96
1.04
2.05
2.18
2.94
2.77
2.08
2.16
f1 (ppm)

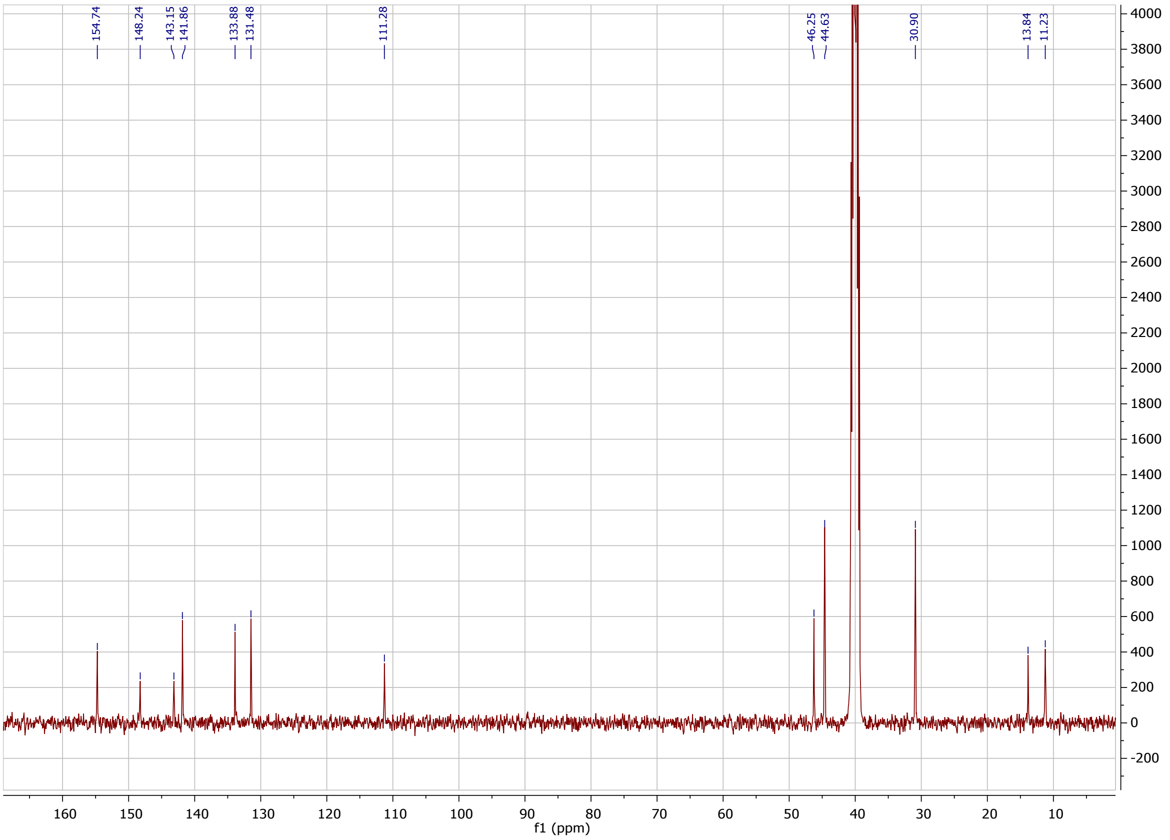

154.74
148.24
143.15
141.86
133.88
131.48
111.28
46.25
44.63
30.90
13.84
11.23
f1 (ppm)

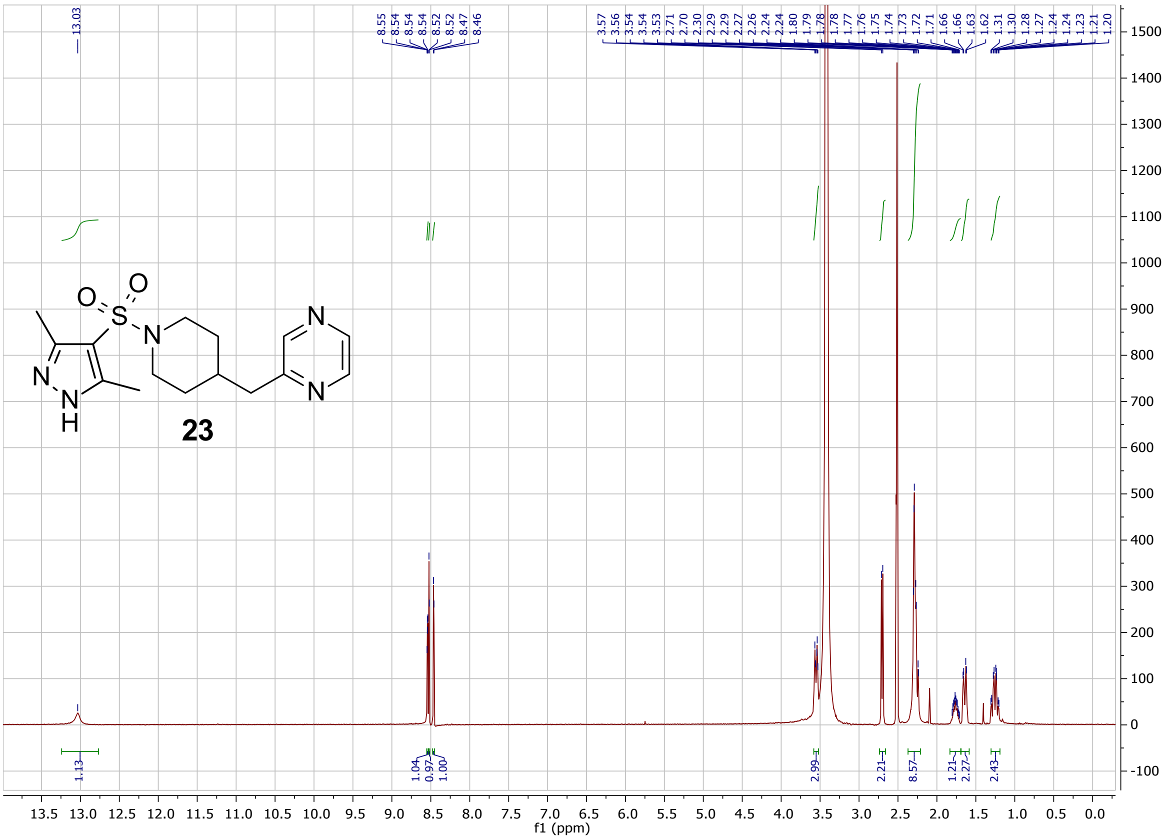

23
f1 (ppm)

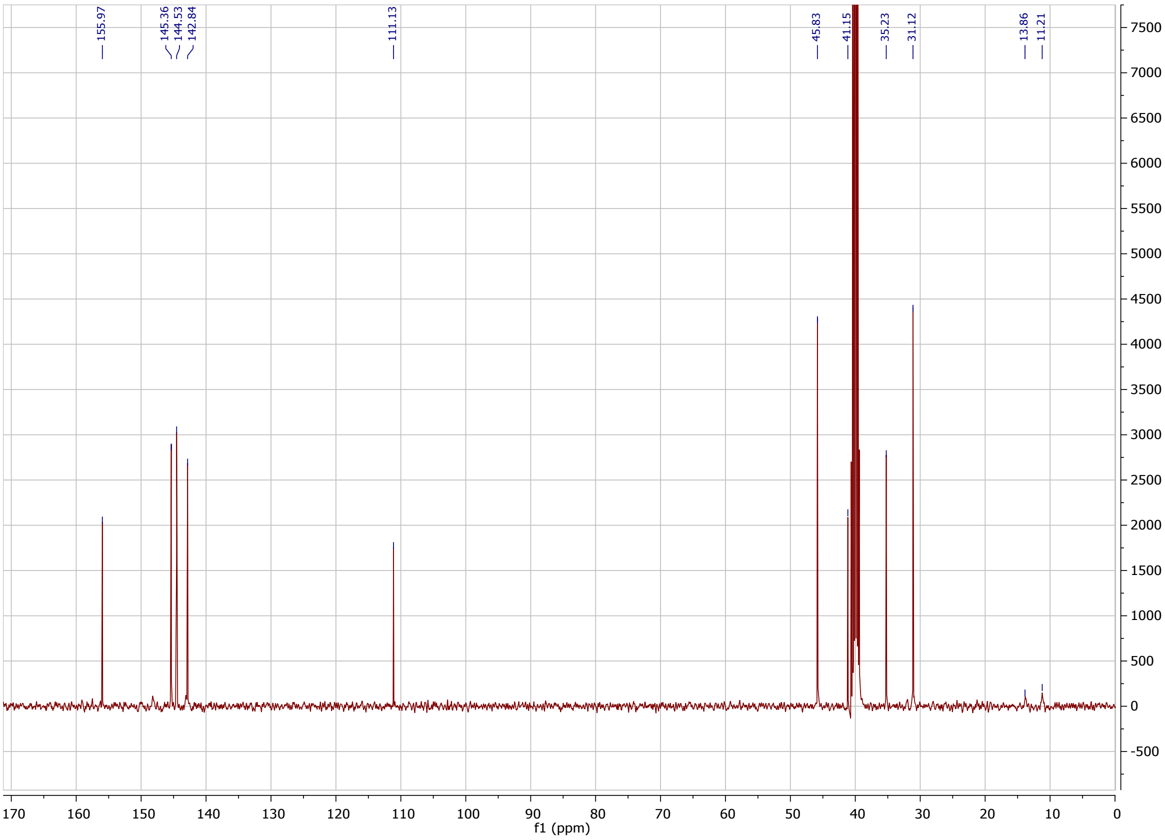

155.97
145.36
144.53
142.84
111.13
45.83
41.15
35.23
31.12
13.86
11.21
f1 (ppm)

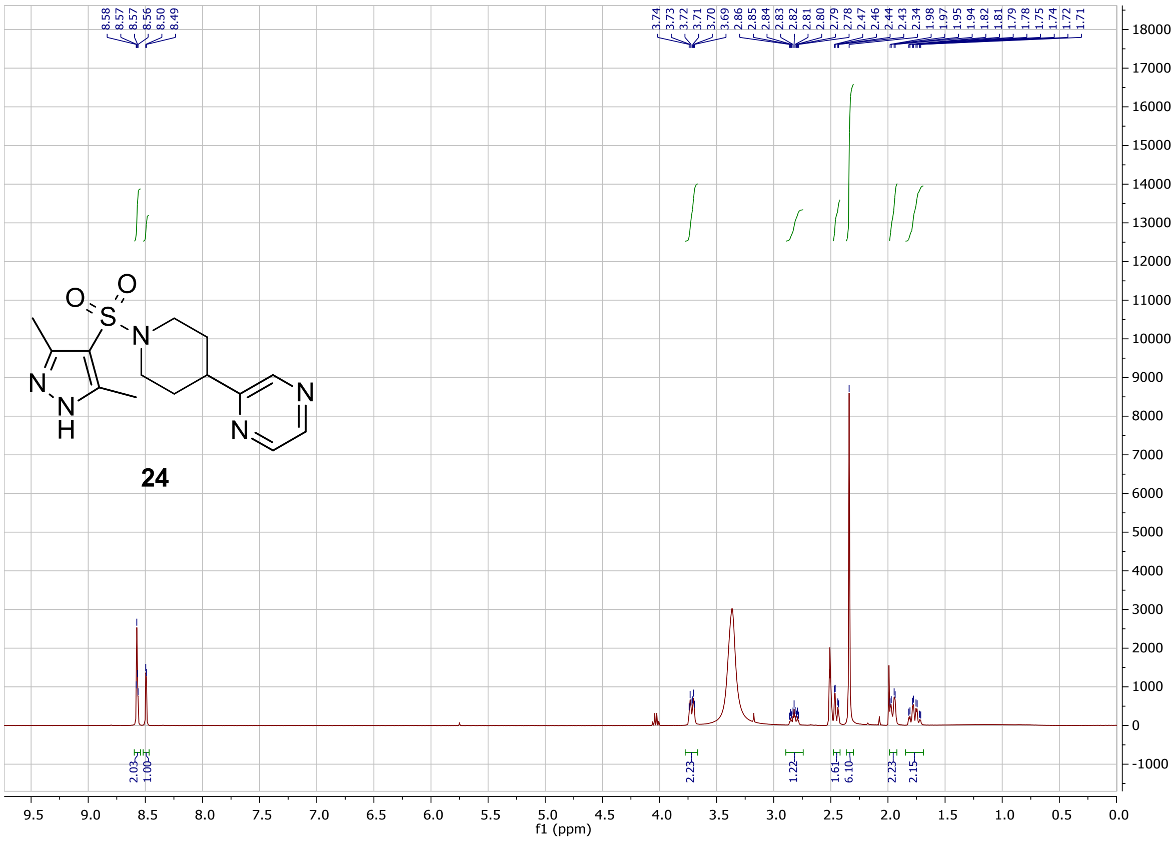
8.58
8.57
8.57
8.56
8.50
8.49
3.74
3.73
3.72
3.71
3.70
3.69
2.86
2.85
2.84
2.83
2.82
2.81
2.80
2.79
2.78
2.47
2.46
2.44
2.43
2.34
1.98
1.97
1.95
1.94
1.82
1.81
1.79
1.78
1.75
1.74
1.72
1.71
2.03
1.00
2.23
1.22
1.61
6.10
2.23
2.15
24
f1 (ppm)

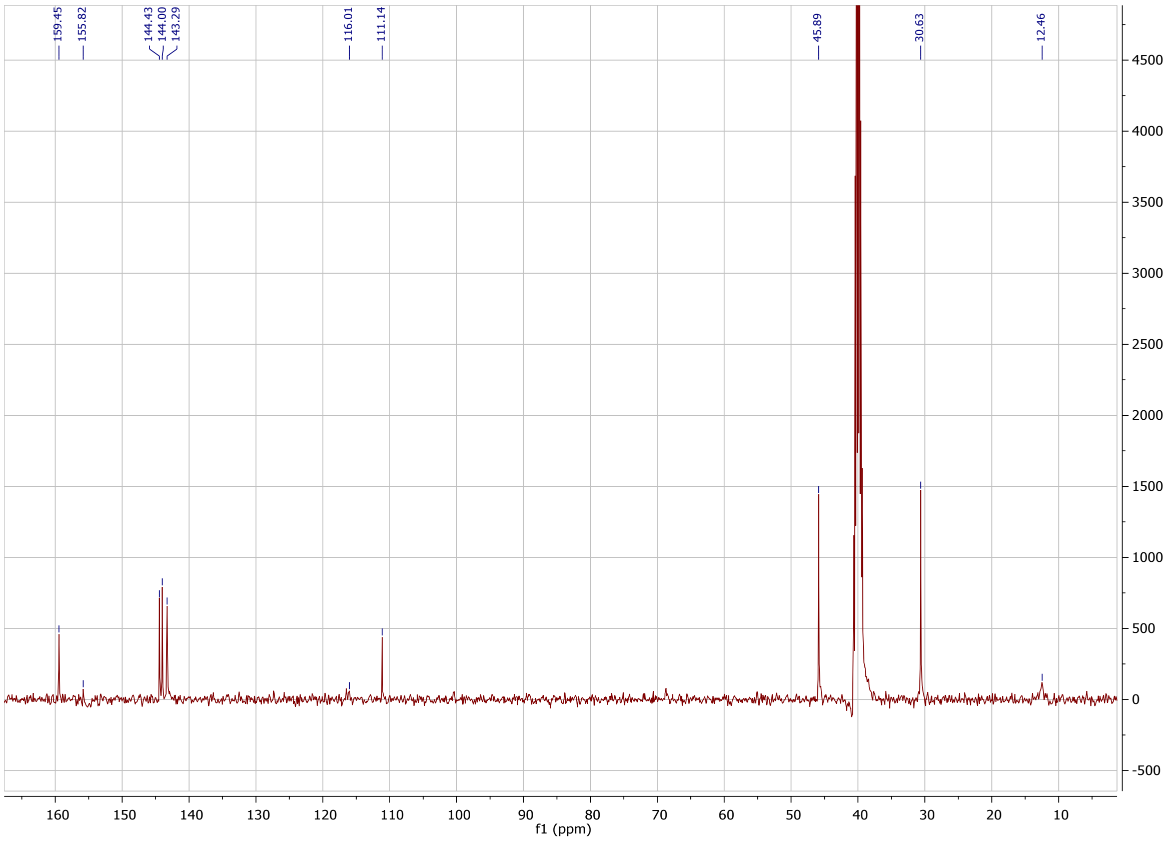
159.45
155.82
144.43
144.00
143.29
116.01
111.14
45.89
30.63
12.46
f1 (ppm)

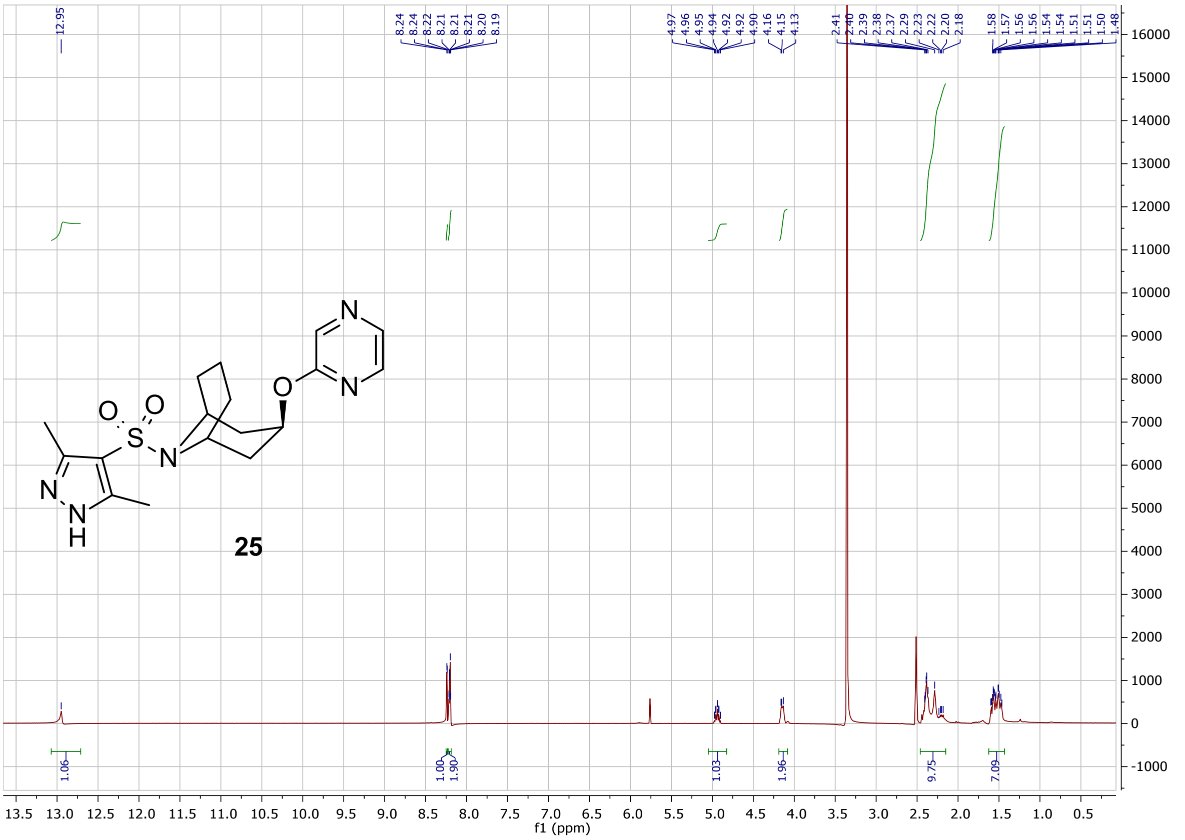
12.95
8.24 8.24 8.22 8.21 8.21 8.21 8.20 8.19
4.97 4.96 4.95 4.94 4.92 4.92 4.90 4.16 4.15 4.13
2.41 2.40 2.39 2.38 2.37 2.29 2.23 2.22 2.20 2.18
1.58 1.57 1.56 1.56 1.54 1.54 1.51 1.50 1.48
1.06
1.00
1.90
1.03
1.96
9.75
7.09
N
O
O
O
S
N
N
N
H
25
f1 (ppm)

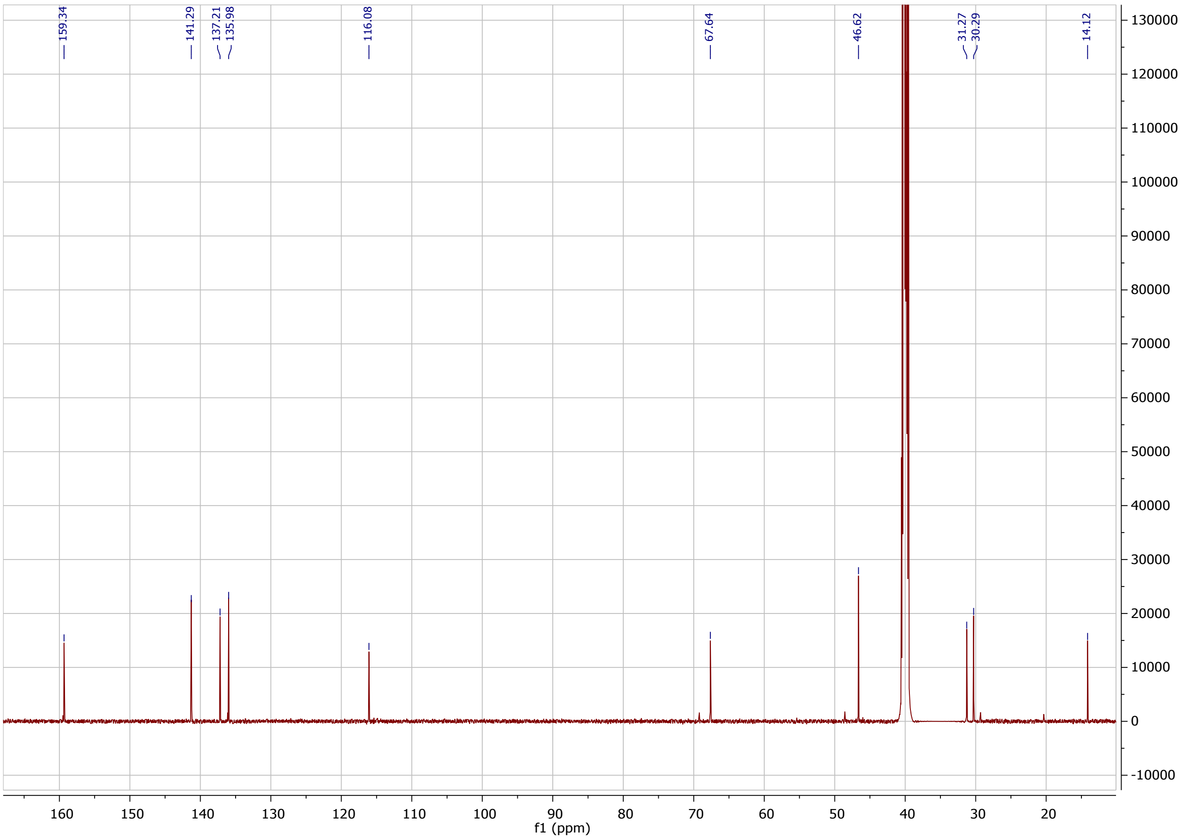
159.34
141.29
137.21
135.98
116.08
67.64
46.62
31.27
30.29
14.12
f1 (ppm)

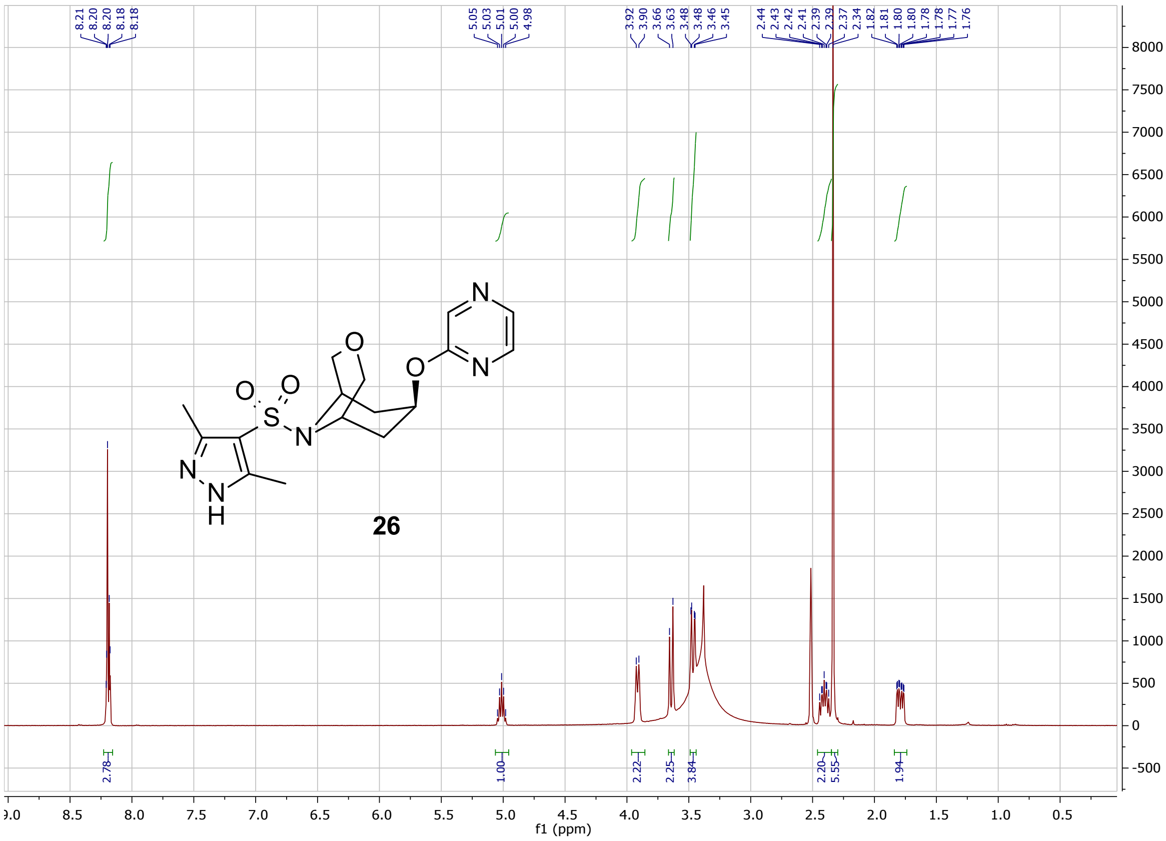
8.21 8.20 8.20 8.18 8.18
5.05 5.03 5.01 5.00 4.98
3.92 3.90 3.66 3.63 3.48 3.48 3.46 3.45
2.44 2.43 2.42 2.41 2.39 2.39 2.37 2.34
1.82 1.81 1.80 1.80 1.78 1.77 1.76
2.78 1.00 2.22 2.25 3.84 2.20 5.55 1.94
26
f1 (ppm)

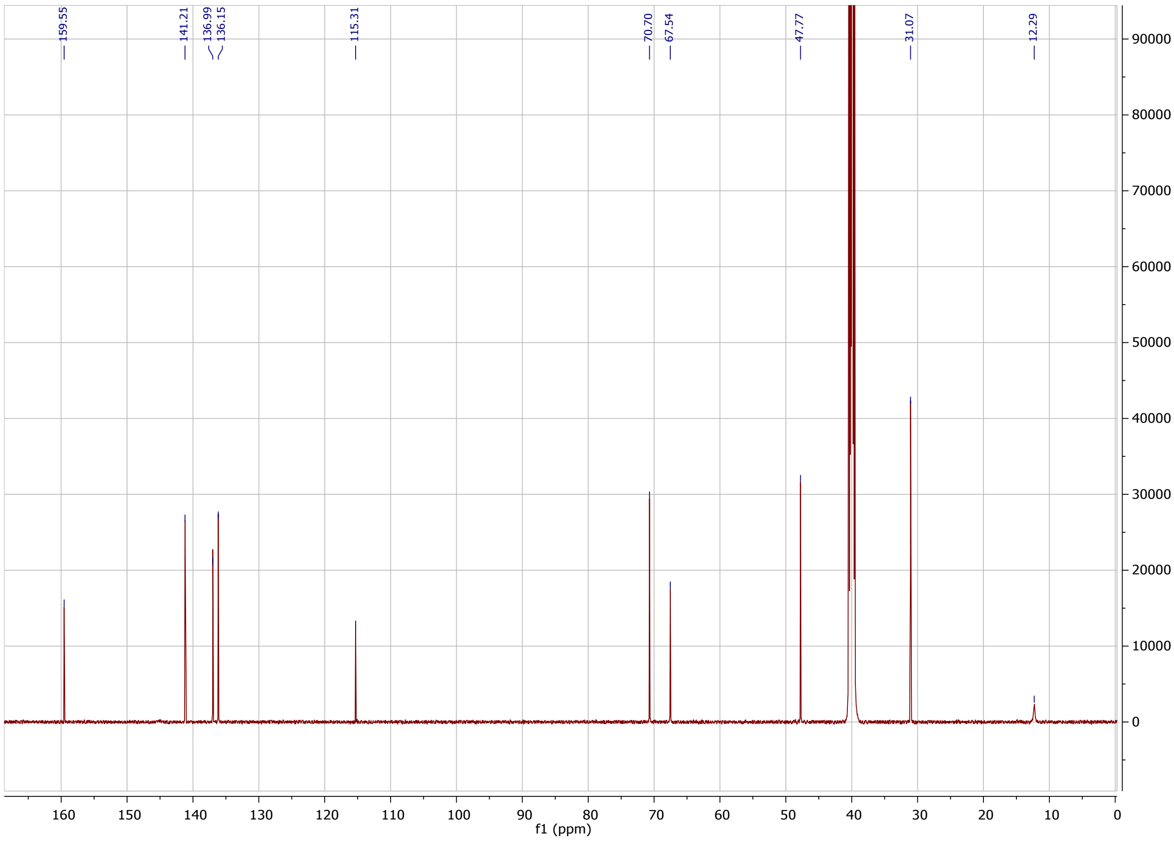
159.55
141.21
136.99
136.15
115.31
70.70
67.54
47.77
31.07
12.29
f1 (ppm)

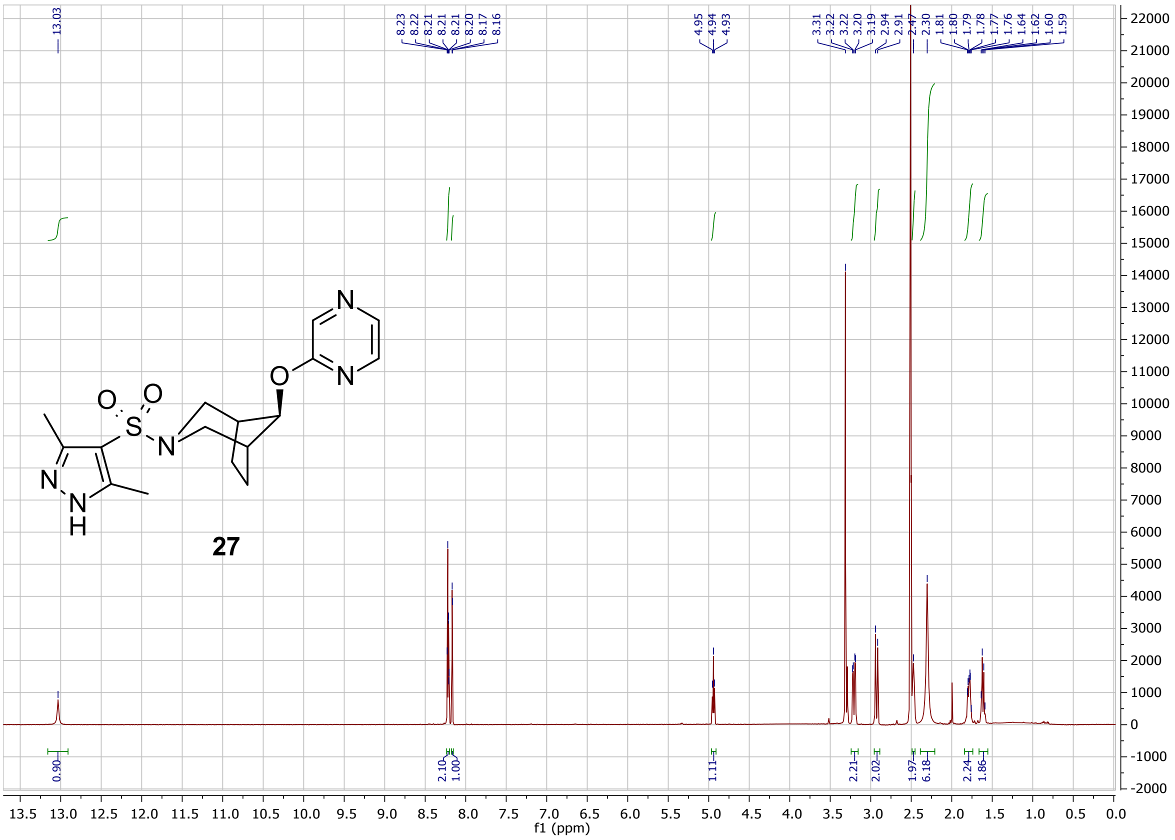
13.03
8.23 8.22 8.21 8.21 8.21 8.20 8.17 8.16
4.95 4.94 4.93
3.31 3.22 3.22 3.20 3.19 2.94 2.91 2.47 2.30 1.81 1.80 1.79 1.78 1.77 1.76 1.64 1.62 1.60 1.59
0.90 2.10 1.00 1.11 2.21 2.02 1.97 6.18 2.24 1.86
27
f1 (ppm)

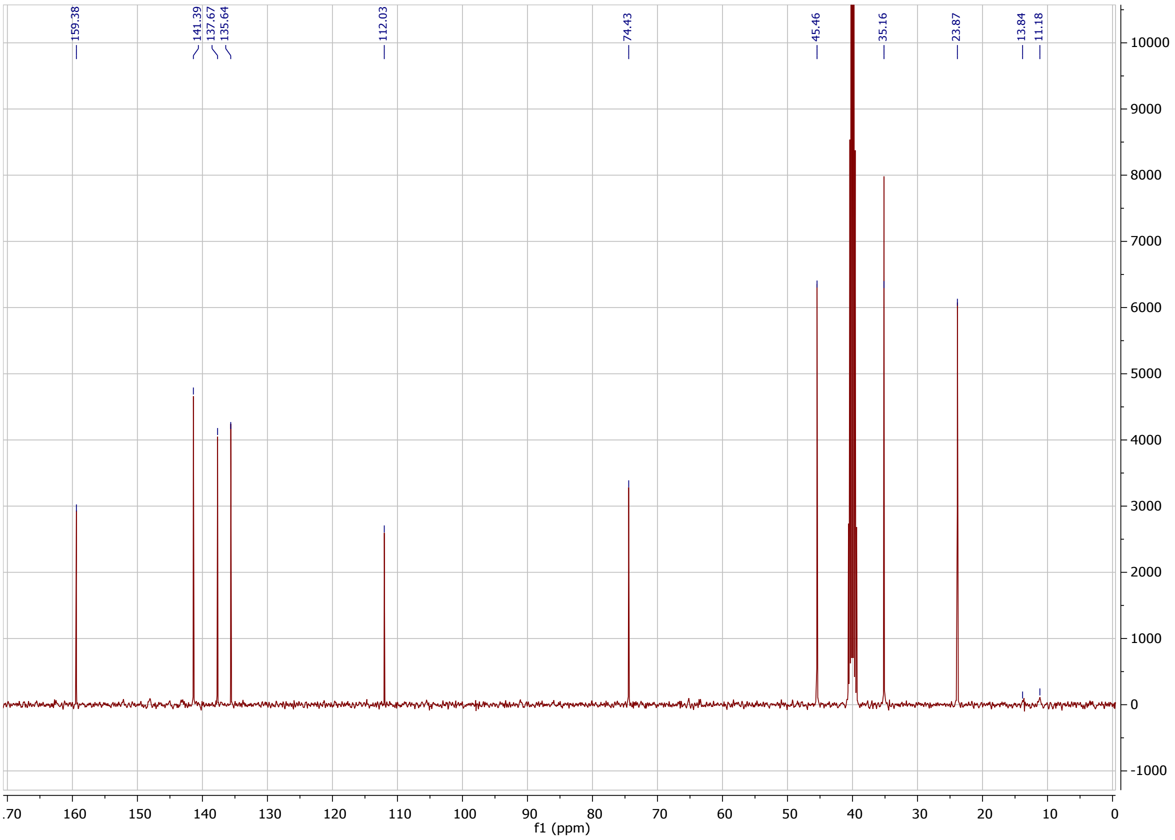
159.38
141.39 137.67 135.64
112.03
74.43
45.46
35.16
23.87
13.84 11.18
f1 (ppm)

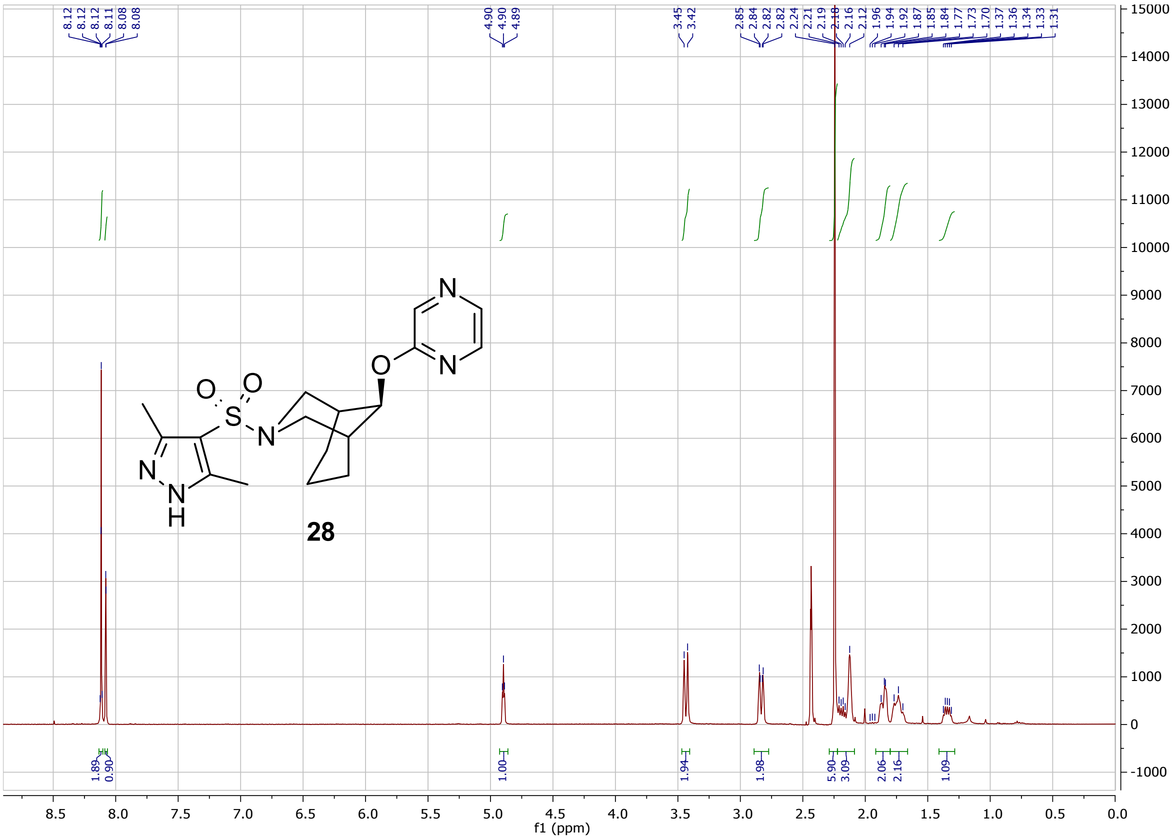

28

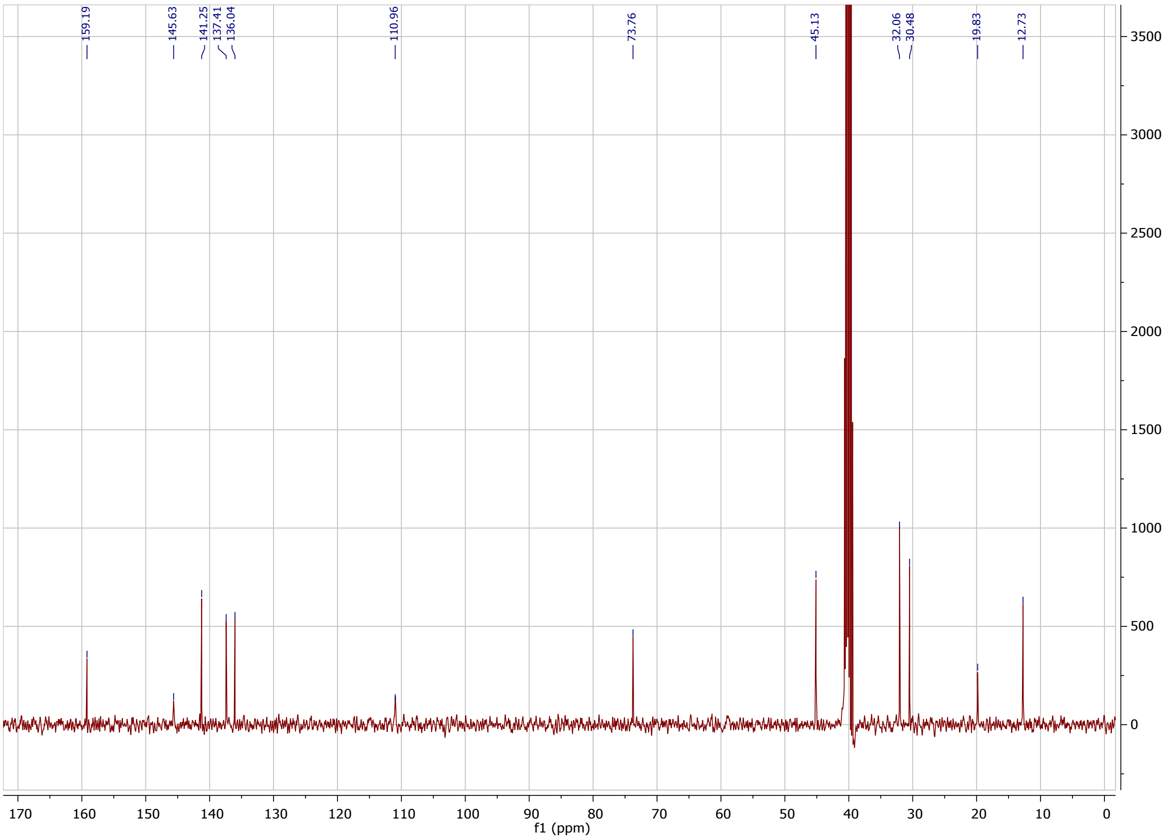

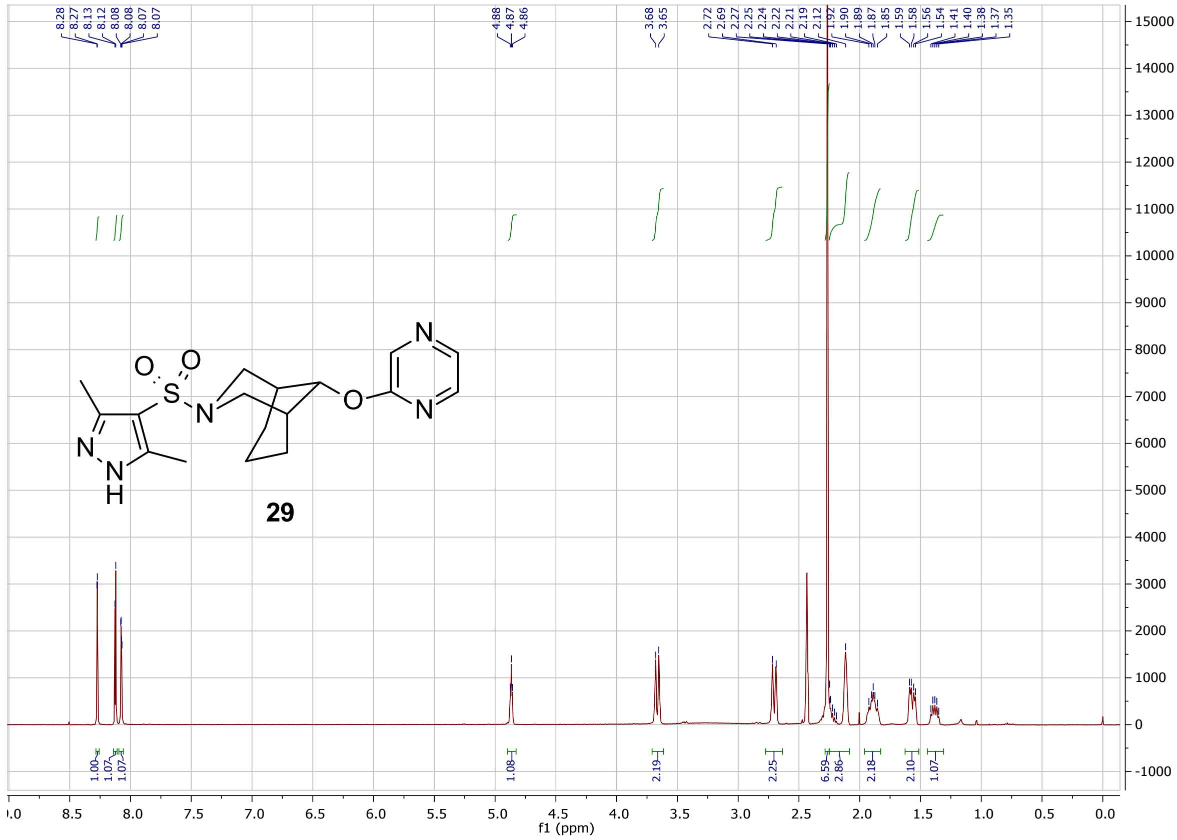
8.28
8.27
8.13
8.12
8.08
8.08
8.07
8.07
4.88
4.87
4.86
3.68
3.65
2.72
2.69
2.27
2.25
2.24
2.22
2.21
2.19
2.12
1.92
1.90
1.89
1.87
1.85
1.59
1.58
1.56
1.54
1.41
1.40
1.38
1.37
1.35
29
1.00
1.07
1.07
1.08
2.19
2.25
6.59
2.86
2.18
2.10
1.07
f1 (ppm)

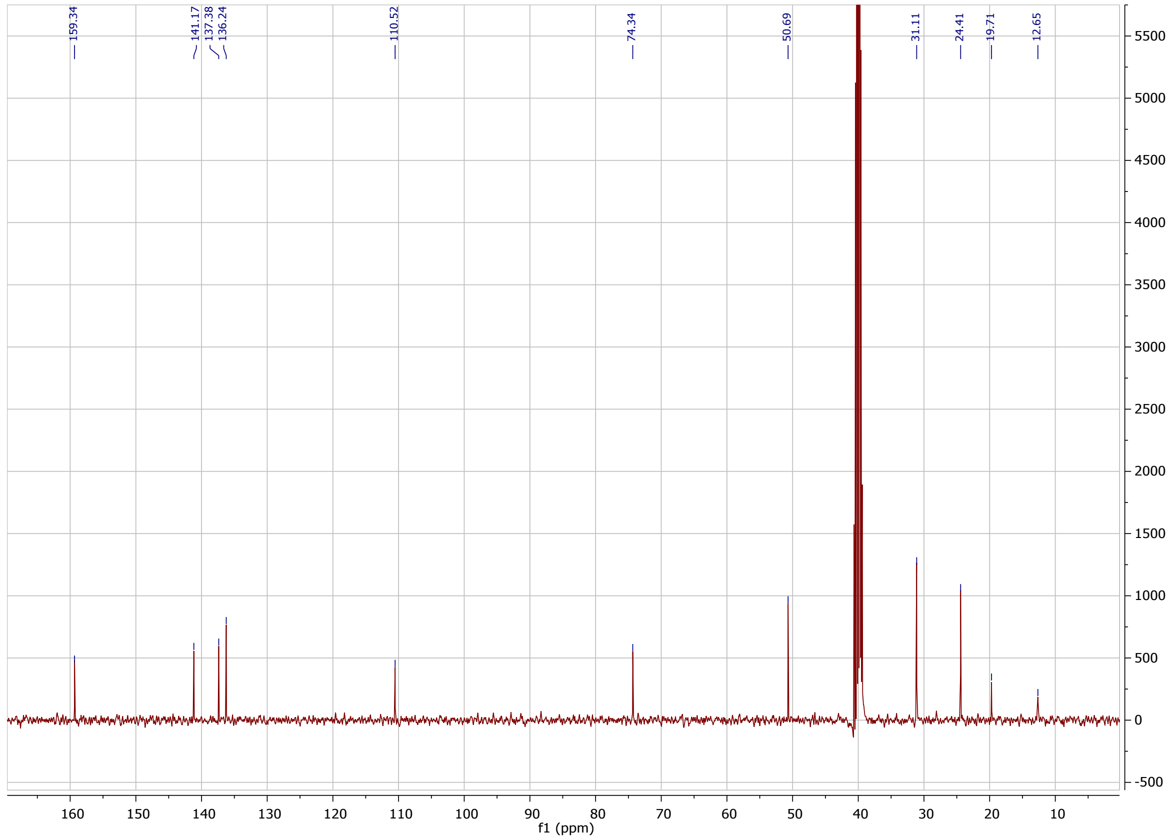
159.34
141.17
137.38
136.24
110.52
74.34
50.69
31.11
24.41
19.71
12.65
f1 (ppm)

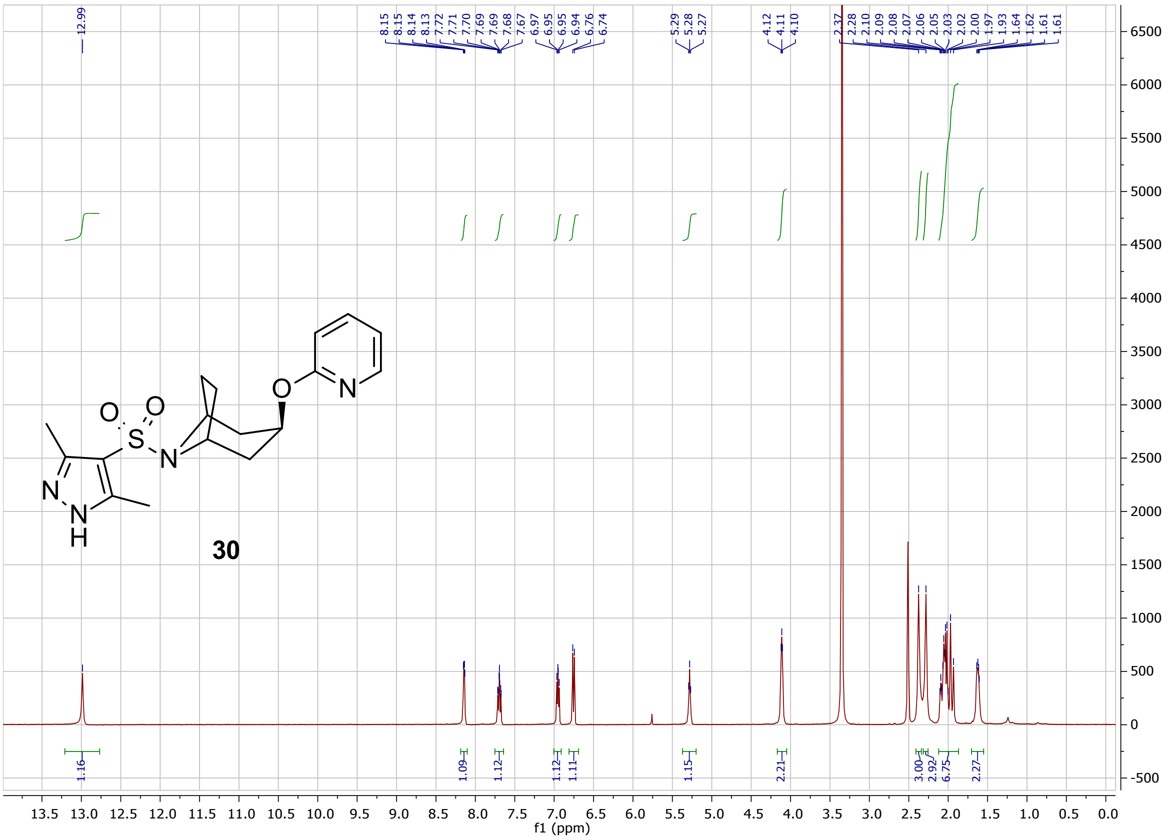
12.99
8.15 8.15 8.14 8.13 7.72 7.71 7.70 7.69 7.69 7.68 7.67 6.97 6.95 6.95 6.94 6.76 6.74
5.29 5.28 5.27
4.12 4.11 4.10
2.37 2.28 2.10 2.09 2.08 2.07 2.06 2.05 2.03 2.02 2.00 1.97 1.93 1.64 1.62 1.61 1.61
1.16 1.09 1.12 1.12 1.11 1.15 2.21 3.00 2.92 6.75 2.27
30
f1 (ppm)

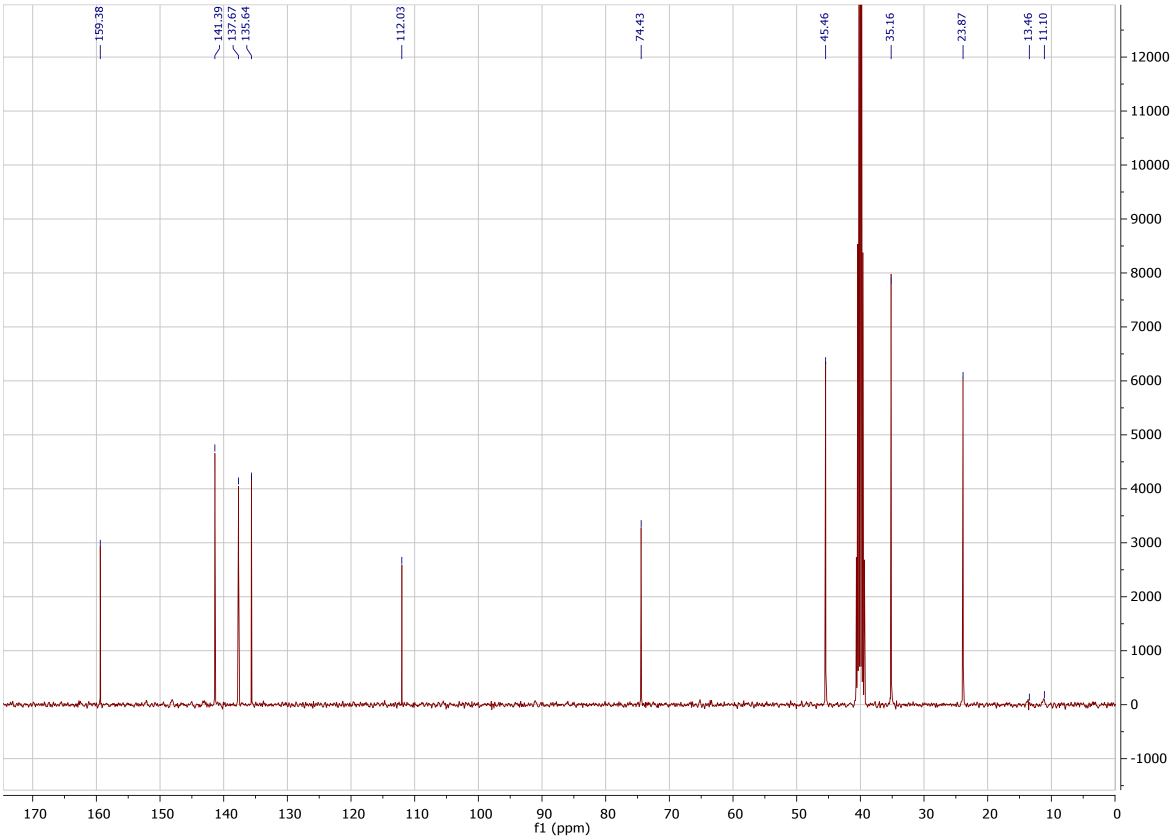
159.38
141.39 137.67 135.64
112.03
74.43
45.46
35.16
23.87
13.46 11.10
f1 (ppm)

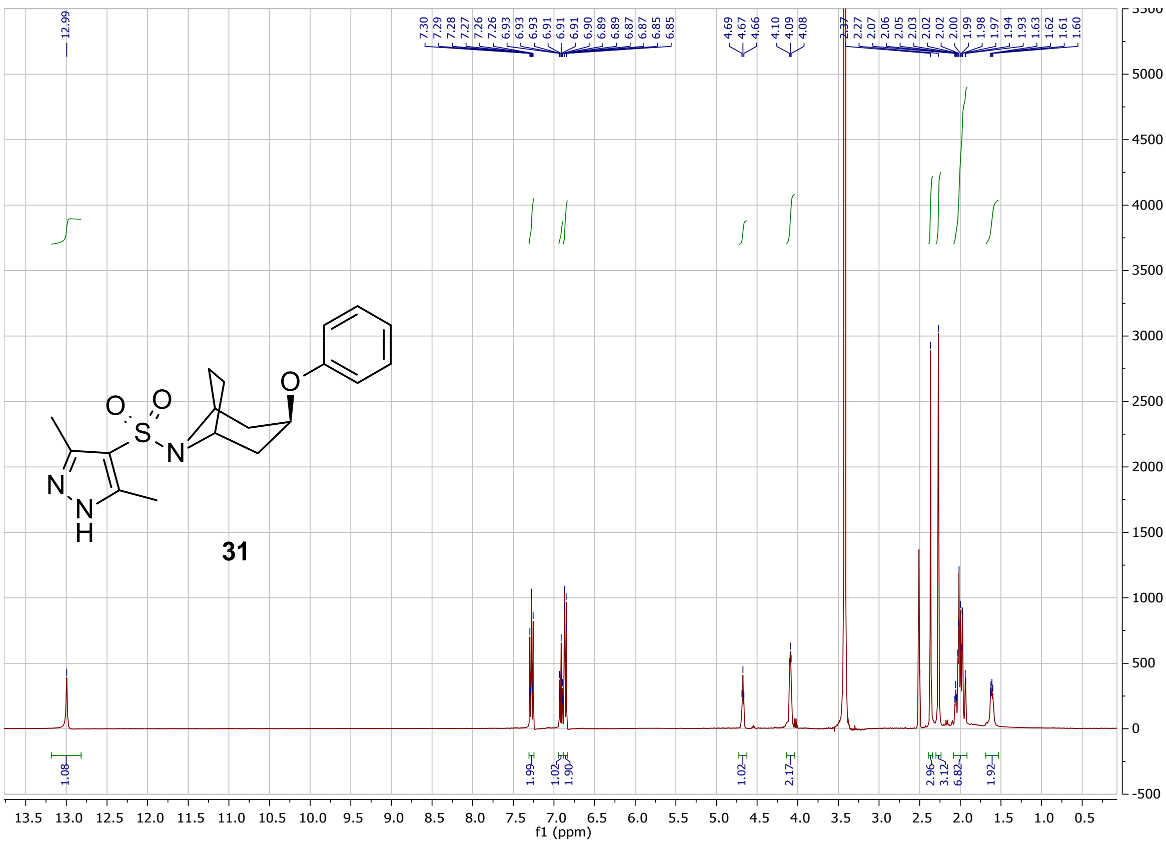

31

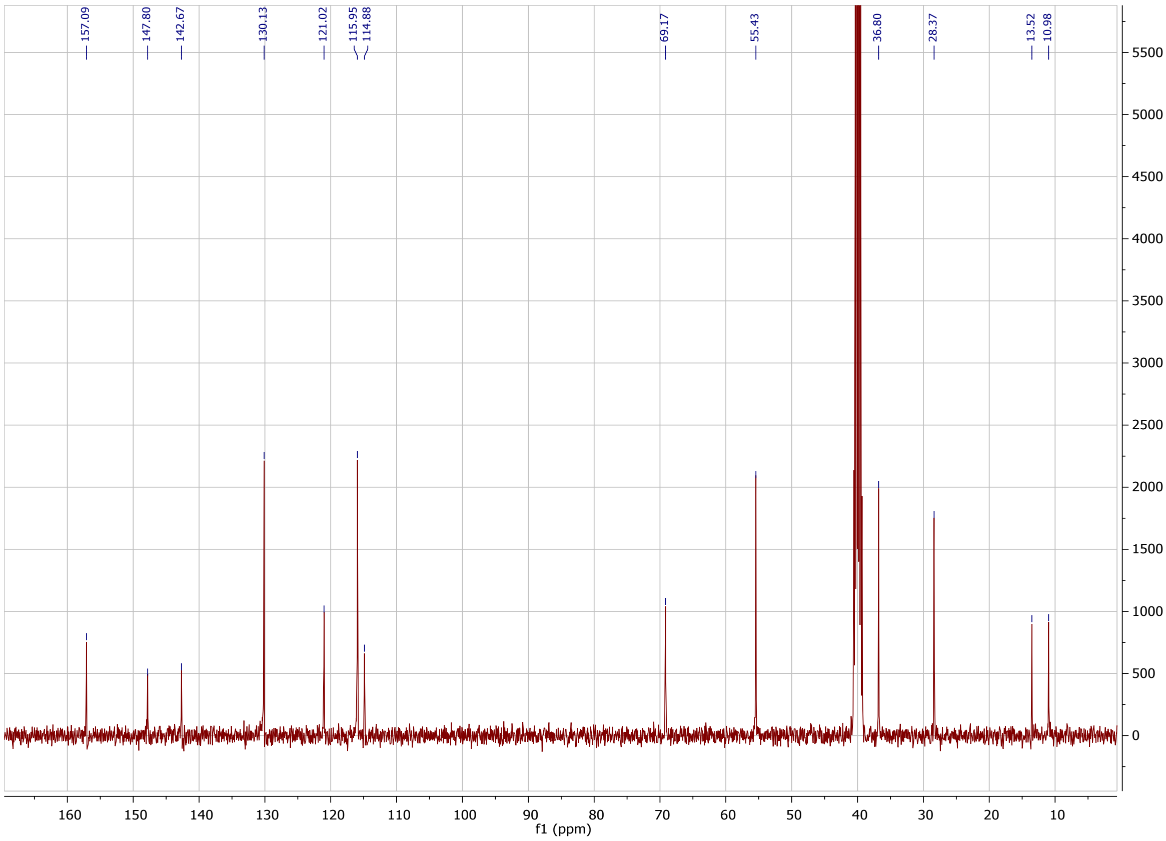

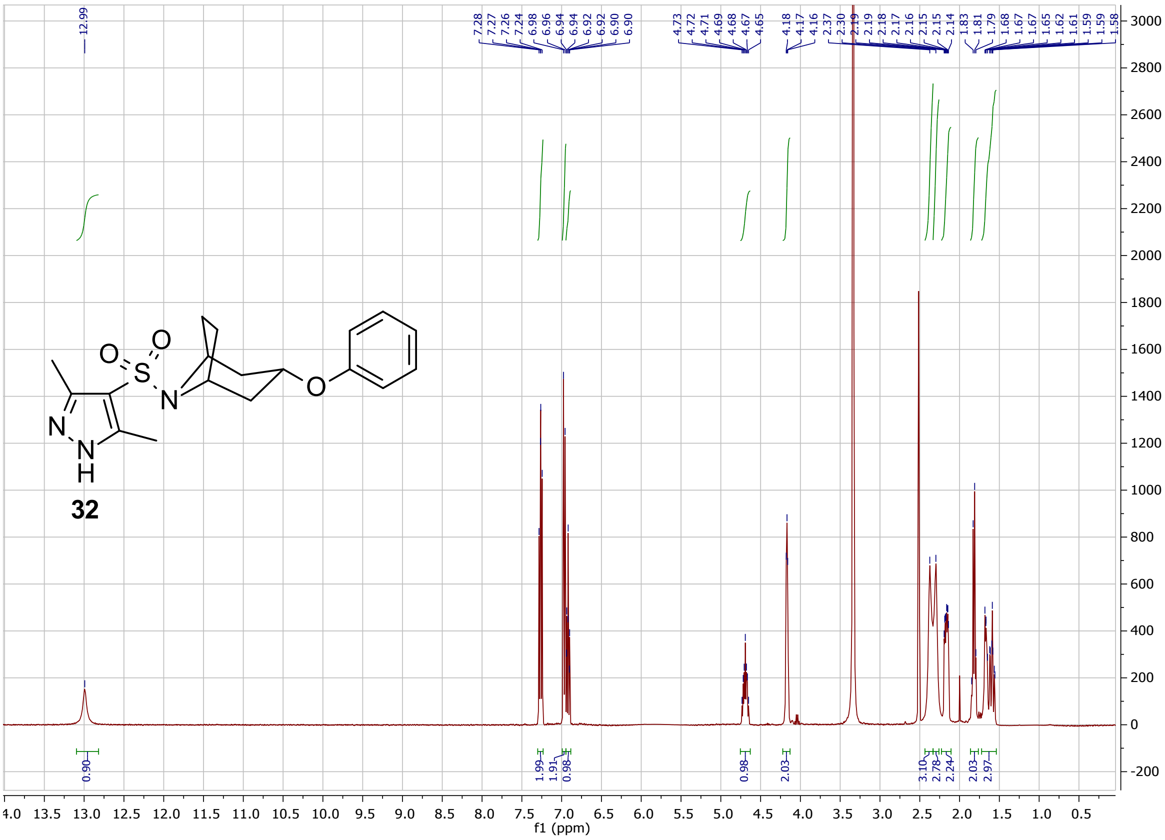
12.99
7.28
7.27
7.26
7.24
6.98
6.96
6.94
6.94
6.92
6.92
6.90
6.90
4.73
4.72
4.71
4.69
4.68
4.67
4.65
4.18
4.17
4.16
2.37
2.30
2.19
2.19
2.18
2.17
2.16
2.15
2.15
2.14
1.83
1.81
1.79
1.68
1.67
1.67
1.65
1.62
1.61
1.59
1.59
1.58
O
O
S
N
O
N
N
H
32
0.90
1.99
1.91
0.98
0.98
2.03
3.10
2.78
2.24
2.03
2.97
f1 (ppm)

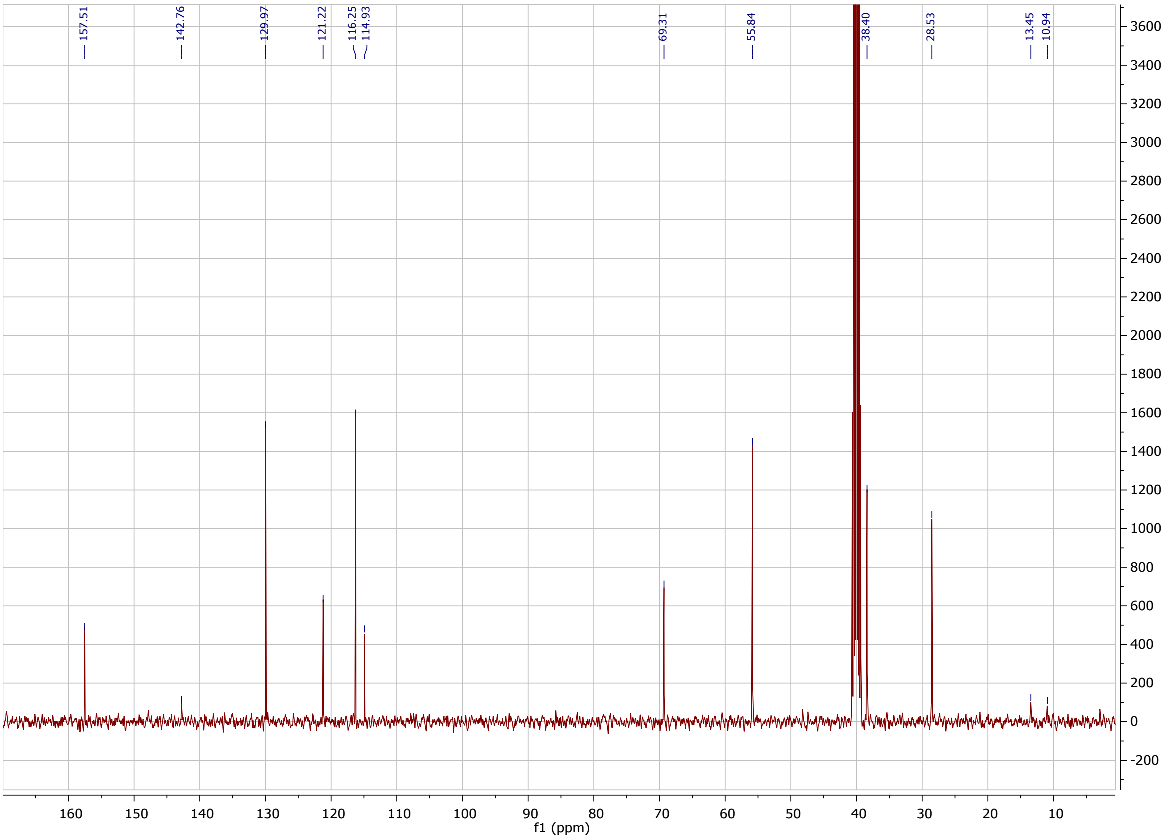
157.51
142.76
129.97
121.22
116.25
114.93
69.31
55.84
38.40
28.53
13.45
10.94
f1 (ppm)

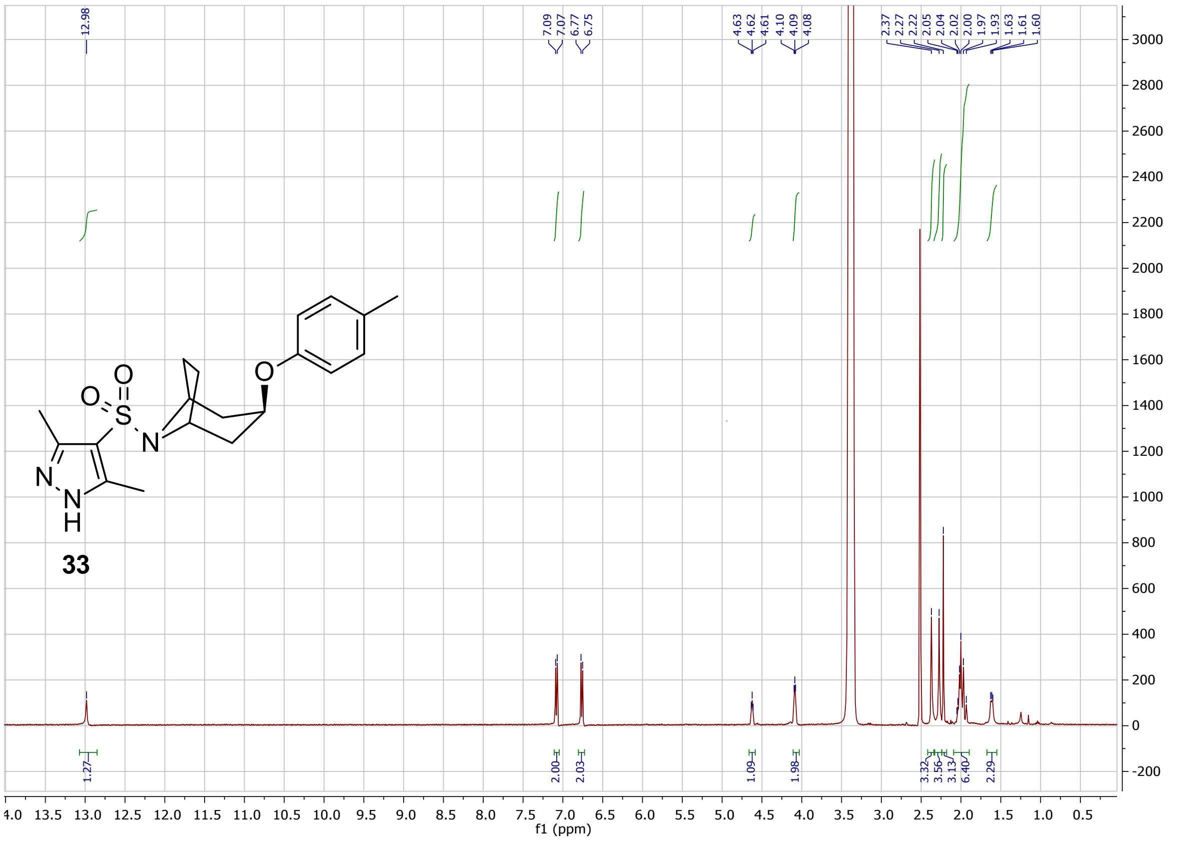
12.98
7.09
7.07
6.77
6.75
4.63
4.62
4.61
4.10
4.09
4.08
2.37
2.27
2.22
2.05
2.04
2.02
2.00
1.97
1.93
1.63
1.61
1.60
1.27
2.00
2.03
1.09
1.98
3.32
3.56
3.13
6.40
2.29
33
f1 (ppm)

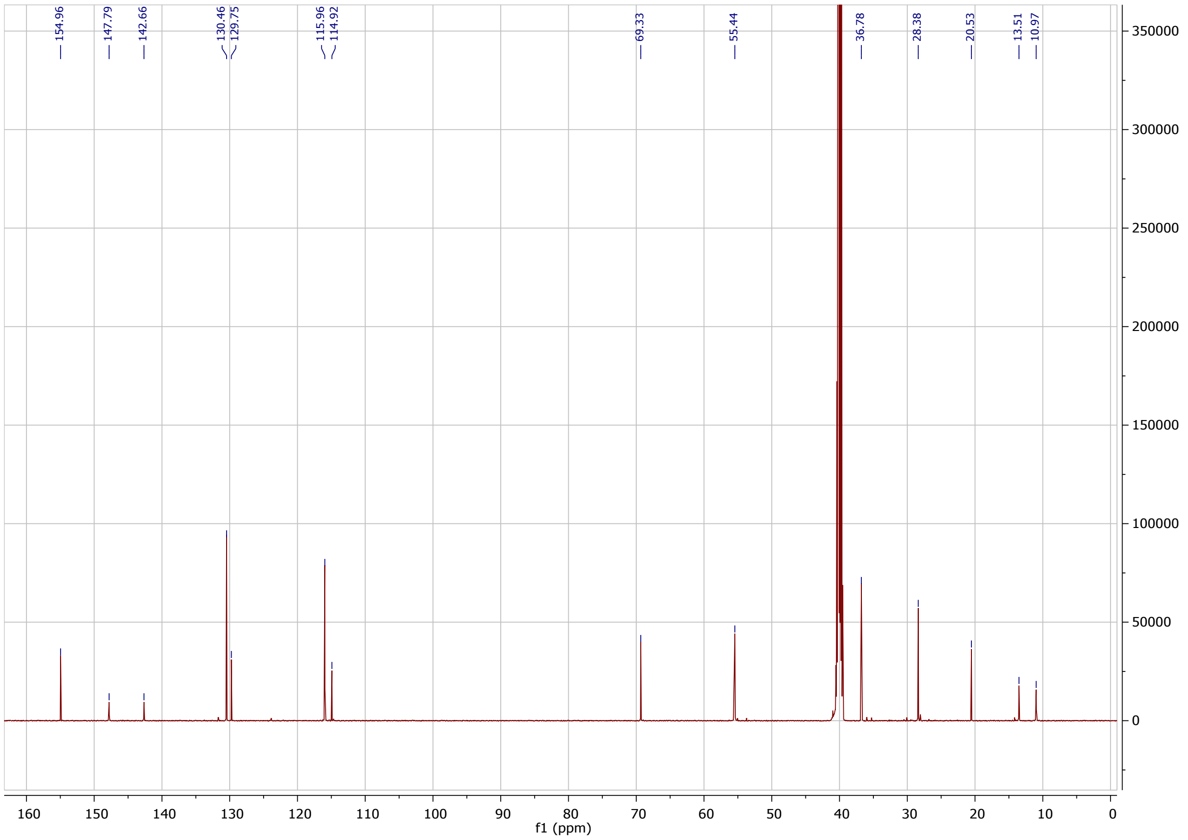
154.96
147.79
142.66
130.46
129.75
115.96
114.92
69.33
55.44
36.78
28.38
20.53
13.51
10.97
f1 (ppm)

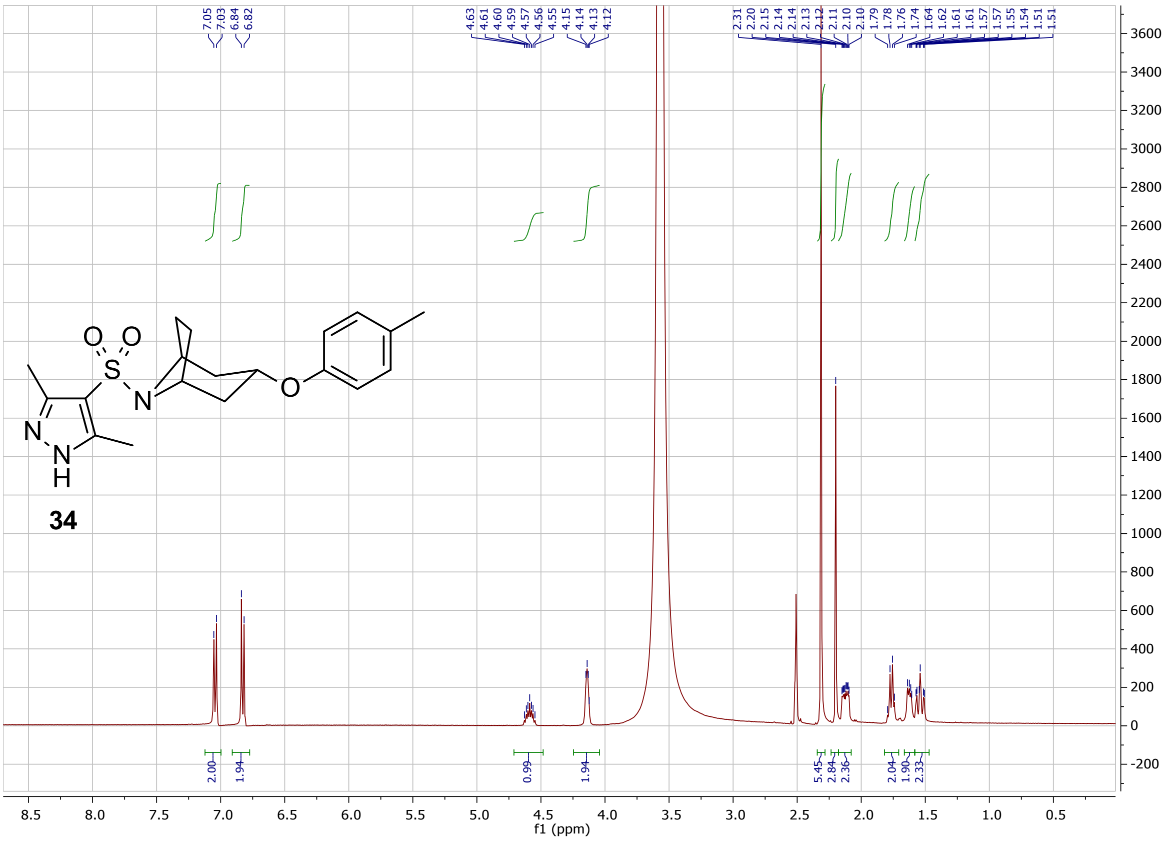
7.05
7.03
6.84
6.82
4.63
4.61
4.60
4.59
4.57
4.56
4.55
4.15
4.14
4.13
4.12
2.31
2.20
2.15
2.14
2.14
2.13
2.12
2.11
2.10
2.10
1.79
1.78
1.76
1.74
1.64
1.62
1.61
1.61
1.57
1.57
1.55
1.54
1.51
1.51
2.00
1.94
0.99
1.94
5.45
2.84
2.36
2.04
1.90
2.33
34
f1 (ppm)

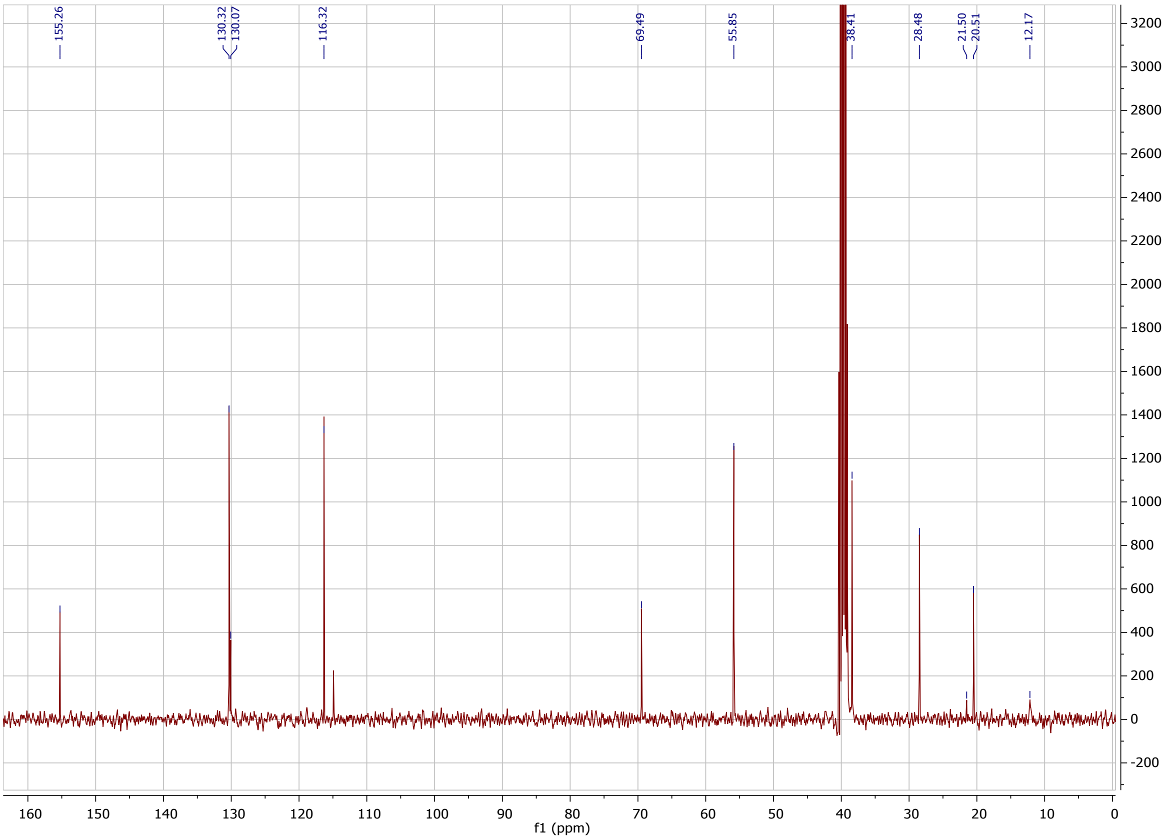
155.26
130.32
130.07
116.32
69.49
55.85
38.41
28.48
21.50
20.51
12.17
f1 (ppm)

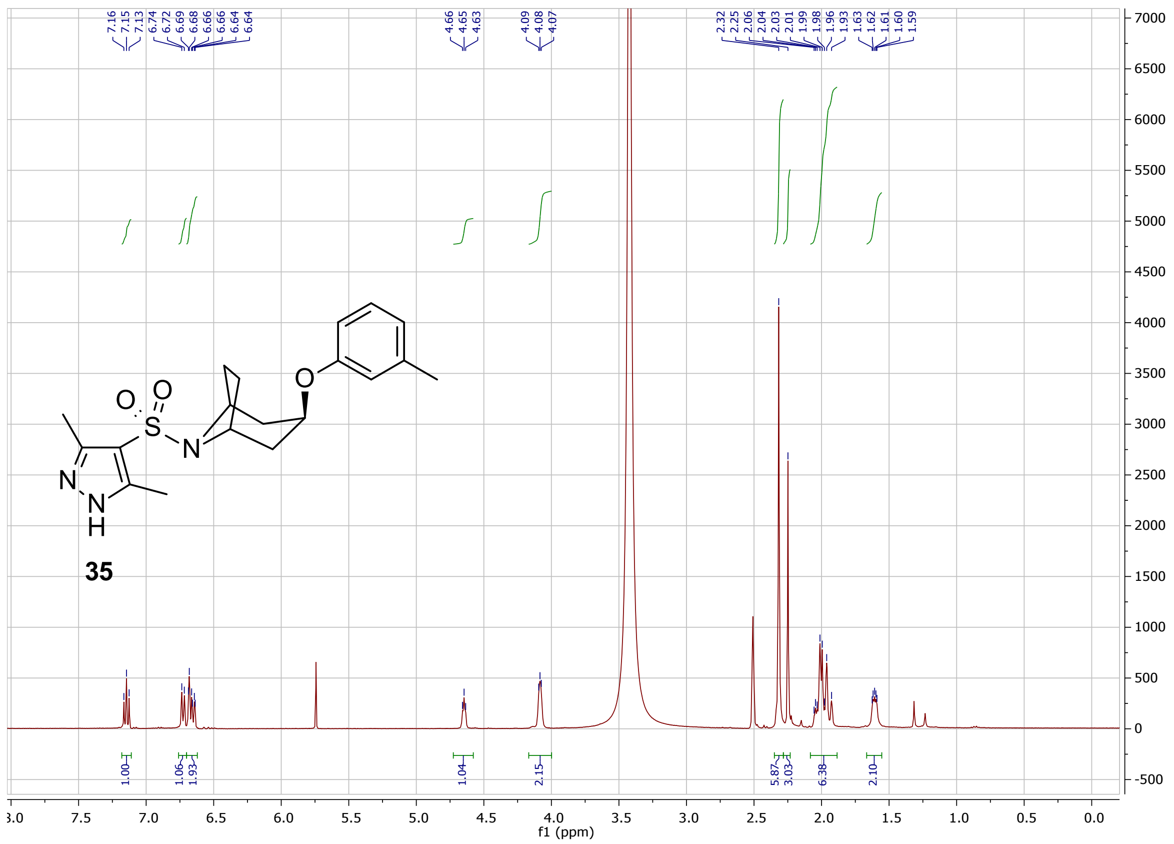
7.16
7.15
7.13
6.74
6.72
6.69
6.68
6.66
6.66
6.64
6.64
4.66
4.65
4.63
4.09
4.08
4.07
2.32
2.25
2.06
2.04
2.03
2.01
1.99
1.98
1.96
1.93
1.63
1.62
1.61
1.60
1.59
35
1.00
1.06
1.93
1.04
2.15
5.87
3.03
6.38
2.10
f1 (ppm)

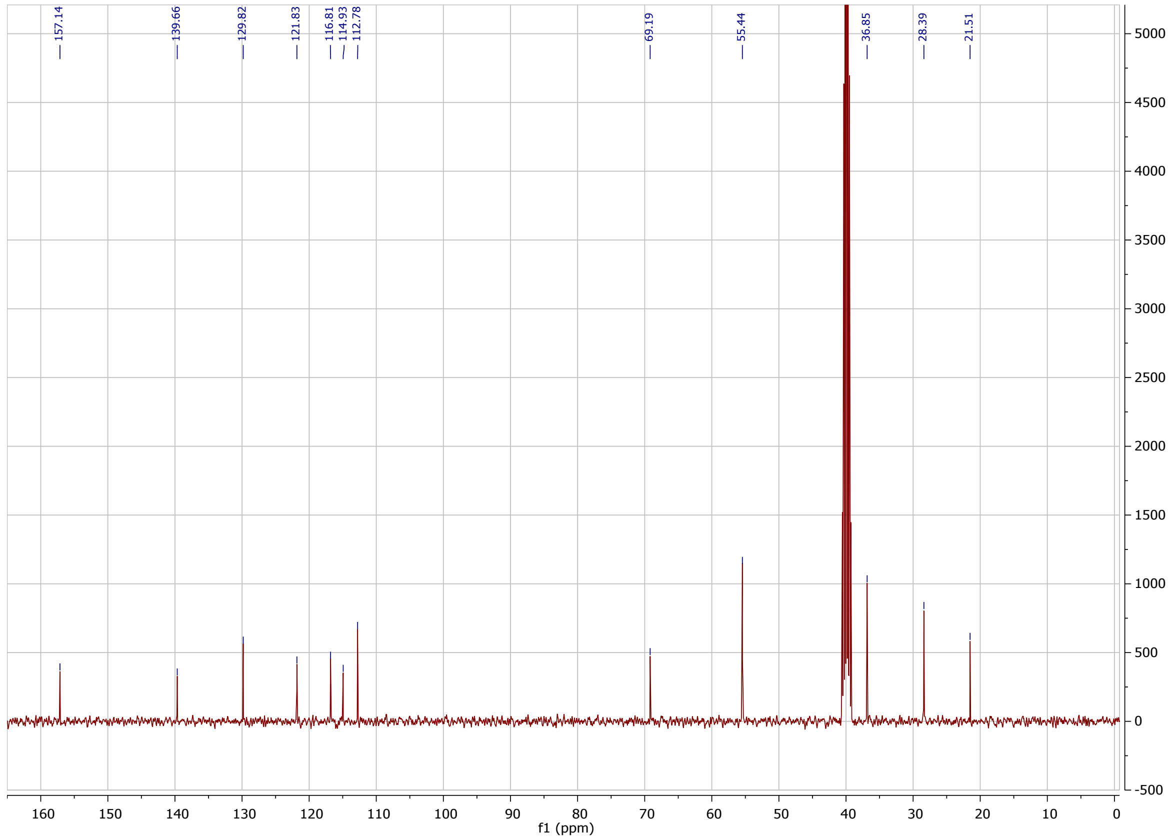
157.14
139.66
129.82
121.83
116.81
114.93
112.78
69.19
55.44
36.85
28.39
21.51
f1 (ppm)

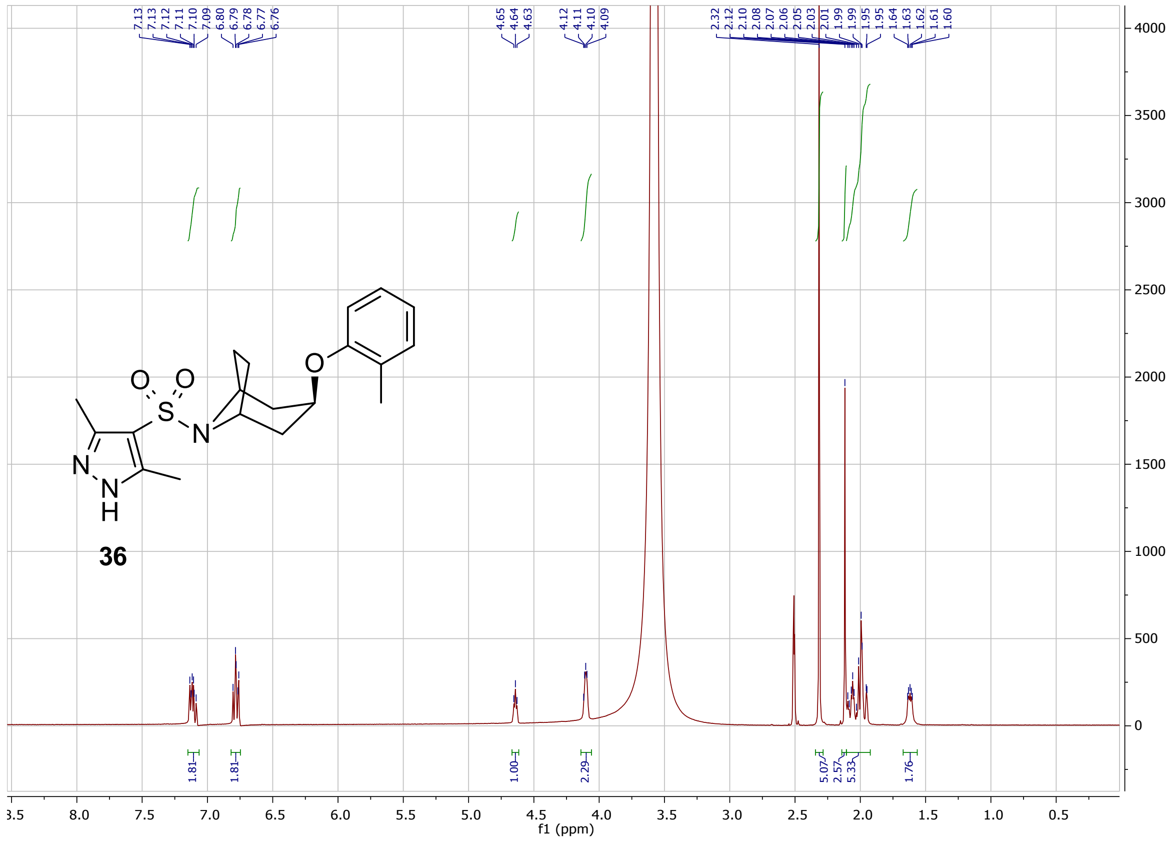
7.13
7.13
7.12
7.11
7.10
7.09
6.80
6.79
6.78
6.77
6.76
4.65
4.64
4.63
4.12
4.11
4.10
4.09
2.32
2.12
2.10
2.08
2.07
2.06
2.05
2.03
2.01
1.99
1.99
1.95
1.95
1.64
1.63
1.62
1.61
1.60
1.81
1.81
1.00
2.29
5.07
2.57
5.33
1.76
O
O
S
N
O
N
N
H
36
f1 (ppm)

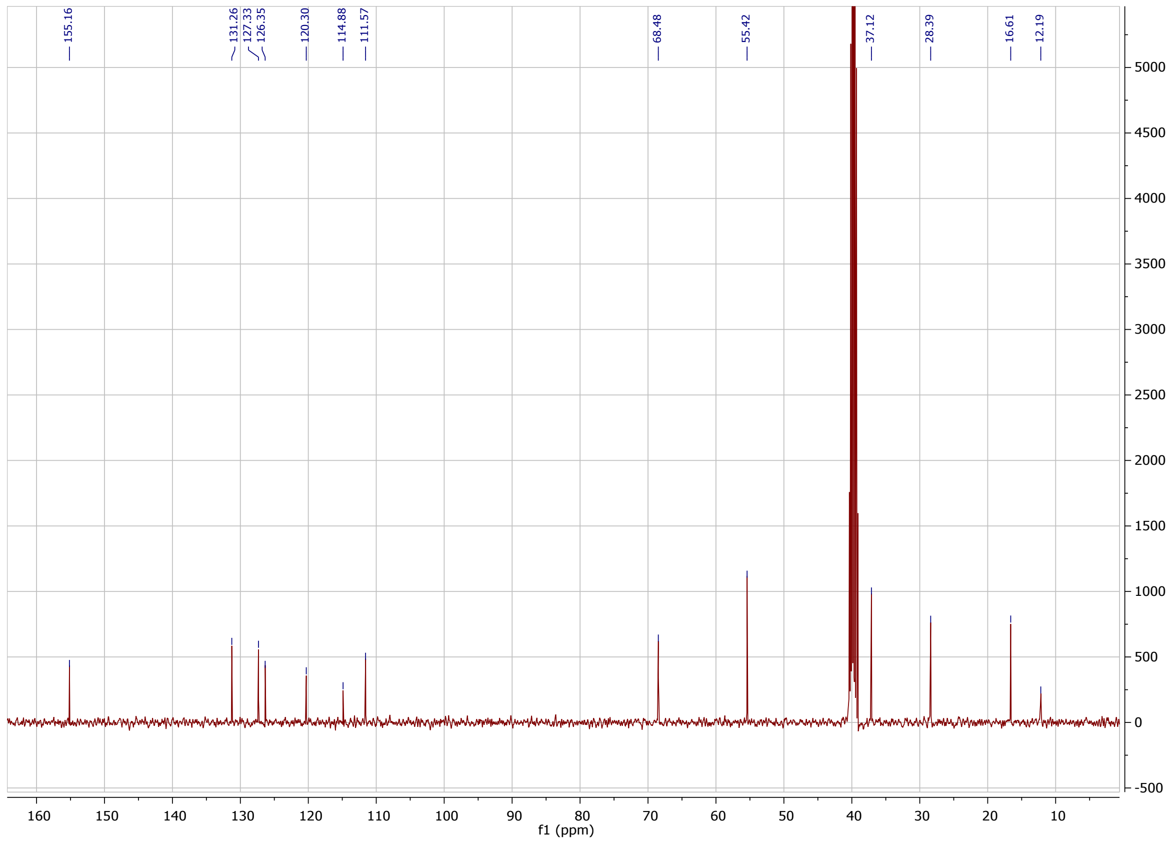
155.16
131.26
127.33
126.35
120.30
114.88
111.57
68.48
55.42
37.12
28.39
16.61
12.19
f1 (ppm)

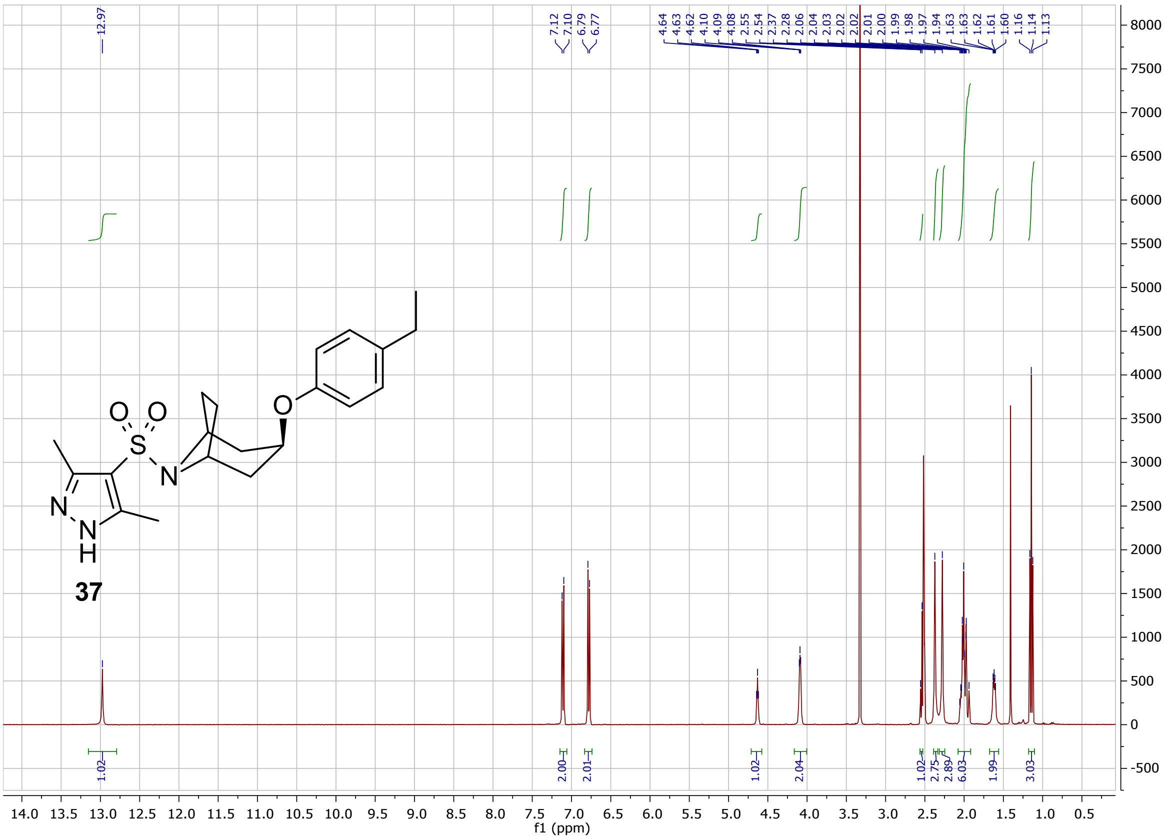

12.97
7.12
7.10
6.79
6.77
4.64
4.63
4.62
4.10
4.09
4.08
2.55
2.54
2.37
2.28
2.06
2.04
2.03
2.02
2.02
2.01
2.00
1.99
1.98
1.97
1.94
1.63
1.63
1.62
1.61
1.60
1.16
1.14
1.13
O O
S
N
O
N
N
H
37
1.02
2.00
2.01
1.02
2.04
1.02
2.75
2.89
6.03
1.99
3.03
f1 (ppm)

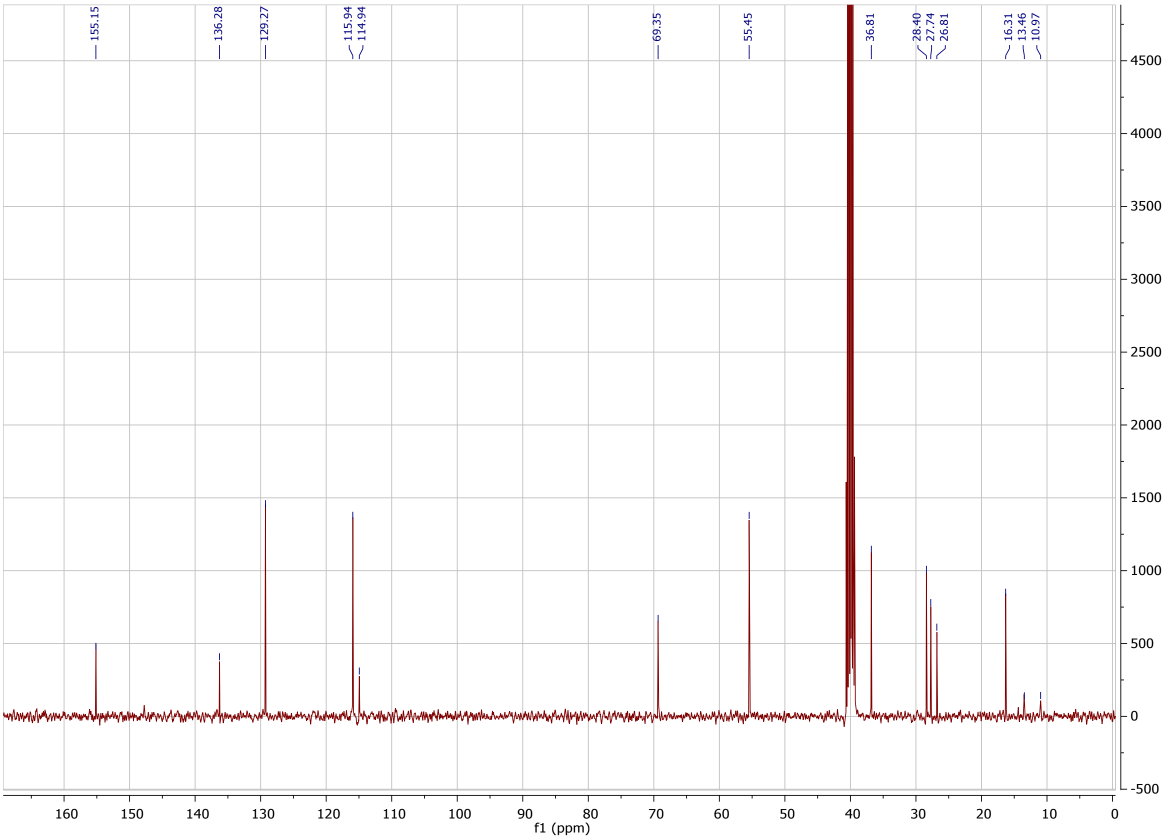

155.15
136.28
129.27
115.94
114.94
69.35
55.45
36.81
28.40
27.74
26.81
16.31
13.46
10.97
f1 (ppm)

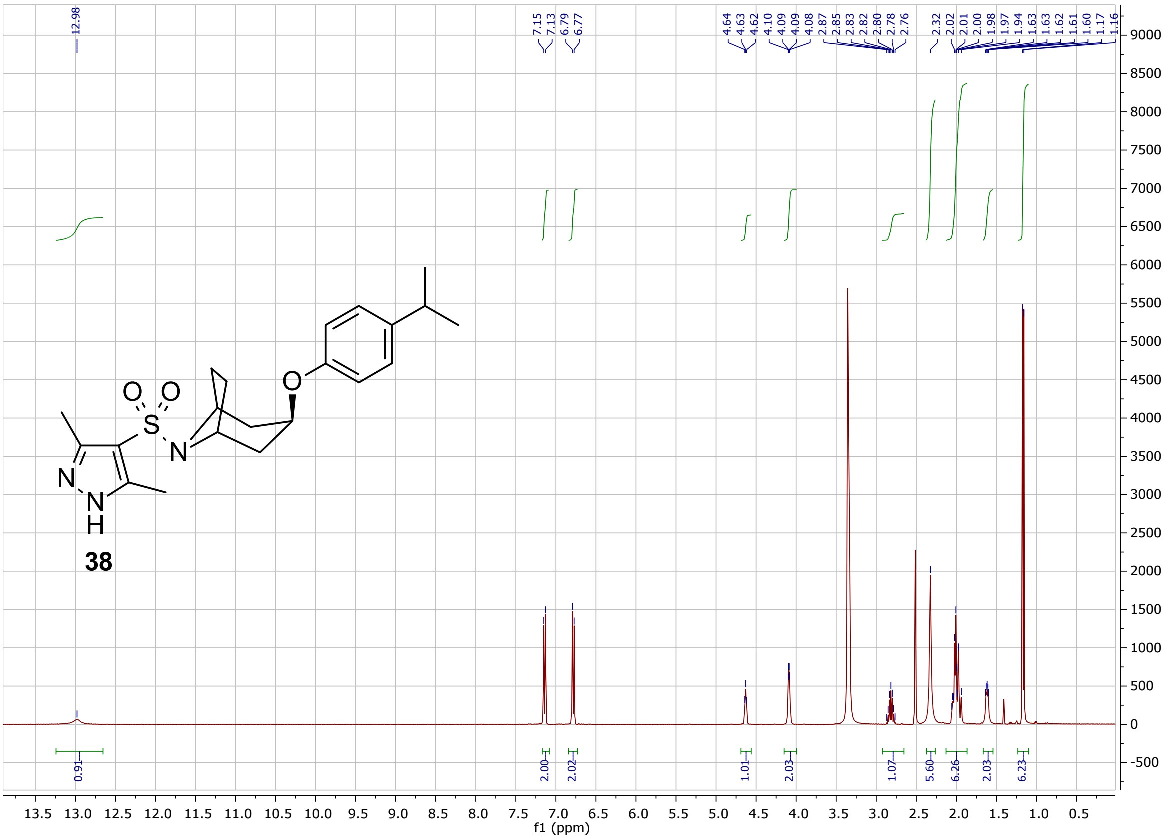
12.98
7.15
7.13
6.79
6.77
4.64
4.63
4.62
4.10
4.09
4.09
4.08
2.87
2.85
2.83
2.82
2.80
2.78
2.76
2.32
2.02
2.01
2.00
1.98
1.97
1.94
1.63
1.63
1.62
1.61
1.60
1.17
1.16
0.91
2.00
2.02
1.01
2.03
1.07
5.60
6.26
2.03
6.23
38
f1 (ppm)

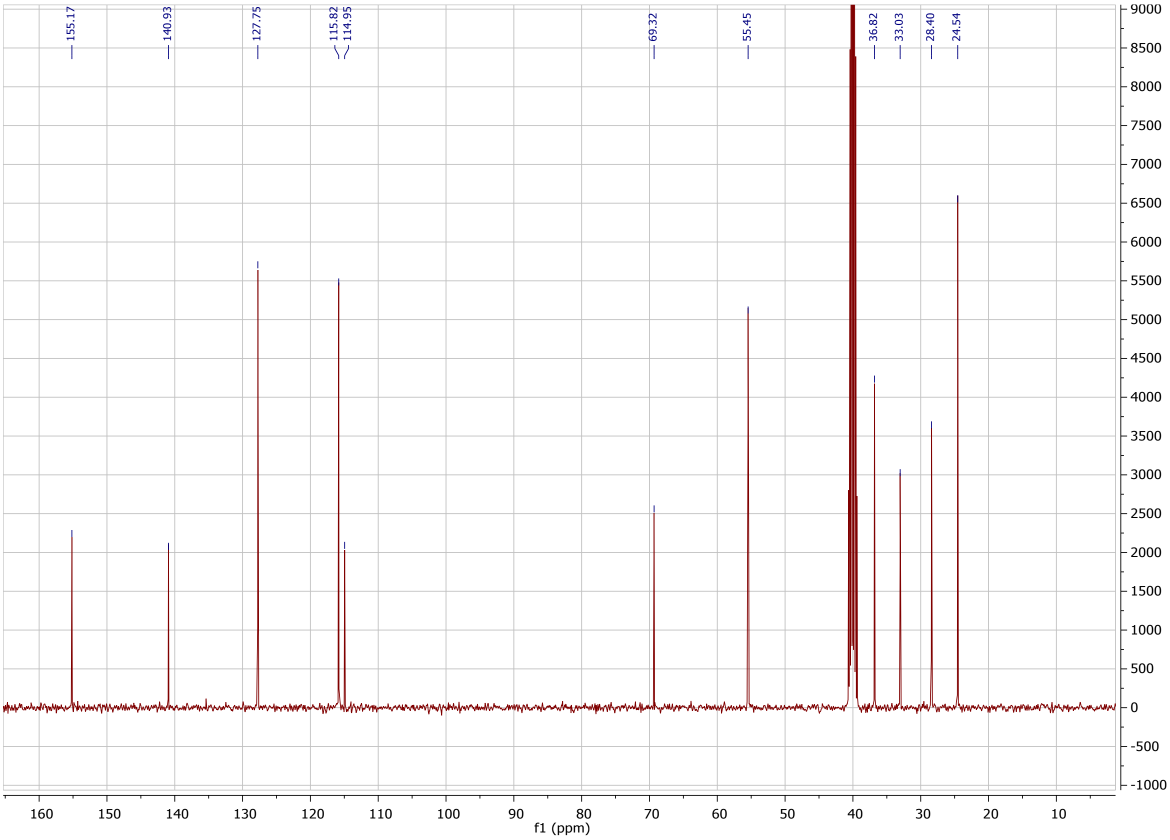
155.17
140.93
127.75
115.82
114.95
69.32
55.45
36.82
33.03
28.40
24.54
f1 (ppm)

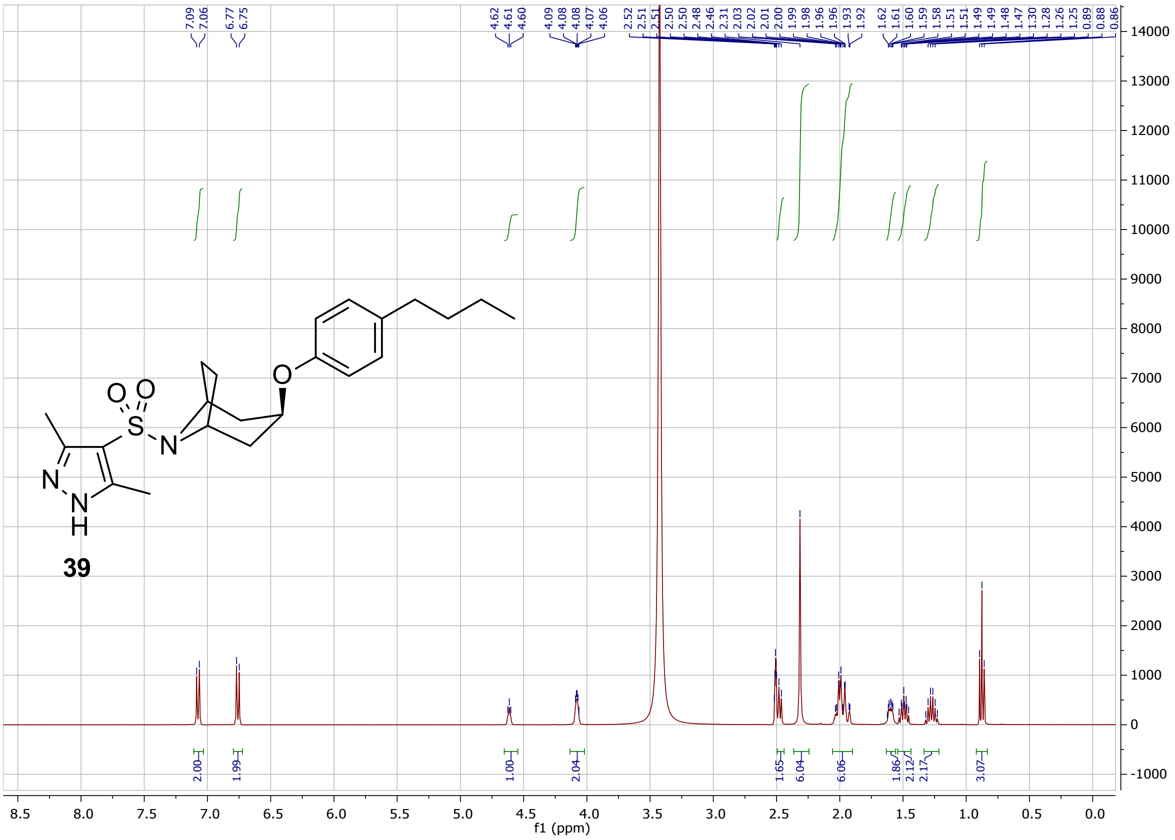
39
f1 (ppm)

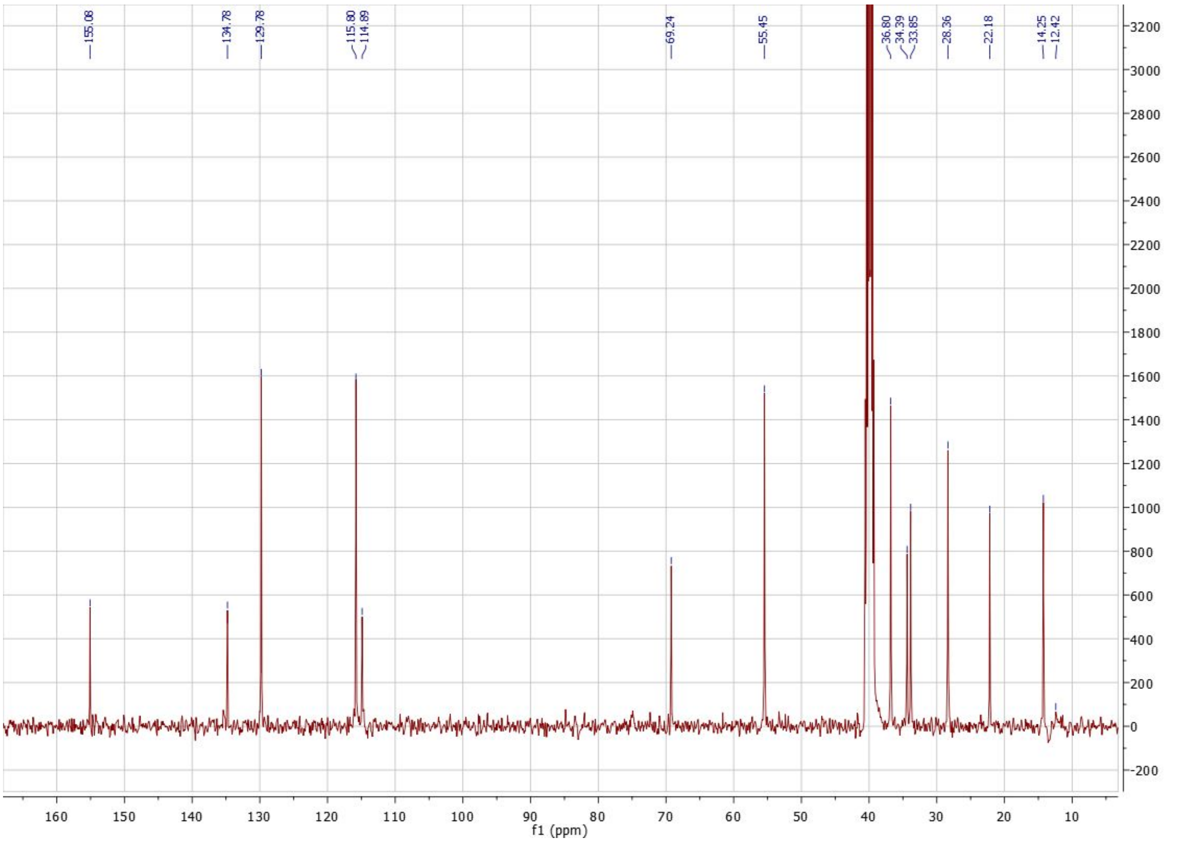
f1 (ppm)

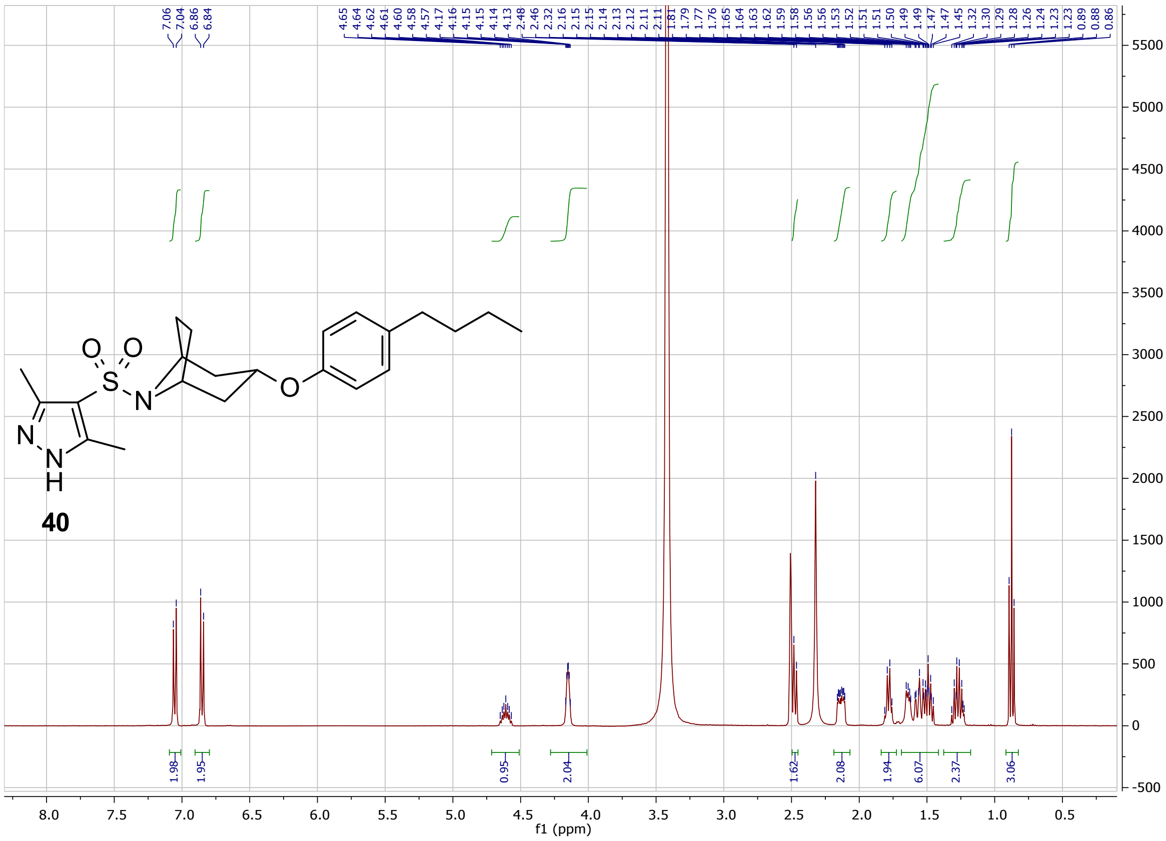
7.06 7.04 6.86 6.84
4.65 4.64 4.62 4.61 4.60 4.58 4.57 4.17 4.16 4.15 4.15 4.14 4.13 2.48 2.46 2.32 2.16 2.15 2.15 2.14 2.13 2.12 2.11 2.11 1.81 1.79 1.77 1.76 1.65 1.64 1.63 1.62 1.59 1.58 1.56 1.56 1.53 1.52 1.51 1.51 1.50 1.49 1.49 1.47 1.47 1.45 1.32 1.30 1.29 1.28 1.26 1.24 1.23 1.23 0.89 0.88 0.86
1.98 1.95 0.95 2.04 1.62 2.08 1.94 6.07 2.37 3.06
40
f1 (ppm)

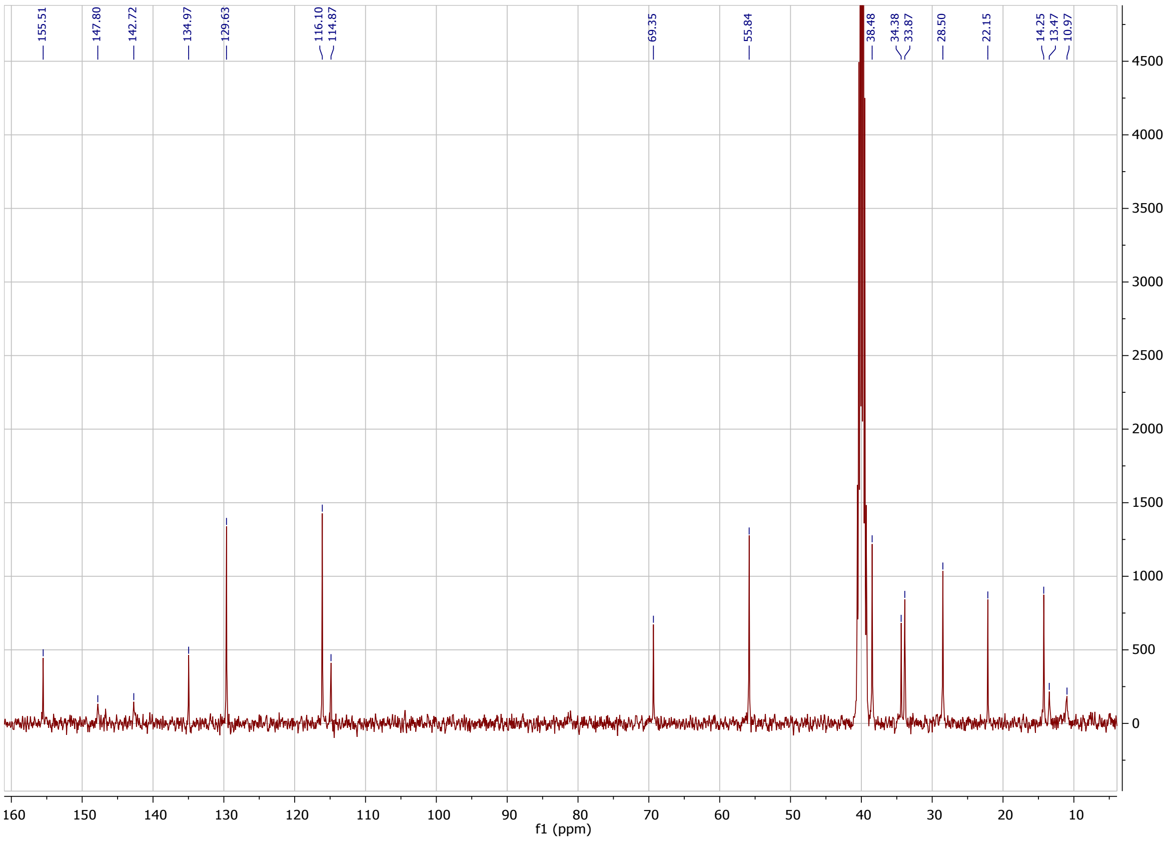
155.51 147.80 142.72 134.97 129.63 116.10 114.87 69.35 55.84 38.48 34.38 33.87 28.50 22.15 14.25 13.47 10.97
f1 (ppm)

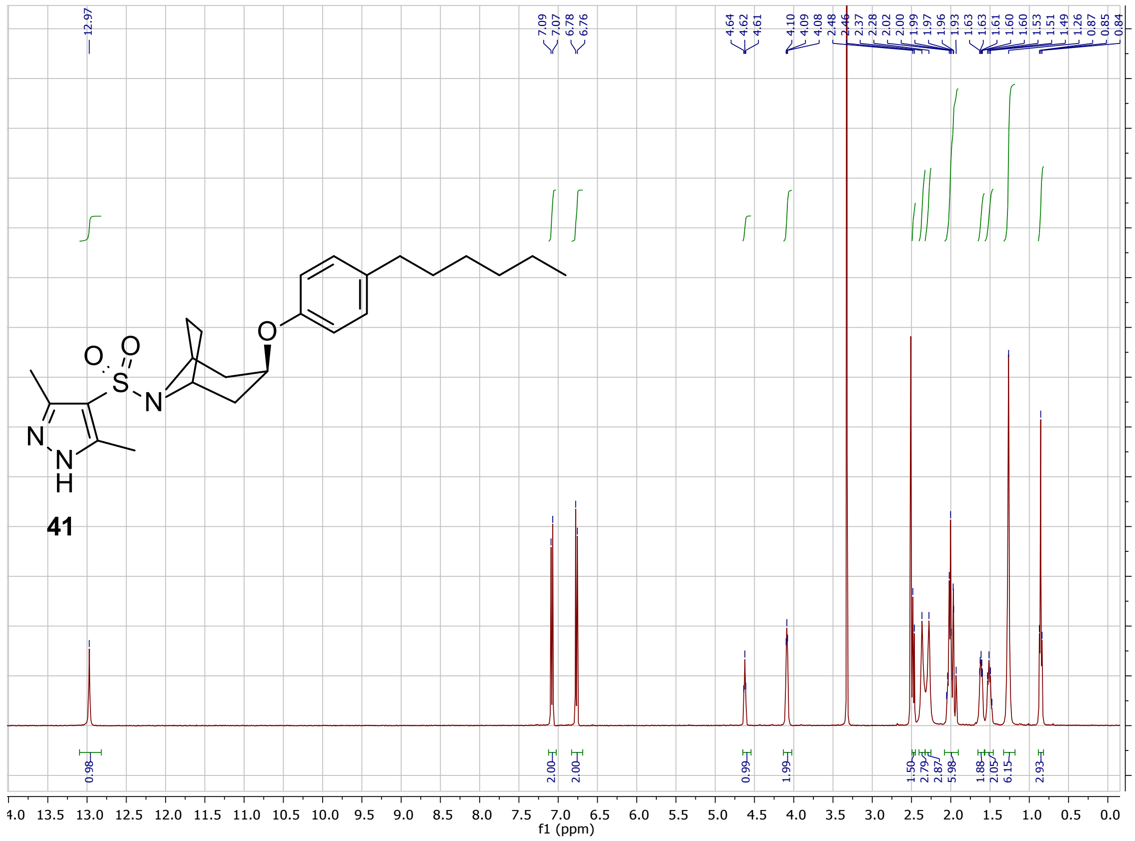
41
f1 (ppm)

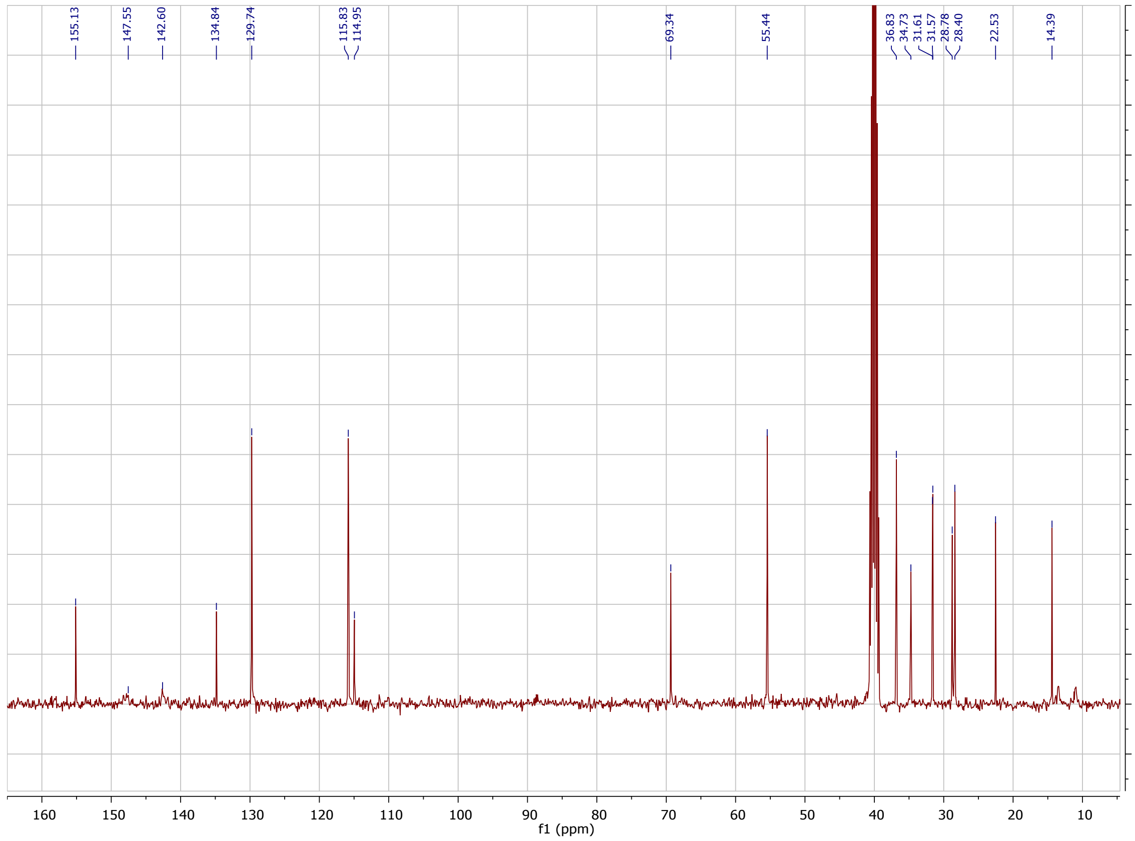
f1 (ppm)

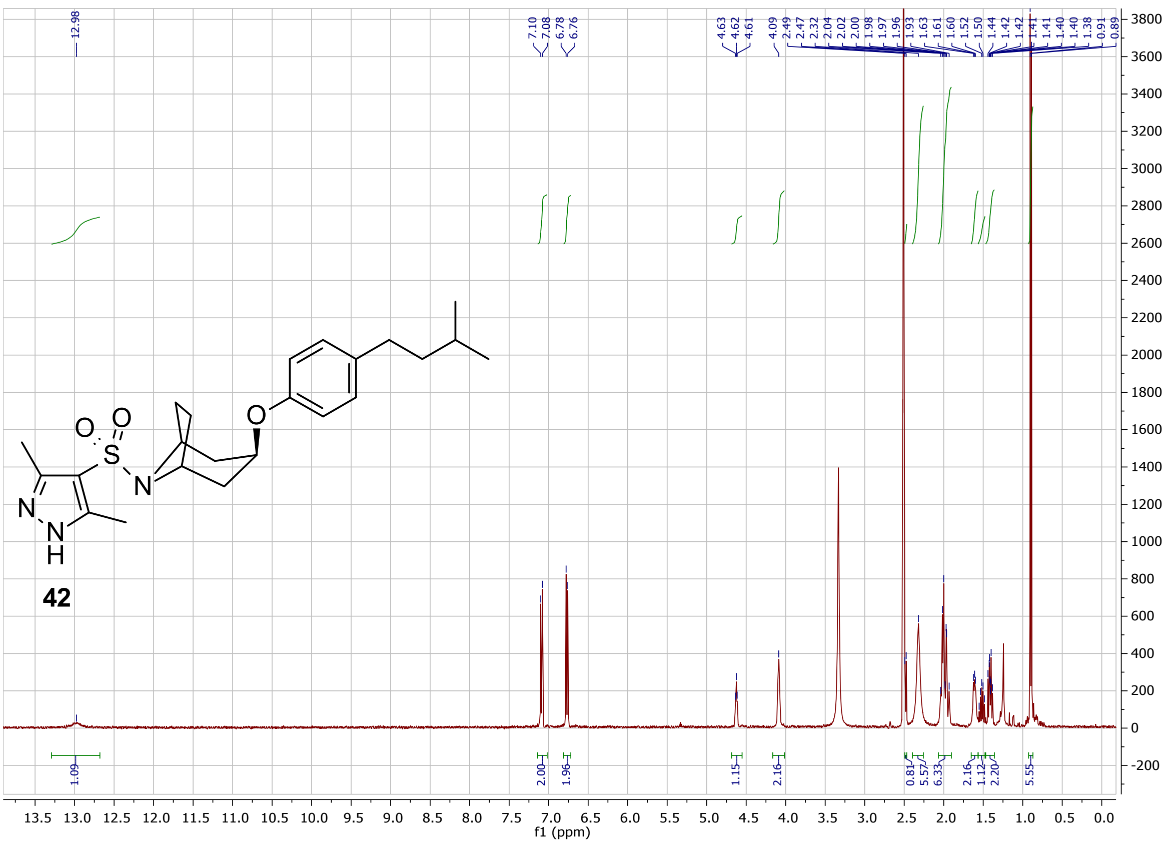

42

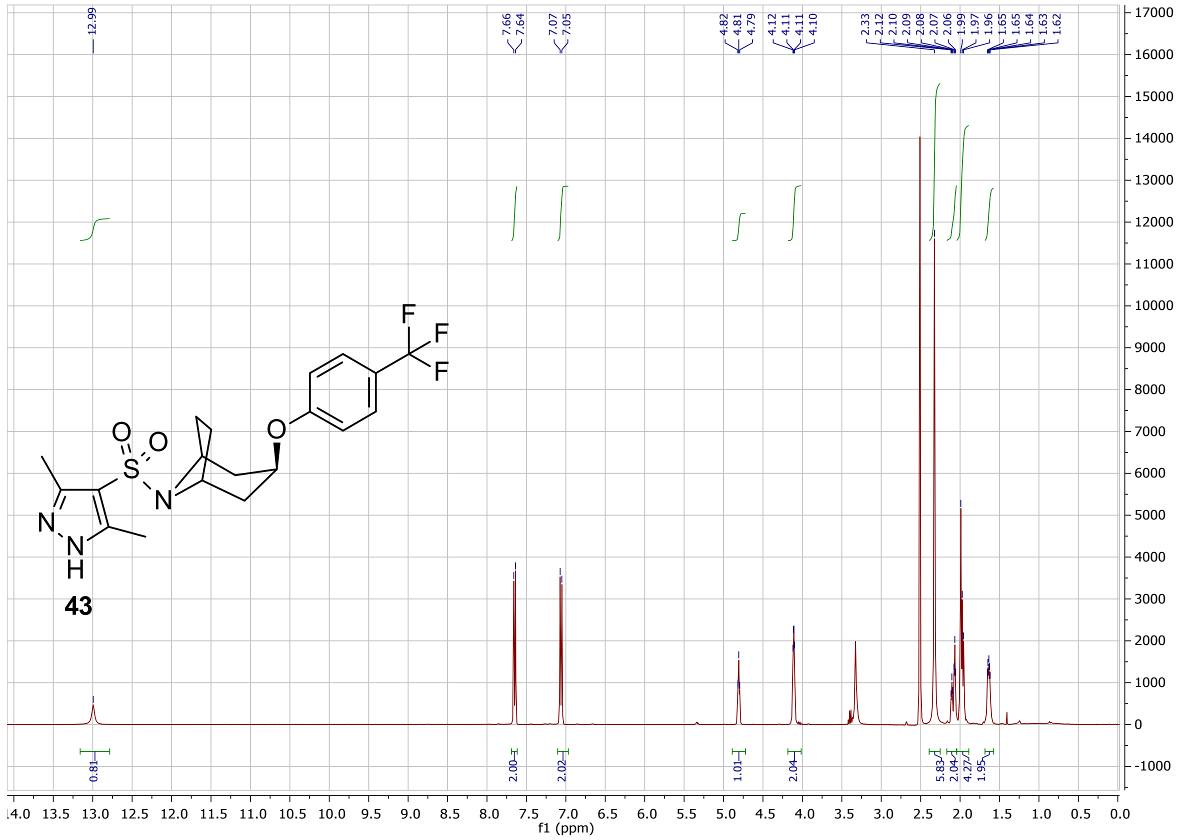

12.99
7.66
7.64
7.07
7.05
4.82
4.81
4.79
4.12
4.11
4.11
4.10
2.33
2.12
2.10
2.09
2.08
2.07
2.06
1.99
1.97
1.96
1.65
1.65
1.64
1.63
1.62
0.81
2.00
2.02
1.01
2.04
5.83
2.04
4.27
1.95
43
f1 (ppm)

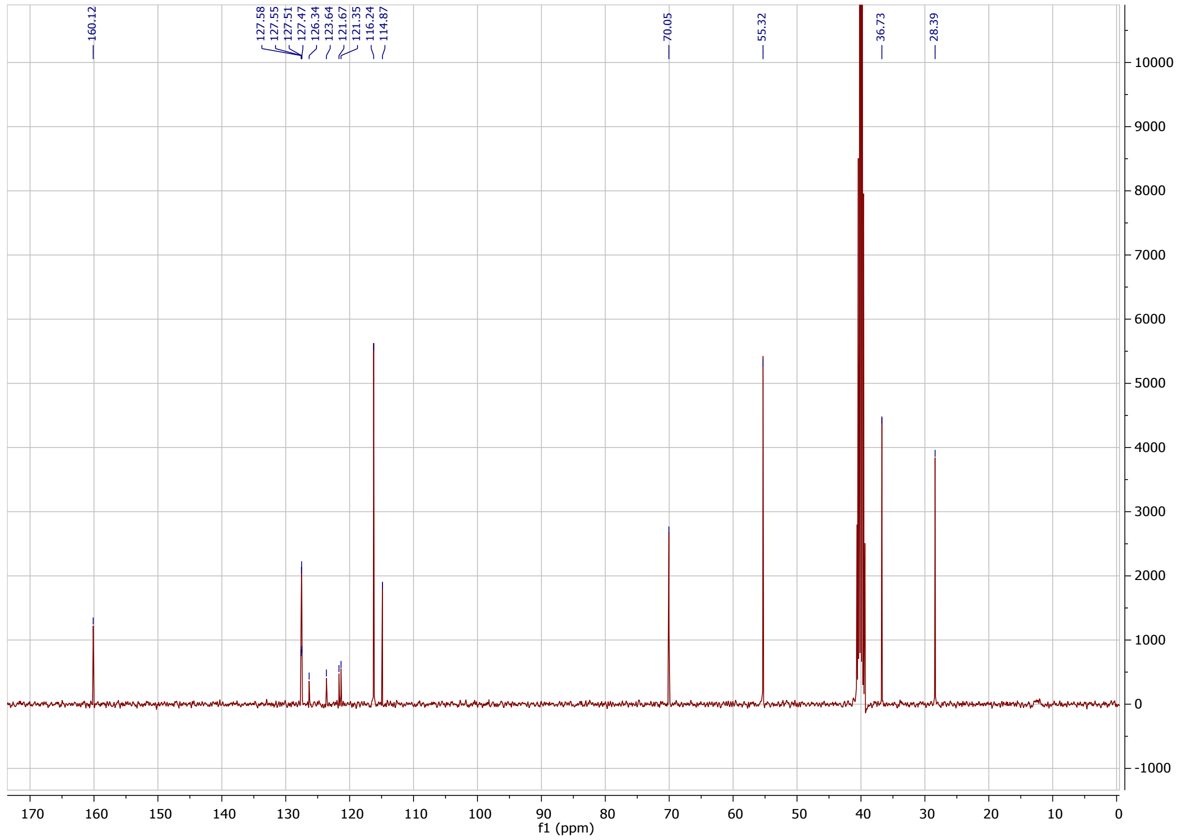

160.12
127.58
127.55
127.51
127.47
126.34
123.64
121.67
121.35
116.24
114.87
70.05
55.32
36.73
28.39
f1 (ppm)

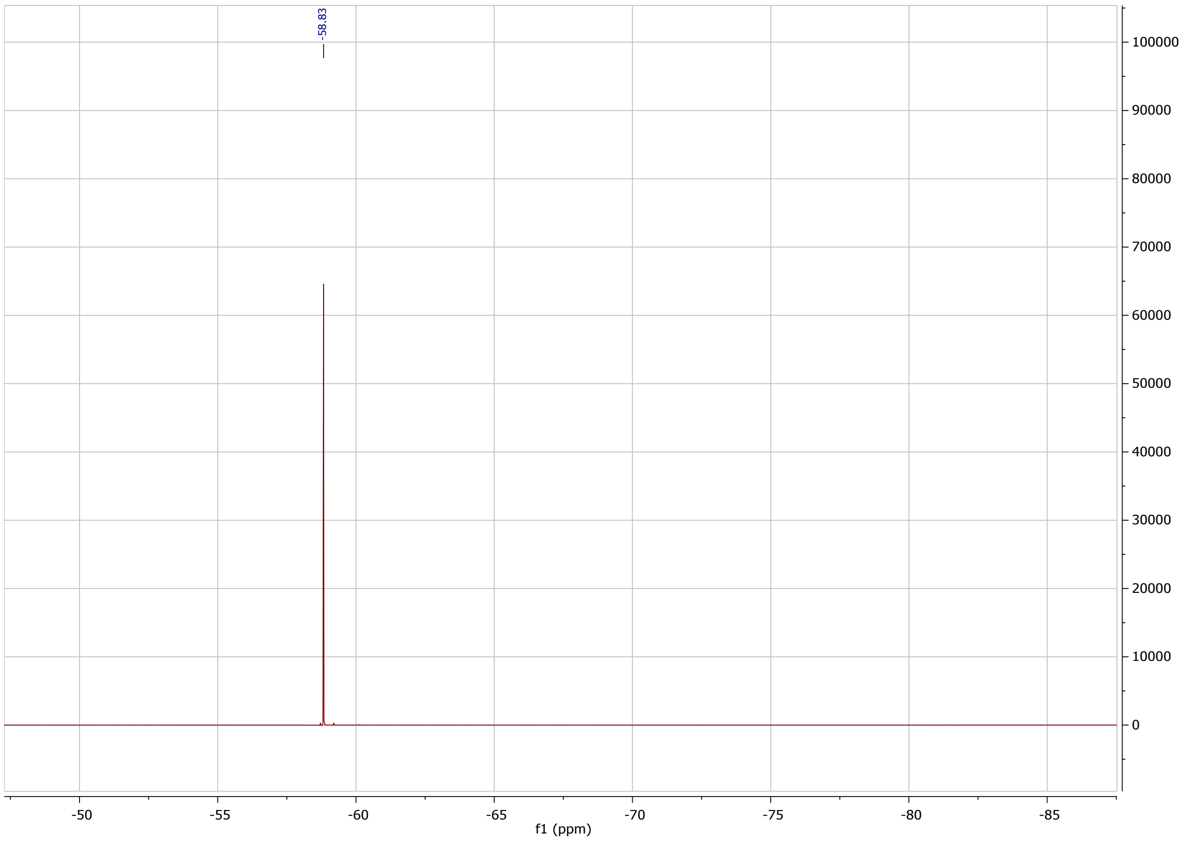
-58.83
100000
90000
80000
70000
60000
50000
40000
30000
20000
10000
0
-50
-55
-60
-65
-70
-75
-80
-85
f1 (ppm)

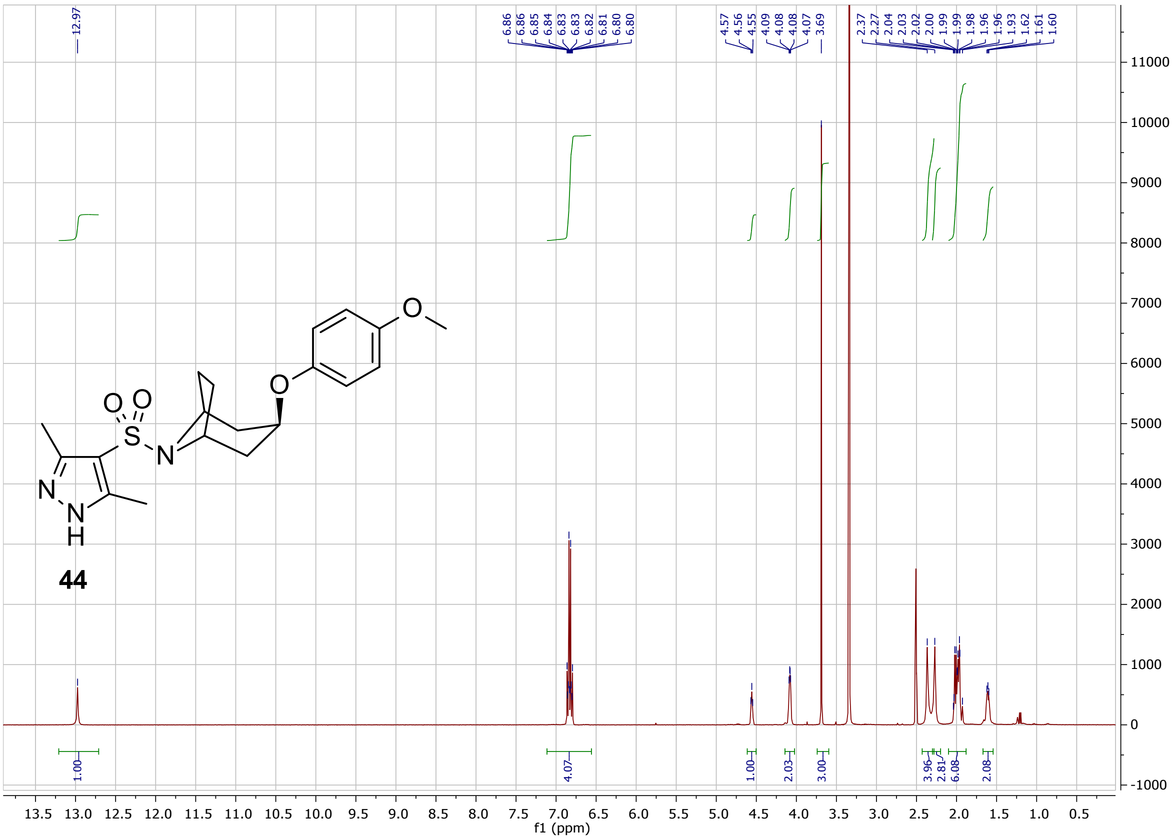
12.97
6.86 6.86 6.85 6.84 6.83 6.83 6.82 6.81 6.80 6.80
4.57 4.56 4.55 4.09 4.08 4.08 4.07 3.69
2.37 2.27 2.04 2.03 2.02 2.00 1.99 1.99 1.98 1.96 1.93 1.62 1.61 1.60
1.00
4.07
1.00
2.03
3.00
3.96
2.81
6.08
2.08
44
f1 (ppm)

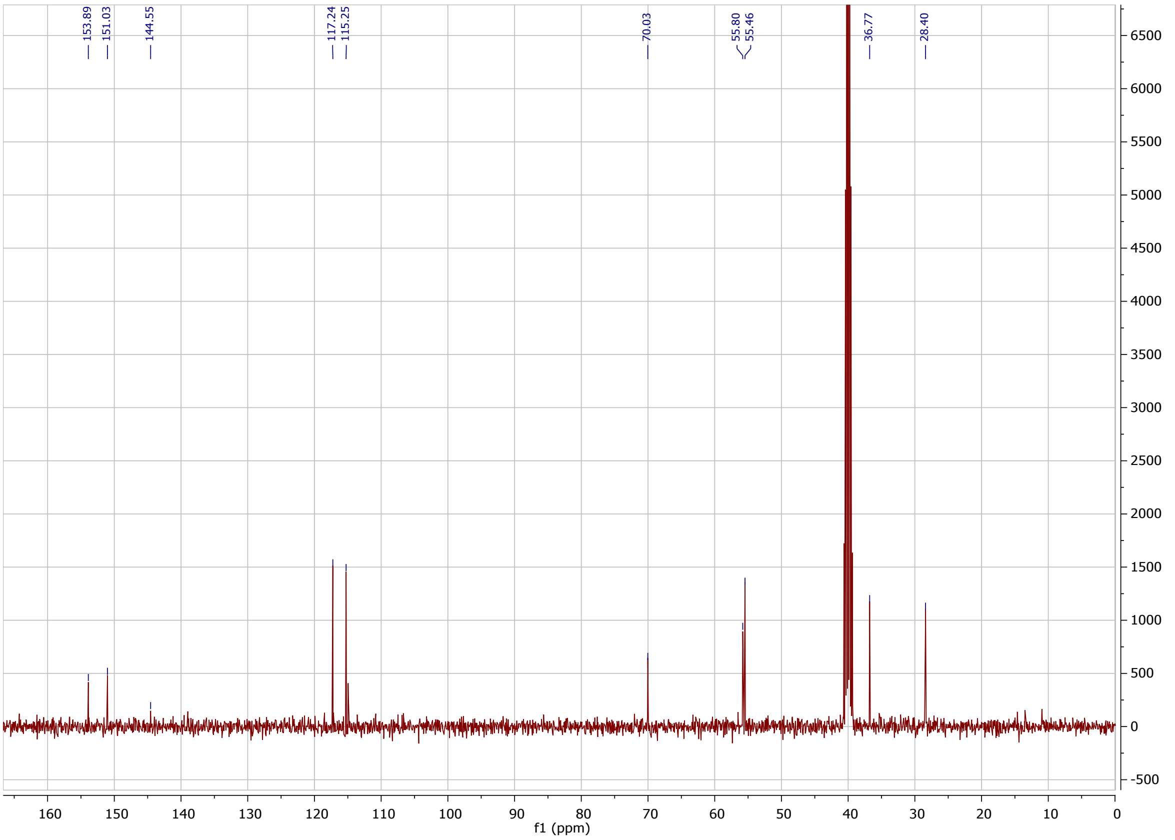
153.89
151.03
144.55
117.24
115.25
70.03
55.80
55.46
36.77
28.40
f1 (ppm)

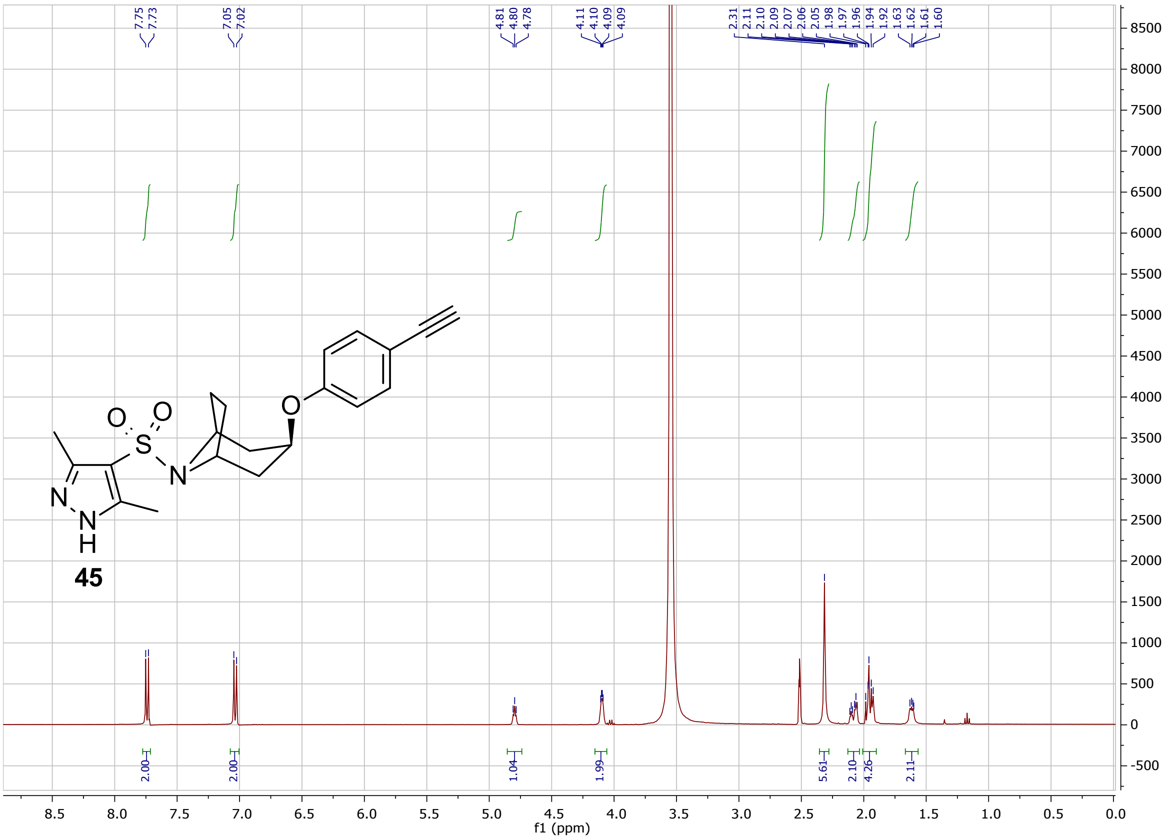

7.75
7.73
7.05
7.02
4.81
4.80
4.78
4.11
4.10
4.09
4.09
2.31
2.11
2.10
2.09
2.07
2.06
2.05
1.98
1.97
1.96
1.94
1.92
1.63
1.62
1.61
1.60
2.00
2.00
1.04
1.99
5.61
2.10
4.26
2.11
45
f1 (ppm)

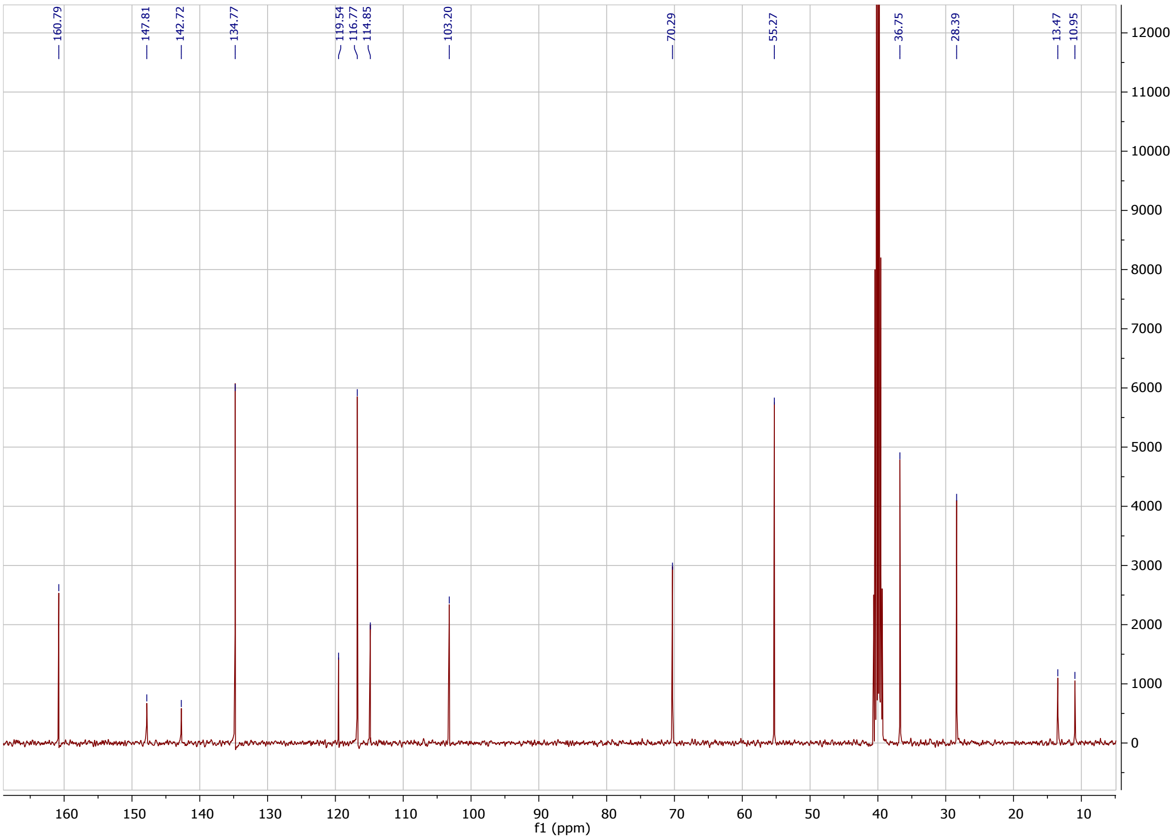

160.79
147.81
142.72
134.77
119.54
116.77
114.85
103.20
70.29
55.27
36.75
28.39
13.47
10.95
f1 (ppm)

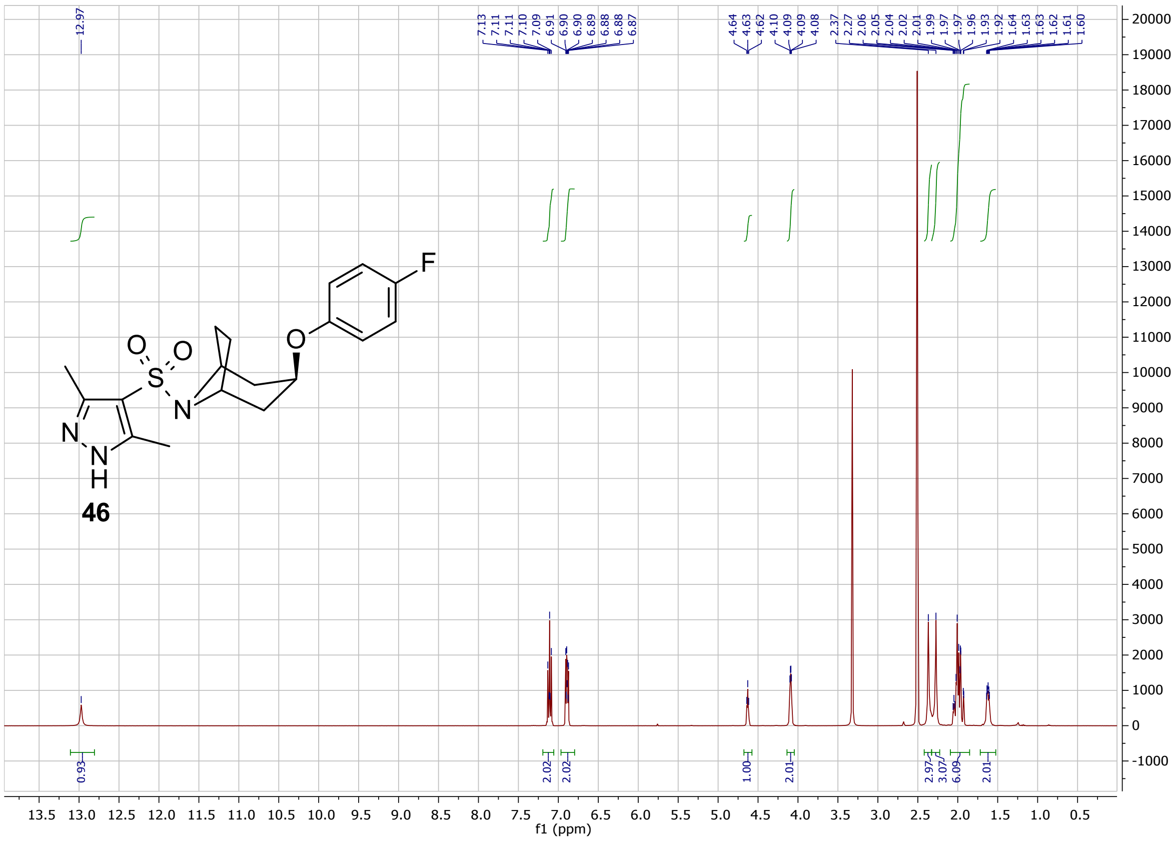

46
f1 (ppm)

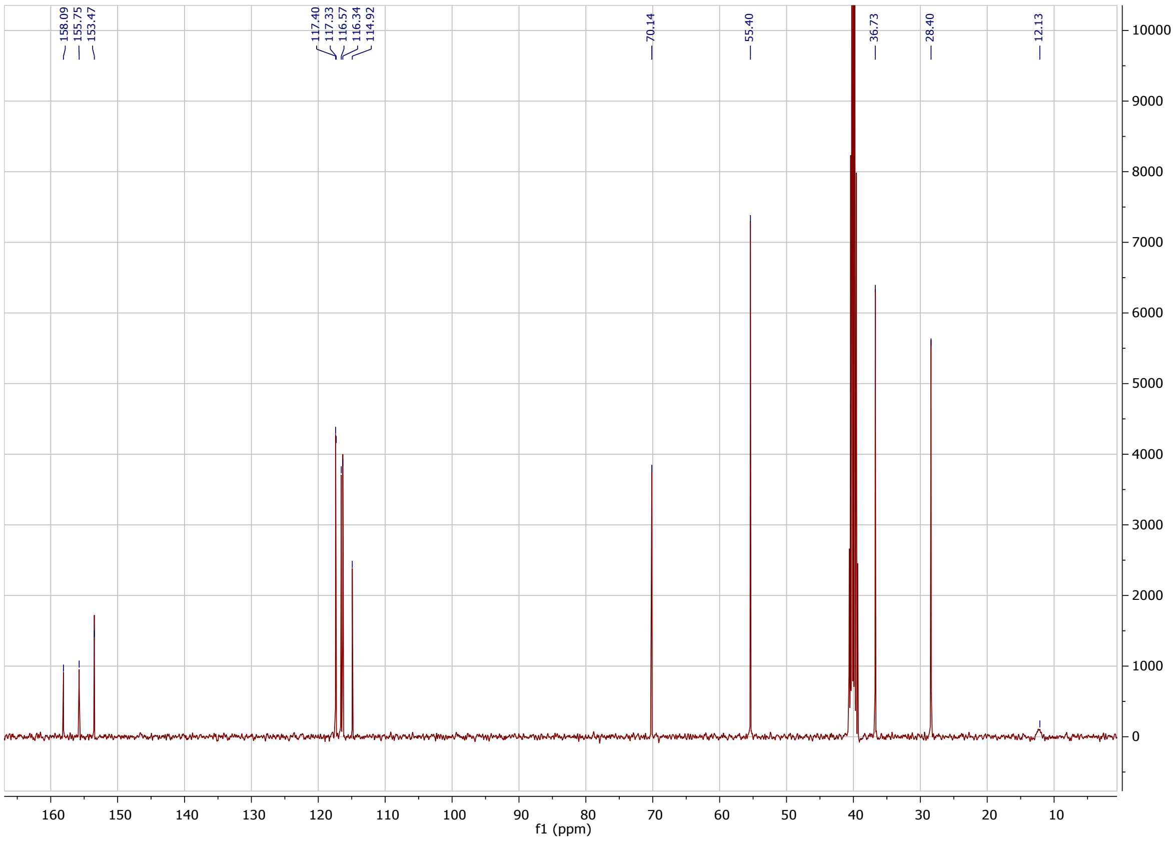

f1 (ppm)

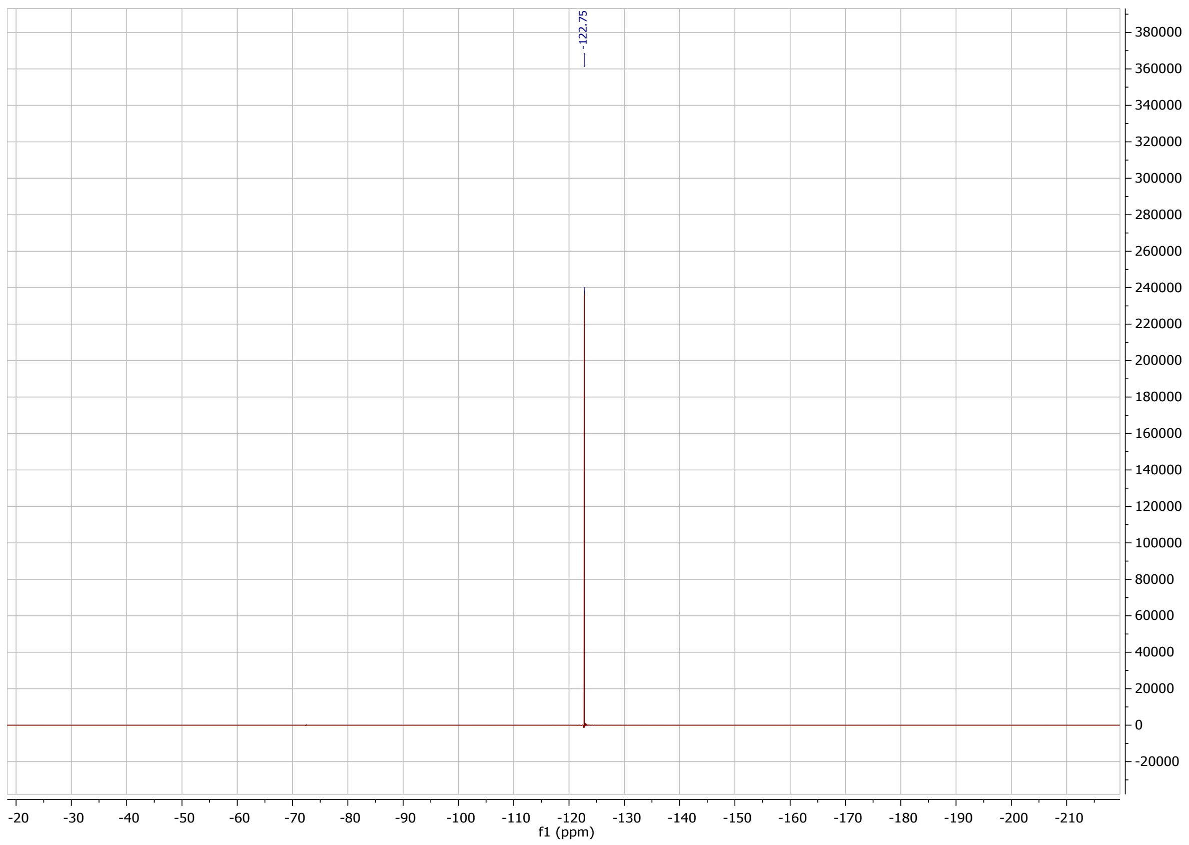

-122.75
380000
360000
340000
320000
300000
280000
260000
240000
220000
200000
180000
160000
140000
120000
100000
80000
60000
40000
20000
0
-20000
-20 -30 -40 -50 -60 -70 -80 -90 -100 -110 -120 -130 -140 -150 -160 -170 -180 -190 -200 -210
f1 (ppm)

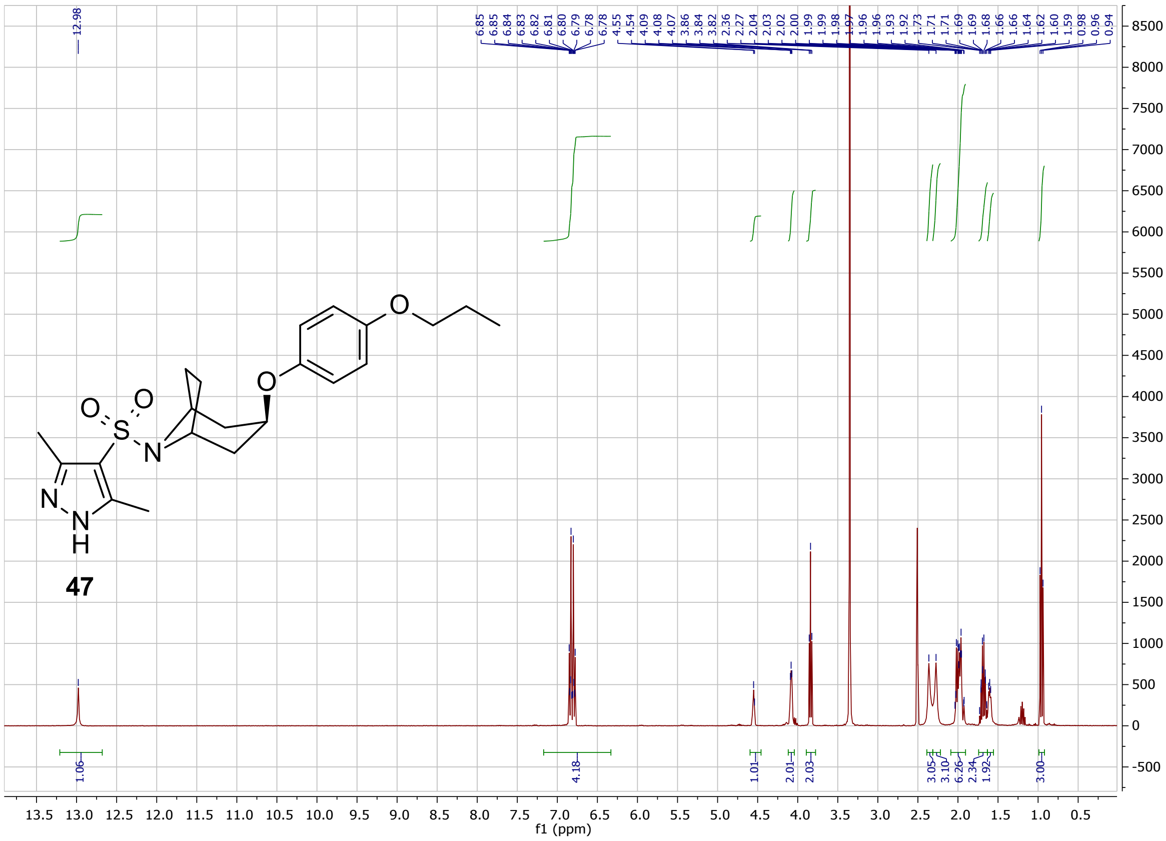
12.98
6.85 6.85 6.84 6.83 6.82 6.81 6.80 6.79 6.78 6.78 4.55 4.54 4.09 4.08 4.07 3.86 3.84 3.82 2.36 2.27 2.04 2.03 2.02 2.00 1.99 1.99 1.98 1.97 1.96 1.96 1.93 1.92 1.73 1.71 1.71 1.69 1.69 1.68 1.66 1.64 1.62 1.60 1.59 0.98 0.96 0.94
1.06
4.18
1.01
2.01
2.03
3.05
3.10
6.26
2.34
1.92
3.00
47
f1 (ppm)

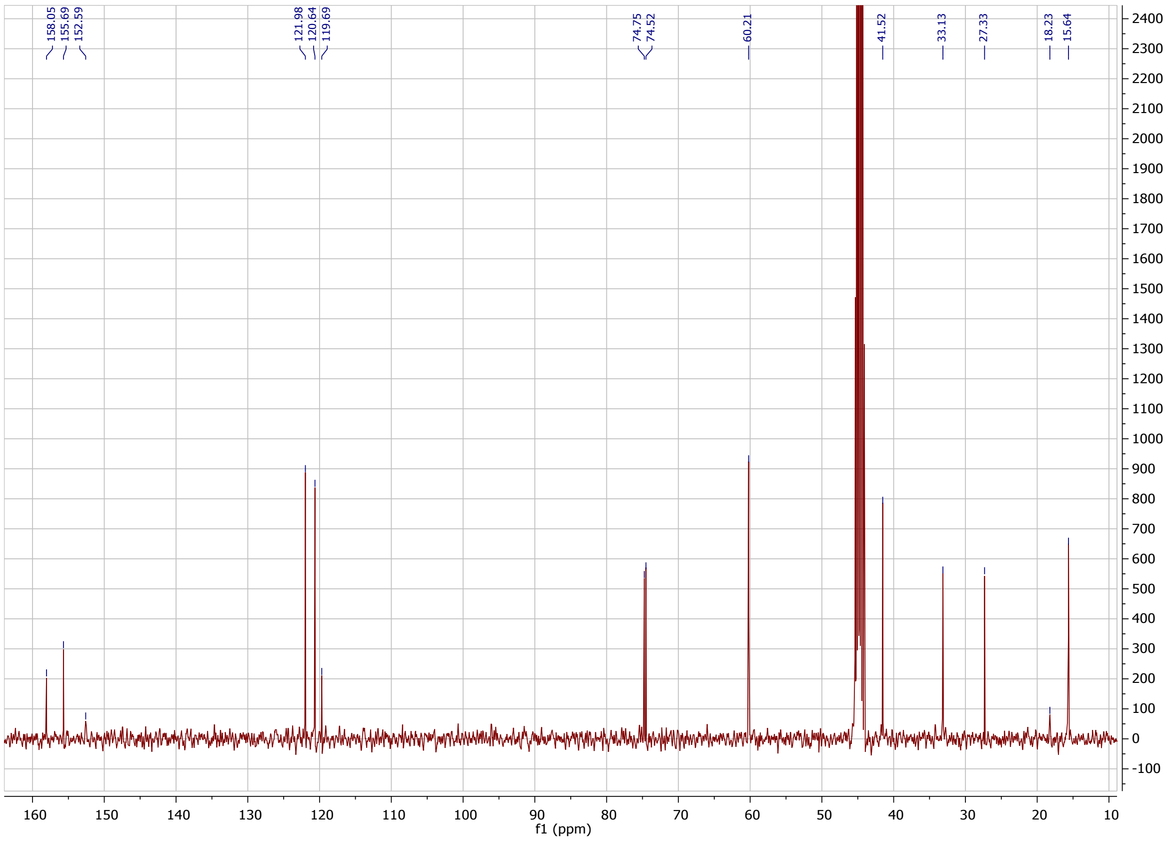
158.05 155.69 152.59
121.98 120.64 119.69
74.75 74.52
60.21
41.52
33.13
27.33
18.23 15.64
f1 (ppm)

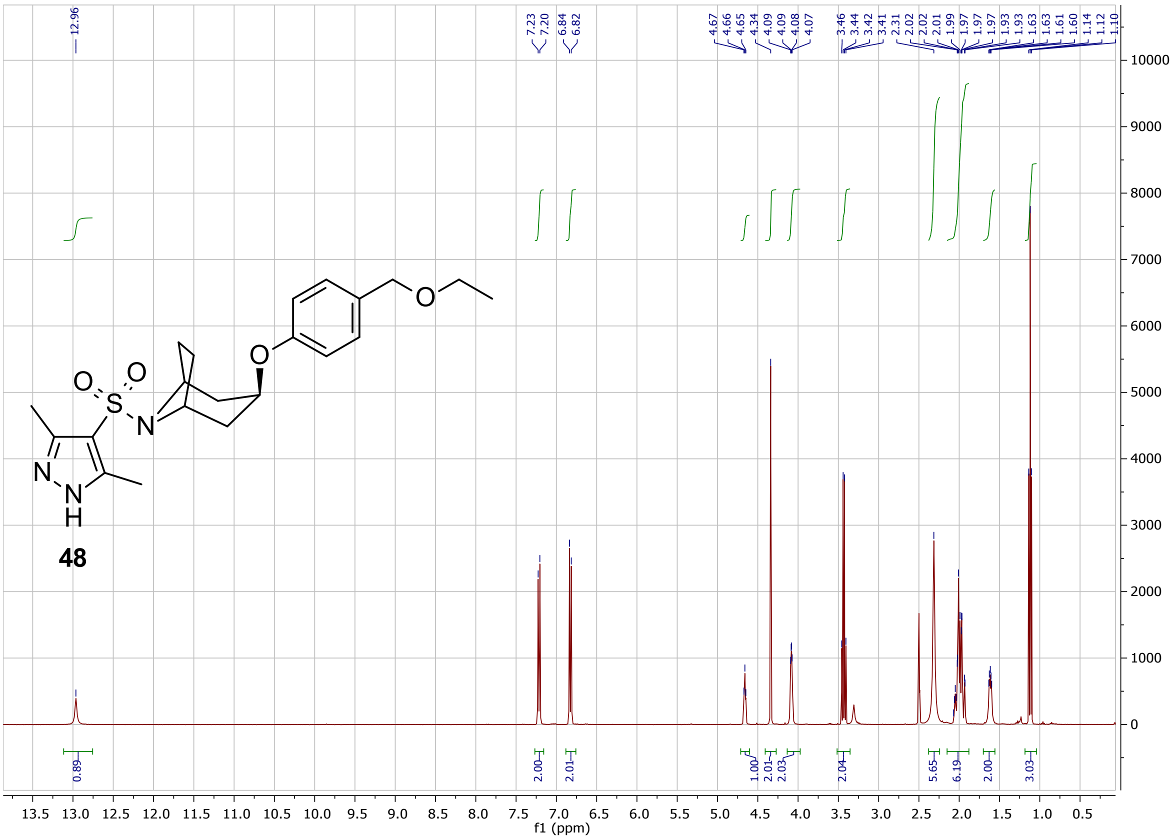
12.96
7.23
7.20
6.84
6.82
4.67
4.66
4.65
4.34
4.09
4.09
4.08
4.07
3.46
3.44
3.42
3.41
2.31
2.02
2.02
2.01
1.99
1.97
1.97
1.97
1.93
1.93
1.63
1.63
1.61
1.60
1.14
1.12
1.10
48
0.89
2.00
2.01
1.00
2.01
2.03
2.04
5.65
6.19
2.00
3.03
f1 (ppm)

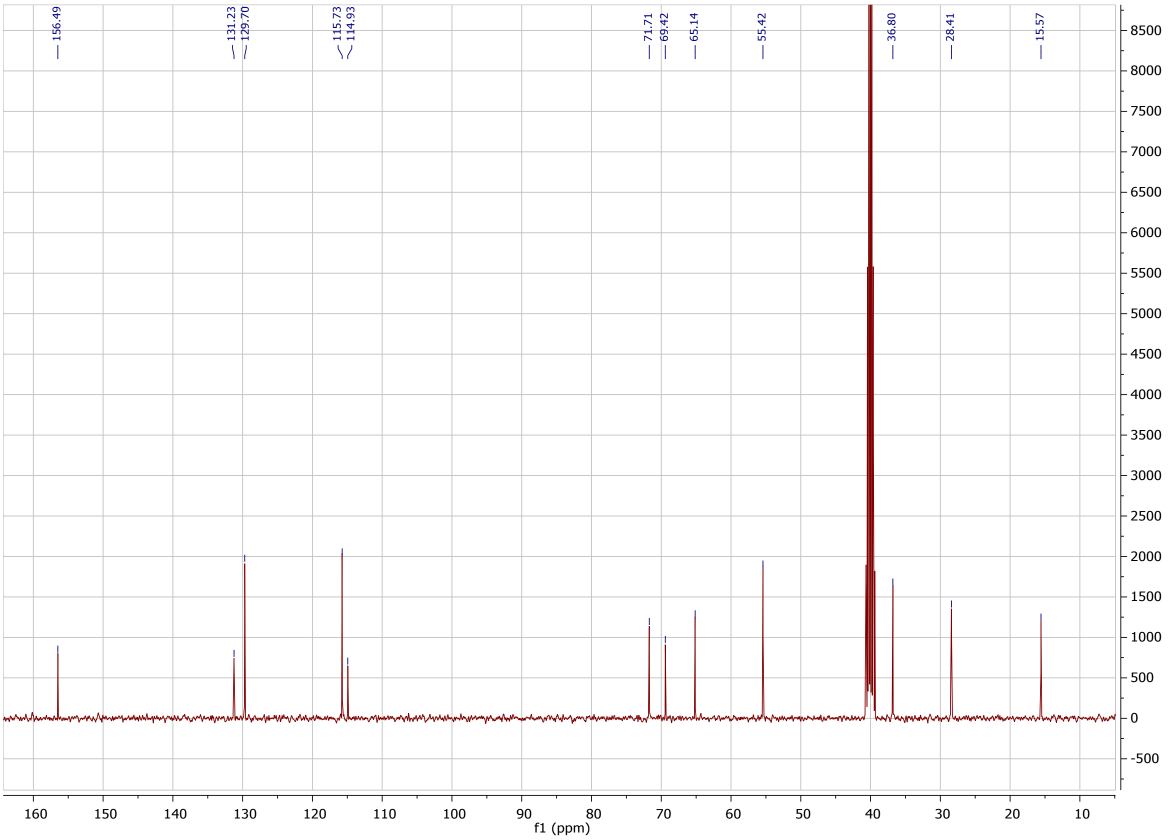
156.49
131.23
129.70
115.73
114.93
71.71
69.42
65.14
55.42
36.80
28.41
15.57
f1 (ppm)

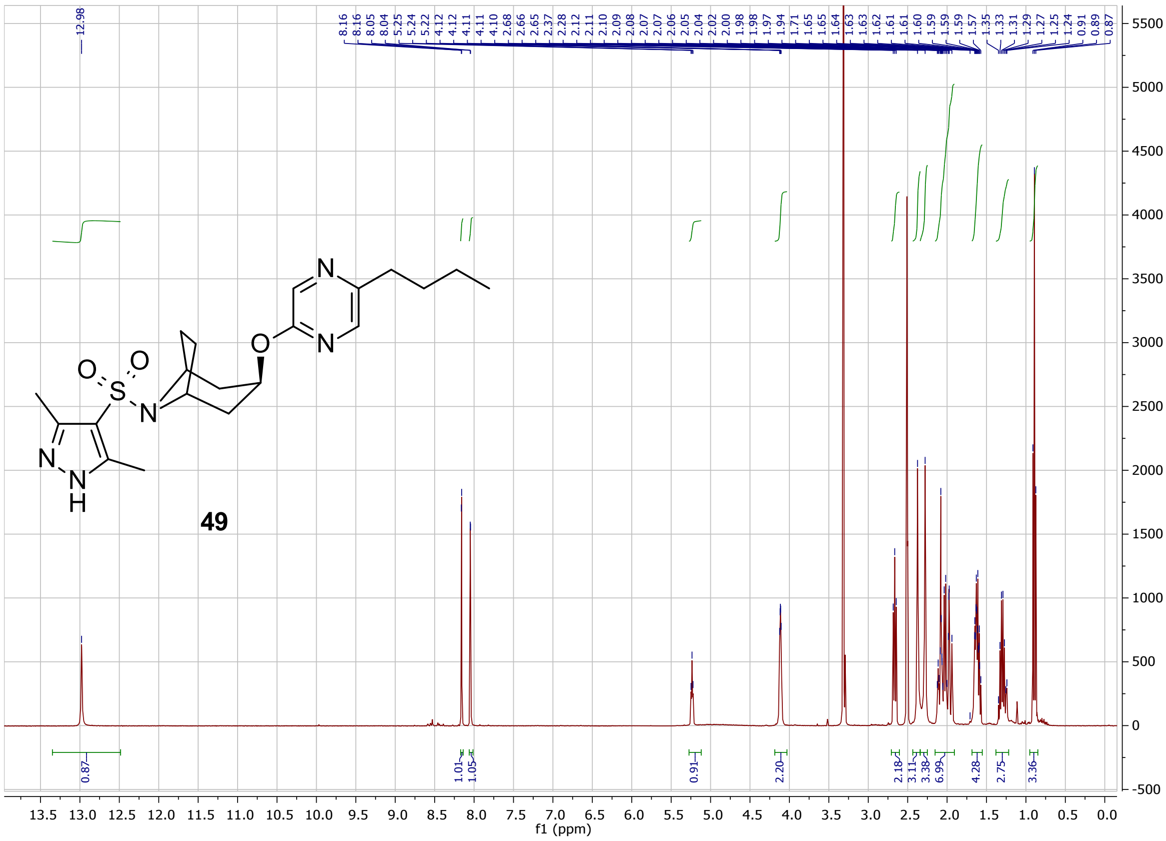
12.98
8.16 8.16 8.05 8.04 5.25 5.24 5.22 4.12 4.12 4.11 4.11 4.10 2.68 2.66 2.65 2.37 2.28 2.12 2.11 2.10 2.09 2.08 2.07 2.07 2.06 2.05 2.04 2.02 2.00 1.98 1.98 1.97 1.94 1.71 1.65 1.65 1.64 1.63 1.63 1.62 1.61 1.61 1.60 1.59 1.59 1.59 1.57 1.35 1.33 1.31 1.29 1.27 1.25 1.24 0.91 0.89 0.87
0.87 1.01 1.05 0.91 2.20 2.18 3.11 3.38 6.99 4.28 2.75 3.36
49
f1 (ppm)

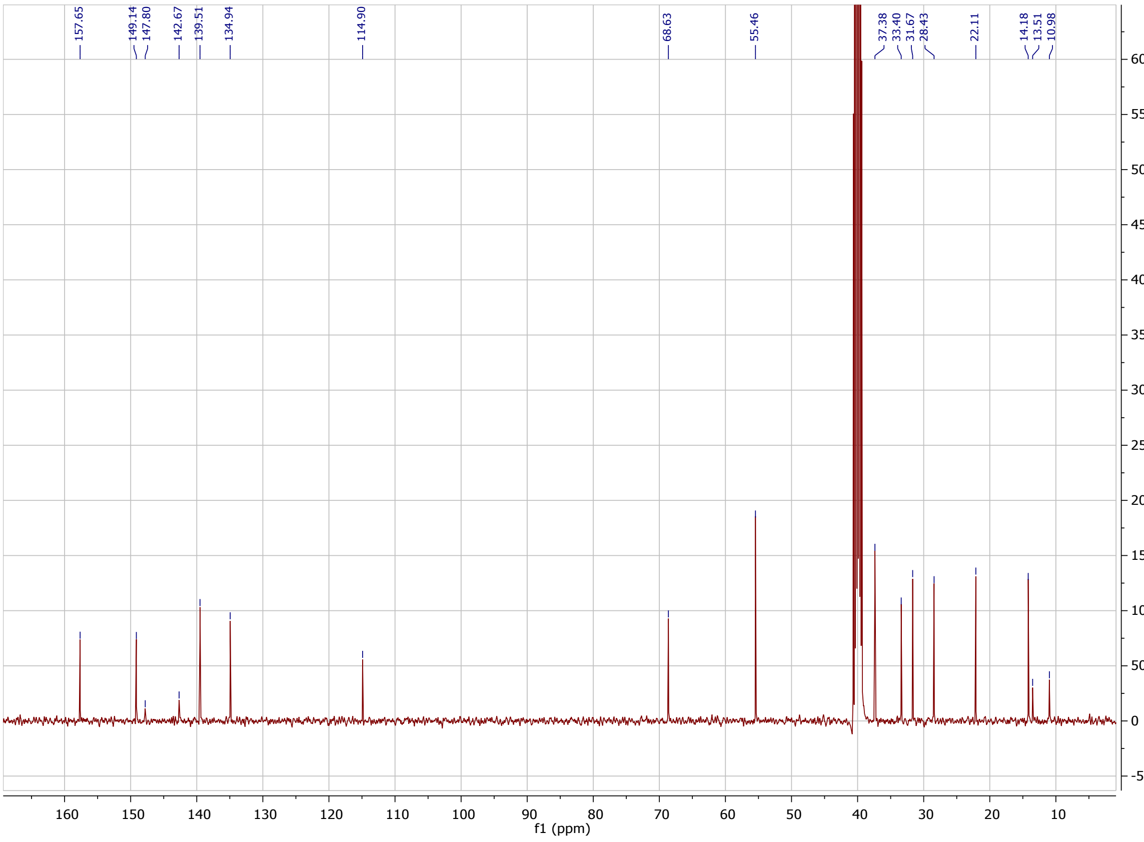
157.65 149.14 147.80 142.67 139.51 134.94 114.90 68.63 55.46 37.38 33.40 31.67 28.43 22.11 14.18 13.51 10.98
f1 (ppm)

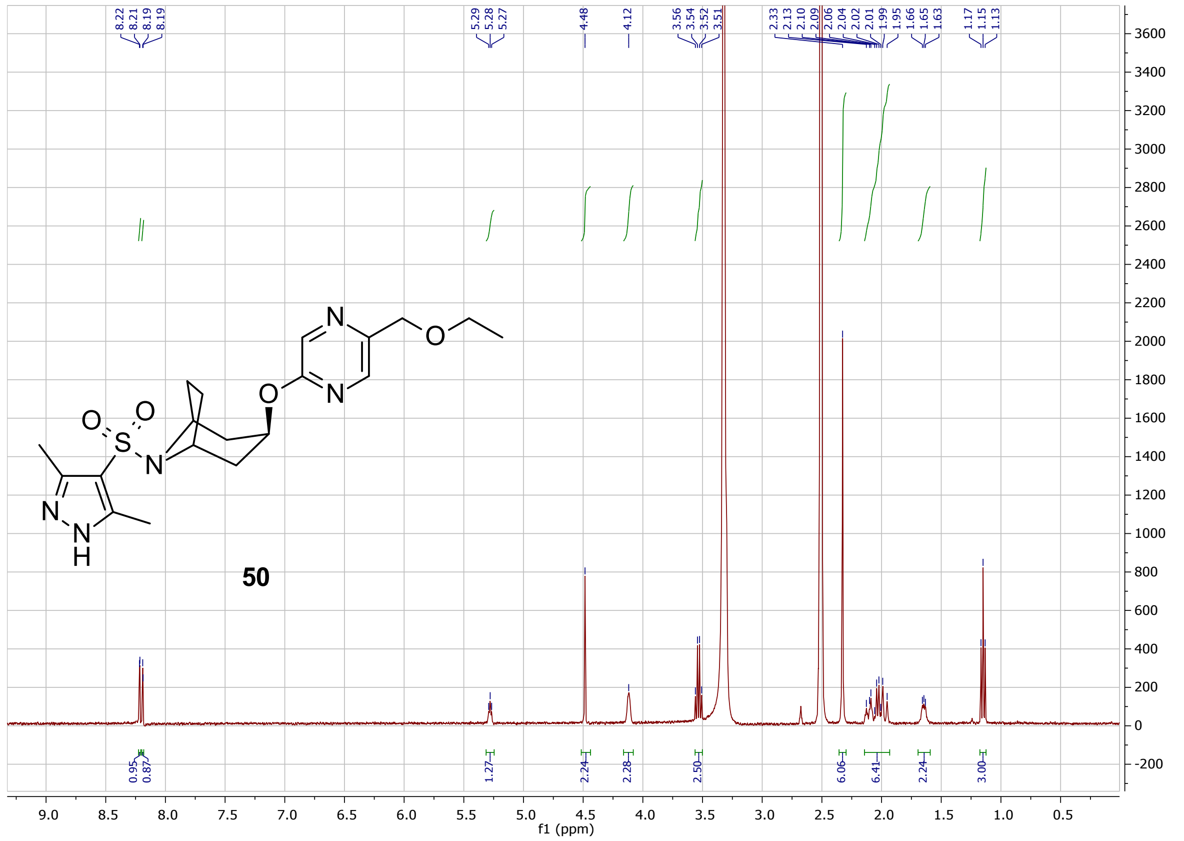
8.22
8.21
8.19
8.19
5.29
5.28
5.27
4.48
4.12
3.56
3.54
3.52
3.51
2.33
2.13
2.10
2.09
2.06
2.04
2.02
2.01
1.99
1.95
1.66
1.65
1.63
1.17
1.15
1.13
50
0.95
0.87
1.27
2.24
2.28
2.50
6.06
6.41
2.24
3.00
f1 (ppm)

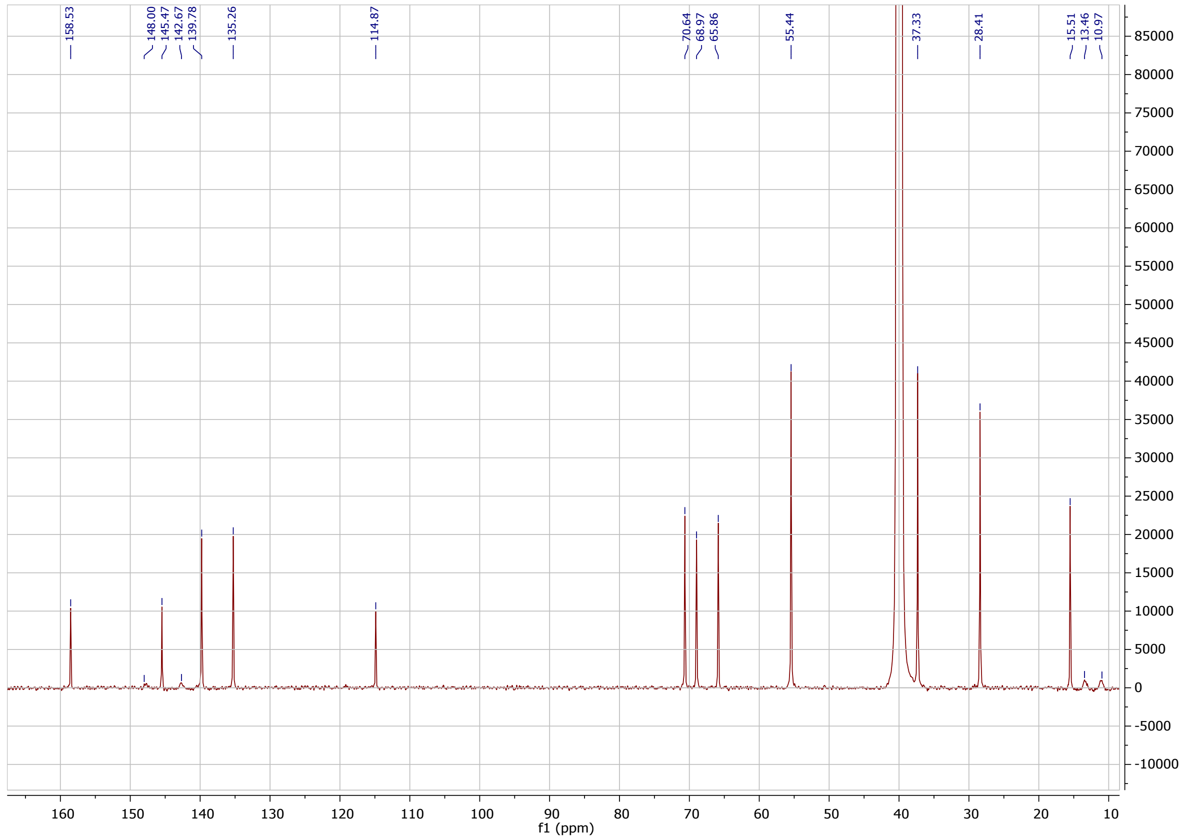
158.53
148.00
145.47
142.67
139.78
135.26
114.87
70.64
68.97
65.86
55.44
37.33
28.41
15.51
13.46
10.97
f1 (ppm)

2. LC-MS analyses of final compounds 1-50

10mM Stock solution of test compound (**1-50**) was prepared in DMSO-d_6 and further diluted 20-fold with CH_3CN-H_2O (1:1) for analysis. The QC analyses were performed on a Waters ACQUITY UPLC-MS system consisting of a single quadrupole detector (SQD) mass spectrometer equipped with an electrospray ionization interface and a photodiode array detector (PDA) from Waters Inc. (Milford, MA, USA). Electrospray ionization in positive and negative mode was applied in the mass scan range 100-500 Da. The PDA range was 210-400 nm. The analyses were run on an ACQUITY UPLC BEH C_{18} column (100 x 2.1 mm ID, particle size 1.7 μm) with a VanGuard BEH C_{18} pre-column (5 x 2.1 mm ID, particle size 1.7 μm). The mobile phase was 10 mM NH_4OAc in H_2O at pH 5 adjusted with AcOH (A) and 10mM NH_4OAc in CH_3CN-H_2O (95:5) at pH 5 (B) with 0.5 mL/min as flow rate. A linear gradient was applied: 0-0.2 min: 10%B, 0.2-6.2 min: 10-90%B, 6.2-6.3 min: 90-100%, 6.3-7.0 min: 100%B. Purity of the final compounds (**1-50**) was equal or greater than 95% by UV at 215 nm for all of the compounds, except for analogue **3** (91% purity).

Compound 1

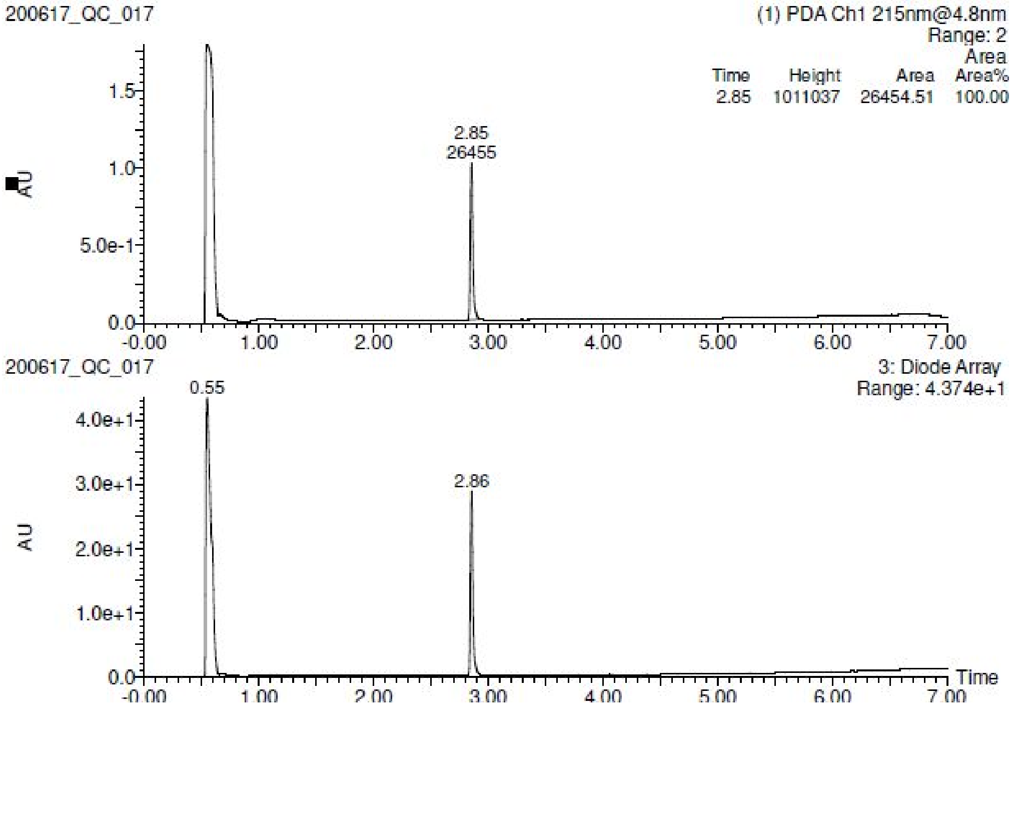

Compound 2

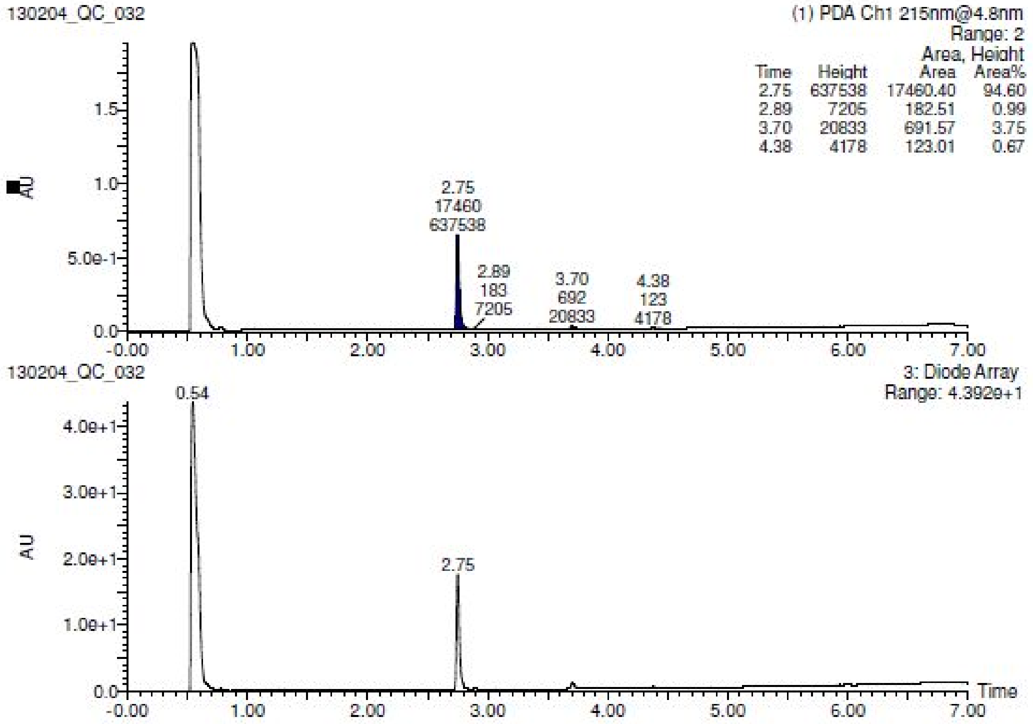

Compound 3

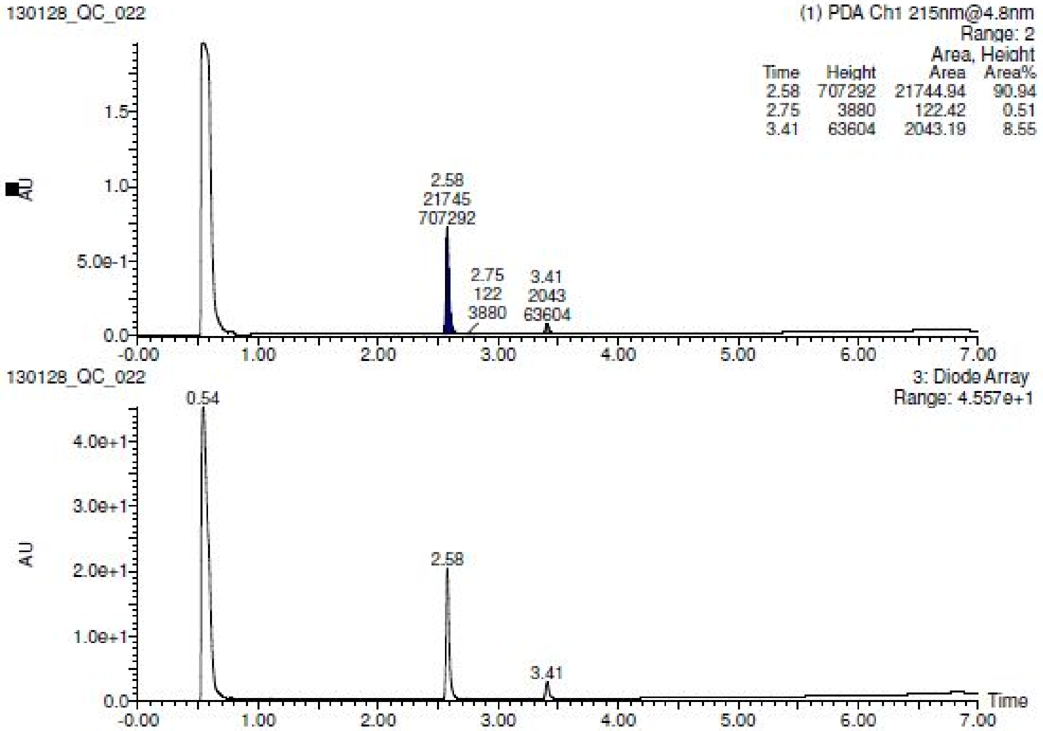

Compound 4

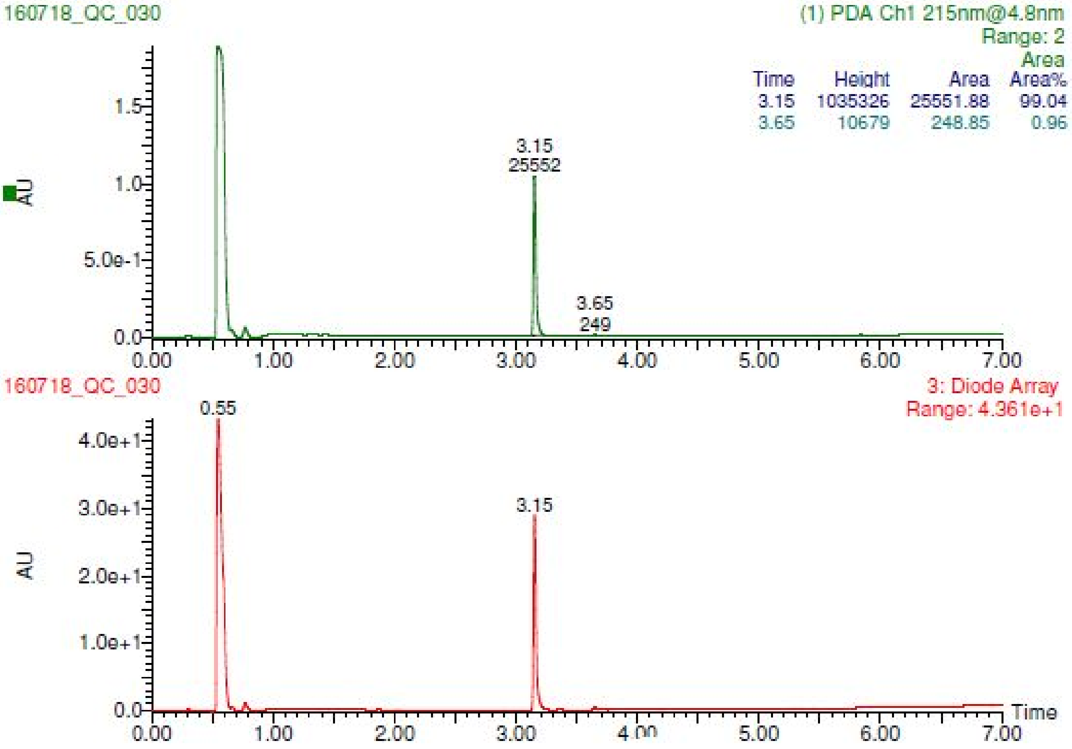

Compound 5

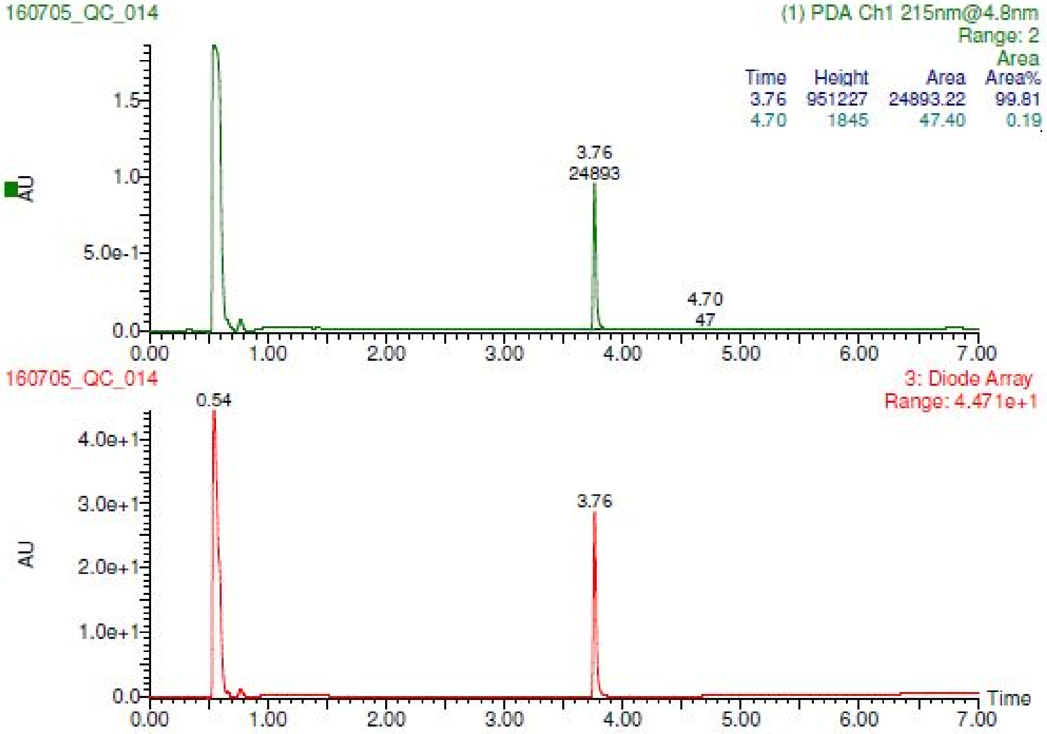

Compound 6

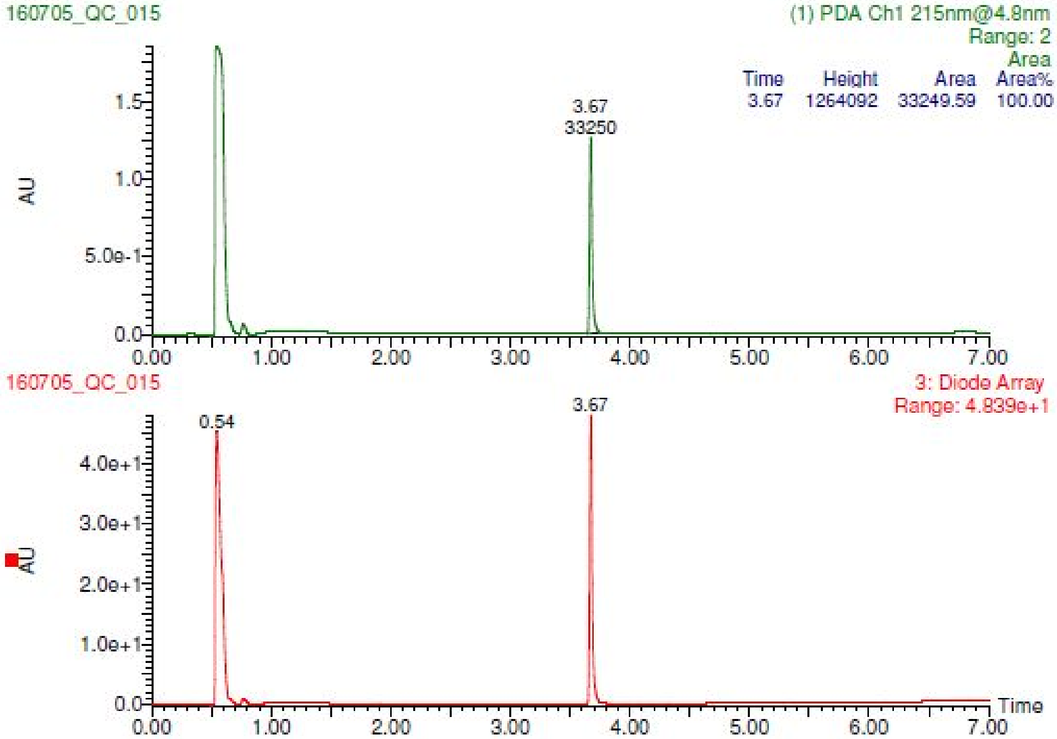

Compound 7

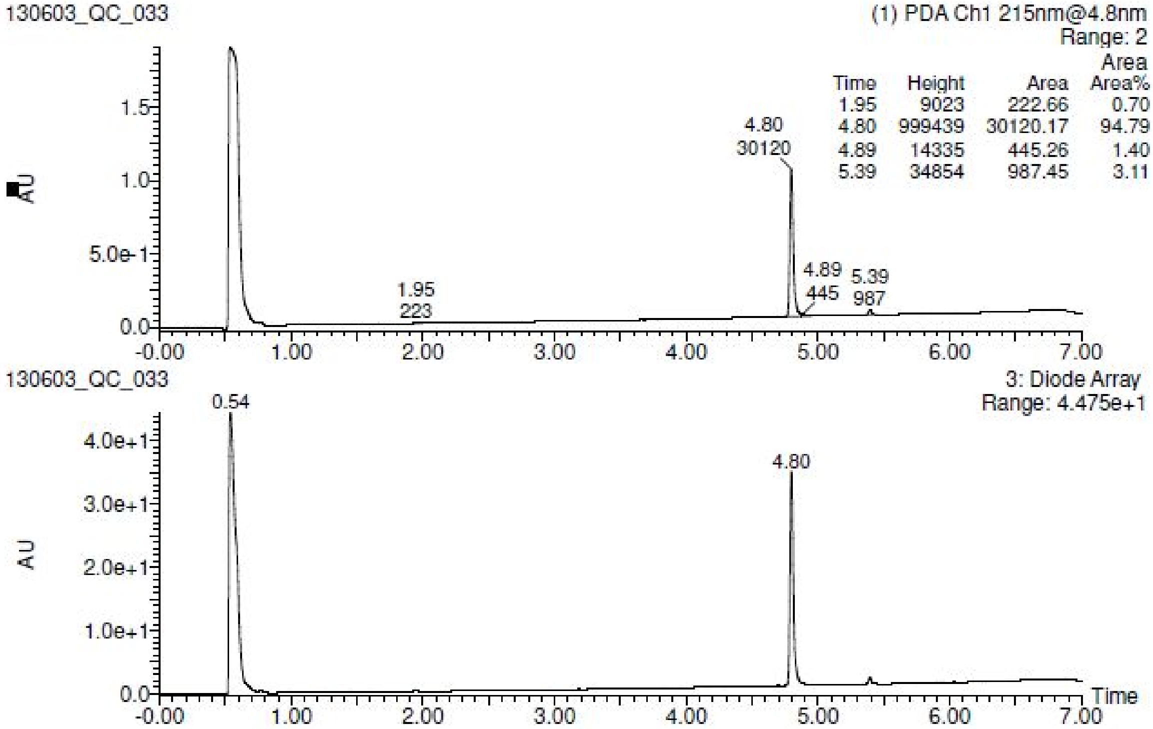

Compound 8

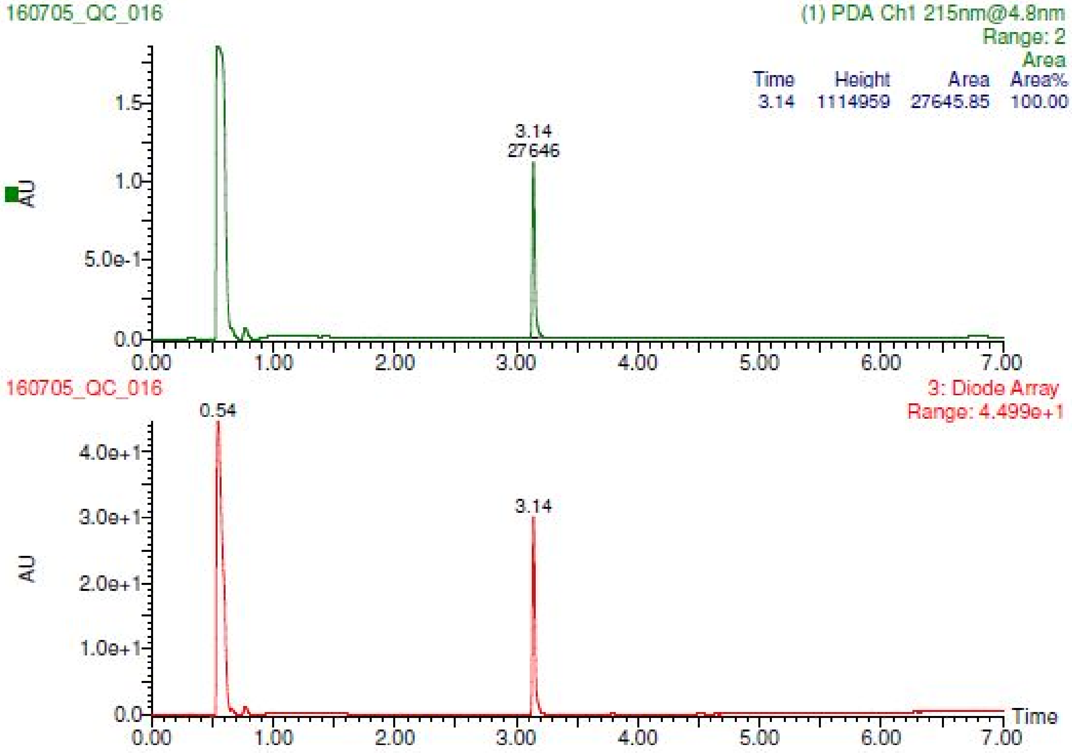

Compound 9

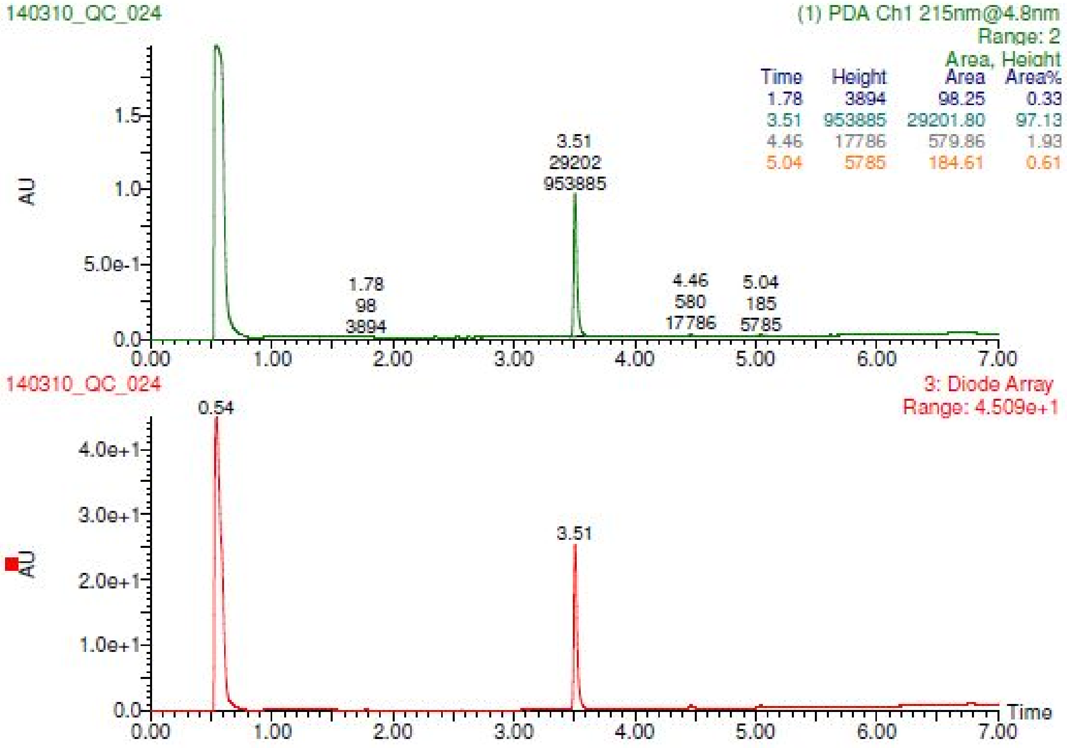

Compound 10

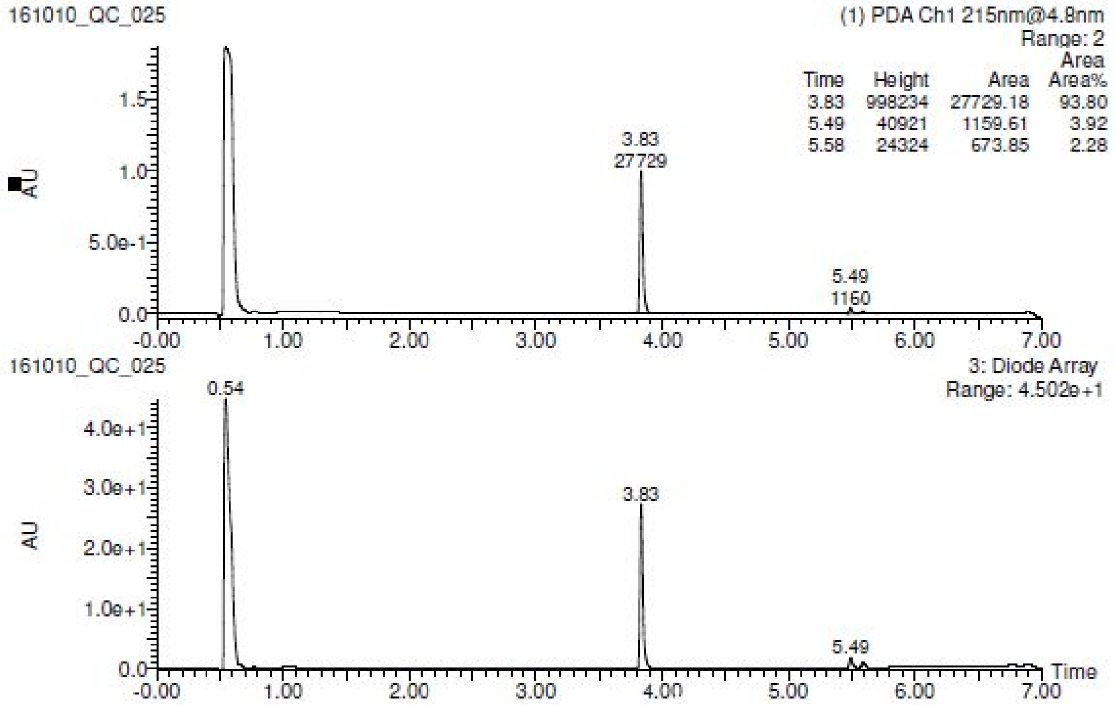

Compound 11

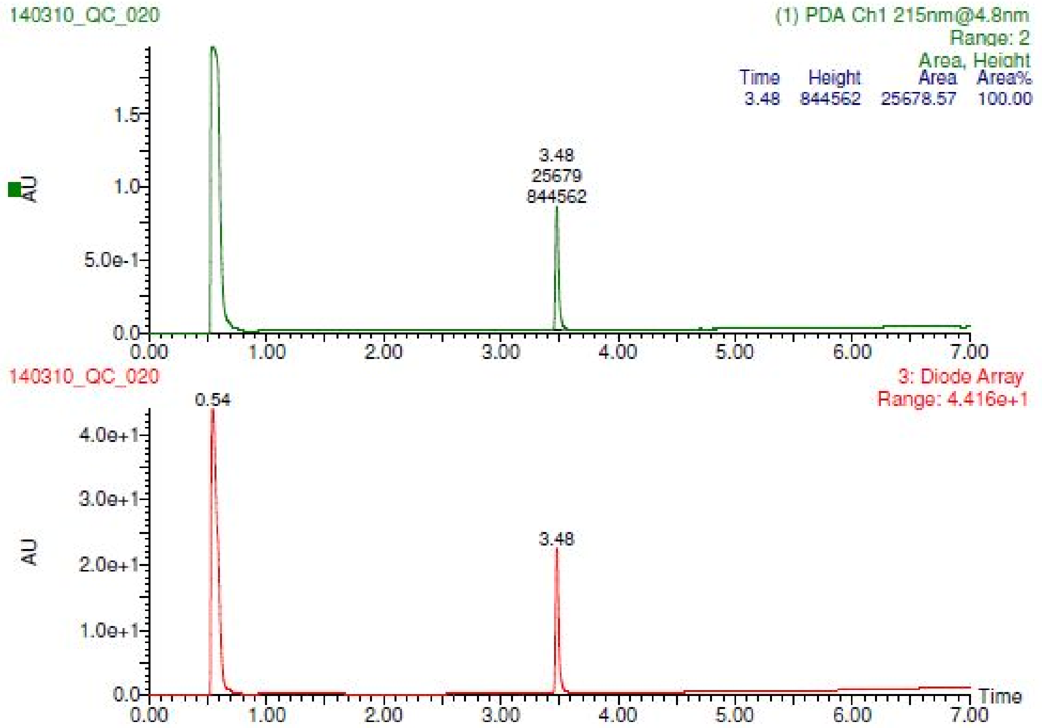

Compound 12

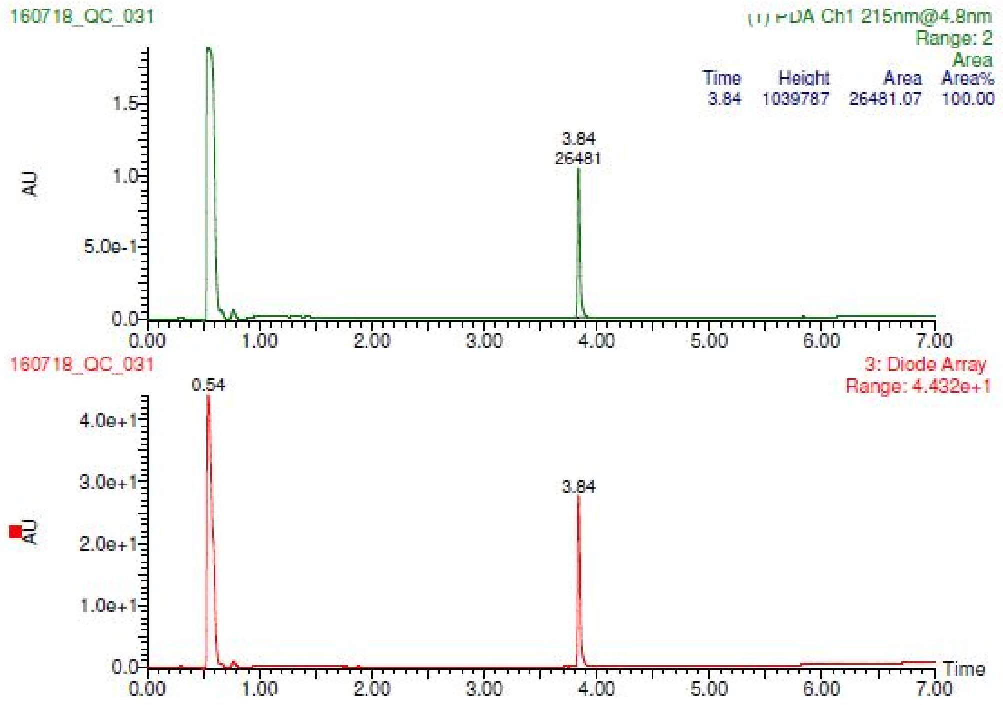

Compound 13

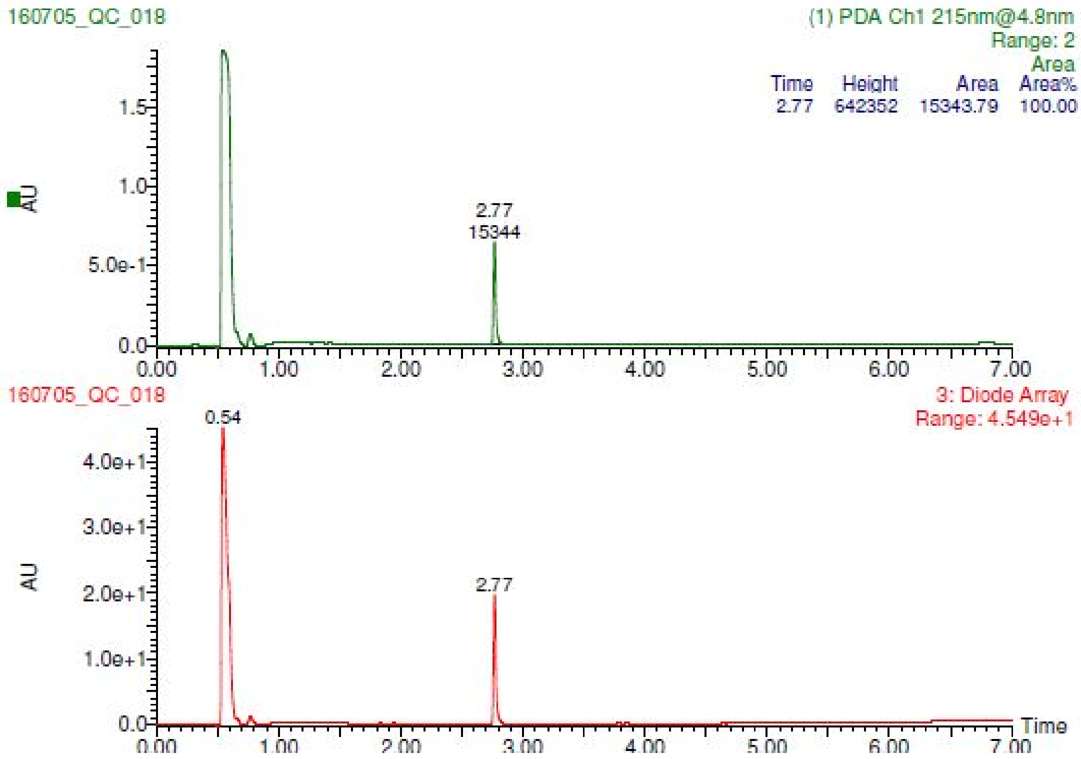

Compound 14

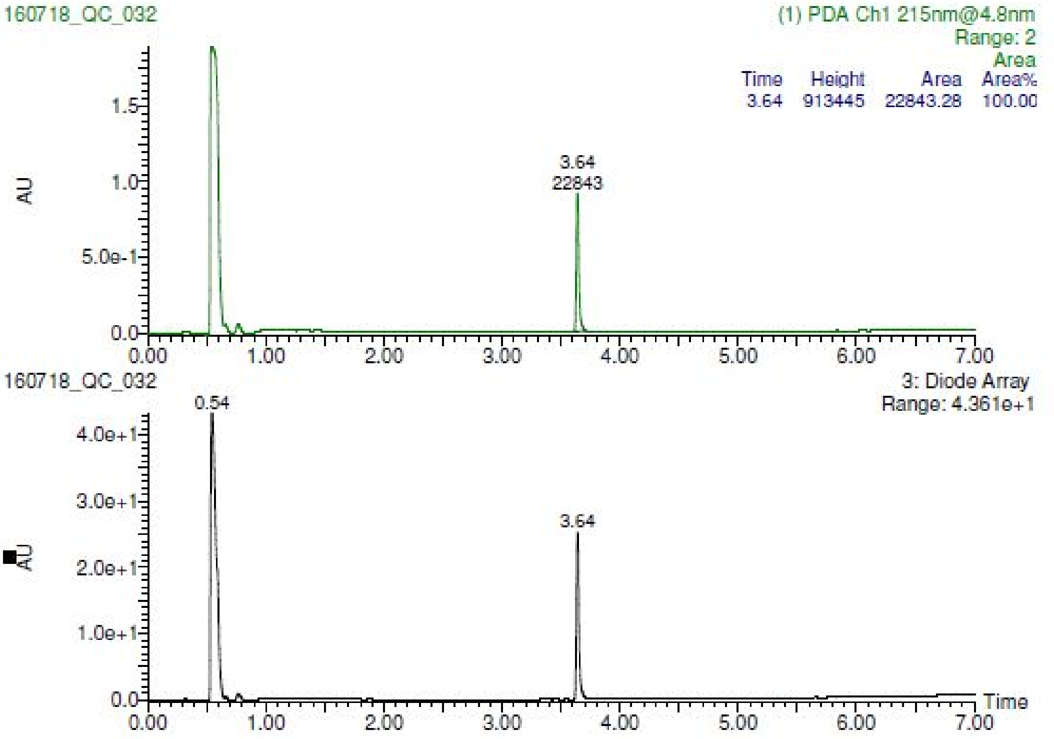

Compound 15

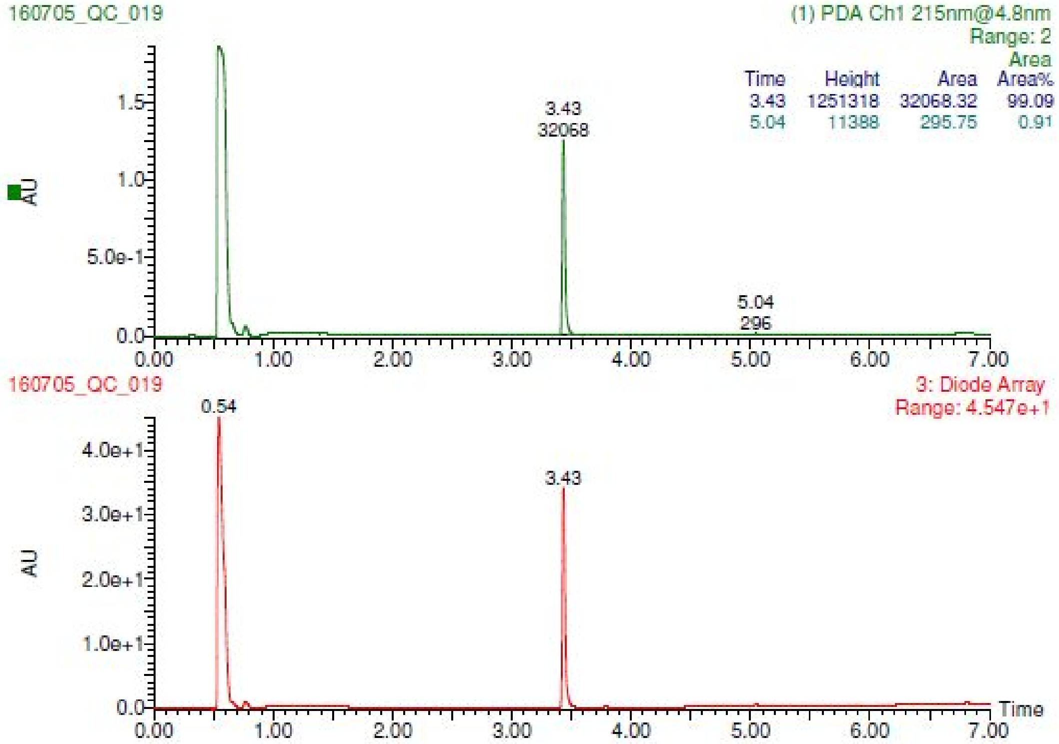

Compound 16

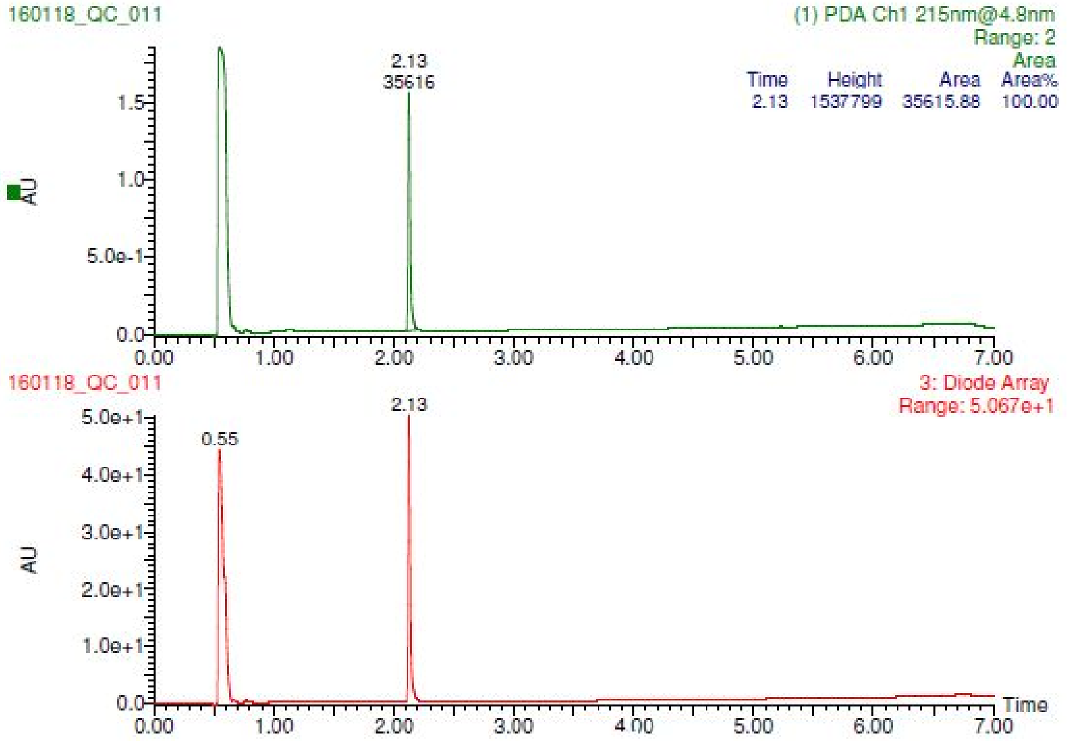

Compound 17

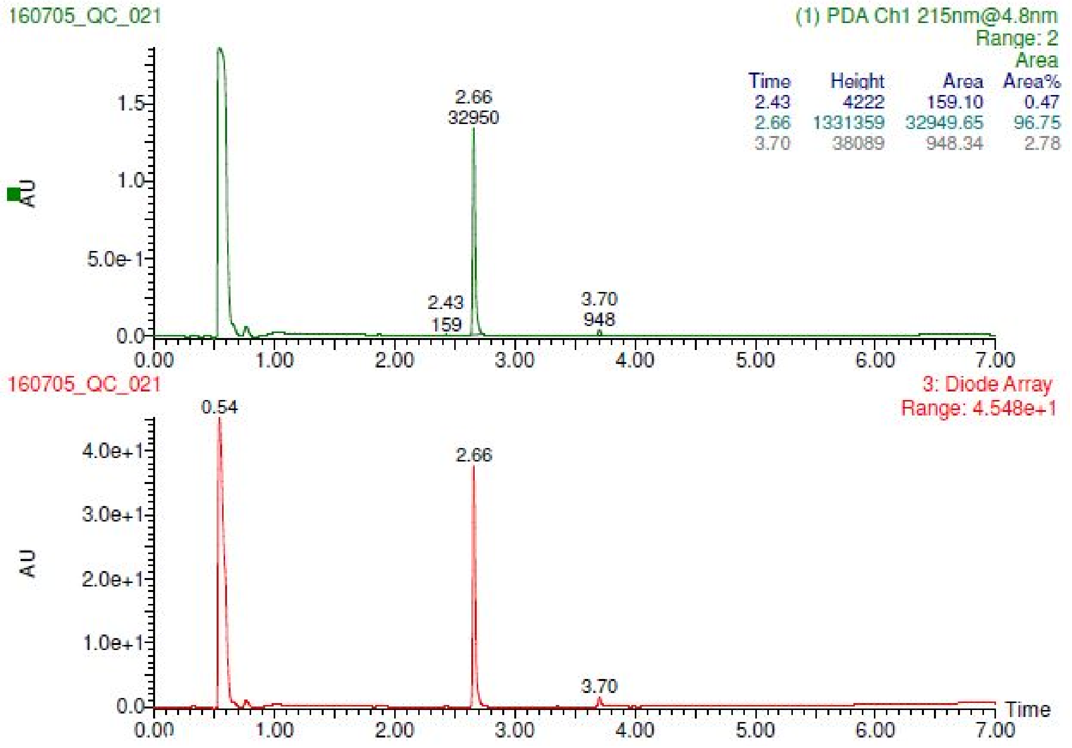

Compound 18

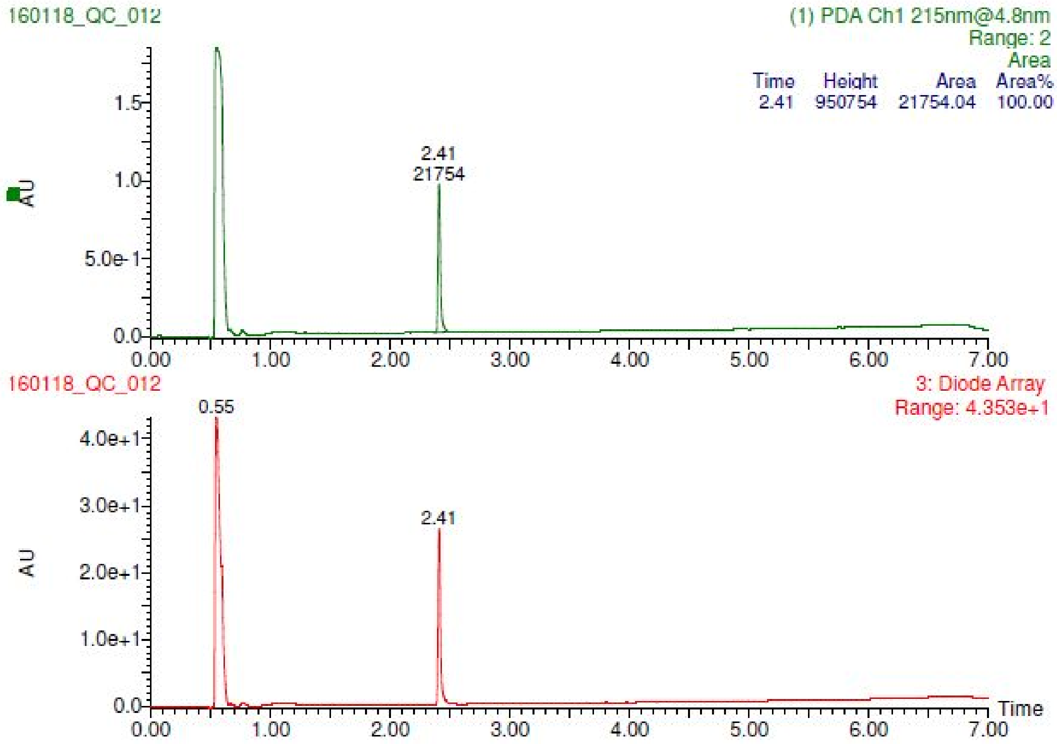

Compound 19

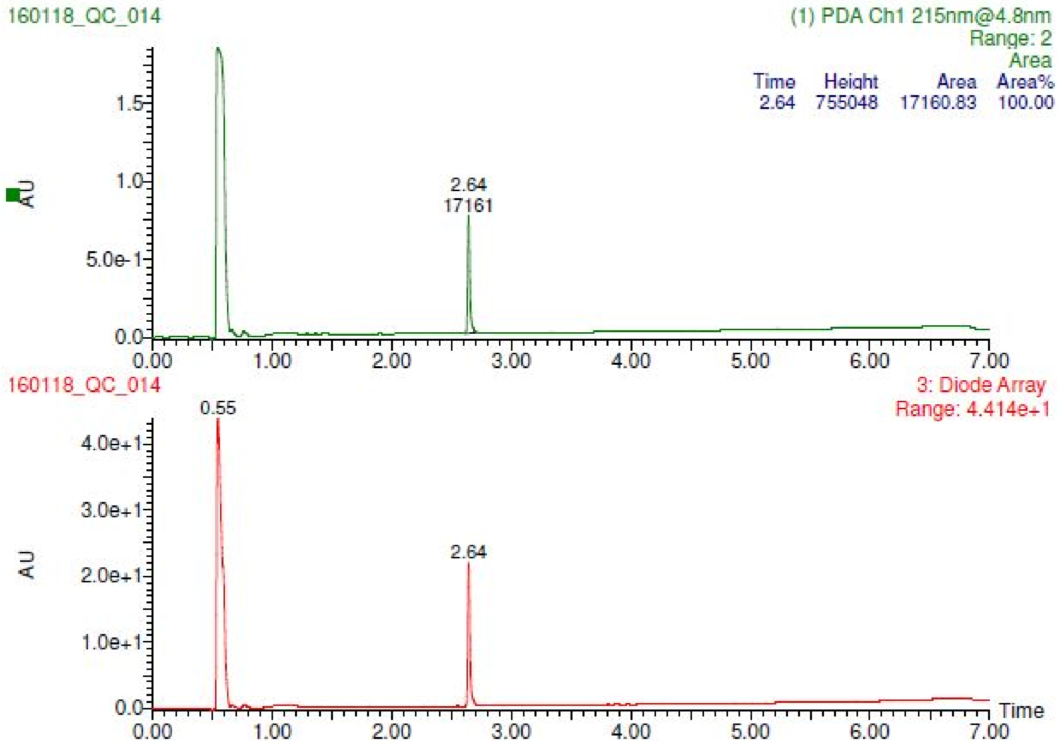

Compound 20

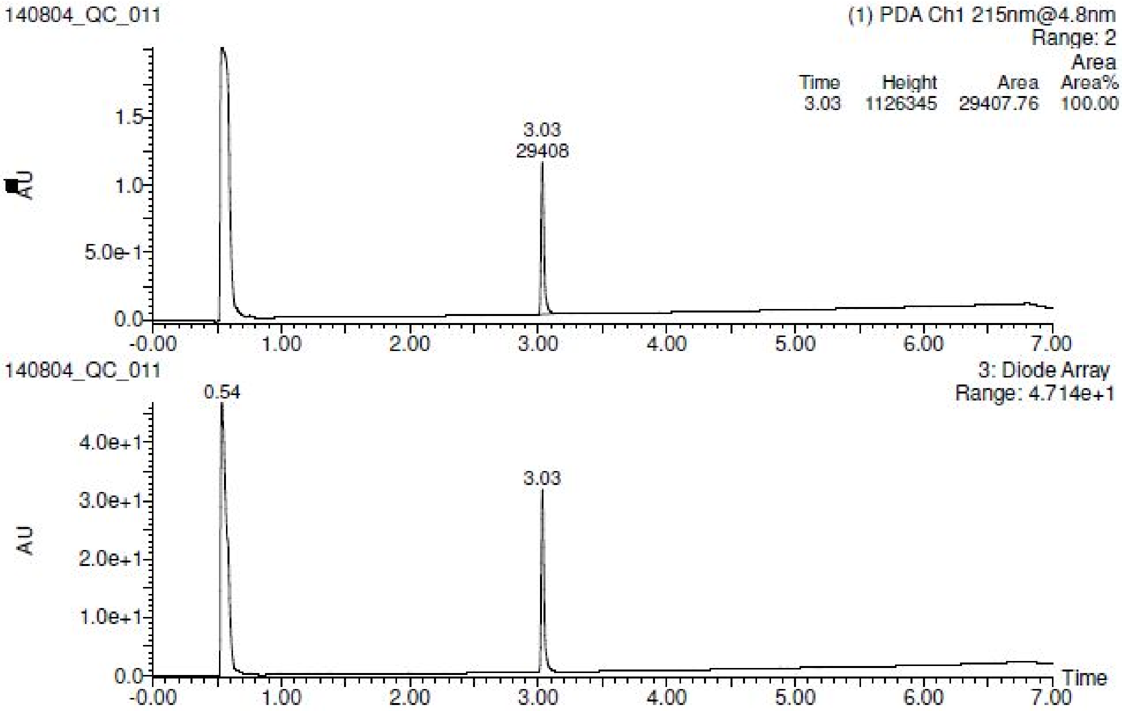

Compound 21

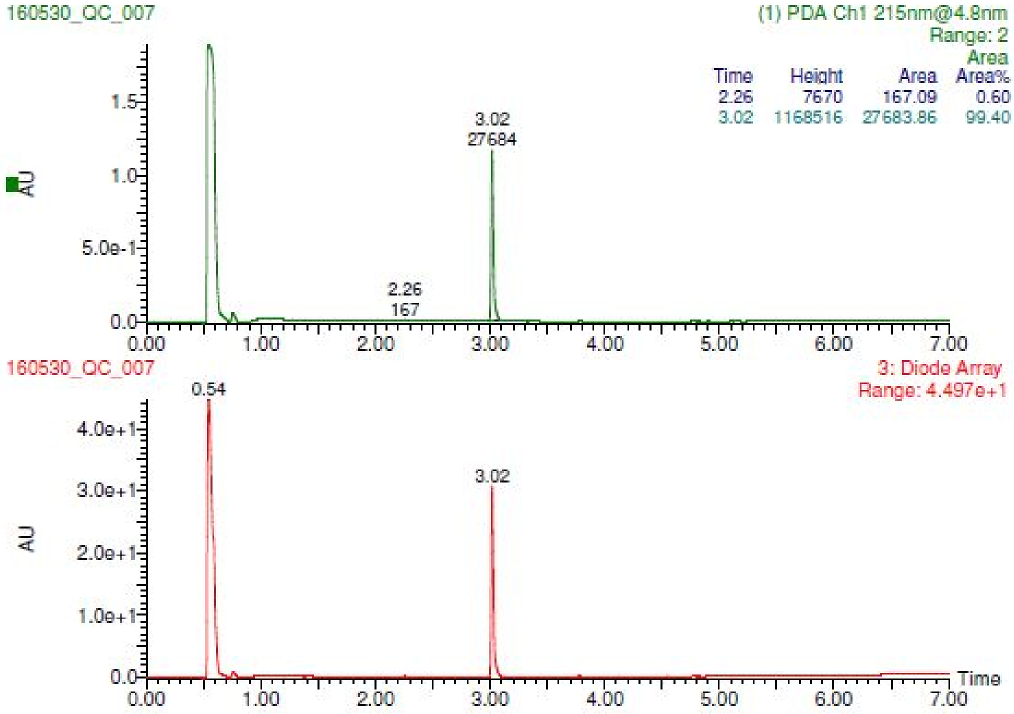

Compound 22

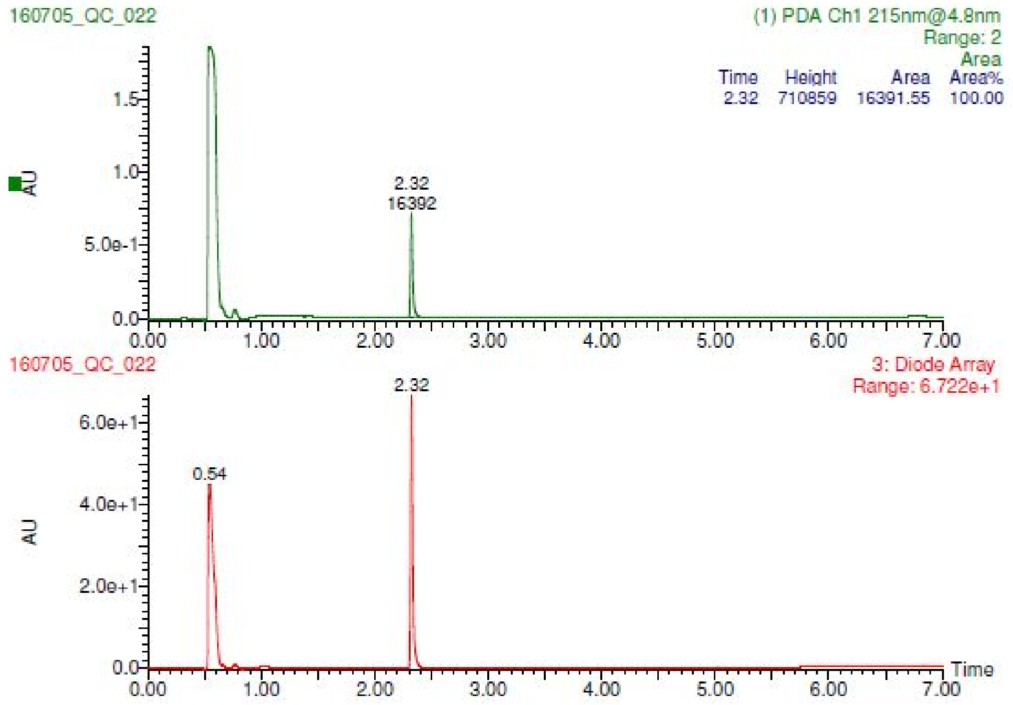

Compound 23

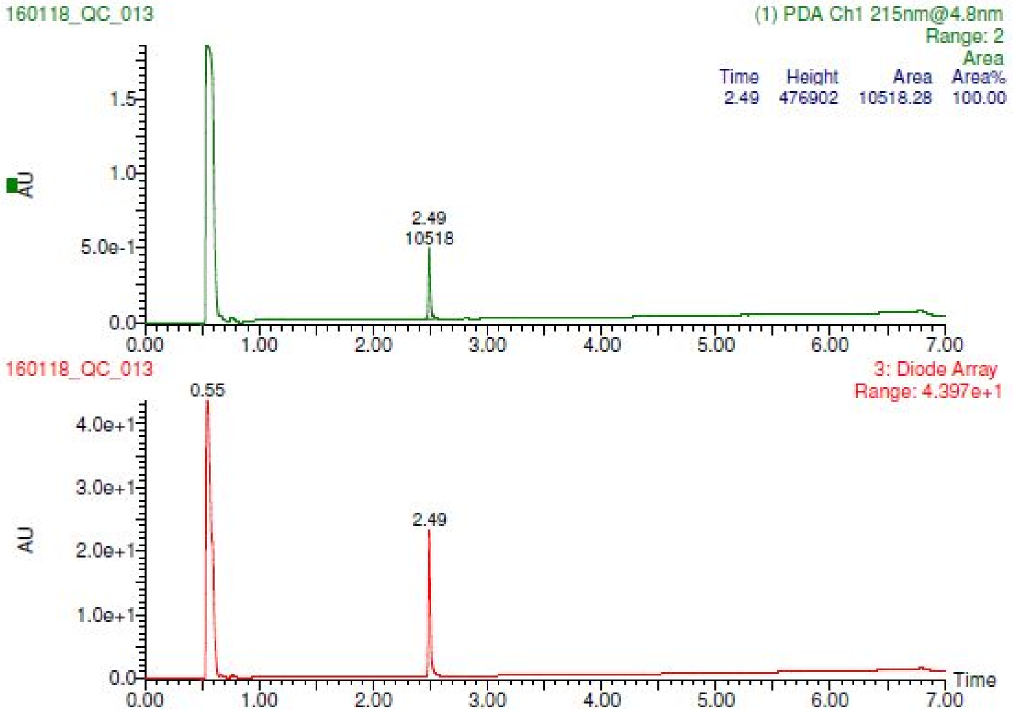

Compound 24

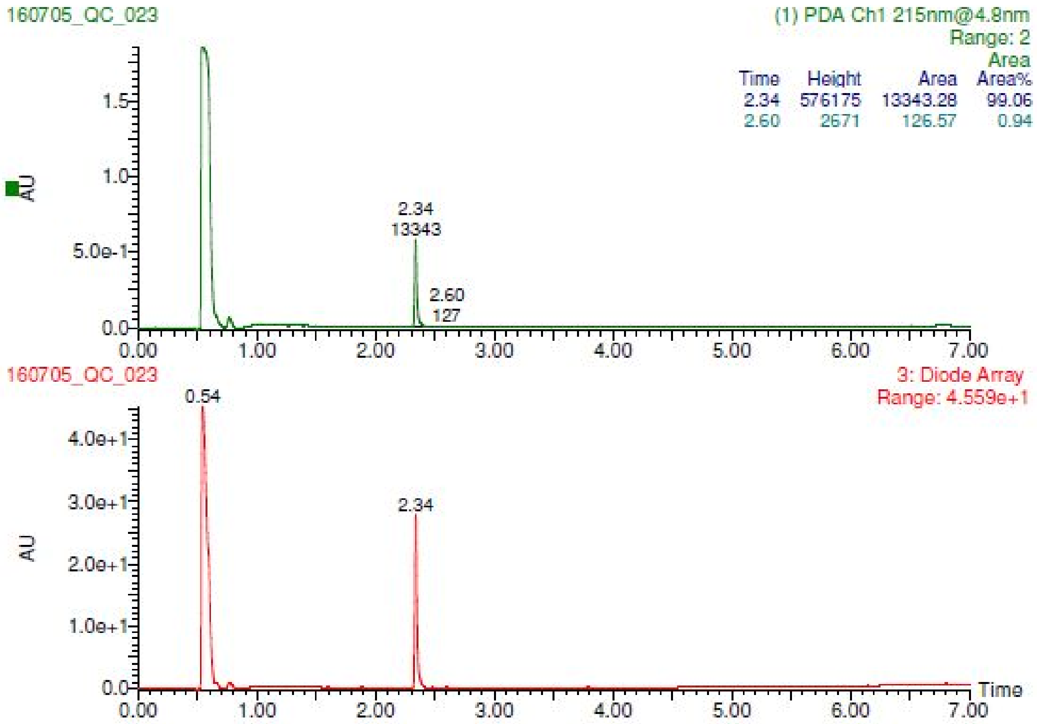

Compound 25

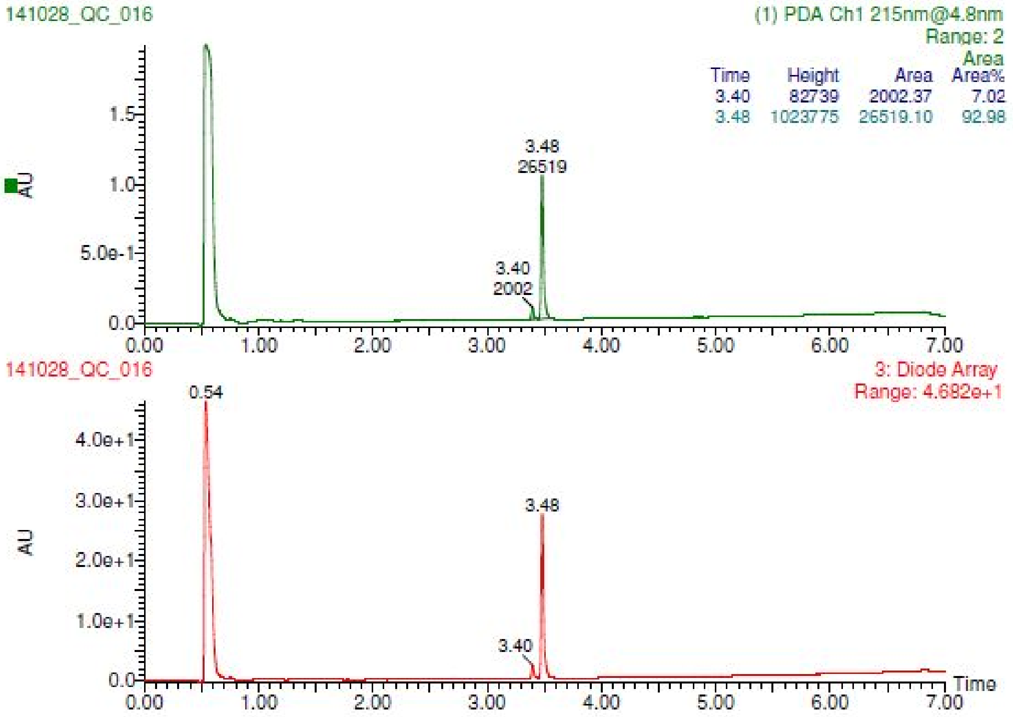

Compound 26

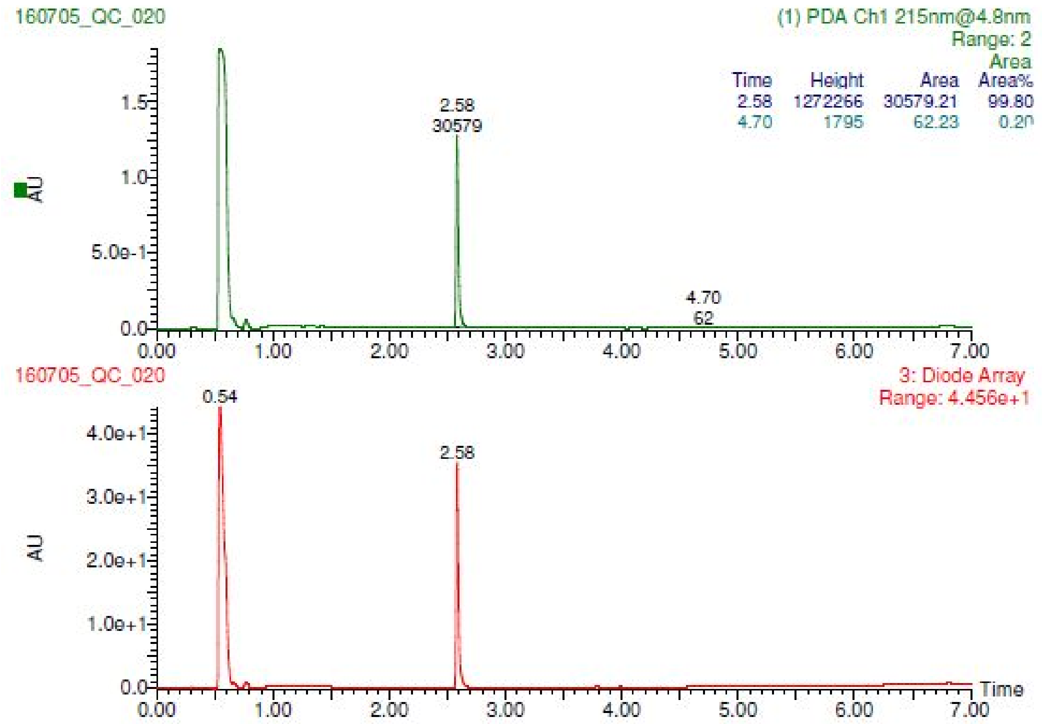

Compound 27

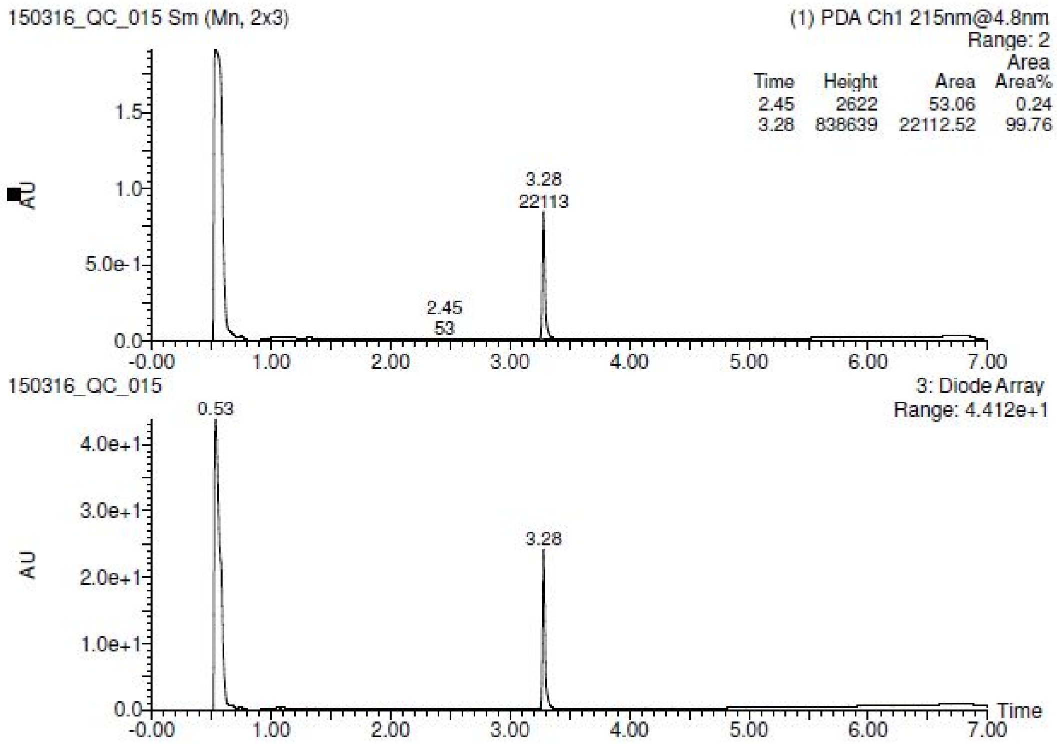

Compound 28

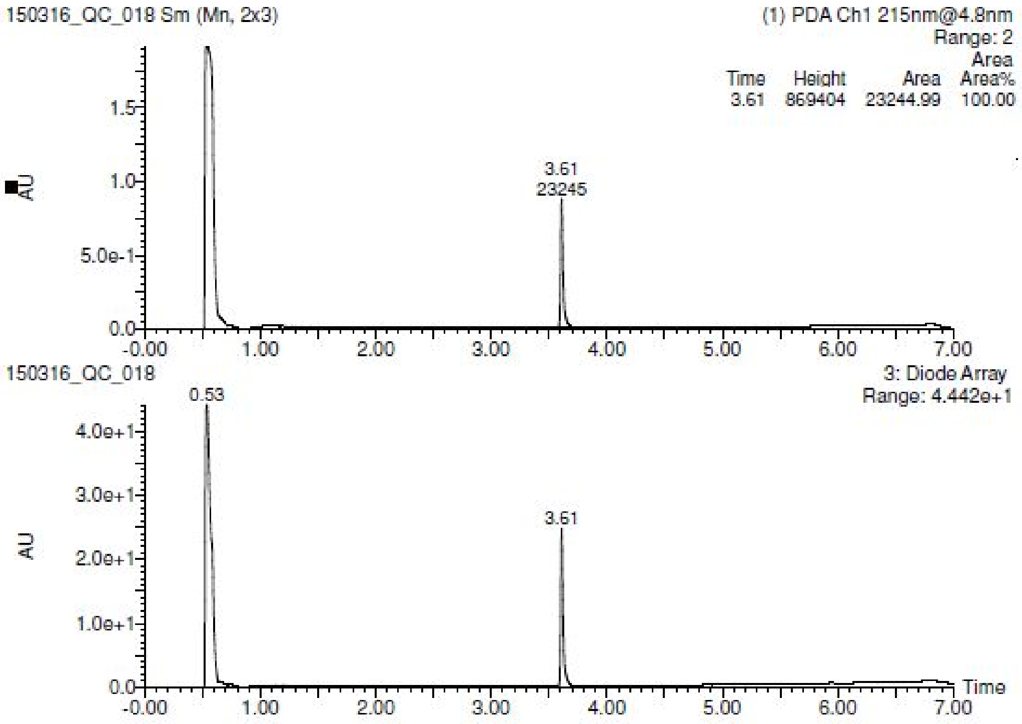

Compound 29

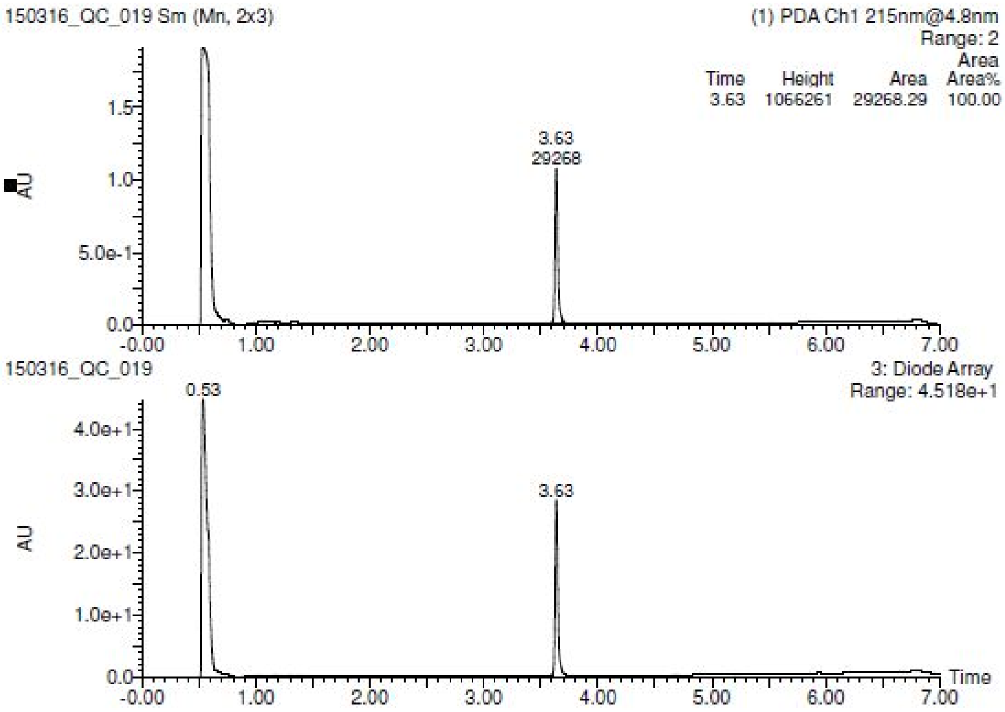

Compound 30

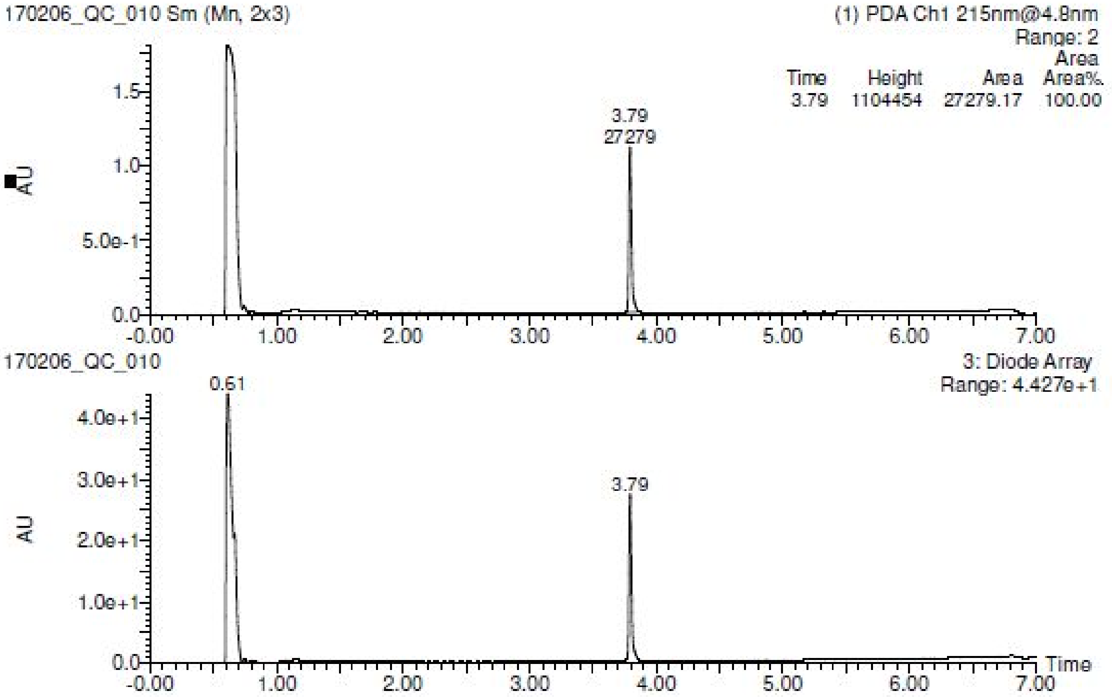

Compound 31

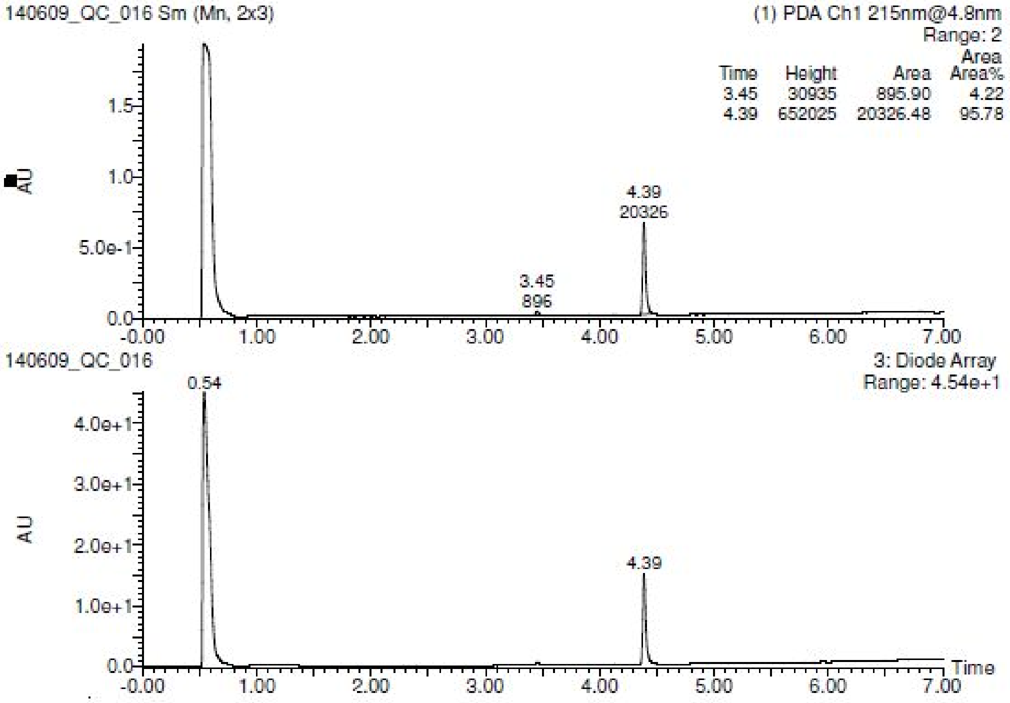

Compound 32

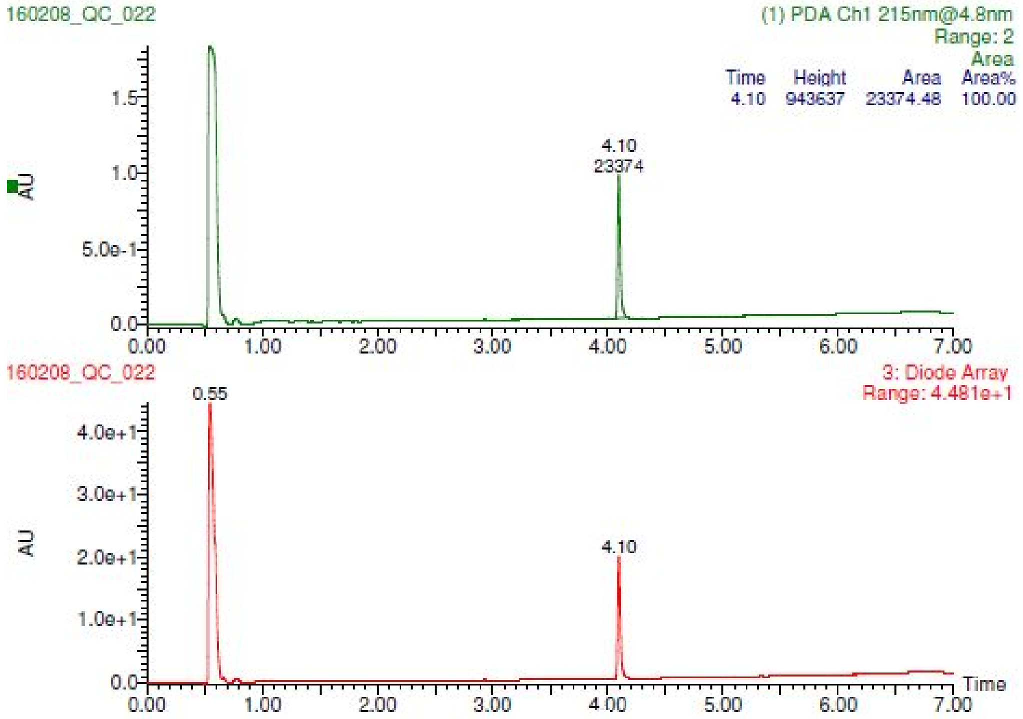

Compound 33

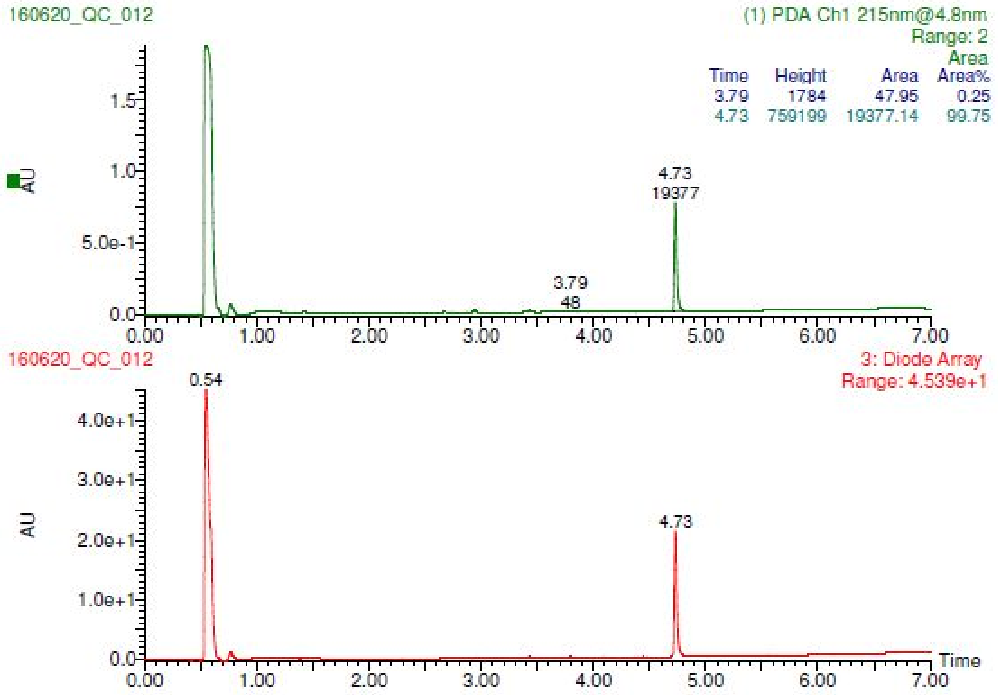

Compound 34

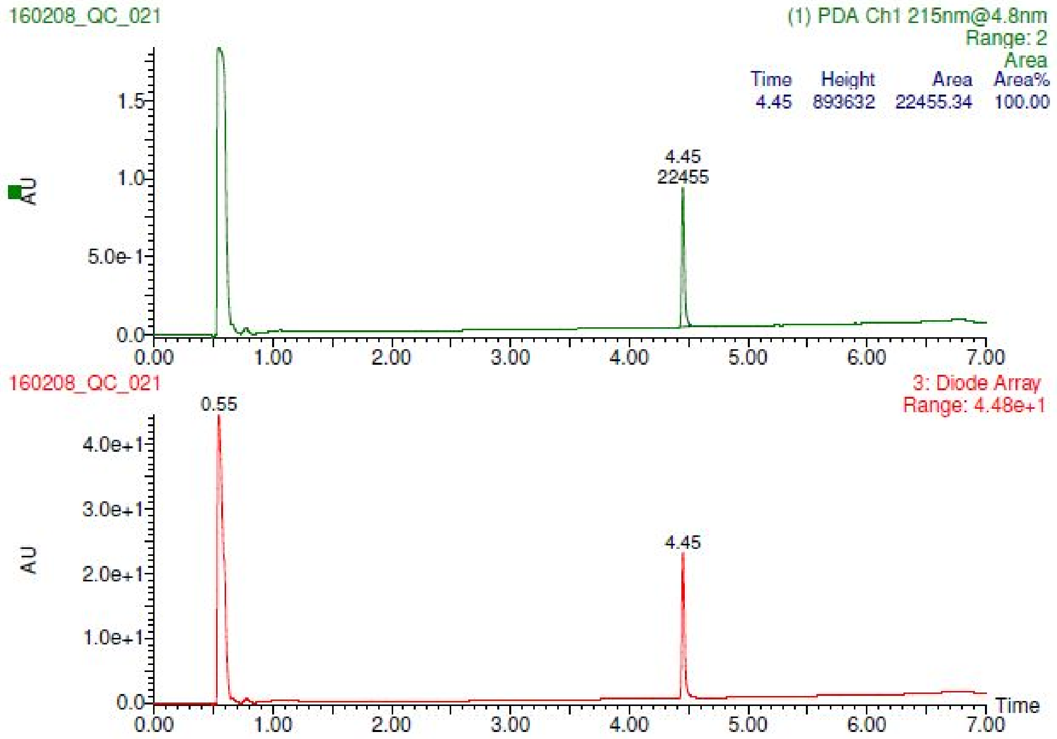

Compound 35

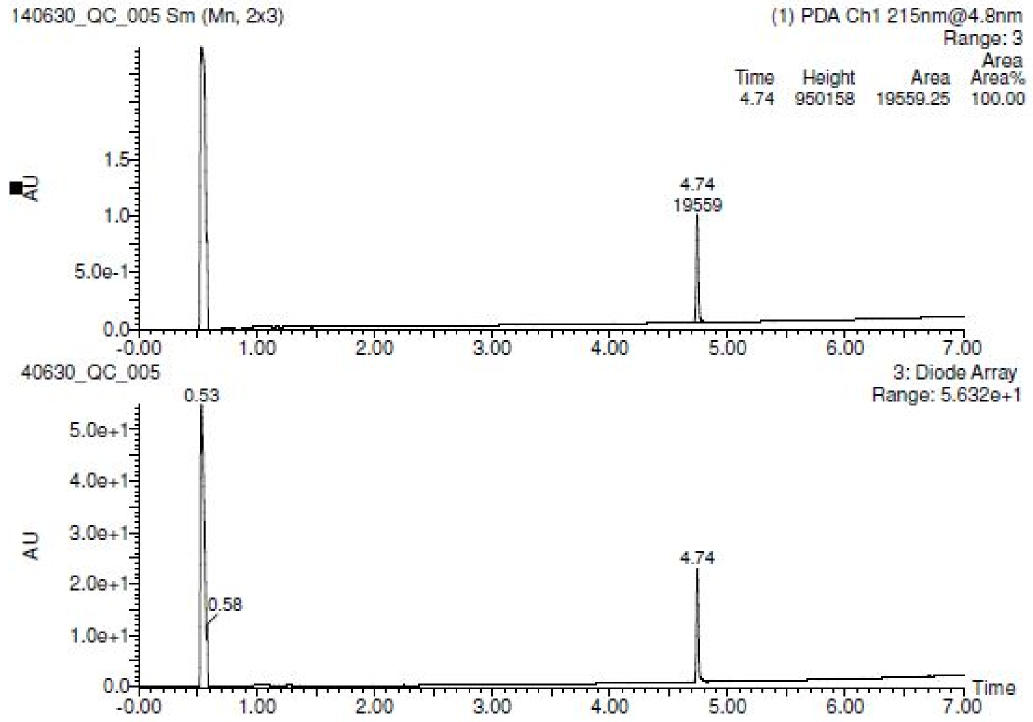

Compound 36

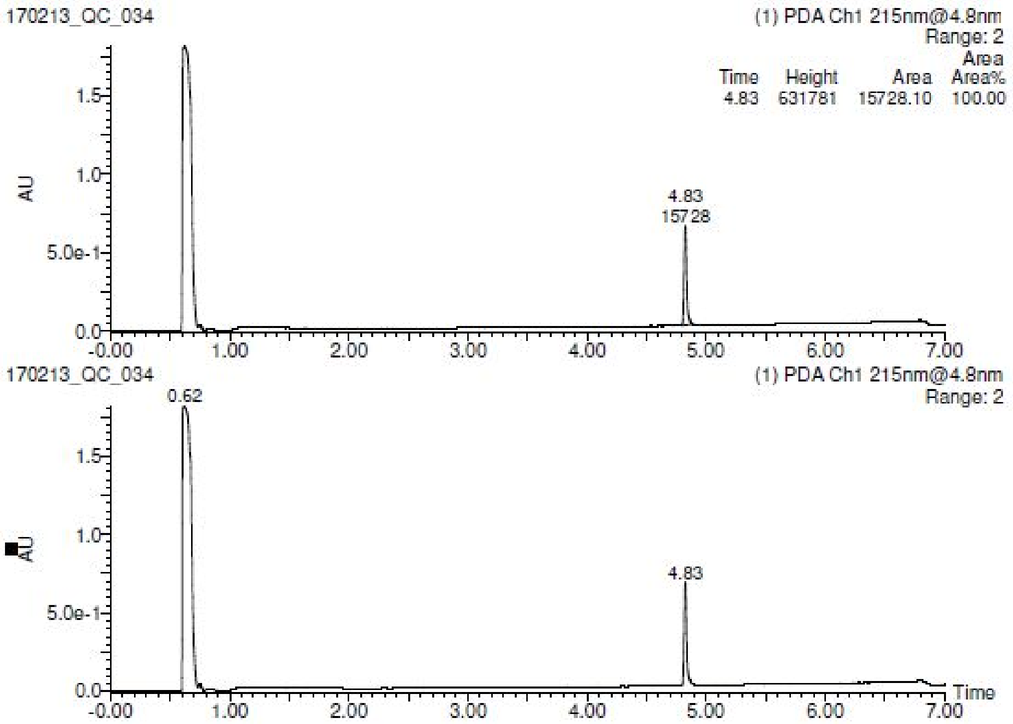

Compound 37

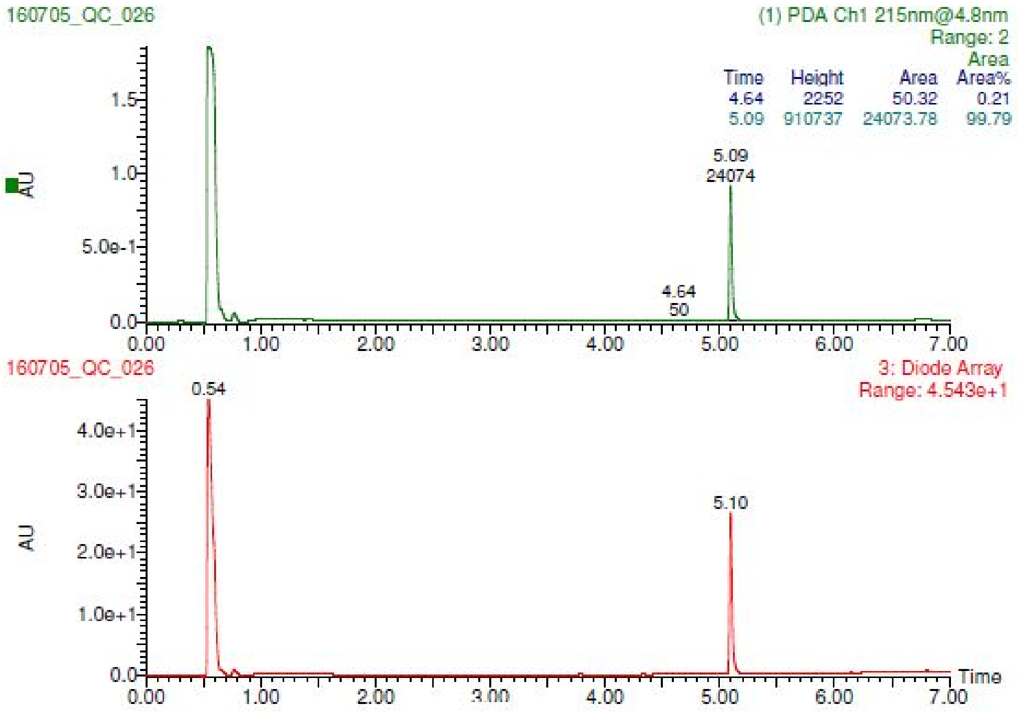

Compound 38

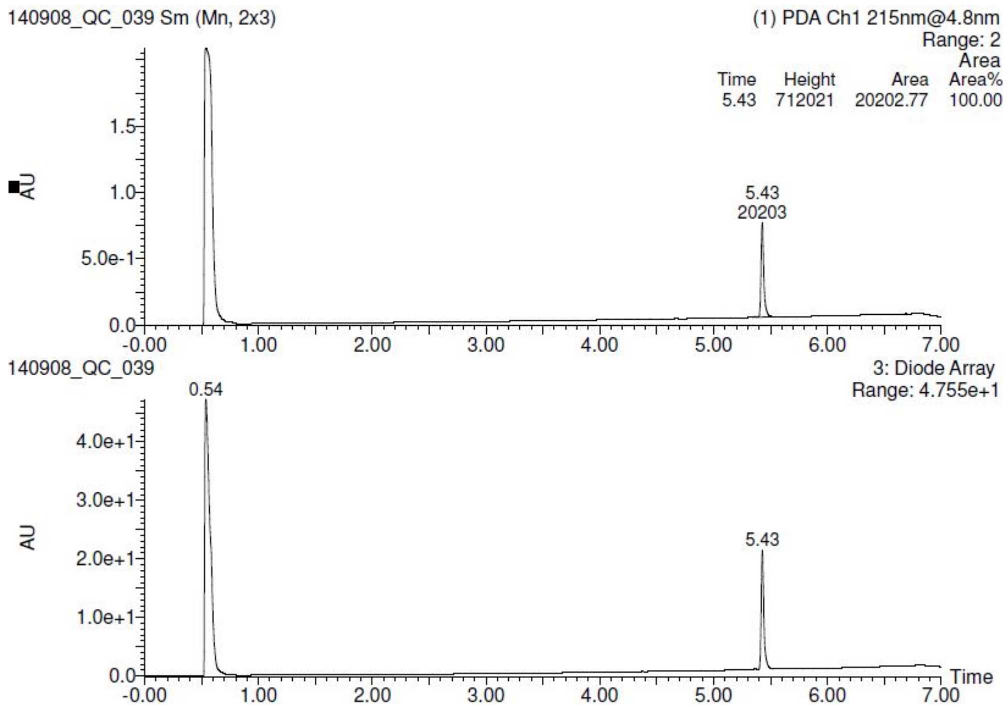

Compound 39

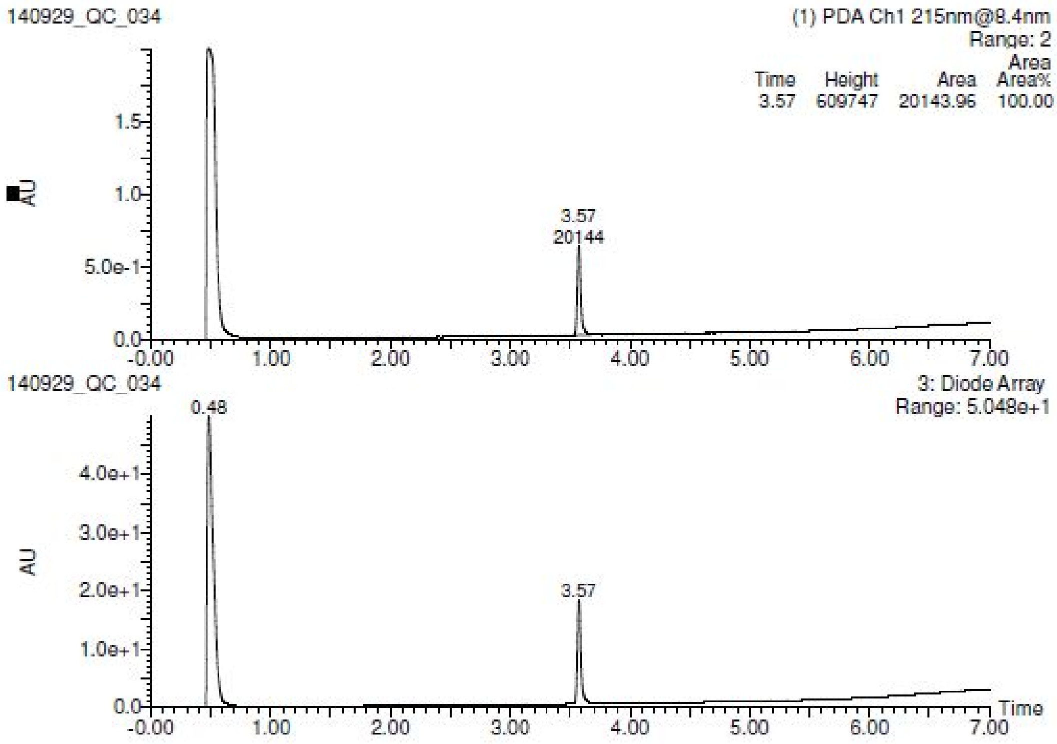

Compound 40

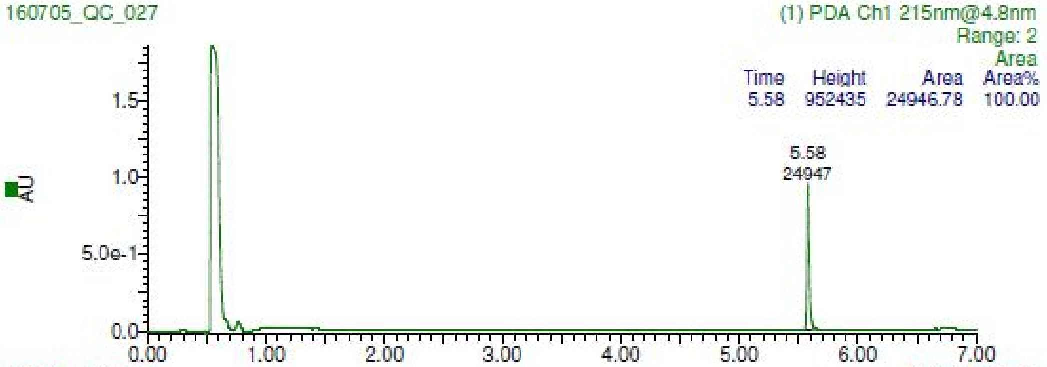

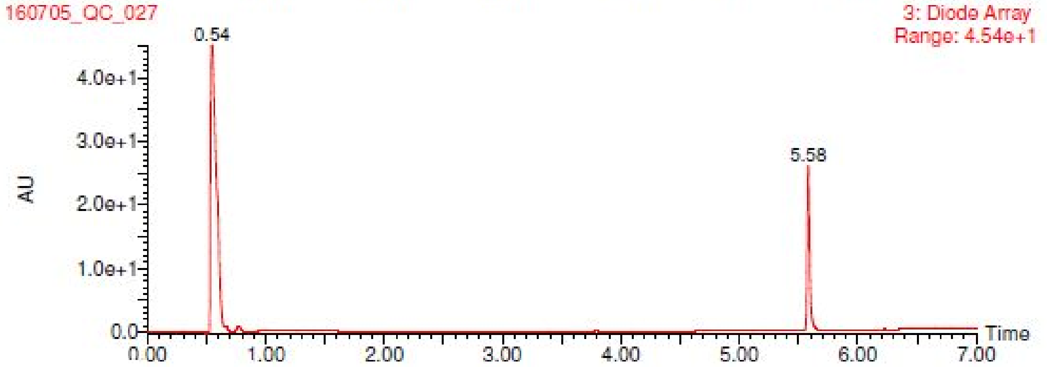

Compound 41

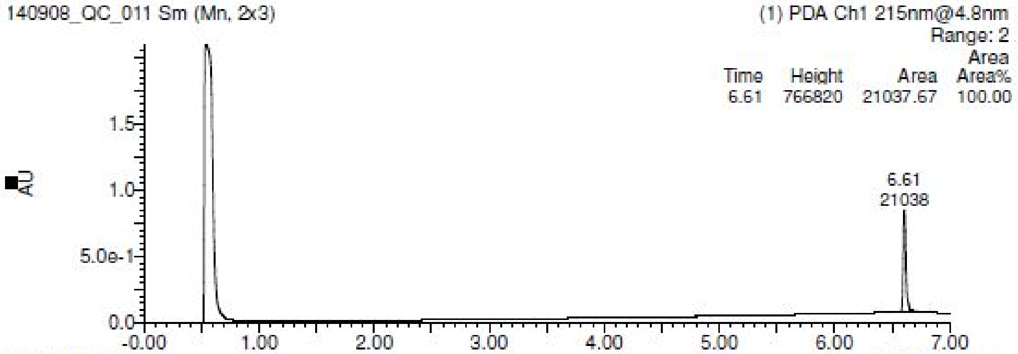

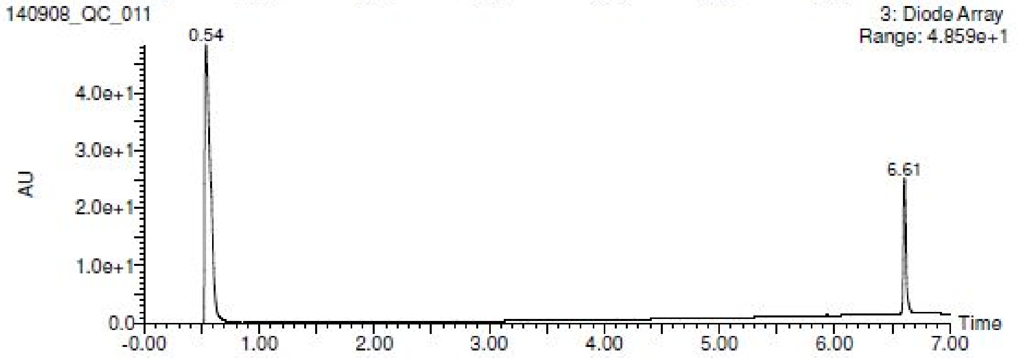

Compound 42

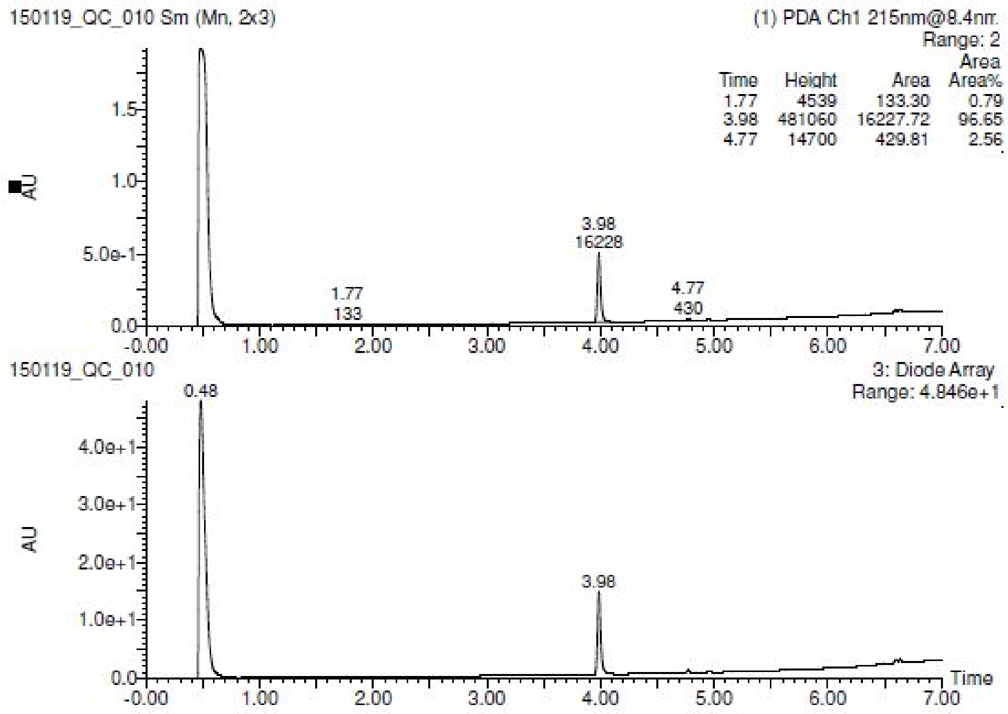

Compound 43

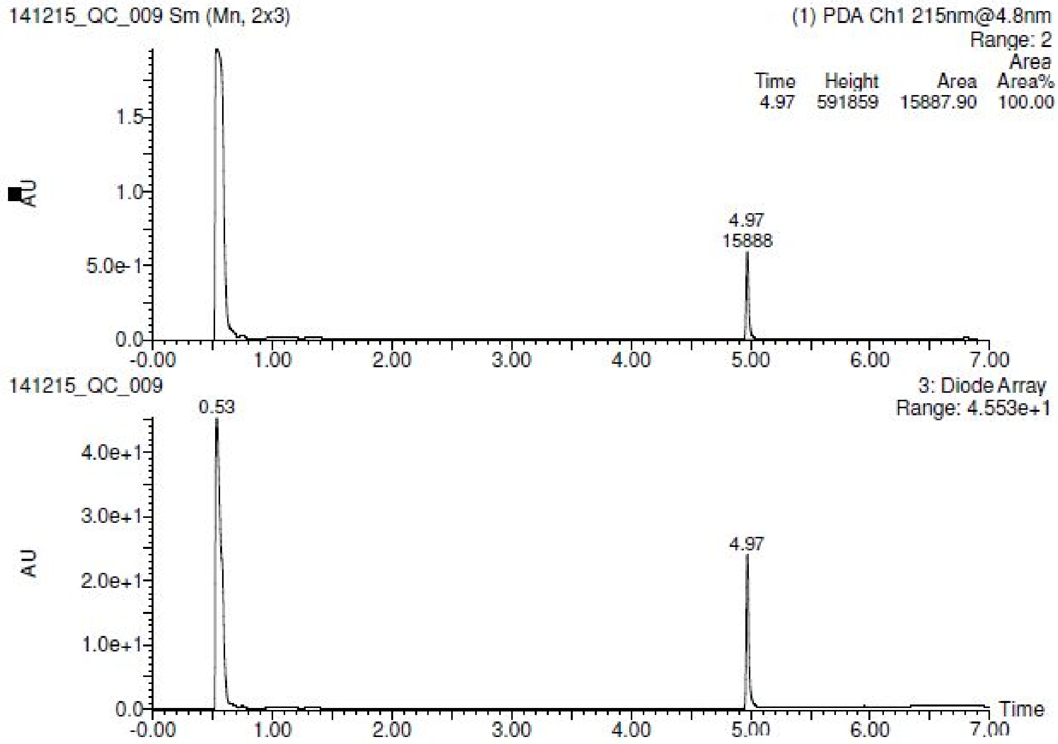

Compound 44

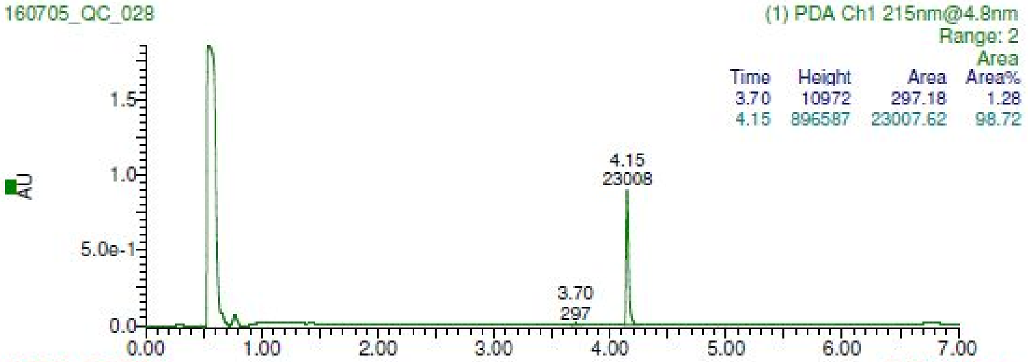

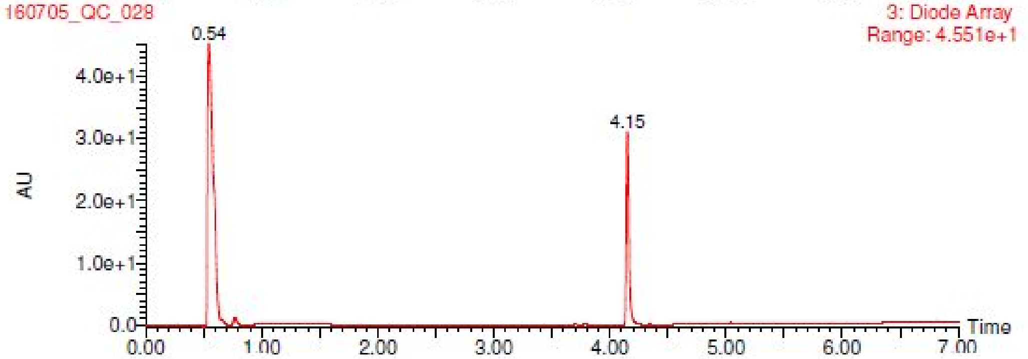

Compound 45

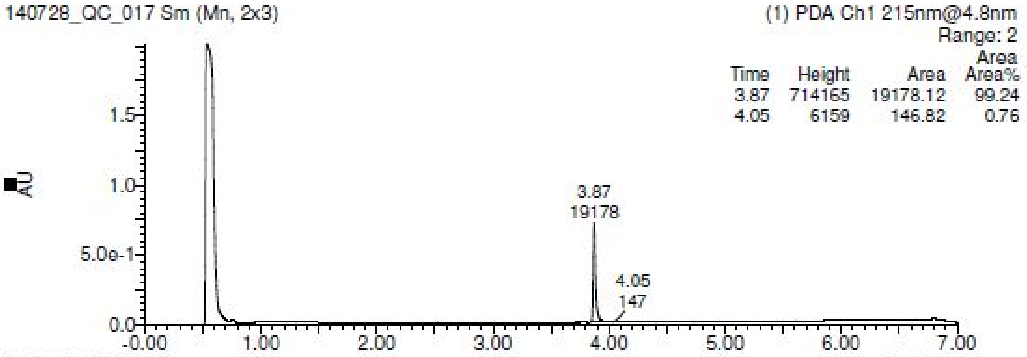

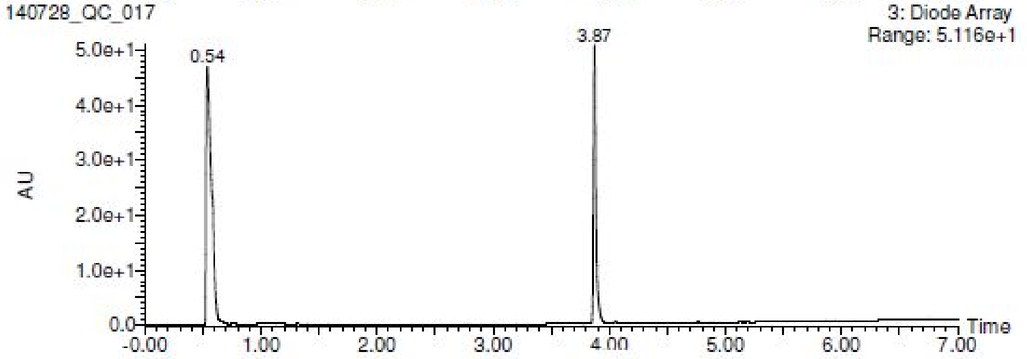

Compound 46

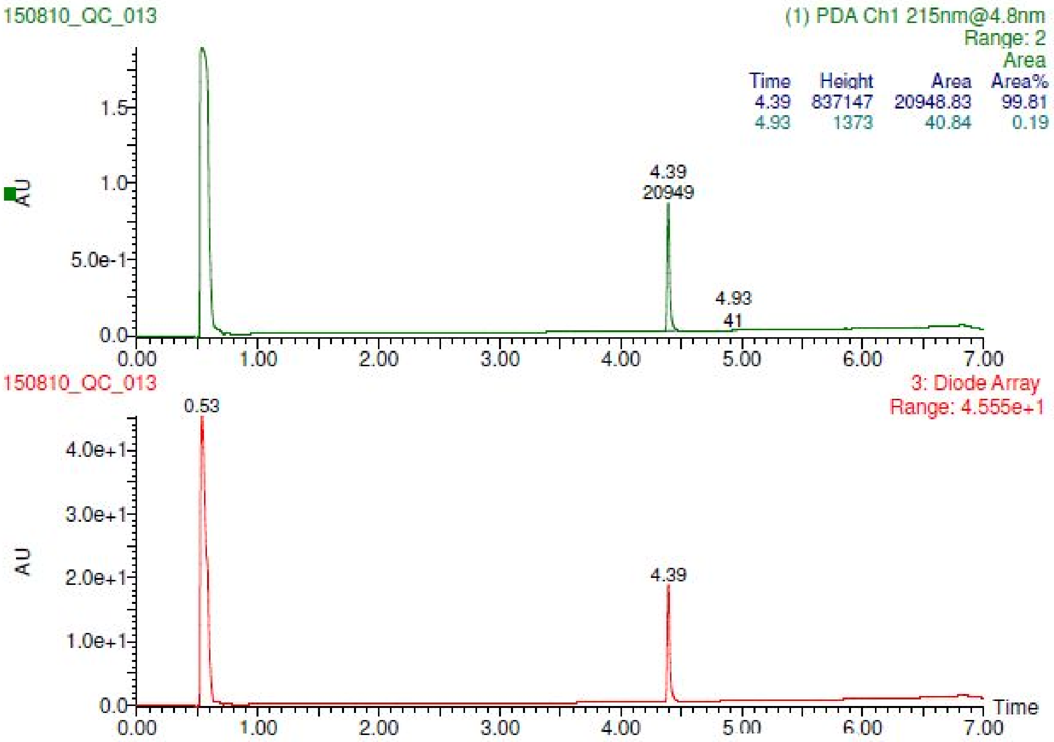

Compound 47

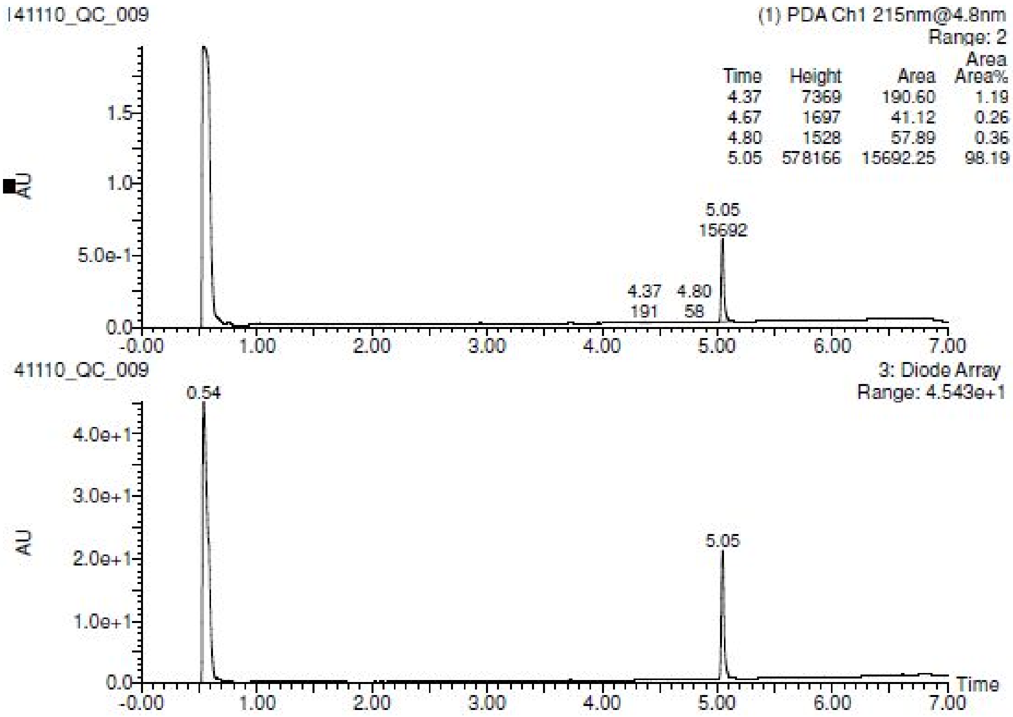

Compound 48

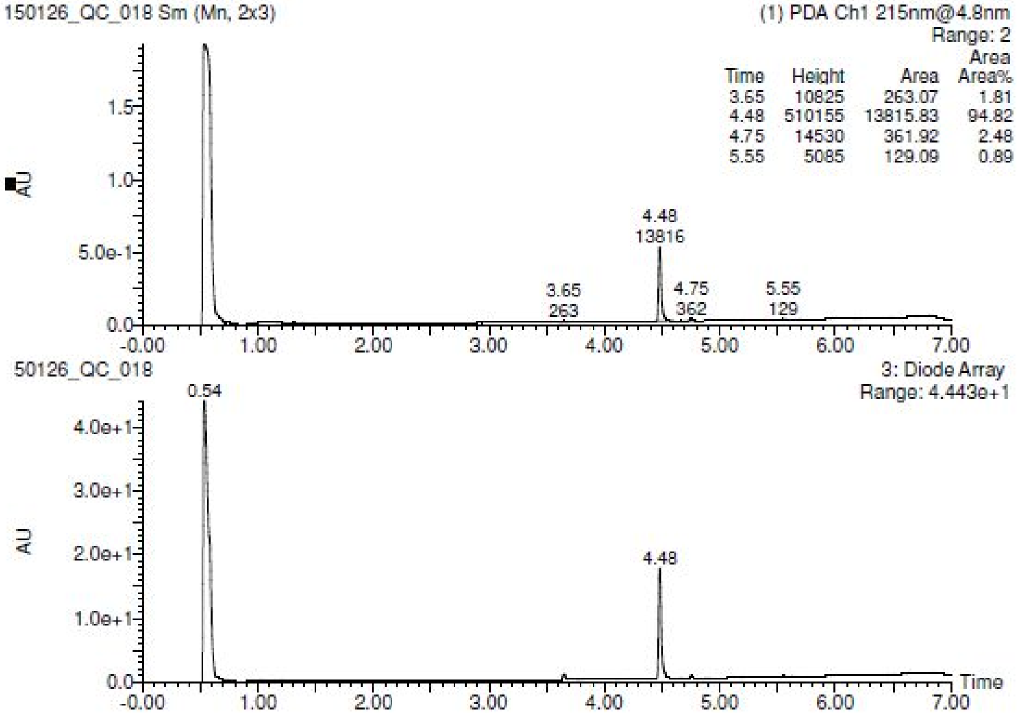

Compound 49

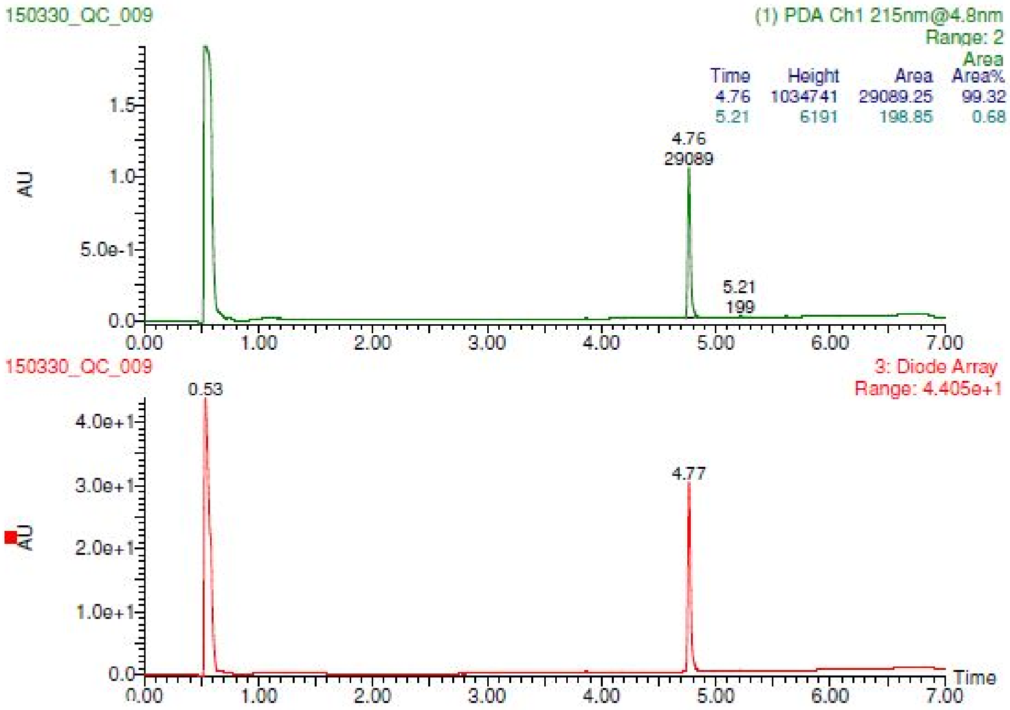

Compound 50

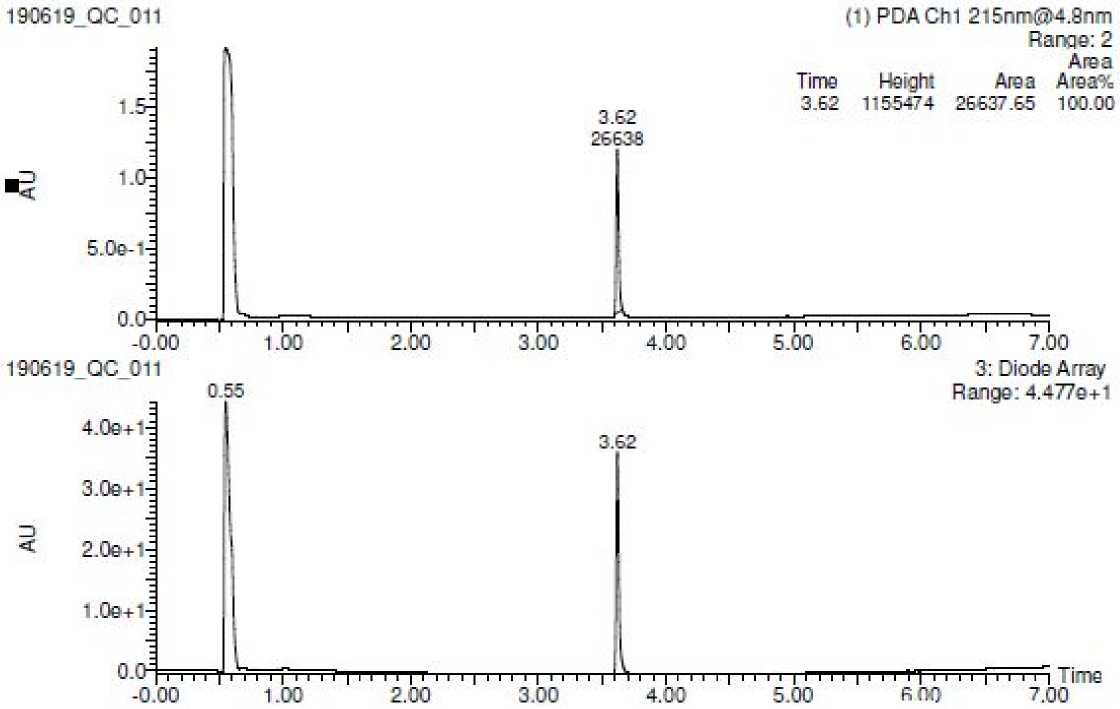

190619_QC_011
(1) PDA Ch1 215nm@4.8nm
Range: 2
Time Height Area Area%
3.62 1155474 26637.65 100.00
3.62
26638
AU
190619_QC_011
3: Diode Array
Range: 4.477e+1
0.55
3.62
AU
Time

3. Structure and LipE data of selected, novel *h*-NAAA inhibitors

Table S1. Human NAAA inhibitory activity (IC_{50} and pIC_{50}), clogP and LipE of pyrazole-sulfonamides **1**,**39**,**47**-**50**.

Compound	Structure	IC_{50} (μM)^a / pIC_{50}	clogP^b	LipE^c
**1**	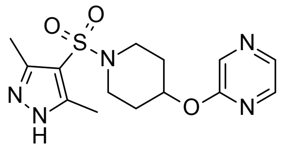	1.09 ± 0.163<br>5.96	-0.34	6.30
**39 (ARN16186)**	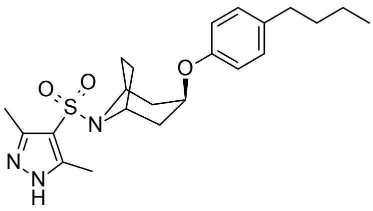	0.023 ± 0.004<br>7.64	3.79	3.84
**47**	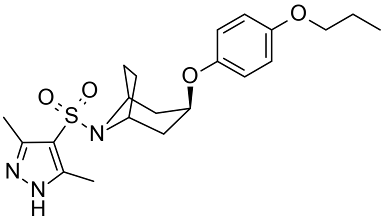	0.45 ± 0.074<br>6.35	2.79	3.55
**48**	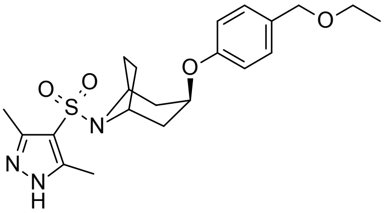	0.016 ± 0.002<br>7.79	2.09	5.70
**49**	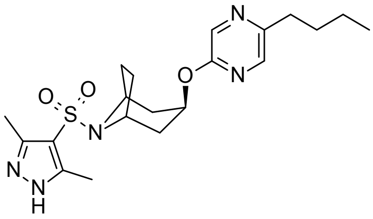	0.017 ± 0.001<br>7.77	2.04	5.73
**50 (ARN19689)**	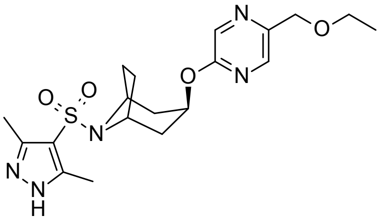	0.042 ± 0.002<br>7.38	0.55	6.83

^{a}*h*-NAAA (fluo.), data are expressed as mean ± SD (n ≥ 3); ^{b}cLogP computed using PipelinePilot WebPort 2017; ^{c}LipE = pIC_{50} – cLogP.[3]

4. Known *h*-NAAA inhibitors as reference compounds

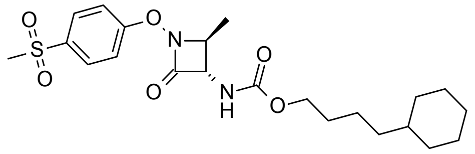

ARN15393 (*β*-lactam covalent NAAA inhibitor)[4]

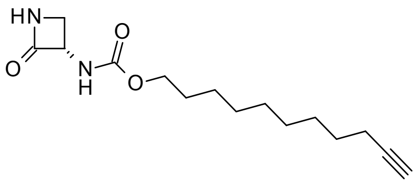

ARN14686 (specific *h*-NAAA activity based probe)[5]

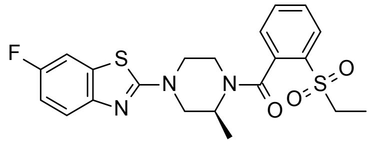

ARN19702 (non-covalent *h*-NAAA inhibitor)[6,7]

Figure S1. Structure and code numbers of known *h*-NAAA inhibitors.

5. Docking study of compound 50 in human NAAA binding site

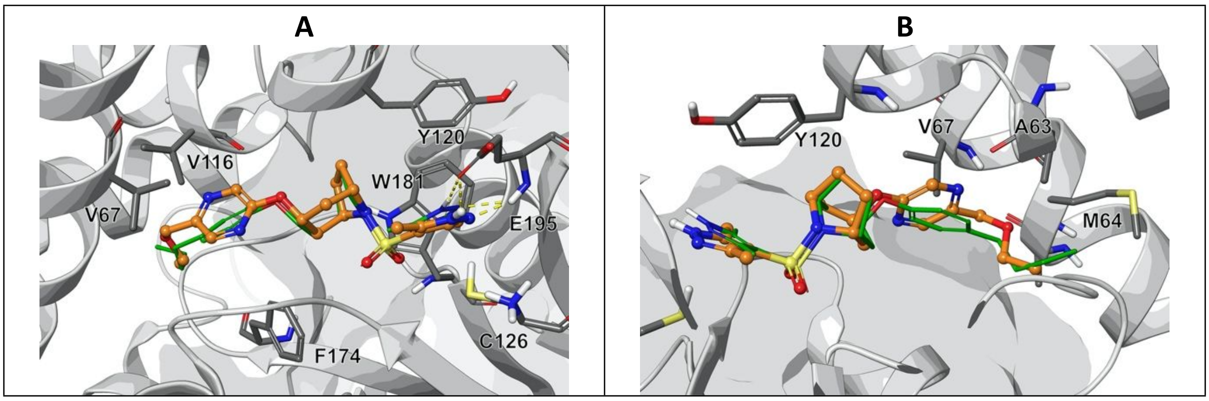

Figure S2. Predicted bound conformations of compound **50** (***ARN19689***) at the binding site of human NAAA (*PDB ID: 6DXX*).[7,8] The protein structure is reported in white ribbon. Residues interacting with the inhibitors are reported in stick representation with grey carbons and labelled explicitly. The predicted bound conformation of compound **39** (***ARN16186***) is reported in green for reference. *A)* Bound conformation of **50** in balls and sticks representation (orange carbons). *B)* Rear view of the bound conformation of **50** in balls and sticks representation (orange carbons).

6. *In-vivo* characterization of compound 50

Animal models. Male C57BL/6 mice, weighing 22-24 g, were used (Charles River). All procedures were performed in accordance with the Ethical Guidelines of European Communities Council (Directive 2010/63/EU of 22 September 2010) and accepted by the Italian Ministry of Health. All efforts were made to minimize animal suffering and to use the minimal number of animals required to produce reliable results, according to the "3Rs concept". Animals were group-housed in ventilated cages and had free access to food and water. They were maintained under a 12-hour light/dark cycle (lights on at 8:00 am) at controlled temperature (21 °C ± 1°C) and relative humidity (55% ± 10%).

Pharmacokinetic studies. Compound **50** (***ARN16189***) was administered orally (*p.o.*) and intravenously (*i.v.*) to C57BL/6 male mice at 10 and 3 mg/kg. The vehicle used was: PEG400/Tween 80/Saline solution at 10/10/80 % in volume respectively. Three animals per each time point were treated. Blood samples at 0, 15, 30, 60, 120, 240, and 480 min after administration were collected for *p.o.* arm. Blood samples at 0, 5, 15, 30, 60, 120 and 240 min after administration were collected for *i.v.* arm. Plasma was separated from blood by centrifugation for 15 min at 1500 rpm a 4 °C, transferred to eppendorf tubes and frozen (-80 °C). Control animals, treated with vehicle only, were also included in the experimental protocol.

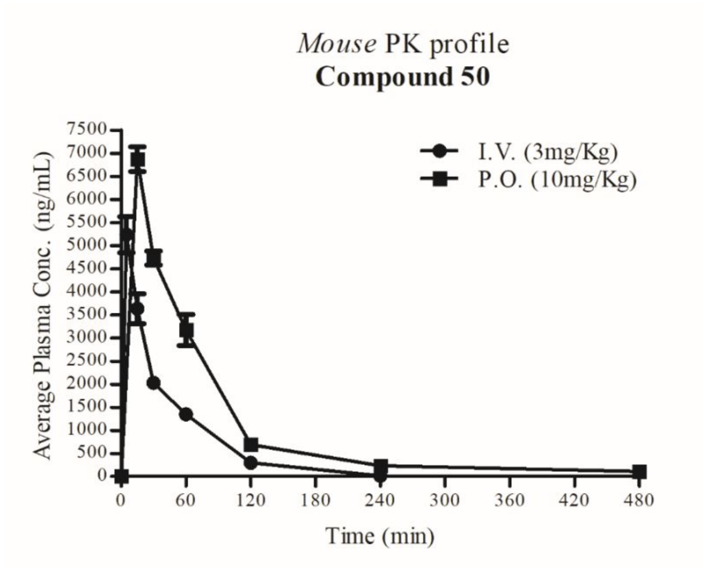

Figure S3. Pharmacokinetic profile of compound **50** after *p.o.* and *i.v.* administration in male C57BL/6 mice (*n = 3* per dose).

7. References

1. Wu, P. S.; Otting, G. Rapid Pulse Length Determination in High-Resolution NMR. *J. Magn. Reson.* **2005,** *176*, 115-119.

2. Wider, G.; Dreier, L. Measuring Protein Concentrations by NMR Spectroscopy. *J. Am. Chem. Soc.* **2006,** *128*, 2571-2576.

3. Freeman-Cook, K. D.; Hoffman, R. L.; Johnson, T. W. Lipophilic Efficiency: the Most Important Efficiency Metric in Medicinal Chemistry. *Future Med. Chem.* **2013**, 5, 113-115.

4. Petracca, R.; Ponzano, S.; Bertozzi, S. M.; Sasso, O.; Piomelli, D.; Bandiera, T.; Bertozzi, F. Progress in the development of *beta*-lactams as *N*-Acylethanolamine Acid Amidase (NAAA) inhibitors: Synthesis and SAR Study of New, Potent *N-O*-substituted Derivatives. *Eur. J. Med. Chem.* **2017**, *126*, 561-575.

5. Romeo, E.; Ponzano, S.; Armirotti, A.; Summa, M.; Bertozzi, F.; Garau, G.; Bandiera, T.; Piomelli, D. Activity-Based Probe for *N*-Acylethanolamine Acid Amidase. *ACS Chem. Biol.* **2015**, *10*, 2057-2064.

6. Migliore, M.; Pontis, S.; Fuentes de Arriba, A. L.; Realini, N.; Torrente, E.; Armirotti, A.; Romeo, E.; Di Martino, S.; Russo, D.; Pizzirani, D.; Summa, M.; Lanfranco, M.; Ottonello, G.; Busquet, P.; Jung, K. M.; Garcia-Guzman, M.; Heim, R.; Scarpelli, R.; Piomelli, D. Second-Generation Non-Covalent NAAA Inhibitors are Protective in a Model of Multiple Sclerosis. *Angew. Chem., Int. Ed. Engl.* **2016**, *55*, 11193-11197.

7. Gorelik, A.; Gebai, A.; Illes, K.; Piomelli, D.; Nagar, B. Molecular Mechanism of Activation of the Immunoregulatory Amidase NAAA. *Proc. Natl. Acad. Sci. USA.* **2018**, *115*, E10032-E10040.

8. Sherman, W.; Day, T.; Jacobson, M. P.; Friesner, R. A.; Farid, R. Novel Procedure for Modeling Ligand/Receptor Induced Fit Effects. *J. Med. Chem.* **2006**, *49*, 534-553.
